# New Multilocus Sequence Typing Scheme for Enterococcus faecium Based on Whole Genome Sequencing Data

**DOI:** 10.1128/spectrum.05107-22

**Published:** 2023-06-12

**Authors:** Matej Bezdicek, Jana Hanslikova, Marketa Nykrynova, Kristyna Dufkova, Iva Kocmanova, Petra Kubackova, Jiri Mayer, Martina Lengerova

**Affiliations:** a Department of Internal Medicine - Haematology and Oncology, University Hospital Brno, Brno, Czech Republic; b Department of Internal Medicine - Haematology and Oncology, Faculty of Medicine, Masaryk University, Brno, Czech Republic; c Department of Biomedical Engineering, Brno University of Technology, Brno, Czech Republic; d Department of Clinical Microbiology and Immunology, University Hospital Brno, Brno, Czech Republic; Health Canada

**Keywords:** *Enterococcus faesium*, multilocus sequence typing, clonal complex, epidemiology, whole genome sequenging

## Abstract

The MLST scheme currently used for Enterococcus faecium typing was designed in 2002 and is based on putative gene functions and Enterococcus faecalis gene sequences available at that time. As a result, the original MLST scheme does not correspond to the real genetic relatedness of E. faecium strains and often clusters genetically distant strains to the same sequence types (ST). Nevertheless, typing has a significant impact on the subsequent epidemiological conclusions and introduction of appropriate epidemiological measures, thus it is crucial to use a more accurate MLST scheme. Based on the genome analysis of 1,843 E. faecium isolates, a new scheme, consisting of 8 highly discriminative loci, was created in this study. These strains were divided into 421 STs using the new MLST scheme, as opposed to 223 STs assigned by the original MLST scheme. The proposed MLST has a discriminatory power of D = 0.983 (CI95% 0.981 to 0.984), compared to the original scheme’s D = 0.919 (CI95% 0.911 to 0.927). Moreover, we identified new clonal complexes with our newly designed MLST scheme. The scheme proposed here is available within the PubMLST database. Although whole genome sequencing availability has increased rapidly, MLST remains an integral part of clinical epidemiology, mainly due to its high standardization and excellent robustness. In this study, we proposed and validated a new MLST scheme for E. faecium, which is based on genome-wide data and thus reflects the tested isolates’ more accurate genetic similarity.

**IMPORTANCE** Enterococcus faecium is one of the most important pathogens causing health care associated infections. One of the main reasons for its clinical importance is a rapidly spreading resistance to vancomycin and linezolid, which significantly complicates antibiotic treatment of infections caused by such resistant strains. Monitoring the spread and relationships between resistant strains causing severe conditions represents an important tool for implementing appropriate preventive measures. Therefore, there is an urgent need to establish a robust method enabling strain monitoring and comparison at the local, national, and global level. Unfortunately, the current, extensively used MLST scheme does not reflect the real genetic relatedness between individual strains and thus does not provide sufficient discriminatory power. This can lead directly to incorrect epidemiological measures due to insufficient accuracy and biased results.

## INTRODUCTION

Enterococcus faecium is an important opportunistic pathogen frequently causing infections associated with hospital care. The clinical importance of E. faecium is directly related to its naturally low susceptibility to a wide range of antimicrobial agents, including penicillin and ampicillin administered in low doses, aminoglycosides, sulfonamides, and cephalosporins, as well as a rapid spread of strains resistant to vancomycin, linezolid, or tigecycline (1).

Molecular typing represents one of the tools to monitor and control the spread of multidrug-resistant or also, on the contrary, sensitive clones in hospitals. Currently, clinical typing is shifting away from methods, such as PFGE and rep-PCR, due to their laboriousness and low to medium discriminatory power (2). Simultaneously, whole-genome sequencing (WGS) is being widely promoted and is progressing toward becoming a new golden standard in molecular typing. Acquired WGS data can be used for many *in silico* typing analyses e.g., multilocus sequence typing (MLST). Despite its lower discriminative capabilities compared to WGS, MLST still has its place in epidemiology, as it enables easy comparison of bacterial strains’ relatedness on both a local and global level. As for E. faecium, the currently used MLST scheme (3) is based on putative gene functions and Enterococcus faecalis gene sequences available at that time. Even though several previously published studies have pointed out the discrepancies between MLST and WGS data (4–6), the MLST scheme itself has not been updated and adapted to the current knowledge of the E. faecium genome.

The aim of this study is to propose a new MLST scheme based on whole genome data obtained by sequencing the local E. faecium population, complemented with available E. faecium genomes sequenced worldwide and obtained from genomic databases. As a result, this scheme reflects the divergence of the E. faecium populations based on the differences in the whole genome as accurately as possible and can be used globally.

## RESULTS

**FIG 1:**
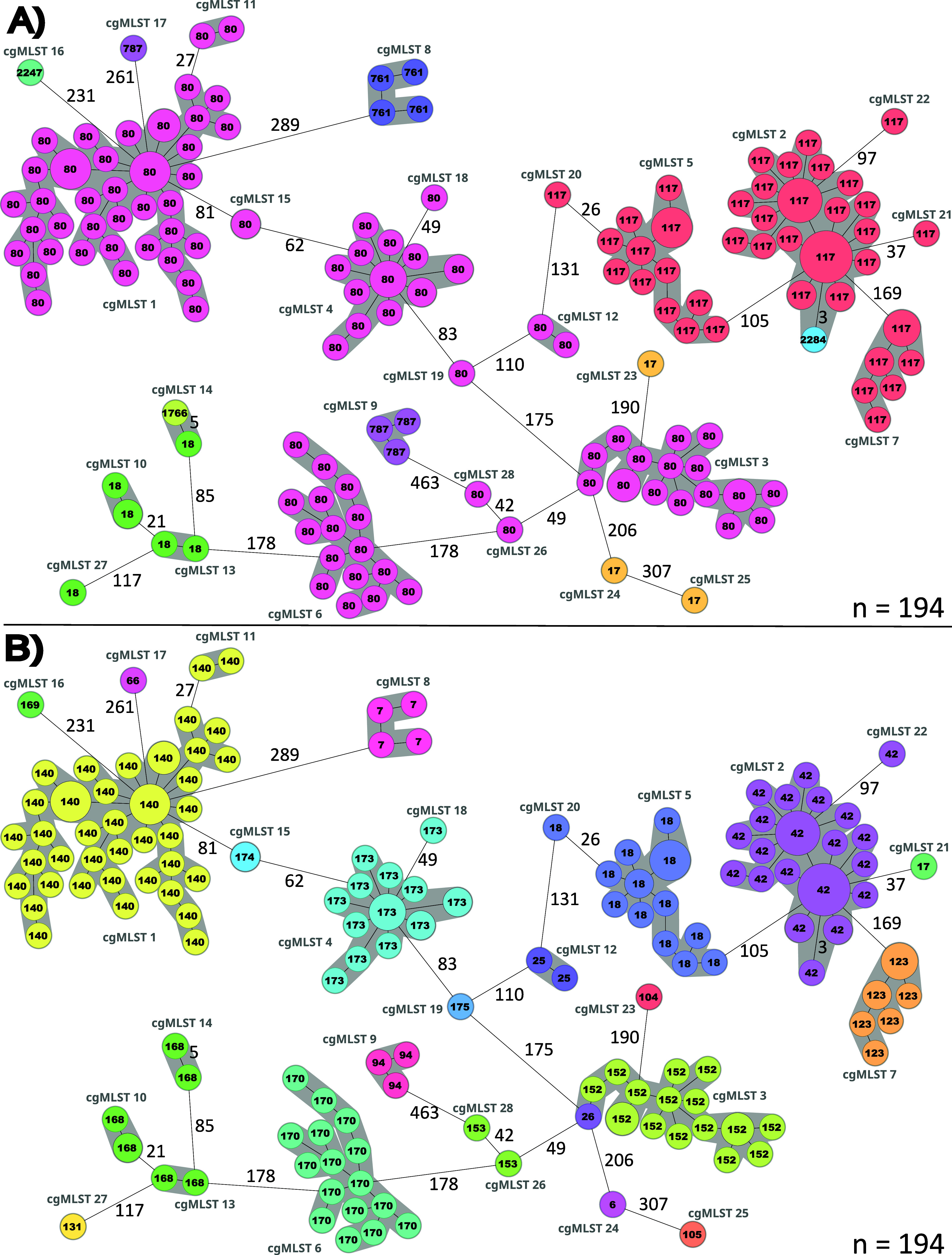
Enterococcus faecium strain clustering based on WGS, original MLST (A) and new MLST (B) schemes. The minimum spanning tree for isolates (*n* = 194) collected within University Hospital Brno was constructed using Ridom SeqSphere + version 8.5 software. Numbers listed between nodes represent allelic differences (only distances of 20 or more differences are displayed), while the length of the branches in this figure does not correlate with the number of differences and therefore is not informative due to the relatedness of the strains. Core genome MLST clusters (cgMLST) are defined as groups of strains with a maximum of 20 allelic differences. Node numbers and colors represent STs of original (A) and new (B) MLST schemes. The node size represents the number of isolates, where the smallest size represents 1 isolate, the largest size represents 8 isolates.

Since March 2017, prospective screening of the vancomycin resistant E. faecium population isolated at the Department of Internal Medicine - Hematology and Oncology (University Hospital Brno, Czech Republic) has been conducted. All vancomycin resistant E. faecium isolates obtained within this screening period were typed using a high-resolution mini-MLST based typing method (7). Based on the obtained results, where more than 90% of the strains belonged to the same melting types (MelT), a long-lasting outbreak was suspected. This was also indicated by the MLST results, as the majority of the strains belonged to ST80 and ST117 (Fig. 1).

To confirm or refute the suspicion of a long-lasting outbreak, 194 E. faecium isolates collected at the University Hospital Brno, Czech Republic were analyzed using both original MLST scheme (3), and core genome MLST (cgMLST) analysis (a total of 1,423 analyzed gene targets). The isolates were divided into 9 different STs using the original MLST scheme (Fig. 1A). The distance between isolates belonging to the same ST was within the range of 0 to 367 allelic differences, which indicated that not all the isolates divided into the same STs according to the original MLST scheme were closely related (8, 9). Concurrently, using a threshold of 20 allelic differences (8) for a core genome, the strains were divided into 28 cgMLST clusters (Fig. 1), which were used as a default for designing a new MLST scheme.

For the new MLST scheme design, 1,174 core genes present in all 194 E. faecium genomes were analyzed as potential targets. From those, 38 highly discriminative candidate genes characterized by high sequence variability present in all the analyzed genomes were forwarded to a subsequent analysis. The gene combination was selected to divide isolates into groups corresponding as closely as possible to the cgMLST clusters. Then, the 8 most discriminative loci, one in each of 8 genes chosen in the previous step, were selected and once again the grouping against cgMLST clusters was checked. Finally, selected loci were used to design the new MLST scheme. The list of alleles for each locus was obtained from sequences available at the cgMLST.org Nomenclature server database. The individual loci and the numbers of determined alleles are shown in Table 1. Based on the new scheme, 194 E. faecium isolates were divided into 23 new STs, which more accurately copied the distribution defined based on cgMLST data (Fig. 1B).

**TABLE 1:** List of targets and primers used for new MLST scheme^*a*^

Gene ID (cgMLST.org)	Gene	Product	Primer ID	Sequence 5-3′	Amplicon length (bp)	No. of alleles
EFAU004_00397	*narB*	Tetronasin resistance transmembrane protein	narB FW	GCCGGTGTTCAAATCTCGAT	403	200
			narB RV	GAGTCGGACGTCAAGCAAAC		
EFAU004_01293	*dnaE*	DNA polymerase III subunit alpha	dnaE FW	CGCAAATAGCAAGTTTGTGAA	568	219
			dnaE RV	CGATAAGCGTTGGCTGAAAT		
EFAU004_01299	*pbp2b*	Penicillin binding protein transpeptidase domain protein	Pbp2b FW	CATCCGTTTAAAGATGGTAGCAA	466	197
			Pbp2b RV	CCCATGTTCCTTTTCGCTTA		
EFAU004_01691	*uvrA*	Excinuclease ABC subunit A	uvrA FW	TACGGCGTTCTTTTGGTACA	694	239
			uvrA RV	AAATGATTTTGGTGGCGTTC		
EFAU004_01915	*copA*	Copper-translocating P-type ATPase	copA FW	TTCTGGAGCTGCTTGATTGC	518	309
			copA RV	AGTCATTGCGTGTCCATGTG		
EFAU004_02011	*mdlA*	Multidrug resistance-like ATP-binding protein mdlA	mdlA FW	AGCAAAGGCAGAGGAATCAG	464	293
			mdlA RV	CGATTTTTAGAAAATTAGGCTGGT		
EFAU004_02027	HP	Hypothetical protein*	HP FW	CCGTTTGCTGCCTTGATATCA	582	282
			HP RV	TTCTGAGCCAGTTGATCCAAA		
EFAU004_01226	*rpoD*	RNA polymerase sigma factor RpoD	rpoD FW	ACGTTCCCGAGTAACACCAA	527	196
			rpoD RV	GGCATGCAGTTCCTTGACTT		

a According to EggNOG 5.0.0 database (42), EFAU004_02027 hypotetical protein corresponds to a putative adhesine protein (ID M7W_985).

The MLST scheme was further validated on a set of 1,843 genomes (194 isolates from the University Hospital Brno and 1,649 genomes from the NCBI Data sets database). A total of 223 STs were determined using the original MLST scheme, and 421 STs according to the new scheme. Furthermore, cgMLST was performed on all genomes, and this divided the genomes into 551 cgMLST clusters (threshold 20 allelic differences out of a 1,423 analyzed gene targets) and 100 isolates with no assigned cgMLST cluster. The UPGMA tree for 1,843 genomes showing the distribution of the new and original STs against whole genome MLST is shown in Fig. S1. Simpson’s Diversity index for individual schemes was 0.919 (CI 95% 0.911 to 0.927), 0.983 (CI 95% 0.981 to 0.984) and 0.991 (CI 95% 0.989 to 0.992) for the original scheme, the new MLST scheme and cgMLST, respectively. Adjusted Wallace values comparing the new and original and cgMLST typing schemes are shown in Table 2. The new MLST scheme has been uploaded to the PubMLST database (10) and run against the collection of 3,625 E. faecium genomes to validate the MLST scheme and expand the list of known alleles and STs. A total of 1,066 STs were determined as of March 2022. Recombination events for all 8 loci were determined using RDP5 software (11) based on alignments of all existing alleles. Our analysis revealed no evidence of recombination events in *copA*, HP2027, *mdlA*, *narB*, *pbp2*, and *uvrA* loci. However, we identified 1 unique recombination event in the *dnaE* locus and 4 unique events in the *rpoD* locus. Importantly, the occurrence of recombination events did not affect the typeability of any of the genomes used for the proposed MLST scheme validation (this included genomes from University Hospital Brno, NCBI data sets database and PubMLST genome collection).

**TABLE 2:** Adjusted Wallace values comparing new MLST scheme, original MLST scheme and cgMLST scheme (cgMLST.org)

	New MLST	Original MLST	cgMLST
New MLST	-	0.837 (CI 95% 0.811-0.962)	0.367 (CI 95% 0.351-0.382)
Original MLST	0.168 (CI 95% 0.155-.0.182)	-	0.071 (CI 95% 0.061-0.081)
cgMLST	0.686 (CI 95% 0.680-0.691)	0.658 (CI 95% 0.648-0.668)	-

One of the most significant outcomes of the proposed MLST was a new ST isolate distribution within the 3 most prevalent original STs: ST80 (*n* = 413, 22.41%), ST117 (*n* = 185, 10.04%), and ST17 (*n* = 170, 9.11%). Isolates grouped into the original ST80 differed in 0 to 549 core genomes alleles (*n* = 1423 core genes), with a median of 296 allele differences. Isolates from the original ST117 had 0 to 455 core genome allele differences, with a median of 256. Isolates from the original ST17 differed in 0 to 507 core genome alleles, with a median of 113 allele differences. Newly, isolates belonging to the original ST80 have been divided into 42 new STs, isolates belonging to the original ST117 into 14 new STs, and isolates belonging to the original ST17 into 35 new STs. A list of the new STs within the original ST80, ST17, and ST117, and a range of allele differences within new ST groups are listed in Table 3.

**TABLE 3:** Distribution of new STs within original ST80, ST17, and ST117 and number of core genome allelic differences within individual STs

Original ST	New ST	No. of isolates	No. of allelic differences (median)	Original ST	New ST	No. of isolates	No. of allelic differences (median)	Original ST	New ST	No. of isolates	No. of allelic differences (median)
ST80		413	0-549 (296)	ST17		170	0-507 (113)	ST117		185	0-455 (113)
	ST24	84	0-51 (1)		ST1	4	2-44 (41.5)		ST17	84	0-140 (51)
	ST25	2	2		ST2	19	0-283 (95)		ST18	36	0-207 (148.5)
	ST26	1	NA		ST3	1	NA		ST19	4	1-7 (3.5)
	ST39	1	NA		ST6	1	NA		ST42	34	0-106 (3)
	ST102	32	0-82 (12)		ST8	63	0-94 (38)		ST106	1	NA
	ST106	3	3-14 (11)		ST9	1	NA		ST123	10	0-8 (4)
	ST107	1	NA		ST10	1	NA		ST128	3	6-13 (11)
	ST111	3	0-2 (2)		ST43	3	8-24 (17)		ST258	2	55
	ST122	13	0-97 (56)		ST47	8	0-3 (0)		ST480	1	NA
	ST130	2	8		ST49	2	3		ST558	1	NA
	ST136	5	5-21 (15)		ST50	1	NA		ST581	1	NA
	ST137	1	NA		ST67	4	0-3 (0)		ST709	5	0-5 (3)
	ST138	13	0-183 (168)		ST100	1	NA		ST839	2	5
	ST139	4	0-15(7.5)		ST101	1	NA		ST1016	1	NA
	ST140	49	0-42 (6)		ST103	1	NA				
	ST141	1	NA		ST104	1	NA				
	ST142	1	NA		ST105	1	NA				
	ST143	10	0-4 (0)		ST115	8	2-93 (57.5)				
	ST144	8	0-275 (163.5)		ST117	11	0-20 (10)				
	ST148	3	0 (0)		ST146	1	NA				
	ST149	1	NA		ST160	1	NA				
	ST150	2	1		ST161	2	3				
	ST151	4	2-8 (6)		ST162	1	NA				
	ST152	88	0-153 (86)		ST163	2	0				
	ST153	2	42		ST167	1	NA				
	ST154	3	1-5 (4)		ST251	1	NA				
	ST170	16	1-16 (8)		ST261	2	20				
	ST173	18	0-64 (3)		ST276	6	1-27 (17)				
	ST174	2	0		ST544	1	NA				
	ST175	1	NA		ST602	5	10-19 (15)				
	ST252	16	0-34 (5)		ST629	1	NA	
	ST482	9	0-54 (0)		ST812	2	59				
	ST564	1	NA		ST831	2	4				
	ST658	2	42		ST942	9	0-7 (4)				
	ST746	1	NA		ST1006	1	NA				
	ST479	1	NA					
	ST838	1	NA					
	ST871	1	NA								
	ST1009	1	NA								
	ST1013	2	0								
	ST1026	1	NA								
	ST1030	3	1-4 (3)								

New clonal complex (CC) founders were determined based on the proposed MLST scheme using PhyloViz 2.0 software and the eBURST algorithm. CCs were assigned to STs using the PubMLST algorithm with the condition of at least 5 identical alleles, regarding the founder allelic profile. The CC distribution based on the original and proposed MLST is shown in Table 4. CC Simpson's Diversity index was 0.238 (CI 95% 0.215 to 0.261) for the original MLST scheme, and 0.808 (CI 95% 0.796 to 0.820) for the proposed MLST scheme. Direct comparison for individual isolates is shown in Fig. S1.

**TABLE 4:** Distribution of CC based on original and new MLST schemes

	Clonal complex	No. of isolates
Original MLST scheme	CC17	1589
	no CC assigned	254
New MLST scheme	CC2	663
	CC152	230
	CC128	179
	CC17	178
	CC36	73
	CC184	45
	CC73	39
	CC89	37
	CC60	35
	CC162	32
	CC62	27
	CC7	14
	no CC assigned	291

## DISCUSSION

Tracking bacterial strain relatedness using molecular typing is one of the fundamental components of modern epidemiology (2, 12). Accurate description of bacterial strains and populations is essential for early outbreak detection, ongoing infection control, monitoring bacterial populations dynamics, and developing and implementing preventive measures to forestall outbreak occurrence. Replacing traditionally used techniques, such as PFGE and MLST, by introducing new, advanced molecular methods, such as WGS, changed bacterial strain typing and revolutionized epidemiology. Extending the classic MLST schemes based on 7 to 8 housekeeping genes to core/whole genome MLSTs, or even detailed evaluation based on single-nucleotide variants (2) are excellent examples of diagnostics directly improved by WGS. As a result, the discriminatory power of molecular typing greatly increases along with the precision/accuracy of strain relatedness determination. However, the lack of optimization and inconsistency in data processing and evaluation (unified core genome schemes are not established, neither are pipelines for data processing) remains the main bottleneck, especially in reproducing results at the interlaboratory level. Consequently, traditional methods such as MLST must remain an integral part of epidemiological investigations, along with their limitations. Regarding MLST, it is advantageous to use the sequencing data obtained by WGS and perform this analysis *in silico*. *In silico* MLST based on WGS data provides inter-laboratory and even globally comparable data, while it does not require extra costs. This approach allows us to use highly accurate WGS typing for local epidemiological investigations and simultaneously compare the situation within other hospitals or regions, and to track strain clonality worldwide.

The original MLST schemes were designed based on the putative gene functions, or gene sequences in closely related bacterial species for the vast majority of clinically important bacteria (3, 13, 14). However, at this time, WGS provides the ability to target hyper-variable loci across the entire core genome to find the most appropriate loci combinations, which maximize the discriminatory power and bring the resulting STs clustering as close as possible to the WGS results and to the real strain distribution/divergence. E. faecium MLST (3) is a great example of clinical typing evolution. Several studies have been published demonstrating the current MLST scheme is not optimally designed (4–6). Among the main drawbacks, the authors mention the possibility of recombining MLST genes (15), the frequent absence of the *pstS* gene (16), and obvious discrepancies between MLST clustering and WGS data (4, 5). This, in summary, shows the limited accuracy of this scheme for its use in typing.

The MLST scheme we propose in this study is based on WGS data obtained from both local strains and online available genomes originating from various countries around the world. The advantage of our approach is in the order of the steps in the scheme design. First, we divided isolates into WGS clusters and then searched for variable loci based on this clustering, so the MLST scheme proposed here reflects the individual STs true genetic relatedness to the maximum extent possible. As the strains classified according to the original scheme to ST80, ST17, and ST117 belong to the most clinically significant strains (17–19), it is likely that dividing them into the new STs can significantly affect further epidemiology studies and epidemiological measures in relation to E. faecium. Certain E. faecium isolates lack the *pstS* gene and belong to a specific group, which is assigned the pstS 0 allele for the purpose of the MLST typing. These strains have been found in clinical environments worldwide (4, 20). With a new MLST scheme, all isolates lacking the *pstS* gene became completely typeable, providing a more accurate ST distribution, particularly for ST1421 (Fig. S2).

The new MLST scheme also groups isolates that differ in a greater number of alleles, although there are fewer such groups than in the original scheme, and the differences within these groups are significantly smaller. However, it is important to note that the discriminatory power of MLST schemes is lower than that of cgMLST, which compares hundreds to thousands of genes. The main disadvantage of cgMLST is the lack of interlaboratory standardization, leading to the utilization of various schemes across different laboratories. This encompasses both the existence of different published schemes for the same species (8, 21–23) and the development of schemes designed to specific populations and employed mostly at a local level. Despite the increasing accessibility of WGS, many clinical laboratories still lack the necessary resources to fully utilize its potential. Therefore, the highly standardized MLST method remains an ideal option to monitor epidemiology, as demonstrated by recent studies that have used only MLST or PFGE (24–27). Moreover, even studies using WGS data might still prefer to use standard MLST schemes to interpret results (28, 29).

In addition to STs, another output of MLST analysis is CCs, which are used to monitor the spread of clones carrying important determinants of antimicrobial resistance or virulence (30). These CCs are still frequently used by epidemiologists despite their relatively low discriminative power. Specifically in E. faecium, the globally widespread CC17 (the original MLST scheme) includes a substantial part of the most widespread STs and thus unites genetically distant strains into 1 complex (17, 31). As part of the proposed MLST scheme design, CCs were also determined on the pilot genome group. These, as in the case of STs, clustered isolates into more CCs than the original scheme and thus allowed more accurate clonal spread monitoring. Despite the stronger discriminative power of the new CC spread assessment, it is well known that origin and relatedness based on CCs alone can easily link genetically and epidemiologically unrelated isolates together and thus be misleading.

When introducing a new typing scheme, it is crucial to anticipate the potential confusion that may arise when comparing results with studies that utilize the old scheme. To mitigate this confusion, 1 practical solution is to use both typing schemes simultaneously and report the combined sequence type as a combination of the individual STs. This approach offers several advantages, such as enabling recent and past studies to be compared, possibly providing higher discrimination power. Furthermore, utilizing both typing schemes can be particularly beneficial when performing MLST analysis *in silico* from sequencing data as it does not require any extra costs, just a simple analysis of the same data. However, for studies using only MLST analysis *per se*, we suggest using only the new MLST scheme, which has a higher discriminatory power.

In this study, we proposed and validated a new MLST scheme for E. faecium, which is based on genome-wide data and thus reflects the tested strains’ more accurate genetic similarity. This newly proposed scheme combines discriminatory power provided by WGS, but also preserves MLST for routine clinical typing, which is still considered a golden standard. The proposed scheme offers a significantly higher discriminatory power than the original MLST scheme, while it preserves the possibility of worldwide interlaboratory comparisons. Currently, it is advisable to use modern methods based on WGS and subsequently perform *in silico* MLST on these data.

## MATERIALS AND METHODS

### Isolates and genomes.

A total of 194 clinical isolates of E. faecium were collected at the University Hospital Brno in the Czech Republic between June 2017 and July 2022. In addition, we downloaded 1,825 E. faecium genomes from the NCBI Data sets database (https://www.ncbi.nlm.nih.gov/) that met our selection criteria, which included only annotated genomes released after 2005 with genome completeness at the scaffold to complete level. Atypical genomes were excluded from our analysis. To ensure the accuracy of our data set, we used high-throughput average nucleotide identity (ANI) analysis (32) against a reference E. faecium genome (GCF_009734005.1) to exclude misidentified genomes. After this process, we excluded 22 genomes due to a low percentage (< 95%) of good targets for cgMLST, 25 genomes due to the presence of ambivalent bases in the original MLST sequence, and 20 genomes due to the presence of ambivalent bases in the proposed MLST sequence. In total, we used 1,843 genomes (194 from University Hospital Brno and 1,649 from NCBI Data sets database) to design and validate our new MLST scheme. For reference, we have included a complete list of genomes in a Supplementary file (Table S1), which also contains information, about the collection date, geographical location of origin, and isolation source, where available.

### DNA isolation.

Genomic DNA (gDNA) for mini-MLST and WGS was purified using GenElute Bacterial Genomic DNA Kit (Sigma-Aldrich) according to the manufacturer’s instructions.

### Mini-MLST.

Mini-MLST was performed on a Bio-Rad CFX96 platform (Bio-Rad) using primers described by Tong et al. (7). The reaction mixture contained 10 μL 2× SensiFAST HRM mix (Bioline Reagents), 0.5 μM each primer, 30 ng of gDNA, and PCR grade water to a final volume of 20 μL. Cycling parameters were: 95°C for 3 min, 40 cycles of 95°C for 5 s, 65°C for 10 s, and 72°C for 20 s, followed by 1 cycle of 95°C for 2 min and 50°C for 20 s, terminated by HRM ramping from 72 to 88°C, increasing by 0.1°C at each step.

### WGS.

Sequencing libraries were prepared using KAPA HyperPlus Kits (Roche). Sequencing was performed on the Illumina MiSeq platform using the MiSeq reagent kit v2 (500-cycles) (Illumina). After quality control, the reads were trimmed via Trimmomatic version 0.36 (33). Burrows-Wheeler Alignment MEM (v0.7.17-r1188) (34) was used for reference-based assembly. E. faecium seed genome NC_017022.1 was used as a reference. Samtools version 1.7 (35) was used to remove low-quality and duplicated reads, followed by generating consensus sequences.

### *In silico* MLST.

*In silico* MLST, cgMLST, and wgMLST analyses were performed for all clinical isolates and database genomes using Ridom SeqSphere+ version 8 software (Ridom, DE) (36).

### New MLST scheme design.

Firstly, 194 E. faecium genomes obtained by sequencing strains isolated at the University Hospital Brno, Czech Republic were used for the initial analysis. Genomes were analyzed via SeqSphere+ software (Ridom) using cgMLST.org Nomenclature server database, and WGS clusters and cgMLST profiles were determined. Next, genes present in all strains were found and used for the subsequent analysis. Among these, a combination of 8 most discriminating genes were found using MATLAB version 2020a (The MathWorks) (37). UPGMA trees were created for the 6 most discriminating gene combinations and compared against the WGS clusters. A final combination of 8 genes were selected based on the highest match to the WGS clusters. Finally, all loci were scanned for recombination events using Recombination Detection program v.5.34 (RDP5) (11).

Afterwards, the most variable regions, with a length of 400 to 800 bp, were determined within the selected genes. Primers targeting these regions were designed using the Primer3 online tool v4.1.0 (38) (Tab. 1). A UPGMA tree based on selected loci was constructed to check the preservation of discriminatory power. The PCR was optimized as follows: reaction mixture contained 2,5 μL of 10×PCR buffer II (Thermo Scientific), 1 μL of 25 mM MgCl_2_ (Thermo Scientific)_2_, 0,3 μL of *Taq* DNA polymerase 5U/μL (Thermo Scientific), 0,5 μL of 10 mM dNTPs (Thermo Scientific), 0,3 μL of 25 μM forward primer, 0,3 μL of 25 μM reverse primer, 150 ng of bacterial DNA, and PCR grade water up to final volume of 25 μL; thermocycling parameters were 94°C for 4 min, 35 cycles of 95°C for 30 s, 60°C for 1 min, and 72°C for 1 min, followed by 1 cycle of 72°C for 7 min.

### New MLST scheme validation.

Furthermore, 1,649 genomes downloaded from the NCBI data sets database (https://www.ncbi.nlm.nih.gov/datasets/) were used for MLST scheme validation. New clonal complexes were calculated using Phyloviz 2.0 software (39), and Simpson’s index of diversity (40) and Adjusted Wallace (41) were calculated using online tools available at http://www.comparingpartitions.info/. The new MLST scheme is available at https://pubmlst.org/organisms/enterococcus-faecium (10).

### Data availability.

The raw sequencing data were deposited in the SRA database under the BioProject accession number PRJNA675431. Accession numbers for all genomes used in MLST scheme design, including both those available from the NCBI database and newly sequenced ones, are listed in Table S1.

## References

[1] Fiore E, Van Tyne D, Gilmore MS. 2019. Pathogenicity of enterococci. Microbiol Spectr 7:GPP3-0053-2018. doi:10.1128/microbiolspec.GPP3-0053-2018. PMC6629438 31298205

[2] Simar SR, Hanson BM, Arias CA. 2021. Techniques in bacterial strain typing: past, present, and future. Curr Opin Infect Dis 34:339–345. doi:10.1097/QCO.0000000000000743. 34039880 PMC9245535

[3] Homan WL, Tribe D, Poznanski S, Li M, Hogg G, Spalburg E, Van Embden JD, Willems RJ. 2002. Multilocus sequence typing scheme for *Enterococcus faecium*. J Clin Microbiol 40:1963–1971. doi:10.1128/JCM.40.6.1963-1971.2002. 12037049 PMC130786

[4] Raven KE, Reuter S, Reynolds R, Brodrick HJ, Russell JE, Török ME, Parkhill J, Peacock SJ. 2016. A decade of genomic history for healthcare-associated *Enterococcus faecium* in the United Kingdom and Ireland. Genome Res 26:1388–1396. doi:10.1101/gr.204024.116. 27527616 PMC5052055

[5] Rios R, Reyes J, Carvajal LP, Rincon S, Panesso D, Echeverri AM, Dinh A, Kolokotronis SO, Narechania A, Tran TT, Munita JM, Murray BE, Planet PJ, Arias CA, Diaz L. 2020. Genomic epidemiology of vancomycin-resistant *Enterococcus faecium* (VREfm) in Latin America: revisiting the global VRE population structure. Sci Rep 10:5636. doi:10.1038/s41598-020-62371-7. 32221315 PMC7101424

[6] van Hal SJ, Ip CLC, Ansari MA, Wilson DJ, Espedido BA, Jensen SO, Bowden R. 2016. Evolutionary dynamics of *Enterococcus faecium* reveals complex genomic relationships between isolates with independent emergence of vancomycin resistance. Microb Genom 2:e000048. doi:10.1099/mgen.0.000048. 27713836 PMC5049587

[7] Tong SY, Xie S, Richardson LJ, Ballard SA, Dakh F, Grabsch EA, Grayson ML, Howden BP, Johnson PD, Giffard PM. 2011. High-resolution melting genotyping of *Enterococcus faecium* based on multilocus sequence typing derived single nucleotide polymorphisms. PLoS One 6:e29189. doi:10.1371/journal.pone.0029189. 22195020 PMC3241712

[8] de Been M, Pinholt M, Top J, Bletz S, Mellmann A, van Schaik W, Brouwer E, Rogers M, Kraat Y, Bonten M, Corander J, Westh H, Harmsen D, Willems RJ. 2015. Core genome multilocus sequence typing scheme for high- resolution typing of *Enterococcus faecium*. J Clin Microbiol 53:3788–3797. doi:10.1128/JCM.01946-15. 26400782 PMC4652124

[9] Higgs C, Sherry NL, Seemann T, Horan K, Walpola H, Kinsella P, Bond K, Williamson DA, Marshall C, Kwong JC, Grayson ML, Stinear TP, Gorrie CL, Howden BP. 2022. Optimising genomic approaches for identifying vancomycin-resistant *Enterococcus faecium* transmission in healthcare settings. Nat Commun 13:509. doi:10.1038/s41467-022-28156-4. 35082278 PMC8792028

[10] Jolley KA, Bray JE, Maiden MCJ. 2018. Open-access bacterial population genomics: BIGSdb software, the PubMLST.org website and their applications. Wellcome Open Res 3:124. doi:10.12688/wellcomeopenres.14826.1. 30345391 PMC6192448

[11] Martin DP, Varsani A, Roumagnac P, Botha G, Maslamoney S, Schwab T, Kelz Z, Kumar V, Murrell B. 2021. RDP5: a computer program for analyzing recombination in, and removing signals of recombination from, nucleotide sequence datasets. Virus Evol 7:veaa087. doi:10.1093/ve/veaa087. 33936774 PMC8062008

[12] Ranjbar R, Karami A, Farshad S, Giammanco GM, Mammina C. 2014. Typing methods used in the molecular epidemiology of microbial pathogens: a how-to guide. New Microbiol 37:1–15. 24531166

[13] Enright MC, Spratt BG. 1998. A multilocus sequence typing scheme for *Streptococcus pneumoniae*: identification of clones associated with serious invasive disease. Microbiology 144:3049–3060. doi:10.1099/00221287-144-11-3049. 9846740

[14] Suerbaum S, Lohrengel M, Sonnevend A, Ruberg F, Kist M. 2001. Allelic diversity and recombination in *Campylobacter jejuni*. J Bacteriol 183:2553–2559. doi:10.1128/JB.183.8.2553-2559.2001. 11274115 PMC95172

[15] Howden BP, Holt KE, Lam MM, Seemann T, Ballard S, Coombs GW, Tong SY, Grayson ML, Johnson PD, Stinear TP. 2013. Genomic insights to control the emergence of vancomycin-resistant enterococci. mBio 4:e00412-13. doi:10.1128/mBio.00412-13. 23943759 PMC3747580

[16] Carter GP, Buultjens AH, Ballard SA, Baines SL, Tomita T, Strachan J, Johnson PD, Ferguson JK, Seemann T, Stinear TP, Howden BP. 2016. Emergence of endemic MLST non-typeable vancomycin-resistant *Enterococcus faecium*. J Antimicrob Chemother 71:3367–3371. doi:10.1093/jac/dkw314. 27530751

[17] Fang H, Fröding I, Ullberg M, Giske CG. 2021. Genomic analysis revealed distinct transmission clusters of vancomycin-resistant *Enterococcus faecium* ST80 in Stockholm, Sweden. J Hosp Infect 107:12–15. doi:10.1016/j.jhin.2020.10.019. 33127458

[18] Dyrkell F, Giske CG, Fang H. 2021. Epidemiological typing of ST80 vancomycin-resistant *Enterococcus faecium*: core genome multilocus sequence typing versus single nucleotide polymorphism-based typing. J Glob Antimicrob Resist 25:119–123. doi:10.1016/j.jgar.2021.03.005. 33762207

[19] Weber A, Maechler F, Schwab F, Gastmeier P, Kola A. 2020. Increase of vancomycin-resistant *Enterococcus faecium* strain type ST117 CT71 at Charité - Universitätsmedizin Berlin, 2008 to 2018. Antimicrob Resist Infect Control 9:109. doi:10.1186/s13756-020-00754-1. 32678047 PMC7364619

[20] Lemonidis K, Salih TS, Dancer SJ, Hunter IS, Tucker NP. 2019. Emergence of an Australian-like pstS-null vancomycin resistant *Enterococcus faecium* clone in Scotland. PLoS One 14:e0218185. doi:10.1371/journal.pone.0218185. 31194809 PMC6563996

[21] Pinholt M, Gumpert H, Bayliss S, Nielsen JB, Vorobieva V, Pedersen M, Feil E, Worning P, Westh H. 2017. Genomic analysis of 495 vancomycin-resistant *Enterococcus faecium* reveals broad dissemination of a vanA plasmid in more than 19 clones from Copenhagen, Denmark. J Antimicrob Chemother 72:40–47. doi:10.1093/jac/dkw360. 27605596

[22] Werner A, Mölling P, Fagerström A, Dyrkell F, Arnellos D, Johansson K, Sundqvist M, Norén T. 2020. Whole genome sequencing of *Clostridioides difficile* PCR ribotype 046 suggests transmission between pigs and humans. PLoS One 15:e0244227. doi:10.1371/journal.pone.0244227. 33347506 PMC7751860

[23] Bortolaia V, Kaas RS, Ruppe E, Roberts MC, Schwarz S, Cattoir V, Philippon A, Allesoe RL, Rebelo AR, Florensa AF, Fagelhauer L, Chakraborty T, Neumann B, Werner G, Bender JK, Stingl K, Nguyen M, Coppens J, Xavier BB, Malhotra-Kumar S, Westh H, Pinholt M, Anjum MF, Duggett NA, Kempf I, Nykäsenoja S, Olkkola S, Wieczorek K, Amaro A, Clemente L, Mossong J, Losch S, Ragimbeau C, Lund O, Aarestrup FM. 2020. ResFinder 4.0 for predictions of phenotypes from genotypes. J Antimicrob Chemother 75:3491–3500. doi:10.1093/jac/dkaa345. 32780112 PMC7662176

[24] Wan TW, Yeo HH, Lee TF, Huang YT, Hsueh PR, Chiu HC. 2023. Molecular epidemiology of bacteraemic vancomycin-resistant *Enterococcus faecium* isolates and *in vitro* activities of SC5005 and other comparators against these isolates collected from a medical centre in northern Taiwan, 2019–2020. J Antimicrob Chemother 78:457–465. doi:10.1093/jac/dkac414. 36527680

[25] Yan MY, He YH, Ruan GJ, Xue F, Zheng B, Lv Y. 2023. The prevalence and molecular epidemiology of vancomycin-resistant *Enterococcus* (VRE) carriage in patients admitted to intensive care units in Beijing, China. J Microbiol Immunol Infect 56:351–357. doi:10.1016/j.jmii.2022.07.001. 35922268

[26] Cherak Z, Bendjama E, Moussi A, Benbouza A, Grainat N, Rolain JM, Loucif L. 2022. First detection of vanA positive *Enterococcus faecium* clonal complex 17 in hospital wastewater in Algeria: an epidemiological report. New Microbes New Infect 47:100977. doi:10.1016/j.nmni.2022.100977. 35586845 PMC9108983

[27] Wardal E, Żabicka D, Hryniewicz W, Sadowy E. 2022. VanA-*Enterococcus faecalis* in Poland: hospital population clonal structure and vanA mobilome. Eur J Clin Microbiol Infect Dis 41:1245–1261. doi:10.1007/s10096-022-04479-4. 36057762 PMC9489580

[28] Liu S, Li Y, He Z, Wang Y, Wang J, Jin D. 2022. A molecular study regarding the spread of vanA vancomycin-resistant *Enterococcus faecium* in a tertiary hospital in China. J Glob Antimicrob Resist 31:270–278. doi:10.1016/j.jgar.2022.10.010. 36273808

[29] Trautmannsberger I, Kolberg L, Meyer-Buehn M, Huebner J, Werner G, Weber R, Heselich V, Schroepf S, Muench HG, von Both U. 2022. Epidemiological and genetic characteristics of vancomycin-resistant *Enterococcus faecium* isolates in a University Children's Hospital in Germany: 2019 to 2020. Antimicrob Resist Infect Control 11:48. doi:10.1186/s13756-022-01081-3. 35279207 PMC8917738

[30] Feil EJ, Li BC, Aanensen DM, Hanage WP, Spratt BG. 2004. eBURST: inferring patterns of evolutionary descent among clusters of related bacterial genotypes from multilocus sequence typing data. J Bacteriol 186:1518–1530. doi:10.1128/JB.186.5.1518-1530.2004. 14973027 PMC344416

[31] Khan MA, Northwood JB, Loor RG, Tholen AT, Riera E, Falcón M, van Belkum A, van Westreenen M, Hays JP, Paraguayan Antimicrobial Network. 2010. High prevalence of ST-78 infection-associated vancomycin-resistant *Enterococcus faecium* from hospitals in Asunción, Paraguay. Clin Microbiol Infect 16:624–627. doi:10.1111/j.1469-0691.2009.02898.x. 19622078

[32] Jain C, Rodriguez-R LM, Phillippy AM, Konstantinidis KT, Aluru S. 2018. High throughput ANI analysis of 90K prokaryotic genomes reveals clear species boundaries. Nat Commun 9:5114. doi:10.1038/s41467-018-07641-9. 30504855 PMC6269478

[33] Bolger AM, Lohse M, Usadel B. 2014. Trimmomatic: a flexible trimmer for Illumina sequence data. Bioinformatics 30:2114–2120. doi:10.1093/bioinformatics/btu170. 24695404 PMC4103590

[34] Li H, Durbin R. 2009. Fast and accurate short read alignment with Burrows-Wheeler transform. Bioinformatics 25:1754–1760. doi:10.1093/bioinformatics/btp324. 19451168 PMC2705234

[35] Li H, Handsaker B, Wysoker A, Fennell T, Ruan J, Homer N, Marth G, Abecasis G, Durbin R, Subgroup GPDP, 1000 Genome Project Data Processing Subgroup. 2009. The sequence alignment/map format and SAMtools. Bioinformatics 25:2078–2079. doi:10.1093/bioinformatics/btp352. 19505943 PMC2723002

[36] Jünemann S, Sedlazeck FJ, Prior K, Albersmeier A, John U, Kalinowski J, Mellmann A, Goesmann A, von Haeseler A, Stoye J, Harmsen D. 2013. Updating benchtop sequencing performance comparison. Nat Biotechnol 31:294–296. doi:10.1038/nbt.2522. 23563421

[37] The Math Works I. 2020. MATLAB, v2020a. The Math Works, Inc., https://www.mathworks.com/. Accessed 11 June 2021.

[38] Kõressaar T, Lepamets M, Kaplinski L, Raime K, Andreson R, Remm M. 2018. Primer3_masker: integrating masking of template sequence with primer design software. Bioinformatics 34:1937–1938. doi:10.1093/bioinformatics/bty036. 29360956

[39] Nascimento M, Sousa A, Ramirez M, Francisco AP, Carriço JA, Vaz C. 2017. PHYLOViZ 2.0: providing scalable data integration and visualization for multiple phylogenetic inference methods. Bioinformatics 33:128–129. doi:10.1093/bioinformatics/btw582. 27605102

[40] Hunter PR, Gaston MA. 1988. Numerical index of the discriminatory ability of typing systems: an application of Simpson's index of diversity. J Clin Microbiol 26:2465–2466. doi:10.1128/jcm.26.11.2465-2466.1988. 3069867 PMC266921

[41] Severiano A, Pinto FR, Ramirez M, Carriço JA. 2011. Adjusted Wallace coefficient as a measure of congruence between typing methods. J Clin Microbiol 49:3997–4000. doi:10.1128/JCM.00624-11. 21918028 PMC3209087

[42] Huerta-Cepas J, Szklarczyk D, Heller D, Hernández-Plaza A, Forslund SK, Cook H, Mende DR, Letunic I, Rattei T, Jensen LJ, von Mering C, Bork P. 2019. eggNOG 5.0: a hierarchical, functionally and phylogenetically annotated orthology resource based on 5090 organisms and 2502 viruses. Nucleic Acids Res 47:D309–D314. doi:10.1093/nar/gky1085. 30418610 PMC6324079

