# Supplementary material for: New Multilocus Sequence Typing Scheme for Enterococcus faecium Based on Whole Genome Sequencing Data
Source: Microbiol Spectr. 2023 Jun 12;11(4):e05107-22. doi: 10.1128/spectrum.05107-22 (PMC10434285; doi:10.1128/spectrum.05107-22)
Supplement: Supplemental file 2 — Supplemental material. Download spectrum.05107-22-s0002.pdf, PDF file, 0.7 MB [file spectrum.05107-22-s0002.pdf]

Fig. S2 Whole genome MLST based UPGMA-Tree for isolates with missing *pstS* gene and comparison of original ST and original Clonal complexes, new ST, new Clonal Complexes, and cgMLST clusters.

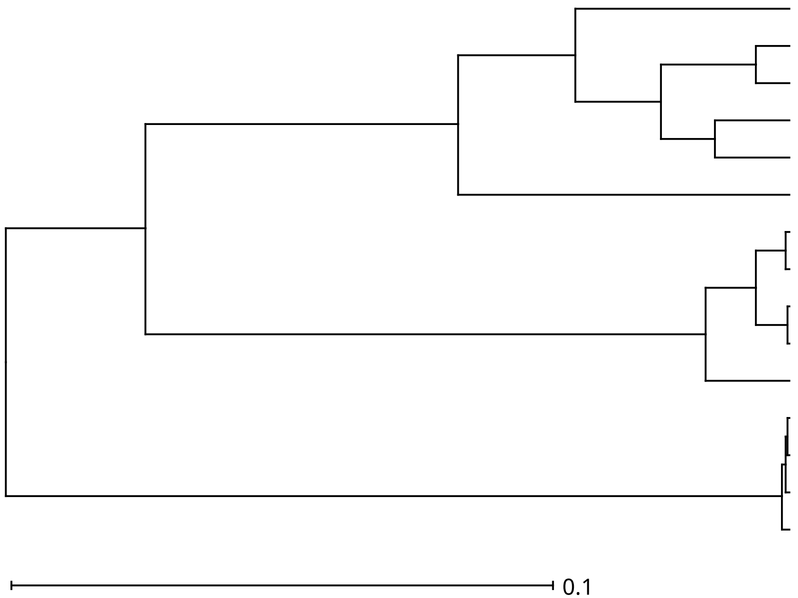

ID	original MLST ST	Original MLST clonal complex	New MLST ST	New MLST clonal complex	cgMLST cluster	Collection date	Isolation location	Isolation source
GCF-003020685.1	1421	CC17	14	CC2	1251	2015	Australia	Clinical isolate
GCF-002007625.1	1421	CC17	4	CC2	1471	2014	South Korea	Rectal swab
GCF-002025065.1	1421	CC17	4	CC2	1471	2014	Korea: republic of	Rectal swab
GCF-021172105.3	2096	CC17	4	CC2	6566	2021	South Korea: Daegu	n/a
GCF-002025045.1	1421	CC17	4	CC2	1747	2015	Korea: republic of	Rectal swab
GCF-002024245.1	1421	CC17	2	CC2	5037	2014	South Korea	Rectal swab
GCF-004300425.1	1478	CC17	17	CC17	4923	2018	USA:Little Rock	Blood
GCF-004300395.1	1478	CC17	17	CC17	4923	2018	USA:Little Rock	Blood
GCF-004152415.1	1478	CC17	17	CC17	3735	2018	USA: St. Louis	Hospital Surface
GCF-004151815.1	1478	CC17	17	CC17	3735	2018	USA: St. Louis	Hospital Surface
GCF-009697285.1	1478	CC17	17	CC17	3579	2017	Canada	Clinical isolate
GCF-003940995.1	2100	CC17	746	CC128	1954	2016	Pakistan	Light switch in hospital intensive care unit
GCF-003938065.1	2100	CC17	746	CC128	1954	2016	Pakistan	Nursing call button in hospital intensive care unit
GCF-003937575.1	2100	CC17	746	CC128	1954	2016	Pakistan	Rail in hospital intensive care unit
GCF-003937935.1	2100	CC17	746	CC128	1954	2016	Pakistan	Rail in hospital intensive care unit
