# Supplementary material for: New Multilocus Sequence Typing Scheme for Enterococcus faecium Based on Whole Genome Sequencing Data
Source: Microbiol Spectr. 2023 Jun 12;11(4):e05107-22. doi: 10.1128/spectrum.05107-22 (PMC10434285; doi:10.1128/spectrum.05107-22)
Supplement: Supplemental file 3 — Supplemental material. Download spectrum.05107-22-s0003.pdf, PDF file, 0.6 MB [file spectrum.05107-22-s0003.pdf]

**Tab. S1** List of genomes, their accession numbers/IDs, and available metadata

| ID | Isolation Source | Host | Host Disease | Collection Date | Collection location |
|---|---|---|---|---|---|
| GCF-001886635.1 | Blood | Homo sapiens | Bloodstream infection | n/a | n/a |
| GCF-014489995.1 | Clinical isolate | Homo sapiens | Bloodstream infection | n/a | n/a |
| GCF-014490015.1 | Clinical isolate | Homo sapiens | Bloodstream infection | n/a | n/a |
| GCF-020176925.1 | Blood | Homo sapiens | Bloodstream infection | n/a | n/a |
| GCF-020178155.1 | Blood | Homo sapiens | Bloodstream infection | n/a | n/a |
| GCF-009697285.1 | Clinical isolate | Homo sapiens | Bloodstream infection | n/a | n/a |
| GCF-000147235.1 | n/a | Homo sapiens | n/a | n/a | n/a |
| GCF-000147315.1 | n/a | Homo sapiens | n/a | n/a | n/a |
| GCF-000148025.1 | n/a | Homo sapiens | n/a | n/a | n/a |
| GCF-000148145.1 | n/a | Homo sapiens | n/a | n/a | n/a |
| GCF-000148285.1 | n/a | Homo sapiens | n/a | n/a | n/a |
| GCF-000148325.1 | n/a | Homo sapiens | n/a | n/a | n/a |
| GCF-000157435.1 | n/a | n/a | n/a | n/a | n/a |
| GCF-000157535.1 | n/a | n/a | n/a | n/a | n/a |
| GCF-000157555.1 | n/a | n/a | n/a | n/a | n/a |
| GCF-000157575.1 | n/a | n/a | n/a | n/a | n/a |
| GCF-000157595.1 | n/a | n/a | n/a | n/a | USA: Texas |
| GCF-000157615.1 | n/a | n/a | n/a | 1998 | Australia: Melbourne |
| GCF-000157635.1 | n/a | n/a | n/a | 2011 | China |
| GCF-000157655.1 | n/a | n/a | n/a | 2011 | China |
| GCF-000161855.1 | n/a | n/a | n/a | n/a | n/a |
| GCF-000258325.1 | n/a | n/a | n/a | n/a | n/a |
| GCF-000313155.1 | Culture | n/a | n/a | n/a | n/a |
| GCF-000313195.1 | Culture | n/a | n/a | n/a | n/a |
| GCF-028437655.1 | n/a | Vicugna vicigna f. pacos | n/a | n/a | n/a |
| GCF-028437725.1 | n/a | Vicugna vicigna f. pacos | n/a | n/a | n/a |
| GCF-000321785.1 | n/a | Canis familiaris | n/a | n/a | n/a |
| GCF-000321625.1 | Stool | Homo sapiens | n/a | n/a | n/a |
| GCF-000321645.1 | Stool | Homo sapiens | n/a | n/a | n/a |
| GCF-000321665.1 | Stool | Homo sapiens | n/a | n/a | n/a |
| GCF-000321685.1 | Blood | Homo sapiens | n/a | n/a | n/a |
| GCF-000321705.1 | Blood | Homo sapiens | n/a | n/a | n/a |
| GCF-000321725.1 | Clinical isolate | n/a | n/a | n/a | n/a |
| GCF-000321745.1 | Stool | Homo sapiens | n/a | n/a | n/a |
| GCF-000321765.1 | Rumen | Bison bison | n/a | n/a | n/a |
| GCF-000322425.1 | Stool | Canis familiaris | n/a | n/a | n/a |
| GCF-000321805.1 | n/a | Gallus gallus domesticus | n/a | n/a | n/a |
| GCF-000321825.1 | Caecum | Struthio camelus | n/a | n/a | n/a |
| GCF-004332275.1 | n/a | Carrot | n/a | n/a | n/a |
| GCF-000321865.1 | Stool | Homo sapiens | n/a | n/a | n/a |
| GCF-000321885.1 | Chesse | n/a | n/a | n/a | n/a |
| GCF-000321905.1 | Fish burger | n/a | n/a | n/a | n/a |
| GCF-000321925.1 | Blood | Homo sapiens | n/a | n/a | n/a |
| GCF-000321945.1 | n/a | Mus musculus | n/a | n/a | n/a |
| GCF-000321965.1 | Pus | Homo sapiens | n/a | n/a | n/a |
| GCF-000321985.1 | Stomach | Homo sapiens | n/a | n/a | n/a |
| GCF-000322005.1 | Gut | Homo sapiens | n/a | n/a | n/a |
| GCF-000322025.1 | River water | n/a | n/a | n/a | n/a |
| GCF-000322045.1 | River water | n/a | n/a | n/a | n/a |
| GCF-000322065.1 | Blood | Homo sapiens | n/a | n/a | n/a |
| GCF-000322085.1 | Stool | Homo sapiens | n/a | n/a | n/a |
| GCF-000322105.1 | Urine | Homo sapiens | n/a | n/a | n/a |
| GCF-000322125.1 | Blood | Homo sapiens | n/a | n/a | n/a |
| GCF-000322145.1 | Clinical isolate | n/a | n/a | n/a | n/a |
| GCF-000322165.1 | Poultry | n/a | n/a | n/a | n/a |
| GCF-000322185.1 | n/a | Gallus gallus domesticus | n/a | n/a | n/a |
| GCF-000322205.1 | Urine | Homo sapiens | n/a | n/a | n/a |
| GCF-000322225.1 | Blood | Homo sapiens | n/a | n/a | n/a |
| GCF-000322245.1 | Blood | Homo sapiens | n/a | n/a | n/a |
| GCF-000322265.1 | Blood | Homo sapiens | n/a | 2011 | China |
| GCF-000322285.1 | Blood | Homo sapiens | n/a | 2011 | China |
| GCF-000322305.1 | Blood | Homo sapiens | n/a | n/a | n/a |
| GCF-000322325.1 | n/a | Gallus gallus domesticus | n/a | 1992 | United Kingdom |
| GCF-000322345.1 | Drainage | Homo sapiens | n/a | 1995 | Netherlands |
| GCF-000322365.1 | Clinical isolate | n/a | n/a | 1996 | Netherlands |
| GCF-000322385.1 | Clinical isolate | n/a | n/a | 1996 | Netherlands |
| GCF-000322405.1 | Blood | Homo sapiens | n/a | 1997 | Israel |
| GCF-002140435.1 | Stool | Corvus corone | n/a | n/a | Belgium |
| GCF-000322445.1 | Clinical isolate | n/a | n/a | n/a | n/a |
| GCF-000322465.1 | Clinical isolate | n/a | n/a | n/a | Spain |
| GCF-000336405.1 | Milk | n/a | n/a | 1998 | Netherlands |
| GCF-000390465.1 | Blood | Homo sapiens | n/a | 1998 | Netherlands |
| GCF-000390485.1 | Blood | Homo sapiens | n/a | 2001 | USA |
| GCF-000391745.1 | n/a | Homo sapiens | n/a | n/a | France |
| GCF-004332055.1 | Animal-cattle-steer | Bos taurus | n/a | n/a | Spain |
| GCF-020593005.1 | n/a | Bos taurus | n/a | 2000 | United Kingdom |
| GCF-021172105.3 | n/a | Canis familiaris | n/a | 2002 | Netherlands |
| GCF-002140515.1 | Stool | Haliaeetus | n/a | 1994 | Belgium |
| GCF-002141135.1 | Stool | Haliaeetus | n/a | 1995 | Belgium |
| GCF-023182835.2 | n/a | Sus domesticus | n/a | 1995 | Belgium |
| GCF-002140315.1 | Stool | Oyster | n/a | 2001 | South Africa |
| GCF-000321565.1 | n/a | Sus domesticus | n/a | 2001 | Germany |
| GCF-000321585.1 | n/a | Sus domesticus | n/a | 2001 | Ireland |
| GCF-000321605.1 | n/a | Sus domesticus | n/a | 1956 | Norway |
| GCF-000321845.1 | Stool | Sus domesticus | n/a | 1964 | Norway |
| GCF-000391785.1 | n/a | Sus domesticus | n/a | 1957 | Netherlands |

| | | | | | |
|---|---|---|---|---|---|
| GCF-000391805.1 | n/a | Sus domesticus | n/a | 1959 | Netherlands |
| GCF-000391825.1 | n/a | Sus domesticus | n/a | 1960 | Netherlands |
| GCF-000391845.1 | n/a | Sus domesticus | n/a | 1965 | Netherlands |
| GCF-000391865.1 | n/a | Sus domesticus | n/a | 1979 | Netherlands |
| GCF-000391885.1 | n/a | Sus domesticus | n/a | 1981 | Netherlands |
| GCF-000391905.1 | n/a | Sus domesticus | n/a | 1982 | Netherlands |
| GCF-000391925.1 | n/a | Sus domesticus | n/a | n/a | Tanzania |
| GCF-000392165.1 | n/a | Homo sapiens | n/a | 2001 | Spain |
| GCF-000392195.1 | n/a | Homo sapiens | n/a | 2001 | Netherlands |
| GCF-000392215.1 | n/a | n/a | n/a | 2000 | Germany |
| GCF-000392255.1 | n/a | Homo sapiens | n/a | 2000 | Germany |
| GCF-000392315.1 | n/a | n/a | n/a | 2001 | Denmark |
| GCF-000393415.1 | n/a | n/a | n/a | 2004 | Netherlands |
| GCF-000393435.1 | n/a | n/a | n/a | 2001 | USA |
| GCF-000393675.1 | n/a | n/a | n/a | 2006 | Netherlands |
| GCF-000393695.1 | n/a | n/a | n/a | 2002 | Netherlands |
| GCF-000393735.1 | n/a | n/a | n/a | 2000 | Netherlands |
| GCF-000393755.1 | n/a | n/a | n/a | 2002 | Netherlands |
| GCF-000393775.1 | n/a | n/a | n/a | 2004 | Netherlands |
| GCF-000393855.1 | n/a | n/a | n/a | 2004 | Sweden |
| GCF-000394415.1 | n/a | n/a | n/a | 1999 | Italy |
| GCF-000394435.1 | n/a | n/a | n/a | 2002 | Germany |
| GCF-000394475.1 | n/a | n/a | n/a | 2005 | Hungary |
| GCF-000394495.1 | n/a | n/a | n/a | 2006 | Netherlands |
| GCF-000394535.1 | n/a | n/a | n/a | n/a | Denmark |
| GCF-000394555.1 | n/a | n/a | n/a | 2010 | Latvia |
| GCF-000394575.1 | n/a | n/a | n/a | 2010 | Portugal |
| GCF-000394595.1 | n/a | n/a | n/a | n/a | n/a |
| GCF-000394635.1 | n/a | n/a | n/a | n/a | Italy |
| GCF-000394655.1 | n/a | n/a | n/a | 2005 | Netherlands |
| GCF-000394675.1 | n/a | n/a | n/a | 1998 | Denmark |
| GCF-000394695.1 | n/a | n/a | n/a | 2000 | Denmark |
| GCF-000394715.1 | n/a | n/a | n/a | 2001 | Denmark |
| GCF-000394735.1 | n/a | n/a | n/a | 2001 | Denmark |
| GCF-000394755.1 | n/a | n/a | n/a | 2001 | Denmark |
| GCF-000391945.1 | n/a | Sus domesticus | n/a | 2001 | Denmark |
| GCF-000395445.1 | n/a | n/a | n/a | 2001 | Denmark |
| GCF-000395465.1 | n/a | n/a | n/a | 2002 | Denmark |
| GCF-000395485.1 | n/a | n/a | n/a | 2003 | Denmark |
| GCF-000395505.1 | n/a | n/a | n/a | 1997 | Denmark |
| GCF-000395525.1 | n/a | n/a | n/a | 1997 | Denmark |
| GCF-000395545.1 | n/a | n/a | n/a | 1997 | Denmark |
| GCF-000395565.1 | n/a | n/a | n/a | 1997 | Denmark |
| GCF-000395585.1 | n/a | n/a | n/a | 1998 | Denmark |
| GCF-000395605.1 | n/a | n/a | n/a | 1998 | Denmark |
| GCF-000395625.1 | n/a | n/a | n/a | 1998 | Denmark |
| GCF-000395645.1 | n/a | n/a | n/a | 1999 | Denmark |
| GCF-000395665.1 | n/a | n/a | n/a | 1999 | Denmark |
| GCF-000395685.1 | n/a | n/a | n/a | 1995 | Denmark |
| GCF-000395705.1 | n/a | Gallus gallus domesticus | n/a | 1995 | Denmark |
| GCF-000395725.1 | n/a | Gallus gallus domesticus | n/a | 1995 | Denmark |
| GCF-000395745.1 | n/a | Gallus gallus domesticus | n/a | 1995 | Denmark |
| GCF-000395765.1 | n/a | Gallus gallus domesticus | n/a | 1994 | n/a |
| GCF-000395785.1 | n/a | Gallus gallus domesticus | n/a | n/a | Netherlands |
| GCF-000395805.1 | n/a | Gallus gallus domesticus | n/a | 1994 | n/a |
| GCF-000395825.1 | n/a | Homo sapiens | n/a | n/a | n/a |
| GCF-000395845.1 | n/a | Homo sapiens | n/a | n/a | n/a |
| GCF-000395865.1 | n/a | Homo sapiens | n/a | 1996 | France: Hopital Toulouse |
| GCF-000395885.1 | n/a | Homo sapiens | n/a | 1996 | France: Hopital Toulouse |
| GCF-000395905.1 | n/a | n/a | n/a | 1998 | France: Institut Gustav-Roussy |
| GCF-000395925.1 | n/a | n/a | n/a | 2000 | France: Hopital Pellegrin-Bordeaux |
| GCF-000395945.1 | n/a | n/a | n/a | 1992 | n/a |
| GCF-000395965.1 | n/a | Gallus gallus domesticus | n/a | n/a | n/a |
| GCF-000391965.1 | n/a | Sus domesticus | n/a | 1986 | n/a |
| GCF-000391985.1 | n/a | Sus domesticus | n/a | 1986 | n/a |
| GCF-000392005.1 | n/a | Sus domesticus | n/a | 1989 | France: Caen |
| GCF-000392025.1 | n/a | Sus domesticus | n/a | 1989 | France: Hopital E. Herriot |
| GCF-000392045.1 | n/a | Sus domesticus | n/a | 1994 | France: Nimes |
| GCF-000392065.1 | n/a | Sus domesticus | n/a | 1994 | Switzerland: Geneve |
| GCF-000396805.1 | n/a | n/a | n/a | 1996 | USA: New York |
| GCF-000396825.1 | n/a | n/a | n/a | 1996 | USA: New York |
| GCF-000396845.1 | n/a | n/a | n/a | n/a | n/a |
| GCF-000396885.1 | n/a | n/a | n/a | n/a | n/a |
| GCF-000396925.1 | n/a | n/a | n/a | n/a | n/a |
| GCF-000396945.1 | n/a | n/a | n/a | n/a | n/a |
| GCF-000396965.1 | n/a | n/a | n/a | n/a | n/a |
| GCF-000397005.1 | n/a | n/a | n/a | n/a | n/a |
| GCF-000397025.1 | n/a | n/a | n/a | n/a | n/a |
| GCF-000397045.1 | n/a | n/a | n/a | 2002 | Denmark |
| GCF-000407065.1 | n/a | n/a | n/a | 1994 | France: Aix en Provence |
| GCF-000407085.1 | n/a | n/a | n/a | 1994 | France: Aix en Provence |
| GCF-000407105.1 | n/a | n/a | n/a | 1994 | France: Aix en Provence |
| GCF-000407325.1 | n/a | n/a | n/a | 1994 | France: Aix en Provence |
| GCF-000407345.1 | n/a | n/a | n/a | 1994 | France: Hopital Saint-Louis |
| GCF-000411015.1 | n/a | Homo sapiens | n/a | 1993 | France: Hopital Saint-Louis |
| GCF-000411035.1 | n/a | Homo sapiens | n/a | 1994 | France: Hopital Saint-Louis |
| GCF-000411655.2 | Fermented sausage | n/a | n/a | 1994 | France: Hopital Saint-Louis |
| GCF-000415265.2 | n/a | n/a | n/a | 1993 | France: Hopital Saint-Louis |
| GCF-000415285.2 | n/a | n/a | n/a | 1993 | France: Hopital Saint-Louis |

| | | | | | |
|---|---|---|---|---|---|
| GCF-000415305.1 | n/a | n/a | n/a | 1994 | France: Hopital Saint-Louis |
| GCF-000415345.1 | n/a | n/a | n/a | 1996 | France: Centre Hospitalier Lyon-Sud |
| GCF-000415365.1 | n/a | n/a | n/a | 1996 | USA: New York |
| GCF-000444405.1 | n/a | n/a | n/a | 2010 | Denmark |
| GCF-000479125.1 | Blood | n/a | n/a | 2010 | Denmark |
| GCF-000479145.1 | Clinical isolate | n/a | n/a | 2010 | Denmark |
| GCF-000479165.1 | Blood | n/a | n/a | 2010 | Denmark |
| GCF-000737555.1 | n/a | n/a | n/a | 2010 | Denmark |
| GCF-000392085.1 | n/a | Sus domesticus | n/a | 2010 | Denmark |
| GCF-000392105.1 | n/a | Sus domesticus | n/a | 2010 | Denmark |
| GCF-000787055.1 | Probiotic preparation | n/a | n/a | 2010 | Denmark |
| GCF-000392125.1 | n/a | Sus domesticus | n/a | 2010 | Denmark |
| GCF-000392145.1 | n/a | Sus domesticus | n/a | 2010 | Denmark |
| GCF-000395425.1 | n/a | Sus domesticus | n/a | 1996 | Switzerland: Geneve |
| GCF-000934365.1 | Urine | Homo sapiens | n/a | 1996 | Switzerland: Geneve |
| GCF-000951815.1 | Bile | Homo sapiens | n/a | 1996 | Switzerland: Geneve |
| GCF-001052395.1 | Clinical isolate | Homo sapiens | n/a | 1997 | Denmark |
| GCF-001052405.1 | Clinical isolate | Homo sapiens | n/a | 2008 | USA: Michigan |
| GCF-001052435.1 | Clinical isolate | Homo sapiens | n/a | 2008 | USA: Michigan |
| GCF-001052485.1 | Clinical isolate | Homo sapiens | n/a | 2008 | USA: Michigan |
| GCF-001052685.1 | Clinical isolate | Homo sapiens | n/a | 2008 | USA: Michigan |
| GCF-001053135.1 | Clinical isolate | Homo sapiens | n/a | 2008 | USA: Michigan |
| GCF-001053835.1 | Clinical isolate | Homo sapiens | n/a | 2008 | USA: Michigan |
| GCF-001054055.1 | Clinical isolate | Homo sapiens | n/a | 1994 | n/a |
| GCF-001054105.1 | Clinical isolate | Homo sapiens | n/a | 1994 | n/a |
| GCF-001054215.1 | Clinical isolate | Homo sapiens | n/a | 1996 | USA: New York |
| GCF-001055235.1 | Clinical isolate | Homo sapiens | n/a | 1996 | USA: New York |
| GCF-001055445.1 | Clinical isolate | Homo sapiens | n/a | 1996 | USA: New York |
| GCF-001055845.1 | Clinical isolate | Homo sapiens | n/a | 1996 | USA: New York |
| GCF-001056605.1 | Clinical isolate | Homo sapiens | n/a | 1996 | n/a |
| GCF-001056745.1 | Clinical isolate | Homo sapiens | n/a | 1996 | n/a |
| GCF-001056925.1 | Clinical isolate | Homo sapiens | n/a | 1996 | Switzerland: Geneve |
| GCF-001058945.1 | Clinical isolate | Homo sapiens | n/a | 1996 | France: Hopital Toulouse |
| GCF-001059685.1 | Clinical isolate | Homo sapiens | n/a | n/a | n/a |
| GCF-001059755.1 | Clinical isolate | Homo sapiens | n/a | n/a | n/a |
| GCF-001059965.1 | Clinical isolate | Homo sapiens | n/a | n/a | n/a |
| GCF-001059995.1 | Clinical isolate | Homo sapiens | n/a | n/a | n/a |
| GCF-001060065.1 | Clinical isolate | Homo sapiens | n/a | n/a | n/a |
| GCF-001076335.1 | Clinical isolate | Homo sapiens | n/a | n/a | China |
| GCF-001298485.1 | n/a | Homo sapiens | n/a | n/a | China |
| GCF-001412695.1 | Laboratory strain | n/a | n/a | 2011 | Italy |
| GCF-001543555.1 | Laboratory strain | n/a | n/a | n/a | n/a |
| GCF-001543565.1 | Laboratory strain | n/a | n/a | n/a | n/a |
| GCF-001543575.1 | Laboratory strain | n/a | n/a | n/a | n/a |
| GCF-001543585.1 | Laboratory strain | n/a | n/a | n/a | n/a |
| GCF-001543635.1 | Laboratory strain | n/a | n/a | n/a | n/a |
| GCF-001543645.1 | Laboratory strain | n/a | n/a | n/a | n/a |
| GCF-001543665.1 | Laboratory strain | n/a | n/a | 2004 | Australia: Adelaide |
| GCF-001543675.1 | Laboratory strain | n/a | n/a | 2009 | France: Nimes |
| GCF-001543715.1 | Laboratory strain | n/a | n/a | 1996 | Brazil |
| GCF-001543735.1 | Laboratory strain | n/a | n/a | n/a | n/a |
| GCF-001543745.1 | Laboratory strain | n/a | n/a | 2011 | Denmark |
| GCF-001543765.1 | Laboratory strain | n/a | n/a | 2011 | Denmark |
| GCF-000772525.1 | Clinical isolate | Homo sapiens | Bacteremia | 1992 | USA: Brooklyn |
| GCF-001543795.1 | Laboratory strain | n/a | n/a | 2010 | Russia: Saint-Petersburg |
| GCF-001543825.1 | n/a | n/a | n/a | 2011 | Denmark |
| GCF-001543835.1 | Laboratory strain | n/a | n/a | 2011 | Denmark |
| GCF-001546375.1 | Vagina | Homo sapiens | n/a | 2011 | Denmark |
| GCF-001582095.1 | n/a | Homo sapiens | n/a | 2013 | China: Beijing |
| GCF-001582105.1 | Clinical isolate | Homo sapiens | n/a | 2013 | China: Beijing |
| GCF-001587115.1 | n/a | Homo sapiens | n/a | n/a | USA:Washington |
| GCF-001594345.1 | Stool | Homo sapiens | n/a | n/a | USA:Washington |
| GCF-000396685.1 | n/a | Sus domesticus | n/a | n/a | USA:Washington |
| GCF-001635875.1 | Blood | n/a | n/a | n/a | USA:Washington |
| GCF-001696275.1 | Stool | Homo sapiens | n/a | n/a | USA:Washington |
| GCF-001696285.1 | Stool | Homo sapiens | n/a | n/a | USA:Washington |
| GCF-001750885.1 | n/a | Homo sapiens | n/a | n/a | USA:Washington |
| GCF-001990565.1 | n/a | Homo sapiens | n/a | n/a | USA:Washington |
| GCF-001990575.1 | n/a | Homo sapiens | n/a | n/a | USA:Washington |
| GCF-001990605.1 | n/a | Homo sapiens | n/a | n/a | USA:Washington |
| GCF-001990615.1 | n/a | Homo sapiens | n/a | n/a | USA:Washington |
| GCF-001996345.1 | Bile | Homo sapiens | n/a | n/a | USA:Washington |
| GCF-002006745.1 | Handmade Mexican Cotija cheese | n/a | n/a | n/a | USA:Washington |
| GCF-002140215.1 | Stool | Bird | n/a | n/a | USA:Washington |
| GCF-002140255.1 | Stool | Reptile | n/a | n/a | USA:Washington |
| GCF-002140295.1 | n/a | Mollusc | n/a | n/a | USA:Washington |
| GCF-000396705.1 | n/a | Sus domesticus | n/a | n/a | USA:Washington |
| GCF-002140375.1 | Stool | Chelonia mydas | n/a | n/a | USA:Washington |
| GCF-002140385.1 | Stool | Meleagris gallopavo | n/a | n/a | USA:Washington |
| GCF-002140415.1 | Stool | Reptile | n/a | n/a | USA:Washington |
| GCF-000396725.1 | n/a | Sus domesticus | n/a | n/a | USA:Washington |
| GCF-002140455.1 | Stool | Gallus gallus domesticus | n/a | n/a | USA:Washington |
| GCF-002140475.1 | Stool | Bird | n/a | n/a | USA:Washington |
| GCF-000396745.1 | n/a | Sus domesticus | n/a | 1992 | Germany |
| GCF-002140535.1 | Stool | n/a | n/a | 2012 | Germany: Wernigerode |
| GCF-001518735.1 | Stool | Homo sapiens | Infection | 2014 | USA |
| GCF-002140555.1 | Stool | Buteo buteo | n/a | 2014 | Sweden: Gaevle |
| GCF-002140595.1 | Stool | Bird | n/a | 2013 | USA: Rhode Island |
| GCF-002140735.1 | Stool | n/a | n/a | 2013 | USA: Rhode Island |

| | | | | | |
|---|---|---|---|---|---|
| GCF-002140805.1 | Stool | Marmota flaviventris | n/a | 2013 | USA: Rhode Island |
| GCF-002140865.1 | Stool | Marmota flaviventris | n/a | 2013 | USA: Rhode Island |
| GCF-002140895.1 | Stool | Iguana iguana | n/a | 2013 | USA: Rhode Island |
| GCF-002140955.1 | Stool | Bird | n/a | 2013 | USA: Rhode Island |
| GCF-002141005.1 | Stool | Spheniscus magellanicus | n/a | 2013 | USA: Rhode Island |
| GCF-002141035.1 | Stool | Spheniscus magellanicus | n/a | 2013 | USA: Rhode Island |
| GCF-002141075.1 | Stool | Bird | n/a | 2013 | USA: Rhode Island |
| GCF-002141115.1 | Stool | Chelonia mydas | n/a | 2013 | USA: Rhode Island |
| GCF-002141125.1 | n/a | n/a | n/a | 2013 | USA: Rhode Island |
| GCF-000396765.1 | n/a | Sus domesticus | n/a | 2013 | USA: Rhode Island |
| GCF-002141175.1 | Stool | Passer domesticus | n/a | 2013 | USA: Rhode Island |
| GCF-002141195.1 | Stool | Bird | n/a | n/a | n/a |
| GCF-002141255.1 | Stool | Bird | n/a | 2013 | USA: Rhode Island |
| GCF-002141335.1 | Stool | n/a | n/a | n/a | n/a |
| GCF-002141355.1 | Stool | n/a | n/a | 1984 | France |
| GCF-002141435.1 | Stool | Spheniscus magellanicus | n/a | 1997 | USA: Ohio |
| GCF-002158275.1 | Blood | Homo sapiens | n/a | 2010 | Germany |
| GCF-001592725.1 | rectal swab | Homo sapiens | Endocarditis | 2009 | Brazil: Belo Horizonte |
| GCF-002158285.1 | Blood | Homo sapiens | n/a | n/a | USA: Connecticut |
| GCF-002158295.1 | Blood | Homo sapiens | n/a | n/a | Spain |
| GCF-002158325.1 | Blood | Homo sapiens | n/a | 2009 | USA: New York |
| GCF-002158355.1 | Blood | Homo sapiens | n/a | 2011 | France |
| GCF-002158365.1 | Blood | Homo sapiens | n/a | 1988 | France |
| GCF-002158375.1 | Blood | Homo sapiens | n/a | 2008 | France: Marseille |
| GCF-001720945.1 | Blood | Homo sapiens | Bacteremia | 2011 | USA:New York City |
| GCF-001720965.1 | Blood | Homo sapiens | Bacteremia | 2011 | USA:New York City |
| GCF-001720985.1 | Blood | Homo sapiens | Bacteremia | 2011 | USA:New York City |
| GCF-001721005.1 | Blood | Homo sapiens | Bacteremia | 2011 | USA:New York City |
| GCF-001721025.1 | Blood | Homo sapiens | Bacteremia | 2011 | USA:New York City |
| GCF-001721065.1 | Blood | Homo sapiens | Bacteremia | 2011 | USA:New York City |
| GCF-001721085.1 | Blood | Homo sapiens | Bacteremia | 2011 | USA:New York City |
| GCF-001721105.1 | Blood | Homo sapiens | Bacteremia | 2011 | USA:New York City |
| GCF-001721905.1 | Blood | Homo sapiens | Bacteremia | 2011 | USA: New York |
| GCF-002158385.1 | Blood | Homo sapiens | n/a | 2000 | Netherlands: Utrecht |
| GCF-002158435.1 | Blood | Homo sapiens | n/a | 2010 | Spain: Madrid |
| GCF-001895905.1 | Blood | Homo sapiens | Bacteremia | 2013 | USA |
| GCF-001953235.1 | Blood | Homo sapiens | Bacteremia | 2011 | USA: New York |
| GCF-001953255.1 | Blood | Homo sapiens | Bacteremia | 2011 | USA: New York |
| GCF-002158445.1 | Blood | Homo sapiens | n/a | 2015 | Chile |
| GCF-002174355.1 | Stool | Equus caballus | n/a | 2015 | Chile |
| GCF-002174435.1 | Stool | Bird | n/a | 2015 | Chile |
| GCF-002174445.1 | Stool | Bird | n/a | 2015 | Chile |
| GCF-002216245.1 | Stool | Bird | n/a | 2013 | Italy: Sassari |
| GCF-002265255.1 | n/a | Equus caballus | n/a | 2008 | Mexico:Jalisco |
| GCF-002007625.1 | Rectal swab | Homo sapiens | Non-insulin-dependent diabets mellitus | 2014 | South Korea |
| GCF-002024245.1 | Rectal swab | Homo sapiens | Chronic kidney disease, stage5 | 2014 | South Korea |
| GCF-002025045.1 | Rectal swab | Homo sapiens | Malignant neoplasm of rectosigmoid junction | 2015 | Korea: republic of |
| GCF-002025065.1 | Rectal swab | Homo sapiens | Malignant neoplasm of brain stem | 2014 | Korea: republic of |
| GCF-002442275.1 | Rectal swab | Homo sapiens | n/a | 2015 | Germany |
| GCF-002442315.1 | Rectal swab | Homo sapiens | n/a | n/a | Brazil |
| GCF-002442445.1 | Stool | Homo sapiens | n/a | 2013 | USA: Massachusetts |
| GCF-002442475.1 | Rectal swab | Homo sapiens | n/a | n/a | USA: MA, Cape Cod |
| GCF-002442955.1 | Stool | Homo sapiens | n/a | n/a | Brazil |
| GCF-002591965.2 | Korean fermented soybean paste | n/a | n/a | n/a | USA: South Carolina |
| GCF-002803675.1 | Stool | n/a | n/a | n/a | Brazil |
| GCF-002848385.1 | Fooder | n/a | n/a | n/a | Germany |
| GCF-002850515.1 | n/a | Homo sapiens | n/a | n/a | Germany: Brandenberg |
| GCF-002880635.1 | n/a | n/a | n/a | 2015 | Germany |
| GCF-002894545.1 | Clinical isolate | Homo sapiens | n/a | n/a | Germany: Berlin |
| GCF-002894565.1 | Clinical isolate | Homo sapiens | n/a | n/a | n/a |
| GCF-003202445.1 | Clinical isolate | Homo sapiens | n/a | n/a | Germany |
| GCF-003269465.1 | n/a | n/a | n/a | n/a | Germany: Berlin |
| GCF-003319975.1 | Breast meat | Gallus gallus domesticus | n/a | n/a | USA: Montana |
| GCF-003319985.1 | Breast meat | Gallus gallus domesticus | n/a | 2015 | Afghanistan |
| GCF-003320055.1 | Breast meat | Gallus gallus domesticus | n/a | 2015 | Afghanistan |
| GCF-003320065.1 | Breast meat | Gallus gallus domesticus | n/a | n/a | Netherlands: Utrecht |
| GCF-003320075.1 | Breast meat | Gallus gallus domesticus | n/a | 2015 | Germany |
| GCF-003320115.1 | Breast meat | Gallus gallus domesticus | n/a | n/a | Brazil |
| GCF-003320135.1 | Breast meat | Gallus gallus domesticus | n/a | n/a | Brazil |
| GCF-003320155.1 | Breast meat | Gallus gallus domesticus | n/a | 2015 | Germany |
| GCF-003320165.1 | Breast meat | Gallus gallus domesticus | n/a | n/a | Brazil |
| GCF-003320195.1 | Breast meat | Gallus gallus domesticus | n/a | 2014 | USA: Massachusetts |
| GCF-003320215.1 | Breast meat | Gallus gallus domesticus | n/a | n/a | Germany: Berlin |
| GCF-003320235.1 | Breast meat | Gallus gallus domesticus | n/a | n/a | Germany: Berlin |
| GCF-003320245.1 | Breast meat | Gallus gallus domesticus | n/a | 2015 | Germany |
| GCF-003320255.1 | Breast meat | Gallus gallus domesticus | n/a | 2015 | Germany |
| GCF-003320275.1 | Breast meat | Gallus gallus domesticus | n/a | n/a | USA: Montana |
| GCF-003320315.1 | Breast meat | Gallus gallus domesticus | n/a | n/a | USA: Montana |
| GCF-003320325.1 | Breast meat | Gallus gallus domesticus | n/a | n/a | Brazil |
| GCF-003320355.1 | Breast meat | Gallus gallus domesticus | n/a | 2012 | USA: Utah |
| GCF-003320365.1 | Breast meat | Gallus gallus domesticus | n/a | 2013 | USA: Utah |
| GCF-003320395.1 | Breast meat | Gallus gallus domesticus | n/a | 2013 | USA: Utah |
| GCF-003320405.1 | Breast meat | Gallus gallus domesticus | n/a | 2013 | USA: Utah |
| GCF-003320415.1 | Breast meat | Gallus gallus domesticus | n/a | 2007 | USA: Utah |
| GCF-003320455.1 | Breast meat | Gallus gallus domesticus | n/a | 2014 | USA: Utah |
| GCF-003320475.1 | Breast meat | Gallus gallus domesticus | n/a | 2014 | USA: Utah |
| GCF-003320485.1 | Breast meat | Gallus gallus domesticus | n/a | 2014 | USA: Utah |
| GCF-003320495.1 | Breast meat | Gallus gallus domesticus | n/a | 2014 | USA: Utah |
| GCF-003320505.1 | Breast meat | Gallus gallus domesticus | n/a | 2014 | USA: Utah |

| | | | | | |
|---|---|---|---|---|---|
| GCF-003320555.1 | Breast meat | Gallus gallus domesticus | n/a | n/a | USA: Montana |
| GCF-003320575.1 | Breast meat | Gallus gallus domesticus | n/a | 2015 | Germany |
| GCF-003320585.1 | Breast meat | Gallus gallus domesticus | n/a | 2015 | Germany |
| GCF-003320595.1 | Breast meat | Gallus gallus domesticus | n/a | 2015 | Germany |
| GCF-003320635.1 | Breast meat | Gallus gallus domesticus | n/a | 2014 | Russia: Orenburg |
| GCF-002334625.1 | Blood | Homo sapiens | Bacteremia | 2008 | Norway |
| GCF-003320655.1 | Breast meat | Gallus gallus domesticus | n/a | 2012 | Netherlands |
| GCF-003320665.1 | Breast meat | Gallus gallus domesticus | n/a | 2012 | Germany |
| GCF-003320695.1 | Breast meat | Gallus gallus domesticus | n/a | 2012 | Netherlands |
| GCF-003320715.1 | Breast meat | Gallus gallus domesticus | n/a | 2012 | Netherlands |
| GCF-003320735.1 | Breast meat | Gallus gallus domesticus | n/a | 2002 | Netherlands: Utrecht |
| GCF-003320755.1 | Breast meat | Gallus gallus domesticus | n/a | 2012 | Russia: St.Petersburg |
| GCF-003320775.1 | Breast meat | Gallus gallus domesticus | n/a | 2016 | Russia: St.Petersburg |
| GCF-003320795.1 | Breast meat | Gallus gallus domesticus | n/a | 2013 | Russia: St.Petersburg |
| GCF-003320805.1 | Breast meat | Gallus gallus domesticus | n/a | 2010 | Russia: St.Petersburg |
| GCF-003320815.1 | Breast meat | Gallus gallus domesticus | n/a | 2013 | Russia: St.Petersburg |
| GCF-003320855.1 | Breast meat | Gallus gallus domesticus | n/a | 2015 | South Korea |
| GCF-003350625.1 | Blood | Homo sapiens | n/a | 2015 | Russia: St.Petersburg |
| GCF-003350635.1 | Blood | Homo sapiens | n/a | 2012 | Russia: St.Petersburg |
| GCF-003350665.1 | Blood | Homo sapiens | n/a | 2012 | Russia: St.Petersburg |
| GCF-003350675.1 | Blood | Homo sapiens | n/a | 2014 | Russia: St.Petersburg |
| GCF-003350705.1 | Blood | Homo sapiens | n/a | 2013 | USA: New York |
| GCF-003350715.1 | Blood | Homo sapiens | n/a | 2013 | USA: New York |
| GCF-002761555.1 | Stool | Homo sapiens | Fecal carriage | 2016 | France: Montbrison |
| GCF-002777275.1 | Wound | Homo sapiens | Localized adiposity | 2013 | USA: New York |
| GCF-003350725.1 | Blood | Homo sapiens | n/a | 2016 | USA |
| GCF-003350765.1 | Blood | Homo sapiens | n/a | 2017 | China: Chendu, Sichuan |
| GCF-003350785.1 | Blood | Homo sapiens | n/a | 2016 | China |
| GCF-002848625.1 | Blood | Homo sapiens | Bacteremia | 2015 | USA: New York |
| GCF-002848645.1 | Blood | Homo sapiens | Bacteremia | 2015 | USA: New York |
| GCF-002848665.1 | Blood | Homo sapiens | Bacteremia | 2015 | USA: New York |
| GCF-002848685.1 | Blood | Homo sapiens | Bacteremia | 2015 | USA: New York |
| GCF-002848705.1 | Blood | Homo sapiens | Bacteremia | 2015 | USA: New York |
| GCF-002848725.1 | Blood | Homo sapiens | Bacteremia | 2015 | USA: New York |
| GCF-002848745.1 | Blood | Homo sapiens | Bacteremia | 2015 | USA: New York |
| GCF-003350805.1 | Blood | Homo sapiens | n/a | 2015 | China: Jiangxi |
| GCF-003350815.1 | Blood | Homo sapiens | n/a | 2009 | USA |
| GCF-003350845.1 | Stool | Homo sapiens | n/a | 2009 | Saudi Arabia: Riyadh |
| GCF-003350865.1 | Stool | Homo sapiens | n/a | 2009 | Saudi Arabia: Riyadh |
| GCF-002909305.1 | Catheter | Homo sapiens | Catheter infection | 2010 | France: Clichy |
| GCF-002973675.1 | Urine | Homo sapiens | Urinary tract infection | 2016 | France: Chambery |
| GCF-002973685.1 | Rectal swab | Homo sapiens | Rectal carriage | 2014 | France: Tours |
| GCF-002973715.1 | Rectal swab | Homo sapiens | Rectal carriage | 2015 | France: Narbonne |
| GCF-002973755.2 | Rectal swab | Homo sapiens | Rectal carriage | 2015 | France: Corbeille-Essonnes |
| GCF-002973795.1 | Rectal swab | Homo sapiens | Rectal carriage | 2013 | France: Creteil |
| GCF-002983785.1 | Blood | Homo sapiens | Bacteremia | 2014 | USA:Washington |
| GCF-002997315.1 | Stool | Homo sapiens | Rectal carriage | 2013 | France: Caen |
| GCF-002997345.1 | Rectal swab | Homo sapiens | Rectal carriage | 2013 | France: Creteil |
| GCF-003350885.1 | Stool | Homo sapiens | n/a | 2015 | Australia |
| GCF-003350905.1 | Stool | Homo sapiens | n/a | 2015 | Australia |
| GCF-003574925.1 | Chinese sausages | n/a | n/a | 2015 | Australia |
| GCF-003667965.1 | Biomedical source | n/a | n/a | 2015 | Australia |
| GCF-003711605.1 | Fermentedfish | n/a | n/a | 2015 | Australia |
| GCF-003071425.1 | Urine | Homo sapiens | Bacteriuria | 2014 | Denmark |
| GCF-003071445.1 | Urine | Homo sapiens | Bacteriuria | 2014 | Denmark |
| GCF-003719355.1 | Clinical isolate | Homo sapiens | n/a | 2009 | Saudi Arabia: Riyadh |
| GCF-003719365.1 | Clinical isolate | Homo sapiens | n/a | 1994 | France |
| GCF-003933345.2 | Rectal swab | Homo sapiens | n/a | n/a | USA: Louisville |
| GCF-003933355.1 | Rectal swab | Homo sapiens | n/a | n/a | USA: Phoenix |
| GCF-003933425.1 | Rectal swab | Homo sapiens | n/a | n/a | USA: Little Rock |
| GCF-003933435.1 | Rectal swab | Homo sapiens | n/a | n/a | USA: Louisville |
| GCF-003936585.1 | Alcohol foam dispenser in hospital intensive care unit | n/a | n/a | n/a | USA: Boise |
| GCF-003936605.1 | Sink in hospital intensive care unit | n/a | n/a | n/a | USA: Little Rock |
| GCF-003936615.1 | Nursing call button in hospital intensive care unit | n/a | n/a | n/a | USA: Baltimore |
| GCF-003937285.1 | Light switch in hospital intensive care unit | n/a | n/a | n/a | USA: Dallas |
| GCF-003937355.1 | Rail in hospital intensive care unit | n/a | n/a | n/a | USA: St. Paul |
| GCF-003937555.1 | Nursing call button in hospital intensive care unit | n/a | n/a | n/a | USA: Nashville |
| GCF-003937575.1 | Rail in hospital intensive care unit | n/a | n/a | n/a | USA: Phoenix |
| GCF-003937635.1 | Light switch in hospital intensive care unit | n/a | n/a | n/a | USA: Louisville |
| GCF-003937935.1 | Rail in hospital intensive care unit | n/a | n/a | n/a | USA: Chelsea |
| GCF-003938005.1 | Alcohol foam dispenser in hospital intensive care unit | n/a | n/a | n/a | USA: Boise |
| GCF-003938025.1 | Nursing call button in hospital intensive care unit | n/a | n/a | n/a | USA: Little Rock |
| GCF-003938035.1 | Rail in hospital intensive care unit | n/a | n/a | n/a | USA: Boston |
| GCF-003938045.1 | Alcohol foam dispenser in hospital intensive care unit | n/a | n/a | n/a | USA: Pennsylvania |
| GCF-003938065.1 | Nursing call button in hospital intensive care unit | n/a | n/a | n/a | USA: San Francisco |
| GCF-003938105.1 | Nursing call button in hospital intensive care unit | n/a | n/a | n/a | USA: New Orleans |
| GCF-003938125.1 | Rail in hospital intensive care unit | n/a | n/a | n/a | USA: Kansas City |
| GCF-003938135.1 | Light switch in hospital intensive care unit | n/a | n/a | n/a | USA: Kansas City |
| GCF-003938265.1 | Sink in hospital intensive care unit | n/a | n/a | n/a | USA: New York |
| GCF-003938275.1 | Rail in hospital intensive care unit | n/a | n/a | n/a | USA: Tuscaloosa |
| GCF-003938285.1 | Light switch in hospital intensive care unit | n/a | n/a | n/a | n/a |
| GCF-003938305.1 | Sink in hospital intensive care unit | n/a | n/a | n/a | USA: Denver |
| GCF-003938345.1 | Alcohol foam dispenser in hospital intensive care unit | n/a | n/a | n/a | USA: New York |
| GCF-003938365.1 | Nursing call button in hospital intensive care unit | n/a | n/a | n/a | USA: Denver |
| GCF-003938375.1 | Sink in hospital intensive care unit | n/a | n/a | n/a | USA: Detroit |
| GCF-003938395.1 | Alcohol foam dispenser in hospital intensive care unit | n/a | n/a | n/a | USA: Dallas |
| GCF-003938445.1 | Rail in hospital intensive care unit | n/a | n/a | n/a | USA: Trenton |
| GCF-003938485.1 | Rail in hospital intensive care unit | n/a | n/a | n/a | USA: Dallas |
| GCF-003938495.1 | Light switch in hospital intensive care unit | n/a | n/a | n/a | n/a |

| | | | | | |
|---|---|---|---|---|---|
| GCF-003938505.1 | Nursing call button in hospital intensive care unit | n/a | n/a | n/a | USA: Charlotte |
| GCF-003938515.1 | Sink in hospital intensive care unit | n/a | n/a | n/a | USA: Chicago |
| GCF-003938525.1 | Alcohol foam dispenser in hospital intensive care unit | n/a | n/a | n/a | USA: Baltimore |
| GCF-003939145.1 | Light switch in hospital intensive care unit | n/a | n/a | n/a | USA: Tuscaloosa |
| GCF-003939165.1 | Rail in hospital intensive care unit | n/a | n/a | n/a | USA: Fairfax |
| GCF-003939175.1 | Alcohol foam dispenser in hospital intensive care unit | n/a | n/a | n/a | USA: Pennsylvania |
| GCF-003939205.1 | Rail in hospital intensive care unit | n/a | n/a | n/a | USA: Tampa |
| GCF-003939215.1 | Nursing call button in hospital intensive care unit | n/a | n/a | n/a | USA: Fairfax |
| GCF-003939705.1 | Alcohol foam dispenser in hospital intensive care unit | n/a | n/a | n/a | USA: Tampa |
| GCF-003939745.1 | Alcohol foam dispenser in hospital intensive care unit | n/a | n/a | n/a | USA: Cincinnati |
| GCF-003939765.1 | Nursing call button in hospital intensive care unit | n/a | n/a | n/a | USA: Detroit |
| GCF-003939775.1 | Light switch in hospital intensive care unit | n/a | n/a | 2011 | USA: Utah |
| GCF-003939805.1 | Rail in hospital intensive care unit | n/a | n/a | 2009 | USA: Utah |
| GCF-003940185.1 | Alcohol foam dispenser in hospital intensive care unit | n/a | n/a | 2012 | USA: Utah |
| GCF-003940205.1 | Alcohol foam dispenser in hospital intensive care unit | n/a | n/a | 2006 | USA: Utah |
| GCF-003940215.1 | Rail in hospital intensive care unit | n/a | n/a | 2007 | USA: Utah |
| GCF-003940245.1 | Nursing call button in hospital intensive care unit | n/a | n/a | 2013 | USA: Utah |
| GCF-003940995.1 | Light switch in hospital intensive care unit | n/a | n/a | 2008 | USA: Utah |
| GCF-003965125.1 | Sink in hospital intensive care unit | n/a | n/a | 2012 | USA: Utah |
| GCF-004006255.1 | n/a | n/a | n/a | 2012 | USA: Utah |
| GCF-004015145.1 | Stool | n/a | n/a | 2012 | USA: Utah |
| GCF-004099785.1 | n/a | Bos taurus | n/a | 2008 | USA: Utah |
| GCF-004099795.1 | n/a | Bos taurus | n/a | 2014 | USA: Utah |
| GCF-004099895.1 | n/a | Bos taurus | n/a | 2013 | USA: Utah |
| GCF-004101385.1 | Food sample | n/a | n/a | 2012 | USA: Utah |
| GCF-004103475.1 | Food sample | n/a | n/a | 2014 | USA: Utah |
| GCF-004151765.1 | Hospital Surface | n/a | n/a | 2017 | China:changsha |
| GCF-004151775.1 | Hospital Surface | n/a | n/a | 2018 | China:Guangzhou |
| GCF-004151785.1 | Hospital Surface | n/a | n/a | 2016 | China: Liping, Guizhou province |
| GCF-004151795.1 | Hospital Surface | n/a | n/a | 2015 | China:Sichuan |
| GCF-004151815.1 | Hospital Surface | n/a | n/a | 2015 | China:Sichuan |
| GCF-004151865.1 | Hospital Surface | n/a | n/a | 2017 | South Africa: Ozwatini |
| GCF-004151875.1 | Hospital Surface | n/a | n/a | 2017 | South Africa: Ozwatini |
| GCF-004151885.1 | Hospital Surface | n/a | n/a | 2017 | South Africa: Ozwatini |
| GCF-004151925.1 | Hospital Surface | n/a | n/a | 2017 | South Africa: Ozwatini |
| GCF-004152415.1 | Hospital Surface | n/a | n/a | 2016 | Pakistan |
| GCF-004299865.1 | Blood | Homo sapiens | n/a | 2016 | Pakistan |
| GCF-004299875.1 | Blood | Homo sapiens | n/a | 2016 | Pakistan |
| GCF-004299885.1 | Blood | Homo sapiens | n/a | 2016 | Pakistan |
| GCF-004299905.1 | Blood | Homo sapiens | n/a | 2016 | Pakistan |
| GCF-004299925.1 | Blood | Homo sapiens | n/a | 2016 | Pakistan |
| GCF-004299965.1 | Blood | Homo sapiens | n/a | 2016 | Pakistan |
| GCF-004299975.1 | Blood | Homo sapiens | n/a | 2016 | Pakistan |
| GCF-004299995.1 | Blood | Homo sapiens | n/a | 2016 | Pakistan |
| GCF-004300005.1 | Blood | Homo sapiens | n/a | 2016 | Pakistan |
| GCF-004300025.1 | Blood | Homo sapiens | n/a | 2016 | Pakistan |
| GCF-004300065.1 | Blood | Homo sapiens | n/a | 2016 | Pakistan |
| GCF-004300075.1 | Blood | Homo sapiens | n/a | 2016 | Pakistan |
| GCF-004300105.1 | Blood | Homo sapiens | n/a | 2016 | Pakistan |
| GCF-004300115.1 | Blood | Homo sapiens | n/a | 2016 | Pakistan |
| GCF-004300125.1 | Blood | Homo sapiens | n/a | 2016 | Pakistan |
| GCF-004300165.1 | Blood | Homo sapiens | n/a | 2016 | Pakistan |
| GCF-004300175.1 | Blood | Homo sapiens | n/a | 2016 | Pakistan |
| GCF-004300185.1 | Blood | Homo sapiens | n/a | 2016 | Pakistan |
| GCF-004300195.1 | Blood | Homo sapiens | n/a | 2016 | Pakistan |
| GCF-004300245.1 | Blood | Homo sapiens | n/a | 2016 | Pakistan |
| GCF-004300265.1 | Blood | Homo sapiens | n/a | 2016 | Pakistan |
| GCF-004300275.1 | Blood | Homo sapiens | n/a | 2016 | Pakistan |
| GCF-004300285.1 | Blood | Homo sapiens | n/a | 2016 | Pakistan |
| GCF-004300315.1 | Blood | Homo sapiens | n/a | 2016 | Pakistan |
| GCF-004300345.1 | Blood | Homo sapiens | n/a | 2016 | Pakistan |
| GCF-004300355.1 | Blood | Homo sapiens | n/a | 2016 | Pakistan |
| GCF-004300375.1 | Blood | Homo sapiens | n/a | 2016 | Pakistan |
| GCF-004300395.1 | Blood | Homo sapiens | n/a | 2016 | Pakistan |
| GCF-004300425.1 | Blood | Homo sapiens | n/a | 2016 | Pakistan |
| GCF-004300435.1 | Blood | Homo sapiens | n/a | 2016 | Pakistan |
| GCF-004300445.1 | Blood | Homo sapiens | n/a | 2016 | Pakistan |
| GCF-004300465.1 | Blood | Homo sapiens | n/a | 2016 | Pakistan |
| GCF-004300505.1 | Blood | Homo sapiens | n/a | 2016 | Pakistan |
| GCF-004300525.1 | Blood | Homo sapiens | n/a | 2016 | Pakistan |
| GCF-004300535.1 | Blood | Homo sapiens | n/a | 2016 | Pakistan |
| GCF-004300545.1 | Blood | Homo sapiens | n/a | 2016 | Pakistan |
| GCF-004300555.1 | Blood | Homo sapiens | n/a | 2016 | Pakistan |
| GCF-004300605.1 | Blood | Homo sapiens | n/a | 2016 | Pakistan |
| GCF-004300635.1 | Blood | Homo sapiens | n/a | 2016 | Pakistan |
| GCF-004300645.1 | Blood | Homo sapiens | n/a | 2016 | Pakistan |
| GCF-004300655.1 | Blood | Homo sapiens | n/a | 2016 | Pakistan |
| GCF-004300705.1 | Blood | Homo sapiens | n/a | 2016 | Pakistan |
| GCF-004300715.1 | Blood | Homo sapiens | n/a | 2016 | Pakistan |
| GCF-004300725.1 | Blood | Homo sapiens | n/a | 2016 | Pakistan |
| GCF-004300755.1 | Blood | Homo sapiens | n/a | 2016 | Pakistan |
| GCF-003957785.1 | Urine | Homo sapiens | Urinary tract infection | 2016 | Australia: Brisbane |
| GCF-004300775.1 | Blood | Homo sapiens | n/a | 2016 | Pakistan |
| GCF-004300805.1 | Blood | Homo sapiens | n/a | n/a | n/a |
| GCF-000396785.1 | n/a | Sus domesticus | n/a | 2016 | South Korea: Seoul |
| GCF-000767345.1 | n/a | Sus domesticus | n/a | 2013 | USA |
| GCF-004328245.1 | n/a | Bos taurus | n/a | 2013 | USA |
| GCF-000767365.1 | n/a | Sus domesticus | n/a | 2013 | USA |
| GCF-000804385.1 | n/a | Sus domesticus | n/a | 2018 | South Korea |

| | | | | | |
|---|---|---|---|---|---|
| GCF-000804405.1 | n/a | Sus domesticus | n/a | 2018 | South Korea |
| GCF-004151665.1 | Clinical isolate | Homo sapiens | Infection | 2014 | Pakistan: Islamabad |
| GCF-004151675.1 | Clinical isolate | Homo sapiens | Infection | 2014 | Pakistan: Islamabad |
| GCF-004151685.1 | Clinical isolate | Homo sapiens | Infection | 2014 | Pakistan: Islamabad |
| GCF-000804415.1 | n/a | Sus domesticus | n/a | 2018 | USA: St. Louis |
| GCF-001622975.1 | Pig | Sus domesticus | n/a | 2018 | USA: St. Louis |
| GCF-005166365.1 | Infant stool | Homo sapiens | n/a | 2018 | USA: St. Louis |
| GCF-005234235.1 | Rectal swab | Homo sapiens | n/a | 2018 | USA: St. Louis |
| GCF-005234245.1 | Rectal swab | Homo sapiens | n/a | 2018 | USA: St. Louis |
| GCF-005234295.1 | Rectal swab | Homo sapiens | n/a | 2018 | USA: St. Louis |
| GCF-005234405.1 | Rectal swab | Homo sapiens | n/a | 2018 | USA: St. Louis |
| GCF-005234435.1 | Rectal swab | Homo sapiens | n/a | 2018 | USA: St. Louis |
| GCF-004151895.1 | Clinical isolate | Homo sapiens | Infection | 2018 | USA: St. Louis |
| GCF-005234505.1 | Rectal swab | Homo sapiens | n/a | 2018 | USA: St. Louis |
| GCF-004151965.1 | Clinical isolate | Homo sapiens | Infection | 2018 | USA: St. Louis |
| GCF-004151975.1 | Clinical isolate | Homo sapiens | Infection | 2018 | USA: St. Louis |
| GCF-004151995.1 | Clinical isolate | Homo sapiens | Infection | 2018 | USA: St. Louis |
| GCF-004152005.1 | Clinical isolate | Homo sapiens | Infection | 2018 | USA: St. Louis |
| GCF-004152065.1 | Clinical isolate | Homo sapiens | Infection | 2018 | USA: St. Louis |
| GCF-004152075.1 | Clinical isolate | Homo sapiens | Infection | 2018 | USA: St. Louis |
| GCF-004152085.1 | Clinical isolate | Homo sapiens | Infection | 2018 | USA: St. Louis |
| GCF-004152105.1 | Clinical isolate | Homo sapiens | Infection | 2018 | USA: St. Louis |
| GCF-004152135.1 | Clinical isolate | Homo sapiens | Infection | 2018 | USA: St. Louis |
| GCF-004152165.1 | Clinical isolate | Homo sapiens | Infection | 2018 | USA: St. Louis |
| GCF-004152175.1 | Clinical isolate | Homo sapiens | Infection | 2018 | USA: St. Louis |
| GCF-004152185.1 | Clinical isolate | Homo sapiens | Infection | 2018 | USA: St. Louis |
| GCF-004152205.1 | Clinical isolate | Homo sapiens | Infection | 2018 | USA: St. Louis |
| GCF-004152265.1 | Clinical isolate | Homo sapiens | Infection | 2018 | USA: St. Louis |
| GCF-004152275.1 | Clinical isolate | Homo sapiens | Infection | 2018 | USA: St. Louis |
| GCF-004152285.1 | Clinical isolate | Homo sapiens | Infection | 2018 | USA: St. Louis |
| GCF-004152305.1 | Clinical isolate | Homo sapiens | Infection | 2018 | USA: St. Louis |
| GCF-004152325.1 | Clinical isolate | Homo sapiens | Infection | 2014 | Pakistan: Islamabad |
| GCF-004152365.1 | Clinical isolate | Homo sapiens | Infection | 2014 | Pakistan: Islamabad |
| GCF-004152375.1 | Clinical isolate | Homo sapiens | Infection | 2014 | Pakistan: Islamabad |
| GCF-004152405.1 | Clinical isolate | Homo sapiens | Infection | 2018 | USA: St. Louis |
| GCF-005234565.1 | Abdominal wound | Homo sapiens | n/a | 2018 | USA: St. Louis |
| GCF-004152465.1 | Clinical isolate | Homo sapiens | Infection | 2018 | USA: St. Louis |
| GCF-004152475.1 | Clinical isolate | Homo sapiens | Infection | 2018 | USA: St. Louis |
| GCF-004152485.1 | Clinical isolate | Homo sapiens | Infection | 2018 | USA: St. Louis |
| GCF-004152505.1 | Clinical isolate | Homo sapiens | Infection | 2018 | USA: St. Louis |
| GCF-004152515.1 | Clinical isolate | Homo sapiens | Infection | 2018 | USA: St. Louis |
| GCF-004152565.1 | Clinical isolate | Homo sapiens | Infection | 2018 | USA: St. Louis |
| GCF-004152575.1 | Clinical isolate | Homo sapiens | Infection | 2018 | USA: St. Louis |
| GCF-004152585.1 | Clinical isolate | Homo sapiens | Infection | 2018 | USA: St. Louis |
| GCF-004152605.1 | Clinical isolate | Homo sapiens | Infection | 2018 | USA: St. Louis |
| GCF-005234745.1 | Poultry feces | Gallus gallus domesticus | n/a | 2018 | USA:Little Rock |
| GCF-005234975.1 | Stool | Homo sapiens | n/a | 2018 | USA:Little Rock |
| GCF-005235015.1 | Rectal swab | Homo sapiens | n/a | 2018 | USA:Little Rock |
| GCF-005235045.1 | Rectal swab | Homo sapiens | n/a | 2018 | USA:Little Rock |
| GCF-005235075.1 | Rectal swab | Homo sapiens | n/a | 2018 | USA:Little Rock |
| GCF-005235285.1 | Rectal swab | Homo sapiens | n/a | 2018 | USA:Little Rock |
| GCF-005235345.1 | Clinical isolate | Homo sapiens | n/a | 2018 | USA:Little Rock |
| GCF-005235395.1 | Rectal swab | Homo sapiens | n/a | 2018 | USA:Little Rock |
| GCF-005235475.1 | Rectal swab | Homo sapiens | n/a | 2018 | USA:Little Rock |
| GCF-005235575.1 | Clinical isolate | Homo sapiens | n/a | 2018 | USA:Little Rock |
| GCF-005235635.1 | Rectal swab | Homo sapiens | n/a | 2018 | USA:Little Rock |
| GCF-005235645.1 | Rectal swab | Homo sapiens | n/a | 2018 | USA:Little Rock |
| GCF-005235665.1 | Rectal swab | Homo sapiens | n/a | 2018 | USA:Little Rock |
| GCF-005235685.1 | Tissue | Homo sapiens | n/a | 2018 | USA:Little Rock |
| GCF-005235735.1 | Catheter | Homo sapiens | n/a | 2018 | USA:Little Rock |
| GCF-005235745.1 | Urine | Homo sapiens | n/a | 2018 | USA:Little Rock |
| GCF-005236155.1 | Poultry feces | Gallus gallus domesticus | n/a | 2018 | USA:Little Rock |
| GCF-005237055.1 | Poultry feces | Gallus gallus domesticus | n/a | 2018 | USA:Little Rock |
| GCF-005237065.1 | Poultry feces | Gallus gallus domesticus | n/a | 2018 | USA:Little Rock |
| GCF-005237545.1 | Poultry feces | Gallus gallus domesticus | n/a | 2018 | USA:Little Rock |
| GCF-005238445.1 | Poultry feces | Gallus gallus domesticus | n/a | 2018 | USA:Little Rock |
| GCF-005451945.1 | Stool | Homo sapiens | n/a | 2018 | USA:Little Rock |
| GCF-005844995.1 | Stool | Homo sapiens | n/a | 2018 | USA:Little Rock |
| GCF-005886545.1 | n/a | Homo sapiens | n/a | 2018 | USA:Little Rock |
| GCF-005886655.1 | n/a | Homo sapiens | n/a | 2018 | USA:Little Rock |
| GCF-005886715.1 | n/a | Homo sapiens | n/a | 2018 | USA:Little Rock |
| GCF-005886735.1 | n/a | Homo sapiens | n/a | 2018 | USA:Little Rock |
| GCF-005952885.1 | n/a | Homo sapiens | n/a | 2018 | USA:Little Rock |
| GCF-006280355.1 | Chicken | Gallus gallus domesticus | n/a | 2018 | USA:Little Rock |
| GCF-006337045.1 | Stool | Homo sapiens | n/a | 2018 | USA:Little Rock |
| GCF-006337145.1 | Stool | Homo sapiens | n/a | 2018 | USA:Little Rock |
| GCF-006351785.1 | Stool | Homo sapiens | n/a | 2018 | USA:Little Rock |
| GCF-006351845.1 | Chicken | n/a | n/a | 2018 | USA:Little Rock |
| GCF-006741355.1 | n/a | n/a | n/a | 2018 | USA:Little Rock |
| GCF-007625025.1 | Intestines | Homo sapiens | n/a | 2018 | USA:Little Rock |
| GCF-007923905.2 | Chicken | n/a | n/a | 2018 | USA:Little Rock |
| GCF-007923925.2 | Chicken | n/a | n/a | 2018 | USA:Little Rock |
| GCF-008000855.1 | n/a | Homo sapiens | n/a | 2018 | USA:Little Rock |
| GCF-008330605.1 | Meju, fermented soybean | n/a | n/a | 2018 | USA:Little Rock |
| GCF-009036045.1 | n/a | Oreochromis niloticus | n/a | 2018 | USA:Little Rock |
| GCF-009659825.1 | Soil | n/a | n/a | 2018 | USA:Little Rock |
| GCF-009659845.1 | Collard green | n/a | n/a | 2018 | USA:Little Rock |
| GCF-009733995.1 | Clinical isolate | Laboratory | n/a | 2018 | USA:Little Rock |
| GCF-009735345.1 | n/a | n/a | n/a | 2018 | USA:Little Rock |

| | | | | | |
|---|---|---|---|---|---|
| GCF-009735525.1 | Kefir grains | n/a | n/a | 2018 | USA:Little Rock |
| GCF-009832445.1 | Stool | Homo sapiens | n/a | 2018 | USA:Little Rock |
| GCF-009832485.1 | Stool | Homo sapiens | n/a | 2018 | USA:Little Rock |
| GCF-004302885.1 | Gastrointestinal tract | Homo sapiens | Colonisation in the gastrointestinal tract | 2013 | Denmark: Copenhagen |
| GCF-009846515.2 | n/a | Homo sapiens | n/a | 2016 | USA |
| GCF-009846545.2 | n/a | Homo sapiens | n/a | 2016 | USA |
| GCF-009846555.2 | n/a | Homo sapiens | n/a | 2016 | USA |
| GCF-009846615.2 | n/a | Homo sapiens | n/a | 2016 | USA |
| GCF-009937865.1 | River water | n/a | n/a | 2016 | USA |
| GCF-009938285.1 | n/a | Homo sapiens | n/a | 2016 | USA:Texas |
| GCF-010671845.1 | Yoghurt | n/a | n/a | 2016 | USA |
| GCF-010722195.1 | Clinical isolate | Homo sapiens | n/a | 2016 | USA |
| GCF-004368135.1 | Rectal swab | Homo sapiens | Rectal carriage | 2013 | France: Boulogne-Billancourt |
| GCF-010722315.1 | Clinical isolate | Homo sapiens | n/a | 2012 | Egypt:Cairo |
| GCF-010722435.1 | Clinical isolate | Homo sapiens | n/a | 2009 | New Zealand |
| GCF-010722535.1 | Clinical isolate | Homo sapiens | n/a | 2009 | New Zealand |
| GCF-010722575.1 | Clinical isolate | Homo sapiens | n/a | 2008 | New Zealand |
| GCF-010722695.1 | Clinical isolate | Homo sapiens | n/a | 2008 | New Zealand |
| GCF-010722815.1 | Clinical isolate | Homo sapiens | n/a | 2008 | New Zealand |
| GCF-011174695.1 | Pollen granules from beehives | n/a | n/a | 2007 | New Zealand |
| GCF-011745645.1 | Stool | Bos taurus | n/a | 2004 | New Zealand |
| GCF-012045365.1 | Fermented dairy products | n/a | n/a | 2003 | New Zealand |
| GCF-012045505.1 | Fermented dairy products | n/a | n/a | 2008 | New Zealand |
| GCF-012063445.1 | Chinese sauerkraut | n/a | n/a | 2008 | New Zealand |
| GCF-013201055.1 | n/a | Homo sapiens | n/a | 2008 | New Zealand |
| GCF-013867835.1 | n/a | n/a | n/a | 2008 | New Zealand |
| GCF-014332725.1 | Fermented soybean past | n/a | n/a | 2008 | New Zealand |
| GCF-014889535.1 | Chicken feces | n/a | n/a | 2008 | New Zealand |
| GCF-015325925.1 | Rectal swab | Homo sapiens | n/a | 2008 | New Zealand |
| GCF-015377765.1 | Probitic products | n/a | n/a | 2008 | New Zealand |
| GCF-015476295.1 | Rectal swab | Homo sapiens | n/a | 2007 | New Zealand |
| GCF-015546985.1 | Stool | Homo sapiens | n/a | 2007 | New Zealand |
| GCF-015549165.1 | Stool | Homo sapiens | n/a | 2007 | New Zealand |
| GCF-015549475.1 | Stool | Homo sapiens | n/a | 2007 | New Zealand |
| GCF-015549905.1 | Stool | Homo sapiens | n/a | 2007 | New Zealand |
| GCF-015550935.1 | Stool | Homo sapiens | n/a | 2005 | New Zealand |
| GCF-015551885.1 | Stool | Homo sapiens | n/a | 2005 | New Zealand |
| GCF-015552705.1 | Stool | Homo sapiens | n/a | 2003 | New Zealand |
| GCF-015554345.1 | Stool | Homo sapiens | n/a | 2001 | New Zealand |
| GCF-015554935.1 | Stool | Homo sapiens | n/a | 2001 | New Zealand |
| GCF-015560445.1 | Stool | Homo sapiens | n/a | 2003 | New Zealand |
| GCF-015667175.1 | Stool | Homo sapiens | n/a | 2001 | New Zealand |
| GCF-015668995.1 | Stool | Homo sapiens | n/a | 2018 | China: Shenzhen |
| GCF-005517315.1 | Pus | Homo sapiens | Abscess | 2019 | India |
| GCF-005576735.1 | Pus | Homo sapiens | Abscess | 2019 | India |
| GCF-015669035.1 | Stool | Homo sapiens | n/a | 2017 | USA |
| GCF-015669235.1 | Stool | Homo sapiens | n/a | 2018 | USA:Arkansas |
| GCF-015670515.1 | Stool | Homo sapiens | n/a | 2018 | USA:Arkansas |
| GCF-015671075.1 | Stool | Homo sapiens | n/a | 2018 | USA:Arkansas |
| GCF-015706535.1 | Rectal swab | Homo sapiens | n/a | 2018 | USA:Arkansas |
| GCF-015706555.1 | Rectal swab | Homo sapiens | n/a | 2015 | USA: Houston |
| GCF-006007925.1 | Urine | Homo sapiens | urinary tract infection | 2018 | China:Hangzhou |
| GCF-015767695.1 | n/a | n/a | n/a | 2018 | USA:Delaware |
| GCF-015767955.1 | Rectal swab | Homo sapiens | n/a | 2019 | South Korea |
| GCF-015767965.1 | Rectal swab | Homo sapiens | n/a | 2019 | South Korea |
| GCF-015767975.1 | Rectal swab | Homo sapiens | n/a | 2019 | South Korea |
| GCF-015767985.1 | Rectal swab | Homo sapiens | n/a | 2015 | USA:Maryland |
| GCF-006575625.1 | Clinical isolate | Homo sapiens | Endocarditis | 2015 | USA: Houston |
| GCF-015767995.1 | Rectal swab | Homo sapiens | n/a | 2013 | Egypt:Kafr-El-Sheik governorate |
| GCF-015768055.1 | Rectal swab | Homo sapiens | n/a | 2016 | Russia: Orenburg |
| GCF-007917035.2 | Stool | Homo sapiens | Liver failure | 2014 | Sweden:Halmstad |
| GCF-007917315.3 | Stool | Homo sapiens | Liver failure | 2014 | Sweden:Halmstad |
| GCF-015768065.1 | Rectal swab | Homo sapiens | n/a | 2017 | China: Henan |
| GCF-015768105.1 | Rectal swab | Homo sapiens | n/a | 2017 | China: HeBei |
| GCF-015768255.1 | Rectal swab | Homo sapiens | n/a | 2019 | South Korea |
| GCF-015768285.1 | Rectal swab | Homo sapiens | n/a | 2016 | Netherlands:Maastricht |
| GCF-015768335.1 | Rectal swab | Homo sapiens | n/a | 2019 | South Korea: Seosan |
| GCF-008728455.1 | Rectal swab | Homo sapiens | Infection | 2017 | Denmark:Copenhagen |
| GCF-008728475.1 | Rectal swab | Homo sapiens | Infection | 2018 | Denmark:Copenhagen |
| GCF-015768355.1 | Rectal swab | Homo sapiens | n/a | 2012 | Brazil |
| GCF-008921735.1 | Urine | Homo sapiens | Bacterial infection | 2012 | Brazil |
| GCF-008921805.1 | Muscle biopsy | Homo sapiens | Infection | 2012 | Brazil: Sao Jose do Rio Preto |
| GCF-008921825.1 | Urine | Homo sapiens | Bacterial infection | 2012 | Brazil |
| GCF-008921845.1 | Urine | Homo sapiens | Bacterial infection | 2012 | Brazil |
| GCF-008921905.1 | Urine | Homo sapiens | urinary tract infection | 2012 | Brazil |
| GCF-008921925.1 | Urine | Homo sapiens | urinary tract infection | 2012 | Brazil |
| GCF-008921935.1 | Urine | Homo sapiens | urinary tract infection | 2012 | Brazil |
| GCF-008922055.1 | Urine | Homo sapiens | Infection | 2012 | Brazil: Sao Jose do Rio Preto |
| GCF-015768365.1 | Rectal swab | Homo sapiens | n/a | 2015 | Brazil: Parana |
| GCF-009078405.1 | Ascitic Fluid | Homo sapiens | Infection | 2012 | Brazil: Sao Jose do Rio Preto |
| GCF-009078415.1 | Broncoalveolar lavage | Homo sapiens | Infection | 2012 | Brazil: Sao Jose do Rio Preto |
| GCF-015768375.1 | Rectal swab | Homo sapiens | n/a | 2015 | USA |
| GCF-015768415.1 | Rectal swab | Homo sapiens | n/a | 2015 | USA |
| GCF-015768425.1 | Rectal swab | Homo sapiens | n/a | 2017 | Canada |
| GCF-015768455.1 | Rectal swab | Homo sapiens | n/a | 2018 | China:Hangzhou |
| GCF-009734005.1 | Clinical isolate | Homo sapiens | Choledocholithiasis | 2017 | China:Hangzhou |
| GCF-015768475.1 | Rectal swab | Homo sapiens | n/a | n/a | n/a |
| GCF-015768485.1 | Rectal swab | Homo sapiens | n/a | n/a | n/a |
| GCF-015768495.1 | Rectal swab | Homo sapiens | n/a | 2017 | USA:Pennsylvania |

| | | | | | |
|---|---|---|---|---|---|
| GCF-015768535.1 | Rectal swab | Homo sapiens | n/a | 2017 | USA:Pennsylvania |
| GCF-015768555.1 | Rectal swab | Homo sapiens | n/a | n/a | Russia: Moscow |
| GCF-015768585.1 | Rectal swab | Homo sapiens | n/a | n/a | Russia: Moscow |
| GCF-015768775.1 | Rectal swab | Homo sapiens | n/a | n/a | Russia: Moscow |
| GCF-015768785.1 | Urine | Homo sapiens | n/a | n/a | Russia: Moscow |
| GCF-015768815.1 | Urine | Homo sapiens | n/a | 2013 | India:River Yamuna, Delhi |
| GCF-009938075.1 | Urine | Homo sapiens | Cancer of anus | 2017 | China: Hangzhou |
| GCF-015768835.1 | Rectal swab | Homo sapiens | n/a | n/a | n/a |
| GCF-010119315.1 | Blood | Homo sapiens | Bacteremia | 2018 | USA: Chicago |
| GCF-010119375.1 | Blood | Homo sapiens | Bacteremia | 2018 | USA: Chicago |
| GCF-015768845.1 | Rectal swab | Homo sapiens | n/a | 2017 | China:Hangzhou |
| GCF-015768875.1 | Rectal swab | Homo sapiens | n/a | 1969 | Bulgaria:Sofia |
| GCF-015768895.1 | Rectal swab | Homo sapiens | n/a | 2016 | Netherlands |
| GCF-015768905.1 | Rectal swab | Homo sapiens | n/a | 2016 | Netherlands |
| GCF-015768915.1 | Rectal swab | Homo sapiens | n/a | 2016 | Netherlands |
| GCF-015768945.1 | Rectal swab | Homo sapiens | n/a | 2016 | Netherlands |
| GCF-015768975.1 | Rectal swab | Homo sapiens | n/a | 2016 | Netherlands |
| GCF-015768985.1 | Rectal swab | Homo sapiens | n/a | 2016 | Netherlands |
| GCF-015769005.1 | Rectal swab | Homo sapiens | n/a | 2016 | Netherlands |
| GCF-015769025.1 | Rectal swab | Homo sapiens | n/a | 2019 | Bulgaria: Dushantzi Village |
| GCF-011386845.1 | Bronchial secretion | Homo sapiens | Infection | 2005 | Colombia: Bogota |
| GCF-011386855.1 | Surgical wound | Homo sapiens | Infection | 2001 | Colombia: Bogota |
| GCF-011386865.1 | Surgical wound | Homo sapiens | Infection | 2001 | Colombia: Bogota |
| GCF-015769035.1 | Rectal swab | Homo sapiens | n/a | 2017 | China:qinghai |
| GCF-015769075.1 | Rectal swab | Homo sapiens | n/a | 2018 | South Korea: Seoul |
| GCF-015769095.1 | Rectal swab | Homo sapiens | n/a | 2018 | South Korea: Seoul |
| GCF-015769105.1 | Rectal swab | Homo sapiens | n/a | 2016 | China:Guangdong |
| GCF-015769115.1 | Rectal swab | Homo sapiens | n/a | 2018 | India:Vellore |
| GCF-015769155.1 | Rectal swab | Homo sapiens | n/a | 2017 | India:Vellore |
| GCF-015769175.1 | Rectal swab | Homo sapiens | n/a | 2019 | India:Vellore |
| GCF-015769195.1 | Rectal swab | Homo sapiens | n/a | 2017 | India:Vellore |
| GCF-015769215.1 | Rectal swab | Homo sapiens | n/a | 2019 | India:Vellore |
| GCF-015769225.1 | Rectal swab | Homo sapiens | n/a | 2019 | India:Vellore |
| GCF-015769235.1 | Rectal swab | Homo sapiens | n/a | 2019 | India:Vellore |
| GCF-015769275.1 | Rectal swab | Homo sapiens | n/a | 2019 | India:Vellore |
| GCF-015769455.1 | Rectal swab | Homo sapiens | n/a | 2019 | India:Vellore |
| GCF-015769495.1 | Rectal swab | Homo sapiens | n/a | 2019 | India:Vellore |
| GCF-015769515.1 | Rectal swab | Homo sapiens | n/a | 2019 | India:Vellore |
| GCF-015769535.1 | Rectal swab | Homo sapiens | n/a | 2016 | Russia: Pushchino |
| GCF-015769545.1 | Rectal swab | Homo sapiens | n/a | 2020 | n/a |
| GCF-015769555.1 | Rectal swab | Homo sapiens | n/a | 2015 | South Korea: Chuncheon |
| GCF-015769595.1 | Rectal swab | Homo sapiens | n/a | 2016 | Germany:Cologne |
| GCF-015769615.1 | Rectal swab | Homo sapiens | n/a | 2016 | Germany:Cologne |
| GCF-015769635.1 | Urine | Homo sapiens | n/a | 2019 | India |
| GCF-015769645.1 | Rectal swab | Homo sapiens | n/a | 2015 | South Korea |
| GCF-015769655.1 | Wound | Homo sapiens | n/a | 2014 | Australia |
| GCF-015769685.1 | Wound | Homo sapiens | n/a | 2019 | China:Zhejiang province |
| GCF-015769715.1 | Blood | Homo sapiens | n/a | 2014 | Australia |
| GCF-015769735.1 | Rectal swab | Homo sapiens | n/a | 2016 | USA |
| GCF-015769745.1 | Rectal swab | Homo sapiens | n/a | 2016 | USA |
| GCF-015769755.1 | Rectal swab | Homo sapiens | n/a | 2017 | USA |
| GCF-015769785.1 | Rectal swab | Homo sapiens | n/a | 2016 | USA |
| GCF-015769815.1 | Rectal swab | Homo sapiens | n/a | 2015 | USA |
| GCF-015769845.1 | Rectal swab | Homo sapiens | n/a | 2018 | USA |
| GCF-015770015.1 | Rectal swab | Homo sapiens | n/a | 2015 | USA |
| GCF-015770075.1 | Rectal swab | Homo sapiens | n/a | 2016 | USA |
| GCF-015770085.1 | Rectal swab | Homo sapiens | n/a | 2016 | USA |
| GCF-015770105.1 | Rectal swab | Homo sapiens | n/a | 2016 | USA |
| GCF-015770125.1 | Rectal swab | Homo sapiens | n/a | 2017 | USA |
| GCF-015770155.1 | Rectal swab | Homo sapiens | n/a | 2017 | USA |
| GCF-015770175.1 | Rectal swab | Homo sapiens | n/a | 2016 | USA |
| GCF-015770185.1 | Rectal swab | Homo sapiens | n/a | 2017 | USA |
| GCF-015770215.1 | Rectal swab | Homo sapiens | n/a | 2017 | USA |
| GCF-015770225.1 | Urine | Homo sapiens | n/a | 2016 | USA |
| GCF-015770255.1 | Wound | Homo sapiens | n/a | 2018 | USA: Pennsylvania |
| GCF-015770275.1 | Wound | Homo sapiens | n/a | 2018 | USA: Pennsylvania |
| GCF-015770295.1 | Rectal swab | Homo sapiens | n/a | n/a | n/a |
| GCF-015770305.1 | Rectal swab | Homo sapiens | n/a | 2018 | USA: Pennsylvania |
| GCF-015770315.1 | Rectal swab | Homo sapiens | n/a | 2018 | USA: Pennsylvania |
| GCF-015770325.1 | Rectal swab | Homo sapiens | n/a | 2018 | USA: Pennsylvania |
| GCF-015770375.1 | Rectal swab | Homo sapiens | n/a | 2018 | USA: Pennsylvania |
| GCF-015770445.1 | Rectal swab | Homo sapiens | n/a | 2018 | USA: Pennsylvania |
| GCF-015770495.1 | Rectal swab | Homo sapiens | n/a | 2018 | USA: Pennsylvania |
| GCF-015770505.1 | Rectal swab | Homo sapiens | n/a | 2018 | USA: Pennsylvania |
| GCF-015770515.1 | Rectal swab | Homo sapiens | n/a | 2018 | USA: Pennsylvania |
| GCF-015770535.1 | Rectal swab | Homo sapiens | n/a | 2018 | USA: Pennsylvania |
| GCF-015770575.1 | Rectal swab | Homo sapiens | n/a | 2018 | USA: Pennsylvania |
| GCF-015770595.1 | Rectal swab | Homo sapiens | n/a | 2018 | USA: Pennsylvania |
| GCF-015770635.1 | Rectal swab | Homo sapiens | n/a | 2018 | USA: Pennsylvania |
| GCF-015770645.1 | Rectal swab | Homo sapiens | n/a | 2018 | USA: Pennsylvania |
| GCF-015770675.1 | Rectal swab | Homo sapiens | n/a | 2018 | USA: Pennsylvania |
| GCF-015770685.1 | Rectal swab | Homo sapiens | n/a | 2018 | USA: Pennsylvania |
| GCF-015770695.1 | Rectal swab | Homo sapiens | n/a | 2018 | USA: Pennsylvania |
| GCF-015770735.1 | Rectal swab | Homo sapiens | n/a | 2018 | USA: Pennsylvania |
| GCF-015770755.1 | Rectal swab | Homo sapiens | n/a | 2018 | USA: Pennsylvania |
| GCF-015770765.1 | Rectal swab | Homo sapiens | n/a | 2018 | USA: Pennsylvania |
| GCF-015770785.1 | Rectal swab | Homo sapiens | n/a | 2018 | USA: Pennsylvania |
| GCF-015771145.1 | Rectal swab | Homo sapiens | n/a | 2018 | USA: Pennsylvania |
| GCF-015771175.1 | Rectal swab | Homo sapiens | n/a | 2018 | USA: Pennsylvania |

| | | | | | |
|---|---|---|---|---|---|
| GCF-015771195.1 | Rectal swab | Homo sapiens | n/a | 2018 | USA: Pennsylvania |
| GCF-015771205.1 | Rectal swab | Homo sapiens | n/a | 2018 | USA: Pennsylvania |
| GCF-015771215.1 | Rectal swab | Homo sapiens | n/a | 2018 | USA: Pennsylvania |
| GCF-015771255.1 | Rectal swab | Homo sapiens | n/a | 2018 | USA: Pennsylvania |
| GCF-015771295.1 | Rectal swab | Homo sapiens | n/a | 2018 | USA: Pennsylvania |
| GCF-015771305.1 | Rectal swab | Homo sapiens | n/a | 2018 | USA: Pennsylvania |
| GCF-015771315.1 | Rectal swab | Homo sapiens | n/a | 2018 | USA: Pennsylvania |
| GCF-015771355.1 | Rectal swab | Homo sapiens | n/a | 2018 | USA: Pennsylvania |
| GCF-015771365.1 | Rectal swab | Homo sapiens | n/a | 2018 | USA: Pennsylvania |
| GCF-015771395.1 | Rectal swab | Homo sapiens | n/a | 2018 | USA: Pennsylvania |
| GCF-015771405.1 | Rectal swab | Homo sapiens | n/a | 2018 | USA: Pennsylvania |
| GCF-015771435.1 | Rectal swab | Homo sapiens | n/a | 2018 | USA: Pennsylvania |
| GCF-015771445.1 | Rectal swab | Homo sapiens | n/a | 2018 | USA: Pennsylvania |
| GCF-015771465.1 | Urine | Homo sapiens | n/a | 2018 | USA: Pennsylvania |
| GCF-015771495.1 | Rectal swab | Homo sapiens | n/a | 2018 | USA: Pennsylvania |
| GCF-015771515.1 | Rectal swab | Homo sapiens | n/a | 2018 | USA: Pennsylvania |
| GCF-015771525.1 | Rectal swab | Homo sapiens | n/a | 2018 | USA: Pennsylvania |
| GCF-015771555.1 | Rectal swab | Homo sapiens | n/a | 2018 | USA: Pennsylvania |
| GCF-015771565.1 | Rectal swab | Homo sapiens | n/a | 2018 | USA: Pennsylvania |
| GCF-015771595.1 | Rectal swab | Homo sapiens | n/a | 2018 | USA: Pennsylvania |
| GCF-015771615.1 | Rectal swab | Homo sapiens | n/a | 2018 | USA: Pennsylvania |
| GCF-015771635.1 | Rectal swab | Homo sapiens | n/a | 2018 | USA: Pennsylvania |
| GCF-015771655.1 | Rectal swab | Homo sapiens | n/a | 2018 | USA: Pennsylvania |
| GCF-015771675.1 | Rectal swab | Homo sapiens | n/a | 2018 | USA: Pennsylvania |
| GCF-015771695.1 | Rectal swab | Homo sapiens | n/a | 2018 | USA: Pennsylvania |
| GCF-015771785.1 | Rectal swab | Homo sapiens | n/a | 2018 | USA: Pennsylvania |
| GCF-015771835.1 | Urine | Homo sapiens | n/a | 2018 | USA: Pennsylvania |
| GCF-015771845.1 | Rectal swab | Homo sapiens | n/a | 2018 | USA: Pennsylvania |
| GCF-015771855.1 | Rectal swab | Homo sapiens | n/a | 2018 | USA: Pennsylvania |
| GCF-015771935.1 | Rectal swab | Homo sapiens | n/a | 2018 | USA: Pennsylvania |
| GCF-015771955.1 | Rectal swab | Homo sapiens | n/a | 2018 | USA: Pennsylvania |
| GCF-015771965.1 | Rectal swab | Homo sapiens | n/a | 2018 | USA: Pennsylvania |
| GCF-015771975.1 | Rectal swab | Homo sapiens | n/a | 2018 | USA: Pennsylvania |
| GCF-015772015.1 | Rectal swab | Homo sapiens | n/a | 2017 | USA: Pennsylvania |
| GCF-015772055.1 | Rectal swab | Homo sapiens | n/a | 2017 | USA: Pennsylvania |
| GCF-015772075.1 | Rectal swab | Homo sapiens | n/a | 2018 | USA: Pennsylvania |
| GCF-015772085.1 | Rectal swab | Homo sapiens | n/a | 2017 | USA: Pennsylvania |
| GCF-015772115.1 | Rectal swab | Homo sapiens | n/a | 2018 | USA: Pennsylvania |
| GCF-015772135.1 | Rectal swab | Homo sapiens | n/a | 2017 | USA: Pennsylvania |
| GCF-015772145.1 | Rectal swab | Homo sapiens | n/a | 2018 | USA: Pennsylvania |
| GCF-015772165.1 | Rectal swab | Homo sapiens | n/a | 2017 | USA: Pennsylvania |
| GCF-015772185.1 | Rectal swab | Homo sapiens | n/a | 2017 | USA: Pennsylvania |
| GCF-015772215.1 | Rectal swab | Homo sapiens | n/a | 2017 | USA: Pennsylvania |
| GCF-015772235.1 | Rectal swab | Homo sapiens | n/a | 2017 | USA: Pennsylvania |
| GCF-015772245.1 | Rectal swab | Homo sapiens | n/a | 2017 | USA: Pennsylvania |
| GCF-015772255.1 | Rectal swab | Homo sapiens | n/a | 2017 | USA: Pennsylvania |
| GCF-015772265.1 | Rectal swab | Homo sapiens | n/a | 2017 | USA: Pennsylvania |
| GCF-015772285.1 | Rectal swab | Homo sapiens | n/a | 2017 | USA: Pennsylvania |
| GCF-015772335.1 | Rectal swab | Homo sapiens | n/a | 2017 | USA: Pennsylvania |
| GCF-015772345.1 | Rectal swab | Homo sapiens | n/a | 2017 | USA: Pennsylvania |
| GCF-015772355.1 | Rectal swab | Homo sapiens | n/a | 2017 | USA: Pennsylvania |
| GCF-015772375.1 | Rectal swab | Homo sapiens | n/a | 2017 | USA: Pennsylvania |
| GCF-015772405.1 | Rectal swab | Homo sapiens | n/a | 2017 | USA: Pennsylvania |
| GCF-015772435.1 | Rectal swab | Homo sapiens | n/a | 2017 | USA: Pennsylvania |
| GCF-015772455.1 | Rectal swab | Homo sapiens | n/a | 2017 | USA: Pennsylvania |
| GCF-015772465.1 | Rectal swab | Homo sapiens | n/a | 2017 | USA: Pennsylvania |
| GCF-015772485.1 | Rectal swab | Homo sapiens | n/a | 2017 | USA: Pennsylvania |
| GCF-015772515.1 | Rectal swab | Homo sapiens | n/a | 2017 | USA: Pennsylvania |
| GCF-015772535.1 | Rectal swab | Homo sapiens | n/a | 2017 | USA: Pennsylvania |
| GCF-015772995.1 | Rectal swab | Homo sapiens | n/a | 2017 | USA: Pennsylvania |
| GCF-015773285.1 | Rectal swab | Homo sapiens | n/a | 2017 | USA: Pennsylvania |
| GCF-015773435.1 | Rectal swab | Homo sapiens | n/a | 2017 | USA: Pennsylvania |
| GCF-015773445.1 | Rectal swab | Homo sapiens | n/a | 2017 | USA: Pennsylvania |
| GCF-015773455.1 | Rectal swab | Homo sapiens | n/a | 2017 | USA: Pennsylvania |
| GCF-015773745.1 | Blood | Homo sapiens | n/a | 2017 | USA: Pennsylvania |
| GCF-015773895.1 | Rectal swab | Homo sapiens | n/a | 2017 | USA: Pennsylvania |
| GCF-015773905.1 | Rectal swab | Homo sapiens | n/a | 2017 | USA: Pennsylvania |
| GCF-015773955.1 | Rectal swab | Homo sapiens | n/a | 2017 | USA: Pennsylvania |
| GCF-015774275.1 | Urine | Homo sapiens | n/a | 2017 | USA: Pennsylvania |
| GCF-015774295.1 | Rectal swab | Homo sapiens | n/a | 2017 | USA: Pennsylvania |
| GCF-015999405.1 | Pleural fluid | Homo sapiens | n/a | 2017 | USA: Pennsylvania |
| GCF-015999425.1 | Rectal swab | Homo sapiens | n/a | 2017 | USA: Pennsylvania |
| GCF-015999605.1 | Rectal swab | Homo sapiens | n/a | 2017 | USA: Pennsylvania |
| GCF-016126675.1 | Rectal swab | Homo sapiens | n/a | 2017 | USA: Pennsylvania |
| GCF-016406345.1 | Rectal swab | Homo sapiens | n/a | 2017 | USA: Pennsylvania |
| GCF-016406365.1 | Rectal swab | Homo sapiens | n/a | 2017 | USA: Pennsylvania |
| GCF-016406385.1 | Rectal swab | Homo sapiens | n/a | 2017 | USA: Pennsylvania |
| GCF-016406405.1 | Rectal swab | Homo sapiens | n/a | 2017 | USA: Pennsylvania |
| GCF-016406425.1 | Rectal swab | Homo sapiens | n/a | 2017 | USA: Pennsylvania |
| GCF-016406445.1 | Rectal swab | Homo sapiens | n/a | 2017 | USA: Pennsylvania |
| GCF-016406465.1 | Rectal swab | Homo sapiens | n/a | 2017 | USA: Pennsylvania |
| GCF-016406485.1 | Rectal swab | Homo sapiens | n/a | 2017 | USA: Pennsylvania |
| GCF-016406505.1 | Rectal swab | Homo sapiens | n/a | 2017 | USA: Pennsylvania |
| GCF-016406525.1 | Rectal swab | Homo sapiens | n/a | 2017 | USA: Pennsylvania |
| GCF-016406545.1 | Rectal swab | Homo sapiens | n/a | 2017 | USA: Pennsylvania |
| GCF-016406565.1 | Rectal swab | Homo sapiens | n/a | 2017 | USA: Pennsylvania |
| GCF-016406585.1 | Rectal swab | Homo sapiens | n/a | 2017 | USA: Pennsylvania |
| GCF-016406605.1 | Rectal swab | Homo sapiens | n/a | 2017 | USA: Pennsylvania |
| GCF-016415025.1 | Rectal swab | Homo sapiens | n/a | 2017 | USA: Pennsylvania |

| | | | | | |
|---|---|---|---|---|---|
| GCF-016415105.1 | Rectal swab | Homo sapiens | n/a | 2017 | USA: Pennsylvania |
| GCF-016415285.1 | Rectal swab | Homo sapiens | n/a | 2017 | USA: Pennsylvania |
| GCF-016415325.1 | Rectal swab | Homo sapiens | n/a | 2017 | USA: Pennsylvania |
| GCF-016415345.1 | Rectal swab | Homo sapiens | n/a | 2017 | USA: Pennsylvania |
| GCF-016415365.1 | Rectal swab | Homo sapiens | n/a | 2017 | USA: Pennsylvania |
| GCF-016415385.1 | Rectal swab | Homo sapiens | n/a | 2017 | USA: Pennsylvania |
| GCF-016415405.1 | Rectal swab | Homo sapiens | n/a | 2017 | USA: Pennsylvania |
| GCF-016415425.1 | Rectal swab | Homo sapiens | n/a | 2017 | USA: Pennsylvania |
| GCF-016415445.1 | Rectal swab | Homo sapiens | n/a | 2017 | USA: Pennsylvania |
| GCF-016415465.1 | Rectal swab | Homo sapiens | n/a | 2017 | USA: Pennsylvania |
| GCF-016415485.1 | Rectal swab | Homo sapiens | n/a | 2017 | USA: Pennsylvania |
| GCF-016415505.1 | Rectal swab | Homo sapiens | n/a | 2017 | USA: Pennsylvania |
| GCF-016415545.1 | Rectal swab | Homo sapiens | n/a | 2017 | USA: Pennsylvania |
| GCF-016415565.1 | Rectal swab | Homo sapiens | n/a | 2017 | USA: Pennsylvania |
| GCF-016455345.1 | Rectal swab | Homo sapiens | n/a | 2017 | USA: Pennsylvania |
| GCF-016743855.1 | Swine | n/a | n/a | 2017 | USA: Pennsylvania |
| GCF-017584065.1 | Rectal swab | Homo sapiens | n/a | 2017 | USA: Pennsylvania |
| GCF-017641685.1 | Clinical isolate | Hospital environment | n/a | 2017 | USA: Pennsylvania |
| GCF-017642135.1 | Peadiatric - unoccupied bed | Hospital environment | n/a | 2017 | USA: Pennsylvania |
| GCF-018219285.1 | Rectal swab | Homo sapiens | n/a | 2017 | USA: Pennsylvania |
| GCF-018219305.1 | Rectal swab | Homo sapiens | n/a | 2017 | USA: Pennsylvania |
| GCF-018219325.1 | Rectal swab | Homo sapiens | n/a | 2017 | USA: Pennsylvania |
| GCF-018219345.1 | Rectal swab | Homo sapiens | n/a | 2017 | USA: Pennsylvania |
| GCF-018279145.1 | Animal | n/a | n/a | 2017 | USA: Pennsylvania |
| GCF-018296025.1 | Rectal swab | Homo sapiens | n/a | 2017 | USA: Pennsylvania |
| GCF-018516845.1 | Digestive | Homo sapiens | n/a | 2017 | USA: Pennsylvania |
| GCF-018516925.1 | Rectal swab | Homo sapiens | n/a | 2017 | USA: Pennsylvania |
| GCF-018517045.1 | Urine | Homo sapiens | n/a | 2017 | USA: Pennsylvania |
| GCF-018517065.1 | Digestive | Homo sapiens | n/a | 2017 | USA: Pennsylvania |
| GCF-018517085.1 | Urine | Homo sapiens | n/a | 2017 | USA: Pennsylvania |
| GCF-018517105.1 | Rectal swab | Homo sapiens | n/a | 2017 | USA: Pennsylvania |
| GCF-018517185.1 | Digestive | Homo sapiens | n/a | 2017 | USA: Pennsylvania |
| GCF-018784845.1 | Stool | Homo sapiens | n/a | 2017 | USA: Pennsylvania |
| GCF-019042295.1 | n/a | Sus domesticus | n/a | 2017 | USA: Pennsylvania |
| GCF-019175425.1 | Stream surface water | n/a | n/a | 2017 | USA: Pennsylvania |
| GCF-019175445.1 | Stream surface water | n/a | n/a | 2017 | USA: Pennsylvania |
| GCF-019175465.1 | Stream surface water | n/a | n/a | 2017 | USA: Pennsylvania |
| GCF-019175525.1 | Stream surface water | n/a | n/a | 2017 | USA: Pennsylvania |
| GCF-019356355.1 | Secretion | Homo sapiens | n/a | 2017 | USA: Pennsylvania |
| GCF-019456555.1 | Stream surface water | n/a | n/a | 2017 | USA: Pennsylvania |
| GCF-019456575.1 | River water | n/a | n/a | 2017 | USA: Pennsylvania |
| GCF-019456595.1 | Stream surface water | n/a | n/a | 2017 | USA: Pennsylvania |
| GCF-019731045.1 | Stool | Homo sapiens | n/a | 2017 | USA: Pennsylvania |
| GCF-019731095.1 | Stool | Homo sapiens | n/a | 2017 | USA: Pennsylvania |
| GCF-019731315.1 | Stool | Homo sapiens | n/a | 2017 | USA: Pennsylvania |
| GCF-019731655.1 | Stool | Homo sapiens | n/a | 2017 | USA: Pennsylvania |
| GCF-019731665.1 | Stool | Homo sapiens | n/a | 2017 | USA: Pennsylvania |
| GCF-019733825.1 | Stool | Homo sapiens | n/a | 2017 | USA: Pennsylvania |
| GCF-019733855.1 | Stool | Homo sapiens | n/a | 2017 | USA: Pennsylvania |
| GCF-019733915.1 | Stool | Homo sapiens | n/a | 2017 | USA: Pennsylvania |
| GCF-019733965.1 | Stool | Homo sapiens | n/a | 2017 | USA: Pennsylvania |
| GCF-019734055.1 | Stool | Homo sapiens | n/a | 2017 | USA: Pennsylvania |
| GCF-019734075.1 | Stool | Homo sapiens | n/a | 2017 | USA: Pennsylvania |
| GCF-019734085.1 | Stool | Homo sapiens | n/a | 2017 | USA: Pennsylvania |
| GCF-019734135.1 | Stool | Homo sapiens | n/a | 2017 | USA: Pennsylvania |
| GCF-019780105.1 | n/a | Homo sapiens | n/a | 2017 | USA: Pennsylvania |
| GCF-019880125.1 | Urine | Homo sapiens | n/a | 2017 | USA: Pennsylvania |
| GCF-019880145.1 | Clinical isolate | n/a | n/a | 2017 | USA: Pennsylvania |
| GCF-019880155.1 | Clinical isolate | n/a | n/a | 2017 | USA: Pennsylvania |
| GCF-019880185.1 | Clinical isolate | n/a | n/a | 2017 | USA: Pennsylvania |
| GCF-019973595.1 | Blood | Homo sapiens | n/a | 2017 | USA: Pennsylvania |
| GCF-019977495.1 | Clinical isolate | Homo sapiens | n/a | 2017 | USA: Pennsylvania |
| GCF-019977575.1 | Clinical isolate | Homo sapiens | n/a | 2014 | Australia |
| GCF-020091325.1 | Clinical isolate | Homo sapiens | n/a | 2014 | Australia |
| GCF-020162155.1 | Clinical isolate | Homo sapiens | n/a | 2015 | USA: Dallas |
| GCF-020162175.1 | Clinical isolate | Homo sapiens | n/a | 2015 | USA: Dallas |
| GCF-020514405.1 | Soft fresh cheese | n/a | n/a | 2015 | USA: Dallas |
| GCF-020592895.1 | n/a | Equus caballus | n/a | 2015 | USA: Dallas |
| GCF-021560195.1 | n/a | Sus domesticus | n/a | 2015 | USA: Dallas |
| GCF-020736585.1 | n/a | Homo sapiens | n/a | 2015 | USA: Dallas |
| GCF-020736625.1 | n/a | Homo sapiens | n/a | 2015 | USA: Dallas |
| GCF-021121925.1 | Enviromental Sample | n/a | n/a | 2015 | USA: Dallas |
| GCF-021122055.1 | Enviromental Sample | n/a | n/a | 2015 | USA: Dallas |
| GCF-021122345.1 | Enviromental Sample | n/a | n/a | 2015 | USA: Dallas |
| GCF-021122425.1 | Enviromental Sample | n/a | n/a | 2015 | USA: Dallas |
| GCF-021122605.1 | Enviromental Sample | n/a | n/a | 2015 | USA: Dallas |
| GCF-021122805.1 | Enviromental Sample | n/a | n/a | 2015 | USA: Dallas |
| GCF-021122985.1 | Enviromental Sample | n/a | n/a | 2015 | USA: Dallas |
| GCF-021123205.1 | Enviromental Sample | n/a | n/a | 2015 | USA: Dallas |
| GCF-021123345.1 | Enviromental Sample | n/a | n/a | 2015 | USA: Dallas |
| GCF-021123545.1 | Enviromental Sample | n/a | n/a | 2015 | USA: Dallas |
| GCF-021124145.1 | Enviromental Sample | n/a | n/a | 2015 | USA: Dallas |
| GCF-022426705.1 | Lung | Sus domesticus | n/a | 2015 | USA: Dallas |
| GCF-021398505.1 | n/a | Apis mellifera | n/a | 2015 | USA: Dallas |
| GCF-023182815.2 | n/a | Sus domesticus | n/a | 2015 | USA: Dallas |
| GCF-021713095.1 | n/a | Homo sapiens | n/a | 2015 | USA: Dallas |
| GCF-021899455.1 | n/a | Homo sapiens | n/a | 2015 | USA: Dallas |
| GCF-023375205.1 | Stool | Sus domesticus | n/a | 2015 | USA: Dallas |
| GCF-022509525.1 | Dahi fermented milk product | n/a | n/a | 2015 | USA: Dallas |

| | | | | | |
|---|---|---|---|---|---|
| GCF-022509555.1 | Dahi fermented milk product | n/a | n/a | 2015 | USA: Dallas |
| GCF-022509585.1 | Dahi fermented milk product | n/a | n/a | 2015 | USA: Dallas |
| GCF-022647825.1 | Blood | Homo sapiens | n/a | 2015 | USA: Dallas |
| GCF-022647945.1 | Blood | Homo sapiens | n/a | 2015 | USA: Dallas |
| GCF-022648125.1 | Blood | Homo sapiens | n/a | 2015 | USA: Dallas |
| GCF-022749455.1 | n/a | Homo sapiens | n/a | 2015 | USA: Dallas |
| GCF-023375225.1 | Stool | Sus domesticus | n/a | 2015 | USA: Dallas |
| GCF-025800545.1 | Enviromental Sample | Sus domesticus | n/a | 2017 | China:henan |
| GCF-016864255.1 | Urine | Homo sapiens | Bacteriuria | 2017 | Norway: Bergen |
| GCF-023299325.1 | Pet food | n/a | n/a | 2014 | Australia |
| GCF-023299725.1 | Pet food | n/a | n/a | 2020 | India |
| GCF-023299745.1 | Pet food | n/a | n/a | 2017 | South Africa: KwaZulu-Natal |
| GCF-023299805.1 | Pet food | n/a | n/a | 2017 | South Africa: KwaZulu-Natal |
| GCF-025800625.1 | Enviromental Sample | Sus domesticus | n/a | 2018 | India |
| GCF-025800645.1 | Enviromental Sample | Sus domesticus | n/a | 2019 | India |
| GCF-023375345.1 | Stool | Homo sapiens | n/a | 2019 | India |
| GCF-023375365.1 | Stool | Homo sapiens | n/a | 2019 | India |
| GCF-023375385.1 | Stool | Homo sapiens | n/a | 2019 | India |
| GCF-023375405.1 | Stool | Homo sapiens | n/a | 2019 | India |
| GCF-023375425.1 | Stool | Homo sapiens | n/a | 2016 | USA: Ann Arbor |
| GCF-023375445.1 | Stool | Homo sapiens | n/a | 2016 | USA: Ann Arbor |
| GCF-023633925.1 | n/a | Homo sapiens | n/a | 2016 | USA: Ann Arbor |
| GCF-023658035.1 | n/a | Homo sapiens | n/a | 2016 | USA: Ann Arbor |
| GCF-023743405.1 | Artisanal Greek Feta cheese | n/a | n/a | 2019 | China:Sichuan |
| GCF-023743415.1 | Artisanal Greek Feta cheese | n/a | n/a | 2016 | USA: Ann Arbor |
| GCF-023743455.1 | Sheep milk | n/a | n/a | 2018 | France: Bordeaux |
| GCF-023743525.1 | Artisanal Greek Kefalograviera cheese | n/a | n/a | 2018 | France: Corbeil-Essonnes |
| GCF-023743555.1 | Artisanal Greek Feta cheese | n/a | n/a | 2017 | France: Amiens |
| GCF-023743575.1 | Artisanal Greek Feta cheese | n/a | n/a | 2017 | France: Rennes |
| GCF-023743585.1 | Artisanal Greek Kefalograviera cheese | n/a | n/a | 2016 | France: Chambery |
| GCF-023743605.1 | Artisanal Greek Kefalograviera cheese | n/a | n/a | 2016 | France: Caen |
| GCF-023743635.1 | Artisanal Greek Kefalograviera cheese | n/a | n/a | 2018 | France: Tourcoing |
| GCF-023743655.1 | Artisanal Greek Kefalograviera cheese | n/a | n/a | n/a | Ireland |
| GCF-023743675.1 | Artisanal Greek Kefalograviera cheese | n/a | n/a | 2014 | Russia: Orenburg |
| GCF-023743695.1 | Artisanal Greek Kefalograviera cheese | n/a | n/a | 2020 | Switzerland: Grenchen |
| GCF-023743715.1 | Artisanal Greek Kefalograviera cheese | n/a | n/a | 2020 | Switzerland: Cazis |
| GCF-023743755.1 | Artisanal Greek Feta cheese | n/a | n/a | 2020 | Switzerland: Martigny |
| GCF-023743775.1 | Artisanal Greek Feta cheese | n/a | n/a | 2020 | Switzerland: Zug |
| GCF-023743815.1 | Artisanal Greek Kefalograviera cheese | n/a | n/a | 2018 | China:Chongqing |
| GCF-023743895.1 | Artisanal Greek Kefalograviera cheese | n/a | n/a | 2020 | Switzerland: Kerzers |
| GCF-023743905.1 | Artisanal Greek Kefalograviera cheese | n/a | n/a | 2020 | Switzerland: Cazis |
| GCF-023743935.1 | Artisanal Greek Kefalograviera cheese | n/a | n/a | 2020 | Switzerland: Altstaetten |
| GCF-023744135.1 | Artisanal Greek Feta cheese | n/a | n/a | 2020 | China |
| GCF-023744175.1 | Artisanal Greek Feta cheese | n/a | n/a | 2020 | China |
| GCF-023744215.1 | Artisanal Greek Kefalograviera cheese | n/a | n/a | 2020 | China |
| GCF-023744235.1 | Artisanal Greek Feta cheese | n/a | n/a | 2020 | China |
| GCF-023744275.1 | Artisanal Greek Kefalograviera cheese | n/a | n/a | 2020 | China |
| GCF-024372785.1 | Gut | Penaeus vannamei | n/a | 2020 | China |
| GCF-024665675.1 | n/a | Homo sapiens | n/a | 2020 | China |
| GCF-025159135.1 | Cecal | Ovis aries | n/a | 2020 | China |
| GCF-026202455.1 | Rectal swab | Sus domesticus | n/a | 2020 | China |
| GCF-026203285.1 | Rectal swab | Sus domesticus | n/a | 2020 | China |
| GCF-026203655.1 | Rectal swab | Sus domesticus | n/a | 2020 | China |
| GCF-025913595.1 | Pig | n/a | n/a | 2020 | China |
| GCF-025916235.1 | Pig | n/a | n/a | 2020 | China |
| GCF-025998395.1 | n/a | n/a | n/a | 2019 | China |
| GCF-026073115.1 | Sputum | Homo sapiens | n/a | 2021 | Belgium |
| GCF-026073415.1 | n/a | Homo sapiens | n/a | n/a | Belgium |
| GCF-026073435.1 | n/a | Homo sapiens | n/a | n/a | Belgium |
| GCF-026073455.1 | n/a | Homo sapiens | n/a | n/a | Belgium |
| GCF-026073625.1 | Urine | Homo sapiens | n/a | 2019 | Iran |
| GCF-026073735.1 | Urine | Homo sapiens | n/a | 2014 | Japan |
| GCF-026073915.1 | Urine | Homo sapiens | n/a | 2014 | Japan |
| GCF-026203855.1 | Rectal swab | Sus domesticus | n/a | 2020 | China:Shandong |
| GCF-026204135.1 | Rectal swab | Sus domesticus | n/a | 2015 | Denmark |
| GCF-026204185.1 | Rectal swab | Sus domesticus | n/a | 2015 | Denmark |
| GCF-004328225.1 | n/a | Vitis vinifera | n/a | 2012 | Netherlands |
| GCF-004328235.1 | n/a | Vitis vinifera | n/a | 2012 | Netherlands |
| GCF-004328255.1 | n/a | Vitis vinifera | n/a | 2012 | Croatia: Zagorje |
| GCF-026304695.1 | Blood | Homo sapiens | n/a | 2018 | South Africa: KwaZulu-Natal |
| GCF-026304705.1 | Urine | Homo sapiens | n/a | 2018 | South Africa: KwaZulu-Natal |
| GCF-026304715.1 | Urine | Homo sapiens | n/a | 2018 | China:wenzhou |
| GCF-026304775.1 | Urine | Homo sapiens | n/a | 2018 | China:wenzhou |
| GCF-026304855.1 | Ascites | Homo sapiens | n/a | 2019 | Belgium |
| GCF-026304865.1 | Puncture fluid | Homo sapiens | n/a | 2019 | Belgium |
| GCF-026304875.1 | Blood | Homo sapiens | n/a | 2019 | Belgium |
| GCF-026304885.1 | Urine | Homo sapiens | n/a | 2019 | Belgium |
| GCF-026304975.1 | Drainage | Homo sapiens | n/a | 2019 | Belgium |
| GCF-026305165.1 | Urine | Homo sapiens | n/a | 2019 | Belgium |
| GCF-026305175.1 | Urine | Homo sapiens | n/a | 2019 | Belgium |
| GCF-026305235.1 | Urine | Homo sapiens | n/a | 2019 | Belgium |
| GCF-026305275.1 | Blood | Homo sapiens | n/a | 2019 | Belgium |
| GCF-026305405.1 | Blood | Homo sapiens | n/a | 2019 | Belgium |
| GCF-026305475.1 | Ascites | Homo sapiens | n/a | 2019 | Belgium |
| GCF-026305515.1 | Secretion | Homo sapiens | n/a | 2021 | South Korea: Daegu |
| GCF-021228615.1 | Blood | Homo sapiens | Intraabdominal Abscess | 2021 | USA: Chicago |
| GCF-021311175.1 | Rectal swab | Homo sapiens | Asymptomatic intestinal carriage | 2019 | Reunion: Saint-Denis |
| GCF-021311345.1 | Rectal swab | Homo sapiens | Asymptomatic intestinal carriage | 2019 | Reunion: Saint-Denis |
| GCF-026308655.1 | Drainage | Homo sapiens | n/a | 2018 | China: Beijing |

| | | | | | |
|---|---|---|---|---|---|
| GCF-026308695.1 | Urine | Homo sapiens | n/a | 2016 | China:Wenzhou |
| GCF-026308785.1 | Urine | Homo sapiens | n/a | 2018 | Germany |
| GCF-026308825.1 | Urine | Homo sapiens | n/a | 2018 | Germany |
| GCF-026308855.1 | Urine | Homo sapiens | n/a | 2017 | China: Zhejiang |
| GCF-022462905.1 | Blood | Homo sapiens | Bacteremia | 2019 | Israel: Jerusalem |
| GCF-022462935.1 | Blood | Homo sapiens | Bacteremia | 2019 | Israel: Jerusalem |
| GCF-022462955.1 | Blood | Homo sapiens | Bacteremia | 2019 | Israel: Jerusalem |
| GCF-022462975.1 | Blood | Homo sapiens | Bacteremia | 2020 | Israel: Jerusalem |
| GCF-022462995.1 | Blood | Homo sapiens | Bacteremia | 2019 | Israel: Jerusalem |
| GCF-022463005.1 | Blood | Homo sapiens | Bacteremia | 2019 | Israel: Jerusalem |
| GCF-022463035.1 | Blood | Homo sapiens | Bacteremia | 2019 | Israel: Jerusalem |
| GCF-022463045.1 | Blood | Homo sapiens | Bacteremia | 2019 | Israel: Jerusalem |
| GCF-022463075.1 | Blood | Homo sapiens | Bacteremia | 2020 | Israel: Jerusalem |
| GCF-022463085.1 | Blood | Homo sapiens | Bacteremia | 2020 | Israel: Jerusalem |
| GCF-022463095.1 | Blood | Homo sapiens | Bacteremia | 2020 | Israel: Jerusalem |
| GCF-022463135.1 | Blood | Homo sapiens | Bacteremia | 2019 | Israel: Jerusalem |
| GCF-022463155.1 | Blood | Homo sapiens | Bacteremia | 2019 | Israel: Jerusalem |
| GCF-022463175.1 | Blood | Homo sapiens | Bacteremia | 2020 | Israel: Jerusalem |
| GCF-022463195.1 | Blood | Homo sapiens | Bacteremia | 2019 | Israel: Jerusalem |
| GCF-022463205.1 | Blood | Homo sapiens | Bacteremia | 2020 | Israel: Jerusalem |
| GCF-022463235.1 | Blood | Homo sapiens | Bacteremia | 2020 | Israel: Jerusalem |
| GCF-022463255.1 | Blood | Homo sapiens | Bacteremia | 2020 | Israel: Jerusalem |
| GCF-022463275.1 | Blood | Homo sapiens | Bacteremia | 2020 | Israel: Jerusalem |
| GCF-022463295.1 | Blood | Homo sapiens | Bacteremia | 2019 | Israel: Jerusalem |
| GCF-022463315.1 | Blood | Homo sapiens | Bacteremia | 2020 | Israel: Jerusalem |
| GCF-022463335.1 | Blood | Homo sapiens | Bacteremia | 2019 | Israel: Jerusalem |
| GCF-022463355.1 | Blood | Homo sapiens | Bacteremia | 2020 | Israel: Jerusalem |
| GCF-022463375.1 | Blood | Homo sapiens | Bacteremia | 2019 | Israel: Jerusalem |
| GCF-022463395.1 | Blood | Homo sapiens | Bacteremia | 2019 | Israel: Jerusalem |
| GCF-022463415.1 | Blood | Homo sapiens | Bacteremia | 2018 | Israel: Jerusalem |
| GCF-022463425.1 | Blood | Homo sapiens | Bacteremia | 2018 | Israel: Jerusalem |
| GCF-022463435.1 | Blood | Homo sapiens | Bacteremia | 2018 | Israel: Jerusalem |
| GCF-022463475.1 | Blood | Homo sapiens | Bacteremia | 2018 | Israel: Jerusalem |
| GCF-022463495.1 | Blood | Homo sapiens | Bacteremia | 2018 | Israel: Jerusalem |
| GCF-022463515.1 | Blood | Homo sapiens | Bacteremia | 2018 | Israel: Jerusalem |
| GCF-022463525.1 | Blood | Homo sapiens | Bacteremia | 2018 | Israel: Jerusalem |
| GCF-022463535.1 | Blood | Homo sapiens | Bacteremia | 2018 | Israel: Jerusalem |
| GCF-022463575.1 | Blood | Homo sapiens | Bacteremia | 2018 | Israel: Jerusalem |
| GCF-022463595.1 | Blood | Homo sapiens | Bacteremia | 2017 | Israel: Jerusalem |
| GCF-022463615.1 | Blood | Homo sapiens | Bacteremia | 2017 | Israel: Jerusalem |
| GCF-022463635.1 | Blood | Homo sapiens | Bacteremia | 2017 | Israel: Jerusalem |
| GCF-022463645.1 | Blood | Homo sapiens | Bacteremia | 2017 | Israel: Jerusalem |
| GCF-022463675.1 | Blood | Homo sapiens | Bacteremia | 2016 | Israel: Jerusalem |
| GCF-022463685.1 | Blood | Homo sapiens | Bacteremia | 2017 | Israel: Jerusalem |
| GCF-022463715.1 | Blood | Homo sapiens | Bacteremia | 2017 | Israel: Jerusalem |
| GCF-022463735.1 | Blood | Homo sapiens | Bacteremia | 2017 | Israel: Jerusalem |
| GCF-022463755.1 | Blood | Homo sapiens | Bacteremia | 2017 | Israel: Jerusalem |
| GCF-022463775.1 | Blood | Homo sapiens | Bacteremia | 2017 | Israel: Jerusalem |
| GCF-022463795.1 | Blood | Homo sapiens | Bacteremia | 2018 | Israel: Jerusalem |
| GCF-022463815.1 | Blood | Homo sapiens | Bacteremia | 2017 | Israel: Jerusalem |
| GCF-022463835.1 | Blood | Homo sapiens | Bacteremia | 2017 | Israel: Jerusalem |
| GCF-022463855.1 | Blood | Homo sapiens | Bacteremia | 2018 | Israel: Jerusalem |
| GCF-022463875.1 | Blood | Homo sapiens | Bacteremia | 2018 | Israel: Jerusalem |
| GCF-022463895.1 | Blood | Homo sapiens | Bacteremia | 2012 | Israel: Jerusalem |
| GCF-022463915.1 | Blood | Homo sapiens | Bacteremia | 2014 | Israel: Jerusalem |
| GCF-022463935.1 | Blood | Homo sapiens | Bacteremia | 2014 | Israel: Jerusalem |
| GCF-022463945.1 | Blood | Homo sapiens | Bacteremia | 2012 | Israel: Jerusalem |
| GCF-022463975.1 | Blood | Homo sapiens | Bacteremia | 2013 | Israel: Jerusalem |
| GCF-022463995.1 | Blood | Homo sapiens | Bacteremia | 2013 | Israel: Jerusalem |
| GCF-022464005.1 | Blood | Homo sapiens | Bacteremia | 2014 | Israel: Jerusalem |
| GCF-022464075.1 | Blood | Homo sapiens | Bacteremia | 2012 | Israel: Jerusalem |
| GCF-022464115.1 | Blood | Homo sapiens | Bacteremia | 2012 | Israel: Jerusalem |
| GCF-022464135.1 | Blood | Homo sapiens | Bacteremia | 2014 | Israel: Jerusalem |
| GCF-022464155.1 | Blood | Homo sapiens | Bacteremia | 2014 | Israel: Jerusalem |
| GCF-022464175.1 | Blood | Homo sapiens | Bacteremia | 2014 | Israel: Jerusalem |
| GCF-022464195.1 | Blood | Homo sapiens | Bacteremia | 2014 | Israel: Jerusalem |
| GCF-022464215.1 | Blood | Homo sapiens | Bacteremia | 2014 | Israel: Jerusalem |
| GCF-022464225.1 | Blood | Homo sapiens | Bacteremia | 2014 | Israel: Jerusalem |
| GCF-022464235.1 | Blood | Homo sapiens | Bacteremia | 2014 | Israel: Jerusalem |
| GCF-022464275.1 | Blood | Homo sapiens | Bacteremia | 2014 | Israel: Jerusalem |
| GCF-022464295.1 | Blood | Homo sapiens | Bacteremia | 2016 | Israel: Jerusalem |
| GCF-022464315.1 | Blood | Homo sapiens | Bacteremia | 2014 | Israel: Jerusalem |
| GCF-022464325.1 | Blood | Homo sapiens | Bacteremia | 2014 | Israel: Jerusalem |
| GCF-022464355.1 | Blood | Homo sapiens | Bacteremia | 2016 | Israel: Jerusalem |
| GCF-022464375.1 | Blood | Homo sapiens | Bacteremia | 2014 | Israel: Jerusalem |
| GCF-022464385.1 | Blood | Homo sapiens | Bacteremia | 2014 | Israel: Jerusalem |
| GCF-022464415.1 | Blood | Homo sapiens | Bacteremia | 2014 | Israel: Jerusalem |
| GCF-022464435.1 | Blood | Homo sapiens | Bacteremia | 2014 | Israel: Jerusalem |
| GCF-022464455.1 | Blood | Homo sapiens | Bacteremia | 2014 | Israel: Jerusalem |
| GCF-022464475.1 | Blood | Homo sapiens | Bacteremia | 2014 | Israel: Jerusalem |
| GCF-022464485.1 | Blood | Homo sapiens | Bacteremia | 2014 | Israel: Jerusalem |
| GCF-022464515.1 | Blood | Homo sapiens | Bacteremia | 2015 | Israel: Jerusalem |
| GCF-022464535.1 | Blood | Homo sapiens | Bacteremia | 2015 | Israel: Jerusalem |
| GCF-022464555.1 | Blood | Homo sapiens | Bacteremia | 2015 | Israel: Jerusalem |
| GCF-022464575.1 | Blood | Homo sapiens | Bacteremia | 2016 | Israel: Jerusalem |
| GCF-022464585.1 | Blood | Homo sapiens | Bacteremia | 2017 | Israel: Jerusalem |
| GCF-022464595.1 | Blood | Homo sapiens | Bacteremia | 2015 | Israel: Jerusalem |
| GCF-022464635.1 | Blood | Homo sapiens | Bacteremia | 2017 | Israel: Jerusalem |
| GCF-022464655.1 | Blood | Homo sapiens | Bacteremia | 2017 | Israel: Jerusalem |

| | | | | | |
|---|---|---|---|---|---|
| GCF-022464675.1 | Blood | Homo sapiens | Bacteremia | 2016 | Israel: Jerusalem |
| GCF-022464695.1 | Blood | Homo sapiens | Bacteremia | 2017 | Israel: Jerusalem |
| GCF-022464715.1 | Blood | Homo sapiens | Bacteremia | 2016 | Israel: Jerusalem |
| GCF-022464735.1 | Blood | Homo sapiens | Bacteremia | 2011 | Israel: Jerusalem |
| GCF-022464755.1 | Blood | Homo sapiens | Bacteremia | 2016 | Israel: Jerusalem |
| GCF-022464775.1 | Blood | Homo sapiens | Bacteremia | 2016 | Israel: Jerusalem |
| GCF-022464785.1 | Blood | Homo sapiens | Bacteremia | 2016 | Israel: Jerusalem |
| GCF-022464815.1 | Blood | Homo sapiens | Bacteremia | 2016 | Israel: Jerusalem |
| GCF-022464835.1 | Blood | Homo sapiens | Bacteremia | 2015 | Israel: Jerusalem |
| GCF-022464845.1 | Blood | Homo sapiens | Bacteremia | 2015 | Israel: Jerusalem |
| GCF-022464875.1 | Blood | Homo sapiens | Bacteremia | 2014 | Israel: Jerusalem |
| GCF-022464895.1 | Blood | Homo sapiens | Bacteremia | 2014 | Israel: Jerusalem |
| GCF-022464905.1 | Blood | Homo sapiens | Bacteremia | 2015 | Israel: Jerusalem |
| GCF-022464935.1 | Blood | Homo sapiens | Bacteremia | 2010 | Israel: Jerusalem |
| GCF-022464955.1 | Blood | Homo sapiens | Bacteremia | 2014 | Israel: Jerusalem |
| GCF-022464975.1 | Blood | Homo sapiens | Bacteremia | 2015 | Israel: Jerusalem |
| GCF-022465015.1 | Blood | Homo sapiens | Bacteremia | 2010 | Israel: Jerusalem |
| GCF-022467695.1 | Blood | Homo sapiens | Bacteremia | 2015 | Israel: Jerusalem |
| GCF-022467715.1 | Blood | Homo sapiens | Bacteremia | 2015 | Israel: Jerusalem |
| GCF-022467755.1 | Blood | Homo sapiens | Bacteremia | 2015 | Israel: Jerusalem |
| GCF-022467765.1 | Blood | Homo sapiens | Bacteremia | 2015 | Israel: Jerusalem |
| GCF-022467795.1 | Blood | Homo sapiens | Bacteremia | 2015 | Israel: Jerusalem |
| GCF-022467835.1 | Blood | Homo sapiens | Bacteremia | 2016 | Israel: Jerusalem |
| GCF-022467855.1 | Blood | Homo sapiens | Bacteremia | 2017 | Israel: Jerusalem |
| GCF-022467875.1 | Blood | Homo sapiens | Bacteremia | 2016 | Israel: Jerusalem |
| GCF-022467895.1 | Blood | Homo sapiens | Bacteremia | 2016 | Israel: Jerusalem |
| GCF-022467935.1 | Blood | Homo sapiens | Bacteremia | 2016 | Israel: Jerusalem |
| GCF-022467945.1 | Blood | Homo sapiens | Bacteremia | 2016 | Israel: Jerusalem |
| GCF-022467995.1 | Blood | Homo sapiens | Bacteremia | 2016 | Israel: Jerusalem |
| GCF-022468015.1 | Blood | Homo sapiens | Bacteremia | 2016 | Israel: Jerusalem |
| GCF-022468035.1 | Blood | Homo sapiens | Bacteremia | 2016 | Israel: Jerusalem |
| GCF-022468055.1 | Blood | Homo sapiens | Bacteremia | 2016 | Israel: Jerusalem |
| GCF-022468095.1 | Blood | Homo sapiens | Bacteremia | 2016 | Israel: Jerusalem |
| GCF-022468105.1 | Blood | Homo sapiens | Bacteremia | 2016 | Israel: Jerusalem |
| GCF-022468135.1 | Blood | Homo sapiens | Bacteremia | 2016 | Israel: Jerusalem |
| GCF-022468155.1 | Blood | Homo sapiens | Bacteremia | 2016 | Israel: Jerusalem |
| GCF-022468175.1 | Blood | Homo sapiens | Bacteremia | 2016 | Israel: Jerusalem |
| GCF-022468215.1 | Blood | Homo sapiens | Bacteremia | 2016 | Israel: Jerusalem |
| GCF-022468225.1 | Blood | Homo sapiens | Bacteremia | 2016 | Israel: Jerusalem |
| GCF-022468255.1 | Blood | Homo sapiens | Bacteremia | 2016 | Israel: Jerusalem |
| GCF-022468265.1 | Blood | Homo sapiens | Bacteremia | 2017 | Israel: Jerusalem |
| GCF-022468315.1 | Blood | Homo sapiens | Bacteremia | 2018 | Israel: Jerusalem |
| GCF-022468335.1 | Blood | Homo sapiens | Bacteremia | 2017 | Israel: Jerusalem |
| GCF-022468375.1 | Blood | Homo sapiens | Bacteremia | 2017 | Israel: Jerusalem |
| GCF-022468385.1 | Blood | Homo sapiens | Bacteremia | 2017 | Israel: Jerusalem |
| GCF-022468415.1 | Blood | Homo sapiens | Bacteremia | 2017 | Israel: Jerusalem |
| GCF-022468435.1 | Blood | Homo sapiens | Bacteremia | 2017 | Israel: Jerusalem |
| GCF-022468475.1 | Blood | Homo sapiens | Bacteremia | 2017 | Israel: Jerusalem |
| GCF-022468495.1 | Blood | Homo sapiens | Bacteremia | 2017 | Israel: Jerusalem |
| GCF-022468505.1 | Blood | Homo sapiens | Bacteremia | 2017 | Israel: Jerusalem |
| GCF-022468535.1 | Blood | Homo sapiens | Bacteremia | 2017 | Israel: Jerusalem |
| GCF-022468555.1 | Blood | Homo sapiens | Bacteremia | 2017 | Israel: Jerusalem |
| GCF-022468595.1 | Blood | Homo sapiens | Bacteremia | 2017 | Israel: Jerusalem |
| GCF-022468615.1 | Blood | Homo sapiens | Bacteremia | 2018 | Israel: Jerusalem |
| GCF-022468625.1 | Blood | Homo sapiens | Bacteremia | 2018 | Israel: Jerusalem |
| GCF-022468675.1 | Blood | Homo sapiens | Bacteremia | 2018 | Israel: Jerusalem |
| GCF-022468695.1 | Blood | Homo sapiens | Bacteremia | 2018 | Israel: Jerusalem |
| GCF-022468815.1 | Blood | Homo sapiens | Bacteremia | 2018 | Israel: Jerusalem |
| GCF-022468875.1 | Blood | Homo sapiens | Bacteremia | 2018 | Israel: Jerusalem |
| GCF-022468885.1 | Blood | Homo sapiens | Bacteremia | 2018 | Israel: Jerusalem |
| GCF-022468915.1 | Blood | Homo sapiens | Bacteremia | 2018 | Israel: Jerusalem |
| GCF-022468925.1 | Blood | Homo sapiens | Bacteremia | 2018 | Israel: Jerusalem |
| GCF-022468955.1 | Blood | Homo sapiens | Bacteremia | 2018 | Israel: Jerusalem |
| GCF-022469035.1 | Blood | Homo sapiens | Bacteremia | 2018 | Israel: Jerusalem |
| GCF-022469055.1 | Blood | Homo sapiens | Bacteremia | 2018 | Israel: Jerusalem |
| GCF-022469095.1 | Blood | Homo sapiens | Bacteremia | 2016 | Israel: Jerusalem |
| GCF-022469115.1 | Blood | Homo sapiens | Bacteremia | 2017 | Israel: Jerusalem |
| GCF-022469135.1 | Blood | Homo sapiens | Bacteremia | 2011 | Israel: Jerusalem |
| GCF-022469155.1 | Blood | Homo sapiens | Bacteremia | 2015 | Israel: Jerusalem |
| GCF-022469165.1 | Blood | Homo sapiens | Bacteremia | 2015 | Israel: Jerusalem |
| GCF-022469195.1 | Blood | Homo sapiens | Bacteremia | 2015 | Israel: Jerusalem |
| GCF-022469215.1 | Blood | Homo sapiens | Bacteremia | 2015 | Israel: Jerusalem |
| GCF-022469235.1 | Blood | Homo sapiens | Bacteremia | 2015 | Israel: Jerusalem |
| GCF-022469255.1 | Blood | Homo sapiens | Bacteremia | 2011 | Israel: Jerusalem |
| GCF-022469265.1 | Blood | Homo sapiens | Bacteremia | 2011 | Israel: Jerusalem |
| GCF-022469295.1 | Blood | Homo sapiens | Bacteremia | 2014 | Israel: Jerusalem |
| GCF-022469315.1 | Blood | Homo sapiens | Bacteremia | 2015 | Israel: Jerusalem |
| GCF-022469335.1 | Blood | Homo sapiens | Bacteremia | 2014 | Israel: Jerusalem |
| GCF-022469355.1 | Blood | Homo sapiens | Bacteremia | 2014 | Israel: Jerusalem |
| GCF-022469375.1 | Blood | Homo sapiens | Bacteremia | 2010 | Israel: Jerusalem |
| GCF-022469395.1 | Blood | Homo sapiens | Bacteremia | 2014 | Israel: Jerusalem |
| GCF-022469415.1 | Blood | Homo sapiens | Bacteremia | 2014 | Israel: Jerusalem |
| GCF-022469435.1 | Blood | Homo sapiens | Bacteremia | 2014 | Israel: Jerusalem |
| GCF-022469455.1 | Blood | Homo sapiens | Bacteremia | 2014 | Israel: Jerusalem |
| GCF-022469465.1 | Blood | Homo sapiens | Bacteremia | 2013 | Israel: Jerusalem |
| GCF-022469495.1 | Blood | Homo sapiens | Bacteremia | 2013 | Israel: Jerusalem |
| GCF-022469515.1 | Blood | Homo sapiens | Bacteremia | 2012 | Israel: Jerusalem |
| GCF-022469525.1 | Blood | Homo sapiens | Bacteremia | 2012 | Israel: Jerusalem |
| GCF-022469555.1 | Blood | Homo sapiens | Bacteremia | 2010 | Israel: Jerusalem |

| | | | | | |
|---|---|---|---|---|---|
| GCF-022469575.1 | Blood | Homo sapiens | Bacteremia | 2010 | Israel: Jerusalem |
| GCF-026308905.1 | Urine | Homo sapiens | n/a | 2018 | Pakistan: Islamabad |
| GCF-026308935.1 | Urine | Homo sapiens | n/a | 2018 | Pakistan: Islamabad |
| GCF-026308975.1 | Secretion | Homo sapiens | n/a | 2018 | Pakistan: Islamabad |
| GCF-026308995.1 | Urine | Homo sapiens | n/a | 2019 | USA:Little Rock |
| GCF-026309035.1 | Urine | Homo sapiens | n/a | 2019 | USA:Little Rock |
| GCF-026309055.1 | Urine | Homo sapiens | n/a | 2019 | USA:Little Rock |
| GCF-026309075.1 | Urine | Homo sapiens | n/a | 2004 | USA |
| GCF-026309095.1 | Urine | Homo sapiens | n/a | 2004 | USA |
| GCF-022699225.1 | Blood | Homo sapiens | Chronic renal insufficiency | 2018 | China: BeiJing |
| GCF-022699545.1 | Urine | Homo sapiens | Lumbar vertebrae fracture | 2020 | China: Shandong |
| GCF-022699565.1 | Sanies | Homo sapiens | Post operation | 2020 | China: Shandong |
| GCF-026309135.1 | Urine | Homo sapiens | n/a | n/a | n/a |
| GCF-026309175.1 | Urine | Homo sapiens | n/a | 2018 | China: Xinjiang |
| GCF-026309215.1 | Bile | Homo sapiens | n/a | 2018 | China: Xinjiang |
| GCF-023204955.1 | Blood | Homo sapiens | Infection | 2019 | USA |
| GCF-023204975.1 | Blood | Homo sapiens | Infection | 2019 | USA |
| GCF-026309245.1 | Ascites | Homo sapiens | n/a | 2022 | n/a |
| GCF-026427575.1 | Sludge | n/a | n/a | 2022 | n/a |
| GCF-026921135.1 | Stool | Homo sapiens | n/a | 2022 | n/a |
| GCF-026921235.1 | Blood | Homo sapiens | n/a | 2022 | n/a |
| GCF-026921615.1 | Stool | Homo sapiens | n/a | 2021 | n/a |
| GCF-026921695.1 | Rectal swab | Homo sapiens | n/a | 2021 | n/a |
| GCF-026923195.1 | Catheter | Homo sapiens | n/a | 2021 | n/a |
| GCF-026928435.1 | Blood | Homo sapiens | n/a | 2021 | n/a |
| GCF-026928445.1 | Rectal swab | Homo sapiens | n/a | 2021 | n/a |
| GCF-026928645.1 | Tissue | Homo sapiens | n/a | 2021 | n/a |
| GCF-026928745.1 | Clinical isolate | Homo sapiens | n/a | 2021 | n/a |
| GCF-026928915.1 | Wound | Homo sapiens | n/a | 2021 | n/a |
| GCF-026928925.1 | Blood | Homo sapiens | n/a | 2017 | USA:New York |
| GCF-026929095.1 | Clinical isolate | Homo sapiens | n/a | 2018 | China: Jinan |
| GCF-026929115.1 | Clinical isolate | Homo sapiens | n/a | 2019 | Greece: Epirus |
| GCF-026929155.1 | Clinical isolate | Homo sapiens | n/a | 2019 | Greece: Epirus |
| GCF-026929245.1 | Clinical isolate | Homo sapiens | n/a | 2019 | Greece: Epirus |
| GCF-026929355.1 | n/a | Homo sapiens | n/a | 2019 | Greece: Western Macedonia |
| GCF-026929395.1 | n/a | Homo sapiens | n/a | 2019 | Greece: Epirus |
| GCF-026929415.1 | n/a | Homo sapiens | n/a | 2019 | Greece: Epirus |
| GCF-026929425.1 | n/a | Homo sapiens | n/a | 2019 | Greece: Western Macedonia |
| GCF-026929575.1 | n/a | Homo sapiens | n/a | 2019 | Greece: Western Macedonia |
| GCF-026929595.1 | n/a | Homo sapiens | n/a | 2019 | Greece: Western Macedonia |
| GCF-026929615.1 | n/a | Homo sapiens | n/a | 2019 | Greece: Western Macedonia |
| GCF-026929655.1 | n/a | Homo sapiens | n/a | 2019 | Greece: Western Macedonia |
| GCF-026934385.1 | n/a | Homo sapiens | n/a | 2019 | Greece: Western Macedonia |
| GCF-026934425.1 | n/a | Homo sapiens | n/a | 2019 | Greece: Western Macedonia |
| GCF-026934465.1 | n/a | Homo sapiens | n/a | 2019 | Greece: Epirus |
| GCF-026934555.1 | n/a | Homo sapiens | n/a | 2019 | Greece: Epirus |
| GCF-026934765.1 | Rectal swab | Homo sapiens | n/a | 2019 | Greece: Western Macedonia |
| GCF-026934785.1 | Stool | Homo sapiens | n/a | 2019 | Greece: Western Macedonia |
| GCF-026934795.1 | Clinical isolate | Homo sapiens | n/a | 2019 | Greece: Western Macedonia |
| GCF-026934965.1 | Rectal swab | Homo sapiens | n/a | 2019 | Greece: Western Macedonia |
| GCF-026941305.1 | Rectal swab | Homo sapiens | n/a | 2019 | Greece: Epirus |
| GCF-026941325.1 | Stool | Homo sapiens | n/a | 2019 | Greece: Epirus |
| GCF-026941345.1 | Stool | Homo sapiens | n/a | 2019 | Greece: Western Macedonia |
| GCF-026941365.1 | Stool | Homo sapiens | n/a | 2019 | Greece: Epirus |
| GCF-026941385.1 | Stool | Homo sapiens | n/a | 2019 | Greece: Western Macedonia |
| GCF-026941405.1 | Rectal swab | Homo sapiens | n/a | 2016 | Mexico:Sinaloa |
| GCF-026941435.1 | Stool | Homo sapiens | n/a | n/a | n/a |
| GCF-026941595.1 | Clinical isolate | Homo sapiens | n/a | 2021 | Turkey: Ankara |
| GCF-025399665.1 | Mastitis milk | Holstein Friesian cross breed lactating cow | Mastitis | 2021 | Bangladesh |
| GCF-025599075.1 | Pus | Homo sapiens | Infection | 2020 | India: Puducherry |
| GCF-026941725.1 | Rectal swab | Homo sapiens | n/a | 2020 | China: Jiangsu |
| GCF-026941865.1 | Stool | Homo sapiens | n/a | 2020 | China: Jiangsu |
| GCF-026942195.1 | Stool | Homo sapiens | n/a | 2020 | China: Jiangsu |
| GCF-026942235.1 | Stool | Homo sapiens | n/a | 2020 | China: Jiangsu |
| GCF-026942285.1 | Stool | Homo sapiens | n/a | 2020 | China: Jiangsu |
| GCF-026942315.1 | Rectal swab | Homo sapiens | n/a | 2021 | Viet Nam:Hanoi |
| GCF-026942325.1 | Pus | Homo sapiens | n/a | 2000 | Japan |
| GCF-026942375.1 | Clinical isolate | Homo sapiens | n/a | n/a | Japan |
| GCF-026942385.1 | Urine | Homo sapiens | n/a | 2010 | Japan |
| GCF-026942685.1 | Urine | Homo sapiens | n/a | 2011 | Japan |
| GCF-026942985.1 | Urine | Homo sapiens | n/a | 2014 | Japan |
| GCF-026943305.1 | Prothesis | Homo sapiens | n/a | 2015 | Japan |
| GCF-026943475.1 | Rectal swab | Homo sapiens | n/a | 2015 | Japan |
| GCF-026943485.1 | Urine | Homo sapiens | n/a | 2021 | China |
| GCF-026943525.1 | Clinical isolate | Homo sapiens | n/a | 2021 | China |
| GCF-026943555.1 | Rectal swab | Homo sapiens | n/a | 2021 | China |
| GCF-026943565.1 | Rectal swab | Homo sapiens | n/a | 2021 | China |
| GCF-026943595.1 | Rectal swab | Homo sapiens | n/a | 2021 | China |
| GCF-026943805.1 | Clinical isolate | Homo sapiens | n/a | 2021 | China |
| GCF-026943895.1 | Clinical isolate | Homo sapiens | n/a | 2016 | China:Liaoning province |
| GCF-026943945.1 | Rectal swab | Homo sapiens | n/a | 2016 | China:Liaoning province |
| GCF-026944055.1 | Rectal swab | Homo sapiens | n/a | 2016 | China:Liaoning province |
| GCF-026944215.1 | Rectal swab | Homo sapiens | n/a | 2016 | China:Liaoning province |
| GCF-026944365.1 | Rectal swab | Homo sapiens | n/a | 2016 | China:Liaoning province |
| GCF-026944485.1 | Rectal swab | Homo sapiens | n/a | 2016 | China:Liaoning province |
| GCF-026944565.1 | Rectal swab | Homo sapiens | n/a | 2016 | China:Liaoning province |
| GCF-026944605.1 | Rectal swab | Homo sapiens | n/a | 2016 | China:Liaoning province |
| GCF-026944625.1 | Rectal swab | Homo sapiens | n/a | 2016 | China:Liaoning province |
| GCF-026944645.1 | Rectal swab | Homo sapiens | n/a | 2015 | China:Liaoning province |

| | | | | | |
|---|---|---|---|---|---|
| GCF-026944655.1 | Stool | Homo sapiens | n/a | 2015 | China:Liaoning province |
| GCF-026944695.1 | Rectal swab | Homo sapiens | n/a | 2015 | China:Liaoning province |
| GCF-026944725.1 | Rectal swab | Homo sapiens | n/a | 2014 | China:Liaoning province |
| GCF-026944745.1 | Stool | Homo sapiens | n/a | 2014 | China:Liaoning province |
| GCF-026944785.1 | Rectal swab | Homo sapiens | n/a | 2014 | China:Liaoning province |
| GCF-026944805.1 | Rectal swab | Homo sapiens | n/a | 2014 | China:Liaoning province |
| GCF-026944945.1 | Rectal swab | Homo sapiens | n/a | 2014 | China:Liaoning province |
| GCF-026945025.1 | Rectal swab | Homo sapiens | n/a | 2014 | China:Liaoning province |
| GCF-026945035.1 | Rectal swab | Homo sapiens | n/a | 2014 | China:Liaoning province |
| GCF-026945125.1 | Rectal swab | Homo sapiens | n/a | 2014 | China:Liaoning province |
| GCF-026945225.1 | Blood | Homo sapiens | n/a | 2014 | China:Liaoning province |
| GCF-026945465.1 | Urine | Homo sapiens | n/a | 2013 | China:Liaoning province |
| GCF-026945505.1 | Blood | Homo sapiens | n/a | 2013 | China:Liaoning province |
| GCF-026945565.1 | Rectal swab | Homo sapiens | n/a | 2013 | China:Liaoning province |
| GCF-026945625.1 | Rectal swab | Homo sapiens | n/a | 2013 | China:Liaoning province |
| GCF-026945635.1 | Rectal swab | Homo sapiens | n/a | 2013 | China:Liaoning province |
| GCF-026945745.1 | Rectal swab | Homo sapiens | n/a | 2013 | China:Liaoning province |
| GCF-026945785.1 | Rectal swab | Homo sapiens | n/a | 2013 | China:Liaoning province |
| GCF-026945825.1 | Rectal swab | Homo sapiens | n/a | 2013 | China:Liaoning province |
| GCF-026945885.1 | Rectal swab | Homo sapiens | n/a | 2013 | China:Liaoning province |
| GCF-026945925.1 | Rectal swab | Homo sapiens | n/a | 2012 | China:Liaoning province |
| GCF-026946005.1 | Rectal swab | Homo sapiens | n/a | 2012 | China:Liaoning province |
| GCF-026946065.1 | Rectal swab | Homo sapiens | n/a | 2012 | China:Liaoning province |
| GCF-026409165.1 | Clinical isolate | Homo sapiens | Clinical isolate | 2019 | USA |
| GCF-026946145.1 | Rectal swab | Homo sapiens | n/a | 2021 | China:Jiangsu |
| GCF-026921075.1 | Urine | Homo sapiens | Urinary tract infection | 2017 | Belgium: Brussels |
| GCF-026946185.1 | Rectal swab | Homo sapiens | n/a | 2017 | Belgium: Bouge |
| GCF-026946195.1 | Rectal swab | Homo sapiens | n/a | 2012 | Belgium: Leuven |
| GCF-026946325.1 | Rectal swab | Homo sapiens | n/a | 2017 | Belgium: Sint-Niklaas |
| GCF-026946365.1 | Rectal swab | Homo sapiens | n/a | 2018 | Belgium: La Louviere |
| GCF-026946385.1 | Rectal swab | Homo sapiens | n/a | 2014 | Belgium: Leuven |
| GCF-026922155.1 | Peritoneal | Homo sapiens | Peritonitis | 2013 | Belgium: La Louviere |
| GCF-026922255.1 | Clinical isolate | Homo sapiens | Pneumonia | 2014 | Belgium: Edegem |
| GCF-026946485.1 | Rectal swab | Homo sapiens | n/a | 2013 | Belgium: Montignies-Le-Tilleul |
| GCF-026922355.1 | Peritoneal | Homo sapiens | Peritonitis | 2013 | Belgium: La Louviere |
| GCF-026946645.1 | Rectal swab | Homo sapiens | n/a | 2016 | Belgium: Edegem |
| GCF-026946665.1 | Clinical isolate | Homo sapiens | n/a | 2014 | Belgium: Edegem |
| GCF-026946745.1 | Rectal swab | Homo sapiens | n/a | 2017 | Belgium: Roeselare |
| GCF-026946805.1 | Clinical isolate | Homo sapiens | n/a | 2016 | Belgium: Gilly |
| GCF-026946825.1 | Clinical isolate | Homo sapiens | n/a | 2016 | Belgium: Leuven |
| GCF-026946905.1 | Tissue | Homo sapiens | n/a | 2016 | Belgium: Gilly |
| GCF-026947065.1 | Stool | Homo sapiens | n/a | 2016 | Belgium: Gilly |
| GCF-026947085.1 | Rectal swab | Homo sapiens | n/a | 2016 | Belgium: Tournai |
| GCF-026947165.1 | Rectal swab | Homo sapiens | n/a | 2015 | Belgium: Gilly |
| GCF-026947205.1 | Rectal swab | Homo sapiens | n/a | 2015 | Belgium: Godinne |
| GCF-026947345.1 | Blood | Homo sapiens | n/a | 2015 | Belgium: Antwerp |
| GCF-026947465.1 | Blood | Homo sapiens | n/a | 2015 | Belgium: Gilly |
| GCF-026947615.1 | Stool | Homo sapiens | n/a | 2015 | Belgium: Gilly |
| GCF-026947645.1 | Stool | Homo sapiens | n/a | 1993 | USA: Iowa |
| GCF-026947705.1 | Stool | Homo sapiens | n/a | 2012 | Denmark: Copenhagen |
| GCF-026947865.1 | Clinical isolate | Homo sapiens | n/a | 2013 | Denmark: Copenhagen |
| GCF-026947945.1 | Stool | Homo sapiens | n/a | 2010 | Denmark: Copenhagen |
| GCF-026948025.1 | Clinical isolate | Homo sapiens | n/a | 2019 | Belgium: Wilrijk |
| GCF-026948065.1 | Clinical isolate | Homo sapiens | n/a | 2019 | Belgium: Wilrijk |
| GCF-026948075.1 | Rectal swab | Homo sapiens | n/a | 2019 | Belgium: Wilrijk |
| GCF-026948585.1 | Wound | Homo sapiens | n/a | 2019 | Belgium: Wilrijk |
| GCF-026948655.1 | Stool | Homo sapiens | n/a | 2019 | Belgium: Wilrijk |
| GCF-026948745.1 | Clinical isolate | Homo sapiens | n/a | 2019 | Belgium: Wilrijk |
| GCF-026948845.1 | Clinical isolate | Homo sapiens | n/a | 2019 | Belgium: Wilrijk |
| GCF-026948855.1 | Clinical isolate | Homo sapiens | n/a | 2019 | Belgium: Wilrijk |
| GCF-026948875.1 | Wound | Homo sapiens | n/a | 2019 | Belgium: Wilrijk |
| GCF-026949005.1 | Wound | Homo sapiens | n/a | 2019 | Belgium: Wilrijk |
| GCF-026949025.1 | Clinical isolate | Homo sapiens | n/a | 2019 | Belgium: Wilrijk |
| GCF-026949055.1 | Urine | Homo sapiens | n/a | 2019 | Belgium: Wilrijk |
| GCF-026949185.1 | Catheter | Homo sapiens | n/a | 2005 | Belgium: Gilly |
| GCF-026949205.1 | Rectal swab | Homo sapiens | n/a | 2017 | Belgium: Roeselare |
| GCF-026949225.1 | Clinical isolate | Homo sapiens | n/a | 2018 | Belgium: Leuven |
| GCF-026949305.1 | Urine | Homo sapiens | n/a | 2010 | Denmark: Copenhagen |
| GCF-026949405.1 | Blood | Homo sapiens | n/a | 2017 | Belgium: Leuven |
| GCF-026949525.1 | Blood | Homo sapiens | n/a | 2017 | Belgium: Godinne |
| GCF-026949565.1 | Blood | Homo sapiens | n/a | 2017 | Belgium: Bouge |
| GCF-026949605.1 | Blood | Homo sapiens | n/a | 2017 | Belgium: Bouge |
| GCF-026949705.1 | Expectoration | Homo sapiens | n/a | 2017 | Belgium: Veurne |
| GCF-026949725.1 | Rectal swab | Homo sapiens | n/a | 2017 | Belgium: Brussels |
| GCF-026949785.1 | Wound | Homo sapiens | n/a | 2017 | Belgium: Brussels |
| GCF-026949845.1 | Rectal swab | Homo sapiens | n/a | 2017 | Belgium: Brussels |
| GCF-026949925.1 | Rectal swab | Homo sapiens | n/a | 2017 | Belgium: Leuven |
| GCF-026950005.1 | Rectal swab | Homo sapiens | n/a | 2017 | Belgium: Leuven |
| GCF-026950015.1 | Rectal swab | Homo sapiens | n/a | 2017 | Belgium: Bouge |
| GCF-026950105.1 | Rectal swab | Homo sapiens | n/a | 2017 | Belgium: Bouge |
| GCF-026950145.1 | Clinical isolate | Homo sapiens | n/a | 2017 | Belgium: Bouge |
| GCF-026950225.1 | Rectal swab | Homo sapiens | n/a | 2017 | Belgium: Veurne |
| GCF-026950285.1 | Rectal swab | Homo sapiens | n/a | 2017 | Belgium: Brussels |
| GCF-026950325.1 | Rectal swab | Homo sapiens | n/a | 2017 | Belgium: Leuven |
| GCF-026950365.1 | Rectal swab | Homo sapiens | n/a | 2018 | Belgium: Liege |
| GCF-026950445.1 | Rectal swab | Homo sapiens | n/a | 2017 | Belgium: Liege |
| GCF-026950515.1 | Stool | Homo sapiens | n/a | 2017 | Belgium: Liege |
| GCF-026950655.1 | Rectal swab | Homo sapiens | n/a | 2018 | Belgium: Liege |
| GCF-026950705.1 | Blood | Homo sapiens | n/a | 2018 | Belgium: Liege |

| | | | | | |
|---|---|---|---|---|---|
| GCF-026942915.1 | Urine | Homo sapiens | Urinary tract infection | 2018 | Belgium: Liege |
| GCF-026950825.1 | Rectal swab | Homo sapiens | n/a | 2017 | Belgium: Liege |
| GCF-026943225.1 | Fistula | Homo sapiens | Peritonitis | 2017 | Belgium: Liege |
| GCF-026950835.1 | Rectal swab | Homo sapiens | n/a | 2018 | Belgium: Liege |
| GCF-026950965.1 | Stool | Homo sapiens | n/a | 2018 | Belgium: Waregem |
| GCF-026951005.1 | Rectal swab | Homo sapiens | n/a | 2018 | Belgium: Liege |
| GCF-026951145.1 | Stool | Homo sapiens | n/a | 2018 | Belgium: Boussu |
| GCF-026951165.1 | Clinical isolate | Homo sapiens | n/a | 2018 | Belgium: Verviers |
| GCF-026951215.1 | Stool | Homo sapiens | n/a | 2017 | Belgium: Brussels |
| GCF-026951305.1 | Tracheal aspirate | Homo sapiens | n/a | 2018 | Belgium: Leuven |
| GCF-026951315.1 | Stool | Homo sapiens | n/a | 2017 | Belgium: Brussels |
| GCF-026951405.1 | Rectal swab | Homo sapiens | n/a | 2017 | Belgium: Brussels |
| GCF-026951465.1 | Rectal swab | Homo sapiens | n/a | 2018 | Belgium: Waregem |
| GCF-026951485.1 | Stool | Homo sapiens | n/a | 2018 | Belgium: Boussu |
| GCF-026951605.1 | Stool | Homo sapiens | n/a | 2018 | Belgium: Waregem |
| GCF-026951665.1 | Stool | Homo sapiens | n/a | 2018 | Belgium: Waregem |
| GCF-026951705.1 | Stool | Homo sapiens | n/a | 2018 | Belgium: Leuven |
| GCF-026951985.1 | Stool | Homo sapiens | n/a | 2018 | Belgium: Waregem |
| GCF-026952025.1 | Clinical isolate | Homo sapiens | n/a | 2018 | Belgium: Waregem |
| GCF-026952165.1 | Wound | Homo sapiens | n/a | 2018 | Belgium: Waregem |
| GCF-026952175.1 | Blood | Homo sapiens | n/a | 2018 | Belgium: Waregem |
| GCF-026952225.1 | Urine | Homo sapiens | n/a | 2018 | Belgium: Waregem |
| GCF-026952485.1 | Blood | Homo sapiens | n/a | 2017 | Belgium: Ghent |
| GCF-026952585.1 | Blood | Homo sapiens | n/a | 2017 | Belgium: Brussels |
| GCF-026952595.1 | Blood | Homo sapiens | n/a | 2017 | Belgium: Roeselare |
| GCF-026952765.1 | Clinical isolate | Homo sapiens | n/a | 2017 | Belgium: Roeselare |
| GCF-026952785.1 | Rectal swab | Homo sapiens | n/a | 2017 | Belgium: Ghent |
| GCF-026952885.1 | Urine | Homo sapiens | n/a | 2017 | Belgium: Godinne |
| GCF-026952925.1 | Clinical isolate | Homo sapiens | n/a | 2017 | Belgium: Godinne |
| GCF-026953005.1 | Clinical isolate | Homo sapiens | n/a | 2017 | Belgium: Godinne |
| GCF-026953065.1 | Blood | Homo sapiens | n/a | 2017 | Belgium: Godinne |
| GCF-026953085.1 | Clinical isolate | Homo sapiens | n/a | 2017 | Belgium: Godinne |
| GCF-026953105.1 | Wound | Homo sapiens | n/a | 2017 | Belgium: Godinne |
| GCF-026953165.1 | Rectal swab | Homo sapiens | n/a | 2012 | Belgium: Gilly |
| GCF-026953205.1 | Skin | Homo sapiens | n/a | 2011 | Belgium: Leuven |
| GCF-026953225.1 | Tissue | Homo sapiens | n/a | 2010 | Belgium: Leuven |
| GCF-026953455.1 | Clinical isolate | Homo sapiens | n/a | 2009 | Belgium: Montignies-Le-Tilleul |
| GCF-026953495.1 | Clinical isolate | Homo sapiens | n/a | 2018 | Belgium: Seraing |
| GCF-026953615.1 | n/a | Homo sapiens | n/a | 2009 | Belgium: Antwerpen |
| GCF-026953675.1 | n/a | Homo sapiens | n/a | 2018 | Belgium: Seraing |
| GCF-026953775.1 | n/a | Homo sapiens | n/a | 2018 | Belgium: Seraing |
| GCF-026953855.1 | n/a | Homo sapiens | n/a | 2018 | Belgium: Seraing |
| GCF-026953895.1 | n/a | Homo sapiens | n/a | 2018 | Belgium: Seraing |
| GCF-026953915.1 | n/a | Homo sapiens | n/a | 2018 | Belgium: Seraing |
| GCF-027662275.1 | n/a | Homo sapiens | n/a | 2018 | Belgium: Seraing |
| GCF-027672285.1 | n/a | Homo sapiens | n/a | 2018 | Belgium: Seraing |
| GCF-027674745.1 | n/a | Homo sapiens | n/a | 2018 | Belgium: Liege |
| GCF-027677565.1 | n/a | Homo sapiens | n/a | 2018 | Belgium: Liege |
| GCF-027677885.1 | n/a | Homo sapiens | n/a | 2018 | Belgium: Liege |
| GCF-026946085.1 | Urine | Homo sapiens | Urinary tract infection | 2018 | Belgium: Liege |
| GCF-027678045.1 | n/a | Homo sapiens | n/a | 2018 | Belgium: Liege |
| GCF-027678105.1 | n/a | Homo sapiens | n/a | 2018 | Belgium: Liege |
| GCF-027678185.1 | n/a | Homo sapiens | n/a | 2018 | Belgium: Liege |
| GCF-027678305.1 | n/a | Homo sapiens | n/a | 2018 | Belgium: Mons |
| GCF-027678325.1 | n/a | Homo sapiens | n/a | 2018 | Belgium: Mons |
| GCF-027678505.1 | n/a | Homo sapiens | n/a | 2018 | Belgium: Mons |
| GCF-027678795.1 | n/a | Homo sapiens | n/a | 2018 | Belgium: Mons |
| GCF-027679065.1 | n/a | Homo sapiens | n/a | 2018 | Belgium: Waregem |
| GCF-027679265.1 | n/a | Homo sapiens | n/a | 2018 | Belgium: Waregem |
| GCF-027679605.1 | n/a | Homo sapiens | n/a | 2018 | Belgium: Waregem |
| GCF-027689725.1 | n/a | Homo sapiens | n/a | 2018 | Belgium: Waregem |
| GCF-027915235.1 | Meju, fermented soybean | n/a | n/a | 2018 | Belgium: Liege |
| GCF-027944535.1 | Sewage | n/a | n/a | 2018 | Belgium: Leuven |
| GCF-026946945.1 | Urine | Homo sapiens | Urinary tract infection | 2017 | Belgium: Tessenderlo |
| GCF-026947025.1 | Urine | Homo sapiens | Urosepsis | 2017 | Belgium: Mol |
| GCF-028335205.1 | Stool | Bos taurus | n/a | 2017 | Belgium: Overpelt |
| GCF-028335225.1 | Stool | Bos taurus | n/a | 2017 | Belgium: Turnhout |
| GCF-028335245.1 | Stool | Bos taurus | n/a | 2017 | Belgium: Tongeren |
| GCF-028335265.1 | Stool | Bos taurus | n/a | 2017 | Belgium: Tongeren |
| GCF-026947245.1 | Urine | Homo sapiens | Urinary tract infection | 2017 | Belgium: Tongeren |
| GCF-026947305.1 | Urine | Homo sapiens | Urinary tract infection | 2017 | Belgium: Tongeren |
| GCF-028335465.1 | Stool | Bos taurus | n/a | 2004 | Belgium: Brussels |
| GCF-028335485.1 | Stool | Bos taurus | n/a | 2003 | Belgium: Ieper |
| GCF-028335525.1 | Stool | Bos taurus | n/a | 2016 | Belgium: Namur |
| GCF-028335565.1 | Stool | Bos taurus | n/a | 2016 | Belgium: Brussels |
| GCF-028335585.1 | Stool | Bos taurus | n/a | 2015 | Belgium: Gilly |
| GCF-028335605.1 | Stool | Bos taurus | n/a | 2017 | Belgium: Eeklo |
| GCF-028335625.1 | Stool | Bos taurus | n/a | 2017 | Belgium: Eeklo |
| GCF-004328385.1 | n/a | Vitis vinifera | n/a | 2017 | Belgium: Eeklo |
| GCF-004332255.1 | n/a | Vitis vinifera | n/a | 2012 | Belgium: Edegem |
| GCF-028551565.1 | Stool | Bos taurus | n/a | 2016 | Belgium: Brussels |
| GCF-028891525.1 | Sewage | n/a | n/a | 2017 | Belgium: Eeklo |
| GCF-900044005.1 | n/a | n/a | n/a | 2016 | Belgium: Brussels |
| GCF-900066025.1 | Stool | n/a | n/a | 2016 | Belgium: Brussels |
| GCF-900092475.1 | Clinical isolate | Homo sapiens | n/a | 2016 | Belgium: Leuven |
| GCF-900094185.1 | Human | Homo sapiens | n/a | 2015 | Belgium: Boussu |
| GCF-900143335.1 | Stool | n/a | n/a | 2015 | Belgium: Mouscron |
| GCF-026948485.1 | Blood | Homo sapiens | Peritonitis | 2017 | Belgium: Brussels |
| GCF-900143345.1 | Stool | n/a | n/a | 2011 | Belgium: Edegem |

| | | | | | |
|---|---|---|---|---|---|
| GCF-900143355.1 | Stool | n/a | n/a | 2017 | Belgium: Leuven |
| GCF-900143385.1 | Stool | n/a | n/a | 2010 | Belgium: Edegem |
| GCF-900143455.1 | Stool | n/a | n/a | 2017 | Belgium: Overpelt |
| GCF-900148565.1 | Stool | n/a | n/a | 2017 | Belgium: Overpelt |
| GCF-900148595.1 | Stool | n/a | n/a | 2017 | Belgium: Overpelt |
| GCF-900148655.1 | Stool | n/a | n/a | 2017 | Belgium: Overpelt |
| GCF-900148665.1 | Stool | n/a | n/a | 2016 | Belgium: Overpelt |
| GCF-900148705.1 | Stool | n/a | n/a | 2016 | Belgium: Overpelt |
| GCF-900148725.1 | Stool | n/a | n/a | 2016 | Belgium: Overpelt |
| GCF-900178555.1 | Human | n/a | n/a | 2016 | Belgium: Overpelt |
| GCF-900178565.1 | Human | n/a | n/a | 2014 | Belgium: Ronse |
| GCF-900178575.1 | Human | n/a | n/a | 2016 | Belgium: Leuven |
| GCF-900178585.1 | Human | n/a | n/a | 2014 | Belgium: Leuven |
| GCF-900178595.1 | Human | n/a | n/a | 2013 | Belgium: Huy |
| GCF-900178605.1 | Human | n/a | n/a | 2011 | Belgium: Leuven |
| GCF-900178615.1 | Human | n/a | n/a | 2012 | Belgium: Liege |
| GCF-900178625.1 | Human | n/a | n/a | 2012 | Belgium: Gosselies |
| GCF-900178635.1 | Human | n/a | n/a | 2011 | Belgium: Mons |
| GCF-900178645.1 | Human | n/a | n/a | 2009 | Belgium: Edegem |
| GCF-900178655.1 | Human | n/a | n/a | 2008 | Belgium: Huy |
| GCF-900178665.1 | Human | n/a | n/a | 2018 | Belgium: Brussels |
| GCF-900178675.1 | Human | n/a | n/a | 2018 | Belgium: Waregem |
| GCF-900178685.1 | Human | n/a | n/a | 2017 | Belgium: Waregem |
| GCF-900178695.1 | Human | n/a | n/a | 2019 | Belgium: Sint-Niklaas |
| GCF-026949865.1 | Urine | Homo sapiens | Urinary tract infection | 2017 | Belgium: Verviers |
| GCF-900178705.1 | Human | n/a | n/a | 2017 | Belgium: Namur |
| GCF-900178715.1 | Human | n/a | n/a | 2017 | Belgium: Tienen |
| GCF-900178725.1 | Human | n/a | n/a | 2017 | Belgium: Tienen |
| GCF-900178735.1 | Human | n/a | n/a | 2017 | Belgium: Tienen |
| GCF-900178745.1 | Human | n/a | n/a | 2017 | Belgium: Tienen |
| GCF-900178755.1 | Human | n/a | n/a | 2017 | Belgium: Sint-Niklaas |
| GCF-900178765.1 | Human | n/a | n/a | 2017 | Belgium: Tienen |
| GCF-900178775.1 | Human | n/a | n/a | 2017 | Belgium: Godinne |
| GCF-900178785.1 | Human | n/a | n/a | 2017 | Belgium: Godinne |
| GCF-900178795.1 | Human | n/a | n/a | 2017 | Belgium: Godinne |
| GCF-026950455.1 | Urine | Homo sapiens | Urinary tract infection | 2017 | Belgium: Sint-Niklaas |
| GCF-900178805.1 | Human | n/a | n/a | 2017 | Belgium: Gilly |
| GCF-900178815.1 | Human | n/a | n/a | 2017 | Belgium: Brussels |
| GCF-900178825.1 | Human | n/a | n/a | 2012 | Belgium: Turnhout |
| GCF-900178835.1 | Human | n/a | n/a | 2018 | Belgium: Seraing |
| GCF-900178845.1 | Human | n/a | n/a | 2018 | Belgium: Mons |
| GCF-900178855.1 | Human | n/a | n/a | 2010 | Belgium: Leuven |
| GCF-900178865.1 | Human | n/a | n/a | 2018 | Belgium: Roeselare |
| GCF-900178875.1 | Human | n/a | n/a | 2018 | Belgium: Brussels |
| GCF-026951045.1 | Urine | Homo sapiens | Urinary tract infection | 2018 | Belgium: Roeselare |
| GCF-900178885.1 | Human | n/a | n/a | 2018 | Belgium: Roeselare |
| GCF-900178895.1 | Human | n/a | n/a | 2018 | Belgium: Liege |
| GCF-900178905.1 | Human | n/a | n/a | 2018 | Belgium: Roeselare |
| GCF-900178915.1 | Human | n/a | n/a | 2018 | Belgium: La Louviere |
| GCF-900178925.1 | Human | n/a | n/a | 2018 | Belgium: Brussels |
| GCF-900178935.1 | Human | n/a | n/a | 2018 | Belgium: Boussu |
| GCF-900178945.1 | Human | n/a | n/a | 2018 | Belgium: Brussels |
| GCF-900178955.1 | Human | n/a | n/a | 2018 | Belgium: Verviers |
| GCF-026951565.1 | Urine | Homo sapiens | Urinary tract infection | 2018 | Belgium: Verviers |
| GCF-900178965.1 | Human | n/a | n/a | 2018 | Belgium: Brussels |
| GCF-900178975.1 | Human | n/a | n/a | 2018 | Belgium: Brussels |
| GCF-900178985.1 | Human | n/a | n/a | 2018 | Belgium: Brussels |
| GCF-026951825.1 | Urine | Homo sapiens | Urinary tract infection | 2018 | Belgium: Brussels |
| GCF-026951835.1 | Bladder probe | Homo sapiens | Respiratory tract infection | 2017 | Belgium: Waregem |
| GCF-900178995.1 | Human | n/a | n/a | 2018 | Belgium: Brussels |
| GCF-900179015.1 | Human | n/a | n/a | 2014 | Belgium: Sint-Niklaas |
| GCF-900179025.1 | Human | n/a | n/a | 2018 | Belgium: Brussels |
| GCF-900179045.1 | Human | n/a | n/a | 2014 | Belgium: Edegem |
| GCF-900179055.1 | Human | n/a | n/a | 2014 | Belgium: Edegem |
| GCF-900179065.1 | Human | n/a | n/a | 2014 | Belgium: Namur |
| GCF-900179075.1 | Human | n/a | n/a | 2014 | Belgium: Gent |
| GCF-900179085.1 | Human | n/a | n/a | 2014 | Belgium: Leuven |
| GCF-900179095.1 | Human | n/a | n/a | 2014 | Belgium: Assebroek |
| GCF-900179105.1 | Human | n/a | n/a | 2014 | Belgium: Tournai |
| GCF-026952505.1 | Blood | Homo sapiens | Peritonitis | 2014 | Belgium: Edegem |
| GCF-900179115.1 | Human | n/a | n/a | 2013 | Belgium: Ronse |
| GCF-900179125.1 | Human | n/a | n/a | 2014 | Belgium: Tournai |
| GCF-900179135.1 | Human | n/a | n/a | 2016 | Belgium: Gilly |
| GCF-900179145.1 | Human | n/a | n/a | 2018 | Belgium: Brussels |
| GCF-900179155.1 | Human | n/a | n/a | 2016 | Belgium: Roeselare |
| GCF-900179165.1 | Human | n/a | n/a | 2016 | Belgium: Leuven |
| GCF-900179175.1 | Human | n/a | n/a | 2016 | Belgium: Gilly |
| GCF-900179185.1 | Human | n/a | n/a | 2016 | Belgium: Brussels |
| GCF-900179195.1 | Human | n/a | n/a | 2016 | Belgium: Gilly |
| GCF-900179215.1 | Human | n/a | n/a | 2016 | Belgium: Gilly |
| GCF-900179225.1 | Human | n/a | n/a | 2016 | Belgium: Leuven |
| GCF-900179235.1 | Human | n/a | n/a | 2016 | Belgium: Gilly |
| GCF-900179245.1 | Human | n/a | n/a | 2016 | Belgium: Gilly |
| GCF-900179255.1 | Human | n/a | n/a | 2016 | Belgium: Roeselare |
| GCF-900179265.1 | Human | n/a | n/a | 2016 | Belgium: Ath |
| GCF-900179285.1 | Human | n/a | n/a | 2015 | Belgium: Mechelen |
| GCF-900179295.1 | Human | n/a | n/a | 2015 | Belgium: Hasselt |
| GCF-900179305.1 | Human | n/a | n/a | 2015 | Belgium: Mechelen |
| GCF-900179315.1 | Human | n/a | n/a | 2015 | Belgium: Gilly |

| | | | | | |
|---|---|---|---|---|---|
| GCF-900179325.1 | Human | n/a | n/a | 2015 | Belgium: Liege |
| GCF-900179335.1 | Human | n/a | n/a | 1991 | Germany |
| GCF-900179345.1 | Human | n/a | n/a | 2012 | Denmark: Copenhagen |
| GCF-900179365.1 | Human | n/a | n/a | 2019 | Belgium: Wilrijk |
| GCF-900179375.1 | Human | n/a | n/a | 2019 | Belgium: Wilrijk |
| GCF-900179385.1 | Human | n/a | n/a | 2019 | Belgium: Wilrijk |
| GCF-900179395.1 | Human | n/a | n/a | 2019 | Belgium: Wilrijk |
| GCF-900179405.1 | Human | n/a | n/a | 2019 | Belgium: Wilrijk |
| GCF-900179415.1 | Human | n/a | n/a | 2019 | Belgium: Wilrijk |
| GCF-027286245.1 | Urine | Homo sapiens | Infection | 2022 | China: Zhuhai |
| GCF-900179425.1 | Human | n/a | n/a | 2014 | China: Shenzhen |
| GCF-900179435.1 | Human | n/a | n/a | 2014 | China: Shenzhen |
| GCF-900179445.1 | Human | n/a | n/a | 2014 | China: Shenzhen |
| GCF-900179455.1 | Human | n/a | n/a | 2014 | China: Shenzhen |
| GCF-900179465.1 | Human | n/a | n/a | 2014 | China: Shenzhen |
| GCF-900179475.1 | Human | n/a | n/a | 2014 | China: Shenzhen |
| GCF-900179485.1 | Human | n/a | n/a | 2014 | China: Shenzhen |
| GCF-900179505.1 | Human | n/a | n/a | 2014 | China: Shenzhen |
| GCF-900179515.1 | Human | n/a | n/a | 2014 | China: Shenzhen |
| GCF-900179525.1 | Human | n/a | n/a | 2014 | China: Shenzhen |
| GCF-900179535.1 | Human | n/a | n/a | 2014 | China: Shenzhen |
| GCF-900179545.1 | Human | n/a | n/a | 2014 | China: Shenzhen |
| GCF-900179555.1 | Human | n/a | n/a | 2014 | China: Shenzhen |
| GCF-900179565.1 | Human | n/a | n/a | 2014 | China: Shenzhen |
| GCF-900179575.1 | Human | n/a | n/a | 2014 | China: Shenzhen |
| GCF-900179585.1 | Human | n/a | n/a | 2014 | China: Shenzhen |
| GCF-900179595.1 | Human | n/a | n/a | 2020 | South Korea |
| GCF-900179605.1 | Human | n/a | n/a | 2022 | China:Zhejiang |
| GCF-900179615.1 | Human | n/a | n/a | 2021 | n/a |
| GCF-900179625.1 | Human | n/a | n/a | 2021 | n/a |
| GCF-900179635.1 | Human | n/a | n/a | 2021 | n/a |
| GCF-900179645.1 | Human | n/a | n/a | 2021 | n/a |
| GCF-900179655.1 | Human | n/a | n/a | 2021 | n/a |
| GCF-900179665.1 | Human | n/a | n/a | 2021 | n/a |
| GCF-900179675.1 | Human | n/a | n/a | 2021 | n/a |
| GCF-900179685.1 | Human | n/a | n/a | 2021 | n/a |
| GCF-900179695.1 | Human | n/a | n/a | 2021 | n/a |
| GCF-900179705.1 | Human | n/a | n/a | 2021 | n/a |
| GCF-900179715.1 | Human | n/a | n/a | 2021 | n/a |
| GCF-900179725.1 | Human | n/a | n/a | n/a | Peru: Lima |
| GCF-900179735.1 | Human | n/a | n/a | n/a | Peru: Lima |
| GCF-900179745.1 | Human | n/a | n/a | 2021 | n/a |
| GCF-900179755.1 | Human | n/a | n/a | 2021 | China:zhumadian |
| GCF-900179765.1 | Human | n/a | n/a | n/a | n/a |
| GCF-900179775.1 | Human | n/a | n/a | 1998 | n/a |
| GCF-900179785.1 | Human | n/a | n/a | 2011 | Australia |
| GCF-900179795.1 | Human | n/a | n/a | 2014 | Australia |
| GCF-900179835.1 | Human | n/a | n/a | n/a | n/a |
| GCF-900179845.1 | Human | n/a | n/a | n/a | n/a |
| GCF-900179855.1 | Human | n/a | n/a | n/a | n/a |
| GCF-900179865.1 | Human | n/a | n/a | n/a | n/a |
| GCF-900179885.1 | Human | n/a | n/a | n/a | n/a |
| GCF-900179895.1 | Human | n/a | n/a | n/a | n/a |
| GCF-900179905.1 | Human | n/a | n/a | n/a | n/a |
| GCF-900179915.1 | Human | n/a | n/a | n/a | n/a |
| GCF-900179925.1 | Human | n/a | n/a | n/a | n/a |
| GCF-900179935.1 | Human | n/a | n/a | n/a | n/a |
| GCF-900179945.1 | Human | n/a | n/a | n/a | n/a |
| GCF-900179965.1 | Human | n/a | n/a | 2015 | United Kingdom |
| GCF-900179975.1 | Human | n/a | n/a | 2015 | United Kingdom |
| GCF-900179985.1 | Human | n/a | n/a | 2015 | United Kingdom |
| GCF-900179995.1 | Human | n/a | n/a | 2015 | United Kingdom |
| GCF-900180015.1 | Human | n/a | n/a | 2015 | United Kingdom |
| GCF-900180025.1 | Human | n/a | n/a | 2015 | United Kingdom |
| GCF-900180035.1 | Human | n/a | n/a | 2015 | United Kingdom |
| GCF-900180045.1 | Human | n/a | n/a | 2015 | United Kingdom |
| GCF-900180075.1 | Human | n/a | n/a | 2015 | United Kingdom |
| GCF-900180085.1 | Human | n/a | n/a | 2015 | United Kingdom |
| GCF-900180115.1 | Human | n/a | n/a | 2015 | United Kingdom |
| GCF-900180125.1 | Human | n/a | n/a | 2015 | United Kingdom |
| GCF-900180135.1 | Human | n/a | n/a | 2015 | United Kingdom |
| GCF-900180145.1 | Human | n/a | n/a | 2015 | United Kingdom |
| GCF-900180155.1 | Human | n/a | n/a | 2015 | United Kingdom |
| GCF-900180165.1 | Human | n/a | n/a | 2015 | United Kingdom |
| GCF-900180175.1 | Human | n/a | n/a | 2015 | United Kingdom |
| GCF-900180185.1 | Human | n/a | n/a | 2015 | United Kingdom |
| GCF-900180195.1 | Human | n/a | n/a | 2015 | United Kingdom |
| GCF-900180205.1 | Human | n/a | n/a | 2015 | United Kingdom |
| GCF-900180235.1 | Human | n/a | n/a | 2015 | United Kingdom |
| GCF-900180245.1 | Human | n/a | n/a | 2015 | United Kingdom |
| GCF-900180265.1 | Human | n/a | n/a | 2015 | United Kingdom |
| GCF-900180275.1 | Human | n/a | n/a | 2015 | United Kingdom |
| GCF-900180285.1 | Human | n/a | n/a | 2015 | United Kingdom |
| GCF-900180295.1 | Human | n/a | n/a | 2015 | United Kingdom |
| GCF-900180305.1 | Human | n/a | n/a | 2015 | United Kingdom |
| GCF-900180325.1 | Human | n/a | n/a | 2015 | United Kingdom |
| GCF-900180455.1 | Human | n/a | n/a | 2015 | United Kingdom |
| GCF-900180465.1 | Human | n/a | n/a | 2015 | United Kingdom |
| GCF-900634805.1 | n/a | n/a | n/a | 2015 | United Kingdom |

| | | | | | |
|---|---|---|---|---|---|
| GCF-900635365.1 | n/a | n/a | n/a | 2015 | United Kingdom |
| GCF-900635415.1 | n/a | n/a | n/a | 2015 | United Kingdom |
| GCF-900637035.1 | n/a | n/a | n/a | 2015 | United Kingdom |
| GCF-900638765.1 | n/a | n/a | n/a | 2015 | United Kingdom |
| GCF-900638775.1 | n/a | n/a | n/a | 2015 | United Kingdom |
| GCF-900638785.1 | n/a | n/a | n/a | 2015 | United Kingdom |
| GCF-900638805.1 | n/a | n/a | n/a | 2015 | United Kingdom |
| GCF-900638815.1 | n/a | n/a | n/a | 2015 | United Kingdom |
| GCF-900638825.1 | n/a | n/a | n/a | 2015 | United Kingdom |
| GCF-900639335.1 | n/a | n/a | n/a | 2015 | United Kingdom |
| GCF-900639345.1 | n/a | n/a | n/a | 2015 | United Kingdom |
| GCF-900639355.1 | n/a | n/a | n/a | 2015 | United Kingdom |
| GCF-900639365.1 | n/a | n/a | n/a | 2015 | United Kingdom |
| GCF-900639385.1 | n/a | n/a | n/a | 2015 | United Kingdom |
| GCF-900639395.1 | n/a | n/a | n/a | 2015 | United Kingdom |
| GCF-900639405.1 | n/a | n/a | n/a | 2015 | United Kingdom |
| GCF-900639415.1 | n/a | n/a | n/a | 2015 | United Kingdom |
| GCF-900639425.1 | n/a | n/a | n/a | 2015 | United Kingdom |
| GCF-900639445.1 | n/a | n/a | n/a | 2015 | United Kingdom |
| GCF-900639455.1 | n/a | n/a | n/a | 2015 | United Kingdom |
| GCF-900639465.1 | n/a | n/a | n/a | 2015 | United Kingdom |
| GCF-900639485.1 | n/a | n/a | n/a | 2015 | United Kingdom |
| GCF-900639495.1 | n/a | n/a | n/a | 2015 | United Kingdom |
| GCF-900639505.1 | n/a | n/a | n/a | 2015 | United Kingdom |
| GCF-900639515.1 | n/a | n/a | n/a | 2015 | United Kingdom |
| GCF-900639525.1 | n/a | n/a | n/a | 2015 | United Kingdom |
| GCF-900639535.1 | n/a | n/a | n/a | 2015 | United Kingdom |
| GCF-900639545.1 | n/a | n/a | n/a | 2015 | United Kingdom |
| GCF-900639555.1 | n/a | n/a | n/a | 2015 | United Kingdom |
| GCF-900639565.1 | n/a | n/a | n/a | 2015 | United Kingdom |
| GCF-900639575.1 | n/a | n/a | n/a | 2015 | United Kingdom |
| GCF-900639585.1 | n/a | n/a | n/a | 2015 | United Kingdom |
| GCF-900639595.1 | n/a | n/a | n/a | 2015 | United Kingdom |
| GCF-900639605.1 | n/a | n/a | n/a | 2015 | United Kingdom |
| GCF-900639615.1 | n/a | n/a | n/a | 2015 | United Kingdom |
| GCF-900639625.1 | n/a | n/a | n/a | 2015 | United Kingdom |
| GCF-900639635.1 | n/a | n/a | n/a | 2015 | United Kingdom |
| GCF-900639655.1 | n/a | n/a | n/a | 2015 | United Kingdom |
| GCF-900639705.1 | n/a | n/a | n/a | 2015 | United Kingdom |
| GCF-900639715.1 | n/a | n/a | n/a | 2015 | United Kingdom |
| GCF-900639725.1 | n/a | n/a | n/a | 2015 | United Kingdom |
| GCF-900639745.1 | n/a | n/a | n/a | 2015 | United Kingdom |
| GCF-900640265.1 | n/a | n/a | n/a | 2015 | United Kingdom |
| GCF-900682725.1 | n/a | n/a | n/a | 2015 | United Kingdom |
| GCF-900683475.1 | n/a | n/a | n/a | 2015 | United Kingdom |
| GCF-902158975.1 | Stool | Homo sapiens | n/a | 2015 | United Kingdom |
| GCF-902159225.1 | Stool | Homo sapiens | n/a | 2015 | United Kingdom |
| GCF-902159305.1 | Stool | Homo sapiens | n/a | 2015 | United Kingdom |
| GCF-902159325.1 | Stool | Homo sapiens | n/a | 2015 | United Kingdom |
| GCF-902159345.1 | Stool | Homo sapiens | n/a | 2015 | United Kingdom |
| GCF-902159355.1 | Stool | Homo sapiens | n/a | 2015 | United Kingdom |
| GCF-902159365.1 | Stool | Homo sapiens | n/a | 2015 | United Kingdom |
| GCF-902159505.1 | Stool | Homo sapiens | n/a | 2015 | United Kingdom |
| GCF-902159555.1 | Stool | Homo sapiens | n/a | 2015 | United Kingdom |
| GCF-902159925.1 | Stool | Homo sapiens | n/a | 2015 | United Kingdom |
| GCF-902160485.1 | Stool | Homo sapiens | n/a | 2015 | United Kingdom |
| GCF-902160525.1 | Stool | Homo sapiens | n/a | 2015 | United Kingdom |
| GCF-902160645.1 | Stool | Homo sapiens | n/a | 2015 | United Kingdom |
| GCF-902160705.1 | Stool | Homo sapiens | n/a | 2015 | United Kingdom |
| GCF-902160865.1 | Stool | Homo sapiens | n/a | 2015 | United Kingdom |
| GCF-902160955.1 | Stool | Homo sapiens | n/a | 2015 | United Kingdom |
| GCF-902161175.1 | Stool | Homo sapiens | n/a | 2015 | United Kingdom |
| GCF-902161605.1 | Stool | Homo sapiens | n/a | 2015 | United Kingdom |
| GCF-902161815.1 | Stool | Homo sapiens | n/a | 2015 | United Kingdom |
| GCF-902161965.1 | Stool | Homo sapiens | n/a | 2015 | United Kingdom |
| GCF-902162005.1 | Stool | Homo sapiens | n/a | 2015 | United Kingdom |
| GCF-902162355.1 | Stool | Homo sapiens | n/a | 2015 | United Kingdom |
| GCF-902163145.1 | Stool | Homo sapiens | n/a | 2015 | United Kingdom |
| GCF-902164005.1 | Stool | Homo sapiens | n/a | 2015 | United Kingdom |
| GCF-902164105.1 | Stool | Homo sapiens | n/a | 2015 | United Kingdom |
| GCF-902164525.1 | Stool | Homo sapiens | n/a | 2015 | United Kingdom |
| GCF-902165155.1 | Stool | Homo sapiens | n/a | 2015 | United Kingdom |
| GCF-902165245.1 | Stool | Homo sapiens | n/a | 2015 | United Kingdom |
| GCF-902165375.1 | Stool | Homo sapiens | n/a | 2015 | United Kingdom |
| GCF-902165525.1 | Stool | Homo sapiens | n/a | 2015 | United Kingdom |
| GCF-902165855.1 | Stool | Homo sapiens | n/a | 2015 | United Kingdom |
| GCF-902165865.1 | Stool | Homo sapiens | n/a | 2015 | United Kingdom |
| GCF-902165955.1 | Stool | Homo sapiens | n/a | 2015 | United Kingdom |
| GCF-902165965.1 | Stool | Homo sapiens | n/a | 2015 | United Kingdom |
| GCF-902165995.1 | Stool | Homo sapiens | n/a | 2015 | United Kingdom |
| GCF-902166385.1 | Stool | Homo sapiens | n/a | 2015 | United Kingdom |
| GCF-902166525.1 | Stool | Homo sapiens | n/a | 2015 | United Kingdom |
| GCF-902166655.1 | Stool | Homo sapiens | n/a | 2015 | United Kingdom |
| GCF-902166835.1 | Stool | Homo sapiens | n/a | 2015 | United Kingdom |
| GCF-907163255.1 | Blood | Homo sapiens | n/a | 2015 | United Kingdom |
| GCF-907163315.1 | Hepatic abscess | Homo sapiens | n/a | 2015 | United Kingdom |
| GCF-907165365.1 | Rectal swab | Homo sapiens | n/a | 2015 | United Kingdom |
| GCF-907176135.1 | Superficial wound | Homo sapiens | n/a | 2015 | United Kingdom |
| GCF-907176815.1 | Superficial wound | Homo sapiens | n/a | 2015 | United Kingdom |

| | | | | | |
|---|---|---|---|---|---|
| GCF-907177285.1 | Superficial wound | Homo sapiens | n/a | 2015 | United Kingdom |
| GCF-000159675.1 | n/a | Homo sapiens | n/a | 2015 | United Kingdom |
| GCF-000174395.2 | n/a | Homo sapiens | n/a | 2015 | United Kingdom |
| GCF-000250945.1 | Blood | Homo sapiens | n/a | 2015 | United Kingdom |
| GCF-000262105.1 | Broncoalveolar lavage | Homo sapiens | n/a | 2015 | United Kingdom |
| GCF-000294345.2 | n/a | Homo sapiens | n/a | 2015 | United Kingdom |
| GCF-000294815.2 | n/a | Homo sapiens | n/a | 2015 | United Kingdom |
| GCF-000294835.2 | n/a | Homo sapiens | n/a | 2015 | United Kingdom |
| GCF-000294855.2 | n/a | Homo sapiens | n/a | 2015 | United Kingdom |
| GCF-000294875.2 | n/a | Homo sapiens | n/a | 2015 | United Kingdom |
| GCF-000294895.2 | n/a | Homo sapiens | n/a | 2015 | United Kingdom |
| GCF-000294915.2 | n/a | Homo sapiens | n/a | 2015 | United Kingdom |
| GCF-000294935.2 | n/a | Homo sapiens | n/a | 2015 | United Kingdom |
| GCF-000294955.2 | n/a | Homo sapiens | n/a | 2015 | United Kingdom |
| GCF-000294975.2 | n/a | Homo sapiens | n/a | 2015 | United Kingdom |
| GCF-000294995.2 | n/a | Homo sapiens | n/a | 2015 | United Kingdom |
| GCF-000295015.1 | n/a | Homo sapiens | n/a | 2015 | United Kingdom |
| GCF-000295035.2 | n/a | Homo sapiens | n/a | 2015 | United Kingdom |
| GCF-000295055.2 | n/a | Homo sapiens | n/a | 2015 | United Kingdom |
| GCF-000295075.2 | n/a | Homo sapiens | n/a | 2015 | United Kingdom |
| GCF-000295115.2 | n/a | Homo sapiens | n/a | 2015 | United Kingdom |
| GCF-000295135.2 | n/a | Homo sapiens | n/a | 2015 | United Kingdom |
| GCF-000295155.2 | n/a | Homo sapiens | n/a | 2015 | United Kingdom |
| GCF-000295175.2 | n/a | Homo sapiens | n/a | 2015 | United Kingdom |
| GCF-000295195.2 | n/a | Homo sapiens | n/a | 2015 | United Kingdom |
| GCF-000295215.2 | n/a | Homo sapiens | n/a | 2015 | United Kingdom |
| GCF-000295235.2 | n/a | Homo sapiens | n/a | 2015 | United Kingdom |
| GCF-000295255.2 | n/a | Homo sapiens | n/a | 2015 | United Kingdom |
| GCF-000295275.2 | n/a | Homo sapiens | n/a | 2015 | United Kingdom |
| GCF-000295315.2 | n/a | Homo sapiens | n/a | 2015 | United Kingdom |
| GCF-000295335.2 | n/a | Homo sapiens | n/a | 2015 | United Kingdom |
| GCF-000295355.2 | n/a | Homo sapiens | n/a | 2015 | United Kingdom |
| GCF-000295375.2 | n/a | Homo sapiens | n/a | 2015 | United Kingdom |
| GCF-000295395.2 | n/a | Homo sapiens | n/a | 2015 | United Kingdom |
| GCF-000295415.2 | n/a | Homo sapiens | n/a | 2015 | United Kingdom |
| GCF-000295435.2 | n/a | Homo sapiens | n/a | 2015 | United Kingdom |
| GCF-000295455.2 | n/a | Homo sapiens | n/a | 2015 | United Kingdom |
| GCF-000295495.2 | n/a | Homo sapiens | n/a | 2015 | United Kingdom |
| GCF-000295515.2 | n/a | Homo sapiens | n/a | 2015 | United Kingdom |
| GCF-000295535.2 | n/a | Homo sapiens | n/a | 2015 | United Kingdom |
| GCF-000295555.2 | n/a | Homo sapiens | n/a | n/a | n/a |
| GCF-000295575.2 | n/a | Homo sapiens | n/a | n/a | n/a |
| GCF-000295595.2 | n/a | Homo sapiens | n/a | n/a | n/a |
| GCF-000295615.2 | n/a | Homo sapiens | n/a | n/a | n/a |
| GCF-000315405.1 | n/a | Homo sapiens | n/a | n/a | n/a |
| GCF-000321465.1 | Stool | Gallus gallus domesticus | n/a | n/a | n/a |
| GCF-000321485.1 | Ascites | Homo sapiens | n/a | n/a | n/a |
| GCF-000321505.1 | Stool | Meleagris gallopavo | n/a | n/a | n/a |
| GCF-000321525.1 | Stool | Meleagris gallopavo | n/a | n/a | n/a |
| GCF-000321545.1 | Blood | Homo sapiens | n/a | n/a | n/a |
| GCF-002562805.1 | Blood | Homo sapiens | Nosocomial infection | n/a | n/a |
| GCF-002562815.1 | Wound | Homo sapiens | Nosocomial infection | n/a | n/a |
| GCF-002562855.1 | Skin | Homo sapiens | Nosocomial infection | n/a | n/a |
| GCF-002562865.1 | Burn wound | Homo sapiens | Nosocomial infection | n/a | n/a |
| GCF-002562875.1 | Skin | Homo sapiens | Nosocomial infection | n/a | n/a |
| GCF-002630965.1 | Wound | Homo sapiens | Nosocomial infection | n/a | n/a |
| GCF-002630975.1 | Stool | Homo sapiens | Nosocomial infection | n/a | n/a |
| GCF-002630985.1 | Wound | Homo sapiens | Nosocomial infection | n/a | n/a |
| GCF-002631165.1 | Skin | Homo sapiens | Nosocomial infection | n/a | n/a |
| GCF-008123945.1 | Stool | Homo sapiens | Pancreatitis | n/a | n/a |
| GCF-012932975.2 | Blood | Homo sapiens | Sepsis | n/a | n/a |
| GCF-012932985.2 | Blood | Homo sapiens | Sepsis | 2010 | Netherlands |
| GCF-012933055.2 | Blood | Homo sapiens | Sepsis | n/a | n/a |
| GCF-012933075.2 | Blood | Homo sapiens | Sepsis | 2012 | Netherlands |
| GCF-012933165.2 | Blood | Homo sapiens | Sepsis | 2012 | Netherlands |
| GCF-012933195.2 | Blood | Homo sapiens | Sepsis | 2013 | Netherlands |
| GCF-012933245.2 | Blood | Homo sapiens | Sepsis | 2013 | Netherlands |
| GCF-012933265.2 | Blood | Homo sapiens | Sepsis | 2015 | Netherlands |
| GCF-012933285.2 | Blood | Homo sapiens | Sepsis | 2013 | Netherlands |
| GCF-012933295.2 | Blood | Homo sapiens | Sepsis | 2014 | Netherlands |
| GCF-012933345.2 | Blood | Homo sapiens | Sepsis | 2014 | Netherlands |
| GCF-014874615.1 | Blood | Homo sapiens | sepsis | 2014 | Netherlands |
| GCF-017603725.1 | Blood | Homo sapiens | Sepsis | 2013 | Netherlands |
| GCF-017815655.1 | Blood | Homo sapiens | Sepsis | 2015 | Netherlands |
| GCF-017815675.1 | Blood | Homo sapiens | Sepsis | 2015 | Netherlands |
| GCF-017815695.1 | Blood | Homo sapiens | Sepsis | 2015 | Netherlands |
| GCF-017897965.1 | Blood | Homo sapiens | sepsis | 2015 | Netherlands |
| GCF-017898005.1 | Blood | Homo sapiens | sepsis | n/a | n/a |
| GCF-017898025.1 | Blood | Homo sapiens | Sepsis | n/a | n/a |
| GCF-001696305.1 | Rectal swab | Homo sapiens | Sepsis | n/a | n/a |
| GCF-002831505.1 | Clinical isolate | Moschus berezovskii | Sepsis | 2015 | Netherlands |
| GCF-026922095.1 | Blood | Homo sapiens | Sepsis | 2015 | Netherlands |
| GCF-026922335.1 | Clinical isolate | Homo sapiens | Sepsis | n/a | n/a |
| GCF-026928635.1 | Blood | Homo sapiens | Sepsis | n/a | n/a |
| GCF-026928695.1 | Blood | Homo sapiens | Sepsis | 2015 | Netherlands |
| GCF-026928855.1 | Blood | Homo sapiens | Sepsis | n/a | n/a |
| GCF-026928895.1 | Blood | Homo sapiens | Sepsis | 2018 | United Kingdom |
| GCF-026929035.1 | Blood | Homo sapiens | Sepsis | 2018 | United Kingdom |
| GCF-026929055.1 | Blood | Homo sapiens | Sepsis | 2018 | United Kingdom |

| | | | | | |
|---|---|---|---|---|---|
| GCF-026934565.1 | Blood | Homo sapiens | Sepsis | 2018 | United Kingdom |
| GCF-026942055.1 | Blood | Homo sapiens | Sepsis | 2018 | United Kingdom |
| GCF-026943515.1 | Blood | Homo sapiens | Sepsis | 2018 | United Kingdom |
| GCF-026944685.1 | Rectal swab | Homo sapiens | Sepsis | 2018 | United Kingdom |
| GCF-026945185.1 | Blood | Homo sapiens | Sepsis | 2018 | United Kingdom |
| GCF-026945195.1 | Blood | Homo sapiens | Sepsis | 2018 | United Kingdom |
| GCF-026945405.1 | Blood | Homo sapiens | Sepsis | 2018 | United Kingdom |
| GCF-026947425.1 | Blood | Homo sapiens | Sepsis | 2018 | United Kingdom |
| GCF-026947505.1 | Blood | Homo sapiens | Sepsis | 2018 | United Kingdom |
| GCF-026947605.1 | Blood | Homo sapiens | Sepsis | 2018 | United Kingdom |
| GCF-026947765.1 | Blood | Homo sapiens | Sepsis | 2018 | United Kingdom |
| GCF-026948185.1 | Blood | Homo sapiens | Sepsis | 2018 | United Kingdom |
| GCF-026948255.1 | Blood | Homo sapiens | Sepsis | 2018 | United Kingdom |
| GCF-026948505.1 | Blood | Homo sapiens | Sepsis | 2018 | United Kingdom |
| GCF-026948645.1 | Blood | Homo sapiens | Sepsis | 2018 | United Kingdom |
| GCF-026949325.1 | Blood | Homo sapiens | Sepsis | 2018 | United Kingdom |
| GCF-026949465.1 | Blood | Homo sapiens | Sepsis | 2018 | United Kingdom |
| GCF-026950845.1 | Blood | Homo sapiens | Sepsis | 2018 | United Kingdom |
| GCF-026951995.1 | Blood | Homo sapiens | Sepsis | 2018 | United Kingdom |
| GCF-026952305.1 | Blood | Homo sapiens | Sepsis | 2018 | United Kingdom |
| GCF-026952325.1 | Blood | Homo sapiens | Sepsis | 2018 | United Kingdom |
| GCF-026952365.1 | Blood | Homo sapiens | Sepsis | 2018 | United Kingdom |
| GCF-026953045.1 | Blood | Homo sapiens | Sepsis | 2018 | United Kingdom |
| GCF-026953125.1 | Blood | Homo sapiens | Sepsis | 2018 | United Kingdom |
| GCF-026953145.1 | Blood | Homo sapiens | Sepsis | 2018 | United Kingdom |
| GCF-026953185.1 | Blood | Homo sapiens | Sepsis | 2018 | United Kingdom |
| GCF-026953245.1 | Blood | Homo sapiens | Sepsis | 2018 | United Kingdom |
| GCF-026953255.1 | Blood | Homo sapiens | Sepsis | 2018 | United Kingdom |
| GCF-026953395.1 | Blood | Homo sapiens | Sepsis | 2018 | United Kingdom |
| GCF-001542895.1 | Stool | Homo sapiens | Colonization | 2018 | United Kingdom |
| GCF-010120755.1 | Clinical isolate | Homo sapiens | Uremia | 2018 | United Kingdom |
| GCF-008921725.1 | Urine | Homo sapiens | Urine tract infection | 2018 | United Kingdom |
| GCF-002761255.1 | Peritoneal fluid | Homo sapiens | Uterus cancer | 2018 | United Kingdom |
| GCF-002761275.1 | Peritoneal fluid | Homo sapiens | Uterus cancer | 2018 | United Kingdom |
| GCF-022691445.1 | Urine | Homo sapiens | Urine tract infection | 2018 | United Kingdom |
| GCF-022693745.1 | Urine | Homo sapiens | Urine tract infection | 2018 | United Kingdom |
| GCF-003020685.1 | Clinical isolate | Homo sapiens | Infection | 2012 | New Zealand |
| GCF-003020705.1 | Clinical isolate | Homo sapiens | Infection | 2012 | New Zealand |
| GCF-003020725.1 | Blood | Homo sapiens | Infection | 2017 | Switzerland |
| GCF-003020745.1 | Blood | Homo sapiens | Infection | 2015 | Switzerland |
| GCF-003020765.1 | Blood | Homo sapiens | Infection | 2018 | Switzerland |
| GCF-026942675.1 | Wound | Homo sapiens | Wound | 2018 | Switzerland |
| FI100 | Hospital Surface | Hospital Surface | Hospital Surface | 2017 | Czech republic: Brno |
| FI101 | Hospital Surface | Hospital Surface | Hospital Surface | 2017 | Czech republic: Brno |
| FI102 | Rectal swab | Homo sapiens | Colonization | 2017 | Czech republic: Brno |
| FI104 | Rectal swab | Homo sapiens | Colonization | 2017 | Czech republic: Brno |
| FI105 | Rectal swab | Homo sapiens | Colonization | 2017 | Czech republic: Brno |
| FI108 | Rectal swab | Homo sapiens | Colonization | 2017 | Czech republic: Brno |
| FI109 | Rectal swab | Homo sapiens | Colonization | 2017 | Czech republic: Brno |
| FI110 | Rectal swab | Homo sapiens | Colonization | 2017 | Czech republic: Brno |
| FI111 | Rectal swab | Homo sapiens | Colonization | 2017 | Czech republic: Brno |
| FI112 | Rectal swab | Homo sapiens | Colonization | 2017 | Czech republic: Brno |
| FI113 | Rectal swab | Homo sapiens | Colonization | 2017 | Czech republic: Brno |
| FI114 | Hospital Surface | Hospital Surface | Hospital Surface | 2017 | Czech republic: Brno |
| FI115 | Hospital Surface | Hospital Surface | Hospital Surface | 2017 | Czech republic: Brno |
| FI116 | Hospital Surface | Hospital Surface | Hospital Surface | 2017 | Czech republic: Brno |
| FI117 | Rectal swab | Homo sapiens | Colonization | 2017 | Czech republic: Brno |
| FI119 | Rectal swab | Homo sapiens | Colonization | 2017 | Czech republic: Brno |
| FI120 | Rectal swab | Homo sapiens | Colonization | 2017 | Czech republic: Brno |
| FI121 | Rectal swab | Homo sapiens | Colonization | 2017 | Czech republic: Brno |
| FI122 | Rectal swab | Homo sapiens | Colonization | 2017 | Czech republic: Brno |
| FI123 | Oral cavity | Homo sapiens | Colonization | 2017 | Czech republic: Brno |
| FI124 | Rectal swab | Homo sapiens | Colonization | 2017 | Czech republic: Brno |
| FI127 | Rectal swab | Homo sapiens | Colonization | 2017 | Czech republic: Brno |
| FI128 | Rectal swab | Homo sapiens | Colonization | 2017 | Czech republic: Brno |
| FI129 | Rectal swab | Homo sapiens | Colonization | 2017 | Czech republic: Brno |
| FI130 | Rectal swab | Homo sapiens | Colonization | 2017 | Czech republic: Brno |
| FI131 | Rectal swab | Homo sapiens | Colonization | 2017 | Czech republic: Brno |
| FI132 | Rectal swab | Homo sapiens | Colonization | 2017 | Czech republic: Brno |
| FI134 | Rectal swab | Homo sapiens | Colonization | 2017 | Czech republic: Brno |
| FI135 | Rectal swab | Homo sapiens | Colonization | 2017 | Czech republic: Brno |
| FI136 | Rectal swab | Homo sapiens | Colonization | 2017 | Czech republic: Brno |
| FI137 | Rectal swab | Homo sapiens | Colonization | 2017 | Czech republic: Brno |
| FI138 | Rectal swab | Homo sapiens | Colonization | 2017 | Czech republic: Brno |
| FI139 | Rectal swab | Homo sapiens | Colonization | 2017 | Czech republic: Brno |
| FI140 | Rectal swab | Homo sapiens | Colonization | 2017 | Czech republic: Brno |
| FI141 | Oral cavity | Homo sapiens | Colonization | 2017 | Czech republic: Brno |
| FI142 | Rectal swab | Homo sapiens | Colonization | 2017 | Czech republic: Brno |
| FI143 | Stool | Homo sapiens | Colonization | 2017 | Czech republic: Brno |
| FI144 | Urine | Homo sapiens | Bacteriuria | 2017 | Czech republic: Brno |
| FI146 | Urine | Homo sapiens | Bacteriuria | 2017 | Czech republic: Brno |
| FI148 | Rectal swab | Homo sapiens | Colonization | 2017 | Czech republic: Brno |
| FI149 | Rectal swab | Homo sapiens | Colonization | 2017 | Czech republic: Brno |
| FI150 | Rectal swab | Homo sapiens | Colonization | 2017 | Czech republic: Brno |
| FI151 | Wound | Homo sapiens | Colonization | 2017 | Czech republic: Brno |
| FI152 | Swab | Homo sapiens | Colonization | 2017 | Czech republic: Brno |
| FI153 | Rectal swab | Homo sapiens | Colonization | 2017 | Czech republic: Brno |
| FI155 | Rectal swab | Homo sapiens | Colonization | 2017 | Czech republic: Brno |
| FI156 | Rectal swab | Homo sapiens | Colonization | 2017 | Czech republic: Brno |

| | | | | | |
|---|---|---|---|---|---|
| FI161 | Urine | Homo sapiens | Bacteriuria | 2017 | Czech republic: Brno |
| FI162 | Rectal swab | Homo sapiens | Colonization | 2017 | Czech republic: Brno |
| FI163 | Rectal swab | Homo sapiens | Colonization | 2017 | Czech republic: Brno |
| FI164 | Rectal swab | Homo sapiens | Colonization | 2017 | Czech republic: Brno |
| FI165 | Rectal swab | Homo sapiens | Colonization | 2017 | Czech republic: Brno |
| FI166 | Rectal swab | Homo sapiens | Colonization | 2017 | Czech republic: Brno |
| FI168 | Rectal swab | Homo sapiens | Colonization | 2017 | Czech republic: Brno |
| FI169 | Urine | Homo sapiens | Bacteriuria | 2017 | Czech republic: Brno |
| FI171 | Rectal swab | Homo sapiens | Colonization | 2017 | Czech republic: Brno |
| FI175 | Rectal swab | Homo sapiens | Colonization | 2018 | Czech republic: Brno |
| FI176 | Rectal swab | Homo sapiens | Colonization | 2018 | Czech republic: Brno |
| FI180 | Urine | Homo sapiens | Bacteriuria | 2018 | Czech republic: Brno |
| FI183 | Rectal swab | Homo sapiens | Colonization | 2018 | Czech republic: Brno |
| FI184 | Respiratory tract secretion | Homo sapiens | Colonization | 2018 | Czech republic: Brno |
| FI190 | Rectal swab | Homo sapiens | Colonization | 2018 | Czech republic: Brno |
| FI191 | Abdomen cavity | Homo sapiens | Infection | 2018 | Czech republic: Brno |
| FI196 | Rectal swab | Homo sapiens | Colonization | 2018 | Czech republic: Brno |
| FI199 | Rectal swab | Homo sapiens | Colonization | 2018 | Czech republic: Brno |
| FI204 | Rectal swab | Homo sapiens | Colonization | 2018 | Czech republic: Brno |
| FI238 | Rectal swab | Homo sapiens | Colonization | 2018 | Czech republic: Brno |
| FI244 | Respiratory tract secretion | Homo sapiens | Colonization | 2018 | Czech republic: Brno |
| FI278 | Wound | Homo sapiens | Infection | 2018 | Czech republic: Brno |
| FI286 | Stool | Homo sapiens | Colonization | 2018 | Czech republic: Brno |
| FI309 | Rectal swab | Homo sapiens | Colonization | 2018 | Czech republic: Brno |
| FI360 | Rectal swab | Homo sapiens | Colonization | 2018 | Czech republic: Brno |
| FI362 | Rectal swab | Homo sapiens | Colonization | 2018 | Czech republic: Brno |
| FI39 | Rectal swab | Homo sapiens | Colonization | 2017 | Czech republic: Brno |
| FI396 | Urine | Homo sapiens | Bacteriuria | 2019 | Czech republic: Brno |
| FI403 | Stool | Homo sapiens | Colonization | 2019 | Czech republic: Brno |
| FI41 | Urine | Homo sapiens | Bacteriuria | 2017 | Czech republic: Brno |
| FI417 | Stool | Homo sapiens | Colonization | 2019 | Czech republic: Brno |
| FI422 | Rectal swab | Homo sapiens | Colonization | 2019 | Czech republic: Brno |
| FI424 | Blood | Homo sapiens | Infection | 2019 | Czech republic: Brno |
| FI428 | Rectal swab | Homo sapiens | Colonization | 2019 | Czech republic: Brno |
| FI429 | Abdomen cavity | Homo sapiens | Infection | 2019 | Czech republic: Brno |
| FI43 | Rectal swab | Homo sapiens | Colonization | 2017 | Czech republic: Brno |
| FI433 | Rectal swab | Homo sapiens | Colonization | 2019 | Czech republic: Brno |
| FI434 | Rectal swab | Homo sapiens | Colonization | 2019 | Czech republic: Brno |
| FI436 | Rectal swab | Homo sapiens | Colonization | 2019 | Czech republic: Brno |
| FI439 | Rectal swab | Homo sapiens | Colonization | 2019 | Czech republic: Brno |
| FI44 | Rectal swab | Homo sapiens | Colonization | 2017 | Czech republic: Brno |
| FI440 | Rectal swab | Homo sapiens | Colonization | 2019 | Czech republic: Brno |
| FI442 | Rectal swab | Homo sapiens | Colonization | 2019 | Czech republic: Brno |
| FI443 | Rectal swab | Homo sapiens | Colonization | 2019 | Czech republic: Brno |
| FI444 | Rectal swab | Homo sapiens | Colonization | 2019 | Czech republic: Brno |
| FI445 | Rectal swab | Homo sapiens | Colonization | 2019 | Czech republic: Brno |
| FI446 | Rectal swab | Homo sapiens | Colonization | 2019 | Czech republic: Brno |
| FI447 | Rectal swab | Homo sapiens | Colonization | 2019 | Czech republic: Brno |
| FI448 | Rectal swab | Homo sapiens | Colonization | 2019 | Czech republic: Brno |
| FI449 | Rectal swab | Homo sapiens | Colonization | 2019 | Czech republic: Brno |
| FI45 | Rectal swab | Homo sapiens | Colonization | 2017 | Czech republic: Brno |
| FI450 | Rectal swab | Homo sapiens | Colonization | 2019 | Czech republic: Brno |
| FI451 | Rectal swab | Homo sapiens | Colonization | 2019 | Czech republic: Brno |
| FI452 | Urine | Homo sapiens | Bacteriuria | 2019 | Czech republic: Brno |
| FI453 | Stool | Homo sapiens | Colonization | 2019 | Czech republic: Brno |
| FI454 | Rectal swab | Homo sapiens | Colonization | 2019 | Czech republic: Brno |
| FI455 | Rectal swab | Homo sapiens | Colonization | 2019 | Czech republic: Brno |
| FI456 | Rectal swab | Homo sapiens | Colonization | 2019 | Czech republic: Brno |
| FI457 | Rectal swab | Homo sapiens | Colonization | 2019 | Czech republic: Brno |
| FI458 | Rectal swab | Homo sapiens | Colonization | 2019 | Czech republic: Brno |
| FI459 | Rectal swab | Homo sapiens | Colonization | 2019 | Czech republic: Brno |
| FI460 | Rectal swab | Homo sapiens | Colonization | 2019 | Czech republic: Brno |
| FI461 | Rectal swab | Homo sapiens | Colonization | 2019 | Czech republic: Brno |
| FI462 | Rectal swab | Homo sapiens | Colonization | 2019 | Czech republic: Brno |
| FI463 | Rectal swab | Homo sapiens | Colonization | 2019 | Czech republic: Brno |
| FI464 | Hospital Surface | Hospital Surface | Hospital Surface | 2019 | Czech republic: Brno |
| FI465 | Rectal swab | Homo sapiens | Colonization | 2019 | Czech republic: Brno |
| FI466 | Stool | Homo sapiens | Colonization | 2019 | Czech republic: Brno |
| FI467 | Rectal swab | Homo sapiens | Colonization | 2019 | Czech republic: Brno |
| FI468 | Rectal swab | Homo sapiens | Colonization | 2019 | Czech republic: Brno |
| FI469 | Rectal swab | Homo sapiens | Colonization | 2019 | Czech republic: Brno |
| FI47 | Rectal swab | Homo sapiens | Colonization | 2017 | Czech republic: Brno |
| FI470 | Stool | Homo sapiens | Colonization | 2019 | Czech republic: Brno |
| FI471 | Rectal swab | Homo sapiens | Colonization | 2019 | Czech republic: Brno |
| FI472 | Rectal swab | Homo sapiens | Colonization | 2019 | Czech republic: Brno |
| FI473 | Rectal swab | Homo sapiens | Colonization | 2019 | Czech republic: Brno |
| FI474 | Rectal swab | Homo sapiens | Colonization | 2019 | Czech republic: Brno |
| FI475 | Rectal swab | Homo sapiens | Colonization | 2019 | Czech republic: Brno |
| FI476 | Rectal swab | Homo sapiens | Colonization | 2019 | Czech republic: Brno |
| FI477 | Rectal swab | Homo sapiens | Colonization | 2019 | Czech republic: Brno |
| FI478 | Stool | Homo sapiens | Colonization | 2019 | Czech republic: Brno |
| FI479 | Rectal swab | Homo sapiens | Colonization | 2019 | Czech republic: Brno |
| FI48 | Rectal swab | Homo sapiens | Colonization | 2017 | Czech republic: Brno |
| FI480 | Rectal swab | Homo sapiens | Colonization | 2019 | Czech republic: Brno |
| FI481 | Rectal swab | Homo sapiens | Colonization | 2019 | Czech republic: Brno |
| FI482 | Rectal swab | Homo sapiens | Colonization | 2019 | Czech republic: Brno |
| FI483 | Rectal swab | Homo sapiens | Colonization | 2019 | Czech republic: Brno |
| FI484 | Urine | Homo sapiens | Bacteriuria | 2019 | Czech republic: Brno |
| FI485 | Rectal swab | Homo sapiens | Colonization | 2019 | Czech republic: Brno |

| | | | | | |
|---|---|---|---|---|---|
| FI486 | Rectal swab | Homo sapiens | Colonization | 2019 | Czech republic: Brno |
| FI487 | Rectal swab | Homo sapiens | Colonization | 2019 | Czech republic: Brno |
| FI488 | Rectal swab | Homo sapiens | Colonization | 2019 | Czech republic: Brno |
| FI489 | Respiratory tract secretion | Homo sapiens | Colonization | 2019 | Czech republic: Brno |
| FI49 | Rectal swab | Homo sapiens | Colonization | 2017 | Czech republic: Brno |
| FI490 | Rectal swab | Homo sapiens | Colonization | 2019 | Czech republic: Brno |
| FI492 | Stool | Homo sapiens | Colonization | 2019 | Czech republic: Brno |
| FI494 | Urine | Homo sapiens | Bacteriuria | 2019 | Czech republic: Brno |
| FI495 | Rectal swab | Homo sapiens | Colonization | 2019 | Czech republic: Brno |
| FI497 | Rectal swab | Homo sapiens | Colonization | 2019 | Czech republic: Brno |
| FI498 | Urine | Homo sapiens | Bacteriuria | 2019 | Czech republic: Brno |
| FI50 | Stool | Homo sapiens | Colonization | 2017 | Czech republic: Brno |
| FI500 | Rectal swab | Homo sapiens | Colonization | 2019 | Czech republic: Brno |
| FI501 | Rectal swab | Homo sapiens | Colonization | 2019 | Czech republic: Brno |
| FI502 | Rectal swab | Homo sapiens | Colonization | 2019 | Czech republic: Brno |
| FI506 | Rectal swab | Homo sapiens | Colonization | 2019 | Czech republic: Brno |
| FI507 | Catheter | Homo sapiens | Colonization | 2019 | Czech republic: Brno |
| FI51 | Rectal swab | Homo sapiens | Colonization | 2017 | Czech republic: Brno |
| FI510 | Rectal swab | Homo sapiens | Colonization | 2020 | Czech republic: Brno |
| FI511 | Oral cavity | Homo sapiens | Colonization | 2020 | Czech republic: Brno |
| FI514 | Rectal swab | Homo sapiens | Colonization | 2022 | Czech republic: Brno |
| FI515 | Urine | Homo sapiens | Bacteriuria | 2022 | Czech republic: Brno |
| FI53 | Stool | Homo sapiens | Colonization | 2017 | Czech republic: Brno |
| FI54 | Urine | Homo sapiens | Bacteriuria | 2017 | Czech republic: Brno |
| FI55 | Rectal swab | Homo sapiens | Colonization | 2017 | Czech republic: Brno |
| FI57 | Rectal swab | Homo sapiens | Colonization | 2017 | Czech republic: Brno |
| FI58 | Rectal swab | Homo sapiens | Colonization | 2017 | Czech republic: Brno |
| FI59 | Rectal swab | Homo sapiens | Colonization | 2017 | Czech republic: Brno |
| FI60 | Rectal swab | Homo sapiens | Colonization | 2017 | Czech republic: Brno |
| FI61 | Rectal swab | Homo sapiens | Colonization | 2017 | Czech republic: Brno |
| FI62 | Rectal swab | Homo sapiens | Colonization | 2017 | Czech republic: Brno |
| FI63 | Rectal swab | Homo sapiens | Colonization | 2017 | Czech republic: Brno |
| FI64 | Rectal swab | Homo sapiens | Colonization | 2017 | Czech republic: Brno |
| FI65 | Rectal swab | Homo sapiens | Colonization | 2017 | Czech republic: Brno |
| FI66 | Rectal swab | Homo sapiens | Colonization | 2017 | Czech republic: Brno |
| FI67 | Rectal swab | Homo sapiens | Colonization | 2017 | Czech republic: Brno |
| FI68 | Rectal swab | Homo sapiens | Colonization | 2017 | Czech republic: Brno |
| FI69 | Rectal swab | Homo sapiens | Colonization | 2017 | Czech republic: Brno |
| FI70 | Rectal swab | Homo sapiens | Colonization | 2017 | Czech republic: Brno |
| FI71 | Rectal swab | Homo sapiens | Colonization | 2017 | Czech republic: Brno |
| FI73 | Rectal swab | Homo sapiens | Colonization | 2017 | Czech republic: Brno |
| FI74 | Rectal swab | Homo sapiens | Colonization | 2017 | Czech republic: Brno |
| FI75 | Rectal swab | Homo sapiens | Colonization | 2017 | Czech republic: Brno |
| FI76 | Rectal swab | Homo sapiens | Colonization | 2017 | Czech republic: Brno |
| FI77 | Stool | Homo sapiens | Colonization | 2017 | Czech republic: Brno |
| FI78 | Rectal swab | Homo sapiens | Colonization | 2017 | Czech republic: Brno |
| FI79 | Rectal swab | Homo sapiens | Colonization | 2017 | Czech republic: Brno |
| FI80 | Rectal swab | Homo sapiens | Colonization | 2017 | Czech republic: Brno |
| FI82 | Rectal swab | Homo sapiens | Colonization | 2017 | Czech republic: Brno |
| FI83 | Urine | Homo sapiens | Bacteriuria | 2017 | Czech republic: Brno |
| FI84 | Stool | Homo sapiens | Colonization | 2017 | Czech republic: Brno |
| FI86 | Rectal swab | Homo sapiens | Colonization | 2017 | Czech republic: Brno |
| FI87 | Rectal swab | Homo sapiens | Colonization | 2017 | Czech republic: Brno |
| FI88 | Rectal swab | Homo sapiens | Colonization | 2017 | Czech republic: Brno |
| FI89 | Rectal swab | Homo sapiens | Colonization | 2017 | Czech republic: Brno |
| FI90 | Stool | Homo sapiens | Colonization | 2017 | Czech republic: Brno |
| FI91 | Urine | Homo sapiens | Bacteriuria | 2017 | Czech republic: Brno |
| FI92 | Stool | Homo sapiens | Colonization | 2017 | Czech republic: Brno |
| FI93 | Stool | Homo sapiens | Colonization | 2017 | Czech republic: Brno |
| FI94 | Urine | Homo sapiens | Bacteriuria | 2017 | Czech republic: Brno |
| FI95 | Urinal catheter | Homo sapiens | Bacteriuria | 2017 | Czech republic: Brno |
| FI98 | Rectal swab | Homo sapiens | Colonization | 2017 | Czech republic: Brno |
| FI99 | Hospital Surface | Hospital Surface | Hospital Surface | 2017 | Czech republic: Brno |
| FIHEM15-6-19 | Blood | Homo sapiens | Infection | 2019 | Czech republic: Brno |
