# Supplementary material for: New Multilocus Sequence Typing Scheme for Enterococcus faecium Based on Whole Genome Sequencing Data
Source: Microbiol Spectr. 2023 Jun 12;11(4):e05107-22. doi: 10.1128/spectrum.05107-22 (PMC10434285; doi:10.1128/spectrum.05107-22)
Supplement: Supplemental file 1 — Supplemental material. Download spectrum.05107-22-s0001.pdf, PDF file, 14.4 MB [file spectrum.05107-22-s0001.pdf]

Fig. S1 Whole genome MLST based UPGMA-Tree and comparison of original ST and original Clonal complexes, new ST, new Clonal Complexes, and cgMLST clusters.

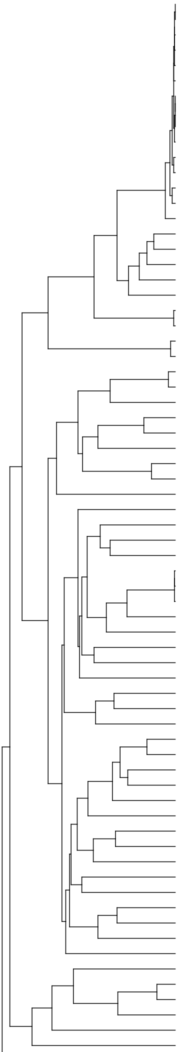

ID	ST original MLST	Clonal complex original MLST	ST new MLST	Clonal complex new MLST	Complex type core genome MLST	Collection Date	Collection location	solation source
GCF-001543555.1	25	CC17	551		729	2013	USA: Rhode Island	Laboratory strain
GCF-001543635.1	25	CC17	551		729	2013	USA: Rhode Island	Laboratory strain
GCF-001543795.1	25	CC17	551		729	2013	USA: Rhode Island	Laboratory strain
GCF-001543735.1	25	CC17	551		729	2013	USA: Rhode Island	Laboratory strain
GCF-001543585.1	25	CC17	551		729	2013	USA: Rhode Island	Laboratory strain
GCF-001543645.1	25	CC17	551		729	2013	USA: Rhode Island	Laboratory strain
GCF-001543565.1	25	CC17	551		729	2013	USA: Rhode Island	Laboratory strain
GCF-001543745.1	25	CC17	551		729	2013	USA: Rhode Island	Laboratory strain
GCF-001543575.1	25	CC17	551		729	2013	USA: Rhode Island	Laboratory strain
GCF-001582095.1	25	CC17	551		729	1984	France	n/a
GCF-001543665.1	25	CC17	551		729	2013	USA: Rhode Island	Laboratory strain
GCF-001543675.1	25	CC17	551		729	2013	USA: Rhode Island	Laboratory strain
GCF-001543835.1	25	CC17	551		4949	2013	USA: Rhode Island	Laboratory strain
GCF-001543715.1	25	CC17	551		4949	2013	USA: Rhode Island	Laboratory strain
GCF-001543765.1	551	CC17	551		4950	2013	USA: Rhode Island	Laboratory strain
GCF-019175525.1	25	CC17	70		6790	2020	Switzerland: Zug	Stream surface water
GCF-000394415.1	25	CC17	70		112	1986	n/a	n/a
GCF-000392315.1	79		582		736	1994	n/a	n/a
GCF-000394535.1	2296	CC17	546		784	1994	France: Nimes	n/a
GCF-000394435.1	25	CC17	596		778	1986	n/a	n/a
GCF-000321685.1	26	CC17	563		491	n/a	France	Blood
GCF-000396825.1	26	CC17	563		491	1994	n/a	n/a
GCF-000479165.1	281		549		84	1996	Brazil	Blood
GCF-000322105.1	210		549		494	2001	Netherlands	Urine
GCF-028335245.1	885		900	CC73	406	2021	n/a	Stool
GCF-026947605.1	885		900	CC73	406	2015	Belgium: Gilly	Blood
GCF-002831505.1	30		215	CC73	1745	2017	China: Chendu, Sichuan	Clinical isolate
GCF-003320415.1	2044		719		4901	n/a	USA: New York	Breast meat
GCF-000394475.1	640		597	CC73	786	1989	France: Caen	n/a
GCF-003320405.1	2319		718	CC73	4900	n/a	USA: Kansas City	Breast meat
GCF-025399665.1	190		82	CC73		2021	Bangladesh	Mastitis milk
GCF-019175445.1	2036		82	CC73	6788	2020	Switzerland: Cazis	Stream surface water
GCF-023182835.2	56	CC17	1040			2018	China: Xinjiang	n/a
GCF-005236155.1	9		204	CC73	6473	2003	New Zealand	Poultry feces
GCF-000395965.1	245		591	CC73	769	1997	Denmark	n/a
GCF-005238445.1	2249		83		4914	2001	New Zealand	Poultry feces
GCF-005237065.1	2279	CC17	75	CC73	6447	2001	New Zealand	Poultry feces
GCF-900640265.1	310		73	CC73	649	n/a	n/a	n/a
GCF-000322325.1	310		73	CC73	649	2004	Sweden	n/a
GCF-900638775.1	310		73	CC73	649	n/a	n/a	n/a
GCF-002140555.1	195		460	CC73	1623	n/a	Germany	Stool
GCF-005237055.1	195		74	CC73	4742	2001	New Zealand	Poultry feces
GCF-005234745.1	9		77	CC73	6472	2003	New Zealand	Poultry feces
GCF-000321465.1	9		560	CC73	667	1992	United Kingdom	Stool
GCF-027944535.1	158		1050	CC73		2022	China:Zhejiang	Sewage
GCF-000321925.1	67		569	CC73	514	1957	Netherlands	Blood
GCF-002141435.1	311		634		4997	n/a	Brazil	Stool
GCF-006741355.1	2256		232	CC73	4930	2013	Egypt:Kafr-El-Sheik governorate	n/a
GCF-003320805.1	12		724	CC73	4678	n/a	USA: Tampa	Breast meat
GCF-003320365.1	2318		717	CC73	4899	n/a	USA: New Orleans	Breast meat
GCF-003320325.1	12		716	CC73	4677	n/a	USA: Pennsylvania	Breast meat
GCF-003320235.1	309		714	CC73		n/a	USA: Louisville	Breast meat
GCF-003320655.1	9		677	CC73	6466	n/a	USA: Charlotte	Breast meat
GCF-003320155.1	8		711	CC73	6460	n/a	USA: Dallas	Breast meat
GCF-003320275.1	157		715	CC73	4726	n/a	USA: Little Rock	Breast meat
GCF-003320075.1	462		710	CC73	4826	n/a	USA: Boise	Breast meat
GCF-000321805.1	158		557		655	1995	Belgium	n/a
GCF-000395585.1	9		584	CC73	783	1994	France: Hopital Saint-Louis	n/a
GCF-005237545.1	2248		84	CC73	4927	2003	New Zealand	Poultry feces
GCF-003320355.1	9		1044	CC73		n/a	USA: San Francisco	Breast meat
GCF-000321525.1	9		567	CC73	652	1996	Netherlands	Stool
GCF-000322185.1	12		573		621	2004	Netherlands	n/a
GCF-026309215.1	195		848	CC73		2012	China:Liaoning province	Bile
GCF-018516845.1	1676		87	CC89	6784	2018	France: Bordeaux	Digestive
GCF-023299805.1	1846		76	CC73	6805	2022	n/a	Pet food
GCF-023299745.1	264		76	CC73	374	2022	n/a	Pet food
GCF-023375385.1	1767		216	CC89	6810	2021	n/a	Stool
GCF-000411655.2	673	CC17	28	CC184	6776	2011	Italy	Fermented sausage
GCF-025916235.1	1991		1038			2020	China: Jiangsu	Pig

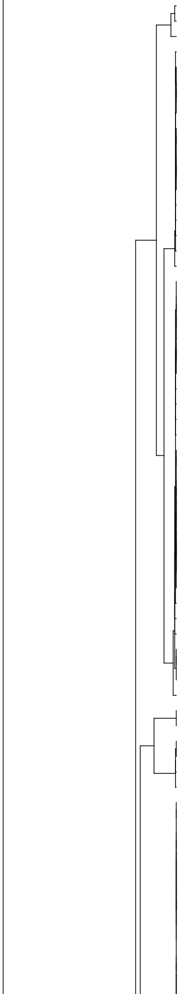

GCF-022463615.1	772	CC17	116	CC2	6894	2017	Israel: Jerusalem	Blood
GCF-022463435.1	772	CC17	116	CC2	6894	2018	Israel: Jerusalem	Blood
GCF-022463635.1	772	CC17	116	CC2	6896	2017	Israel: Jerusalem	Blood
GCF-022464585.1	772	CC17	156	CC128	6893	2017	Israel: Jerusalem	Blood
GCF-022464455.1	772	CC17	156	CC128	6893	2014	Israel: Jerusalem	Blood
GCF-022464655.1	772	CC17	156	CC128	6893	2017	Israel: Jerusalem	Blood
GCF-022469115.1	772	CC17	156	CC128	6893	2017	Israel: Jerusalem	Blood
GCF-022464635.1	772	CC17	156	CC128	6893	2017	Israel: Jerusalem	Blood
GCF-022464845.1	772	CC17	156	CC128	6893	2015	Israel: Jerusalem	Blood
GCF-022468335.1	772	CC17	156	CC128	6893	2017	Israel: Jerusalem	Blood
GCF-022468175.1	772	CC17	156	CC128	6893	2016	Israel: Jerusalem	Blood
GCF-022468155.1	772	CC17	156	CC128	6893	2016	Israel: Jerusalem	Blood
GCF-022469155.1	772	CC17	156	CC128	6893	2015	Israel: Jerusalem	Blood
GCF-022463415.1	772	CC17	156	CC128	6893	2018	Israel: Jerusalem	Blood
GCF-022463535.1	772	CC17	156	CC128	6893	2018	Israel: Jerusalem	Blood
GCF-022467875.1	772	CC17	156	CC128	6893	2016	Israel: Jerusalem	Blood
GCF-022469195.1	772	CC17	156	CC128	6893	2015	Israel: Jerusalem	Blood
GCF-022464775.1	772	CC17	156	CC128	6893	2016	Israel: Jerusalem	Blood
GCF-022463525.1	772	CC17	157	CC152	6890	2018	Israel: Jerusalem	Blood
GCF-022463675.1	772	CC17	157	CC152	6890	2016	Israel: Jerusalem	Blood
GCF-022464435.1	772	CC17	157	CC152	6890	2014	Israel: Jerusalem	Blood
GCF-022464375.1	772	CC17	157	CC152	6890	2014	Israel: Jerusalem	Blood
GCF-022463495.1	772	CC17	157	CC152	6890	2018	Israel: Jerusalem	Blood
GCF-022463475.1	772	CC17	157	CC152	6890	2018	Israel: Jerusalem	Blood
GCF-022464275.1	772	CC17	157	CC152	6890	2014	Israel: Jerusalem	Blood
GCF-022464355.1	772	CC17	157	CC152	6890	2016	Israel: Jerusalem	Blood
GCF-022463195.1	772	CC17	157	CC152	6890	2019	Israel: Jerusalem	Blood
GCF-022463945.1	772	CC17	157	CC152	6890	2012	Israel: Jerusalem	Blood
GCF-022464135.1	2277	CC17	157	CC152	6890	2014	Israel: Jerusalem	Blood
GCF-022462905.1	772	CC17	157	CC152	6890	2019	Israel: Jerusalem	Blood
GCF-022462935.1	772	CC17	157	CC152	6890	2019	Israel: Jerusalem	Blood
GCF-022463395.1	772	CC17	157	CC152	6890	2019	Israel: Jerusalem	Blood
GCF-022463335.1	772	CC17	157	CC152	6890	2019	Israel: Jerusalem	Blood
GCF-022463375.1	772	CC17	157	CC152	6890	2019	Israel: Jerusalem	Blood
GCF-022463035.1	772	CC17	157	CC152	6890	2019	Israel: Jerusalem	Blood
GCF-022463045.1	772	CC17	157	CC152	6890	2019	Israel: Jerusalem	Blood
GCF-022462995.1	772	CC17	157	CC152	6890	2019	Israel: Jerusalem	Blood
GCF-022462955.1	772	CC17	157	CC152	6890	2019	Israel: Jerusalem	Blood
GCF-022463135.1	772	CC17	157	CC152	6890	2019	Israel: Jerusalem	Blood
GCF-022463205.1	772	CC17	157	CC152	6890	2020	Israel: Jerusalem	Blood
GCF-022462975.1	772	CC17	157	CC152	6890	2020	Israel: Jerusalem	Blood
GCF-022467995.1	772	CC17	157	CC152	6890	2016	Israel: Jerusalem	Blood
GCF-022463315.1	772	CC17	157	CC152	6890	2020	Israel: Jerusalem	Blood
GCF-022463175.1	772	CC17	157	CC152	6890	2020	Israel: Jerusalem	Blood
GCF-022463255.1	772	CC17	157	CC152	6890	2020	Israel: Jerusalem	Blood
GCF-022463895.1	772	CC17	157	CC152	6890	2012	Israel: Jerusalem	Blood
GCF-022463735.1	17	CC17	49	CC7	6897	2017	Israel: Jerusalem	Blood
GCF-022463685.1	17	CC17	49	CC7	6897	2017	Israel: Jerusalem	Blood
GCF-022468495.1	80	CC17	151	CC152	6915	2017	Israel: Jerusalem	Blood
GCF-022469035.1	80	CC17	151	CC152	6915	2018	Israel: Jerusalem	Blood
GCF-022468505.1	80	CC17	151	CC152	6915	2017	Israel: Jerusalem	Blood
GCF-022468815.1	80	CC17	151	CC152	6915	2018	Israel: Jerusalem	Blood
GCF-004300435.1	80	CC17	152	CC152	3233	2018	USA:Little Rock	Blood
GCF-004300165.1	80	CC17	152	CC152	3233	2018	USA:Little Rock	Blood
GCF-004300655.1	80	CC17	152	CC152	16	2018	USA:Little Rock	Blood
GCF-004300705.1	80	CC17	152	CC152	16	2018	USA:Little Rock	Blood
GCF-004300725.1	80	CC17	152	CC152	16	2018	USA:Little Rock	Blood
GCF-004300755.1	80	CC17	152	CC152	16	2018	USA:Little Rock	Blood
GCF-005886545.1	80	CC17	152	CC152	16	2018	USA:Arkansas	n/a
GCF-005886655.1	80	CC17	152	CC152	16	2018	USA:Arkansas	n/a
GCF-004300805.1	80	CC17	152	CC152	16	2018	USA:Little Rock	Blood
GCF-004300645.1	80	CC17	152	CC152	3233	2018	USA:Little Rock	Blood
GCF-004300125.1	80	CC17	152	CC152	3233	2018	USA:Little Rock	Blood
GCF-004300635.1	80	CC17	152	CC152	3233	2018	USA:Little Rock	Blood
GCF-004300715.1	80	CC17	152	CC152	3233	2018	USA:Little Rock	Blood

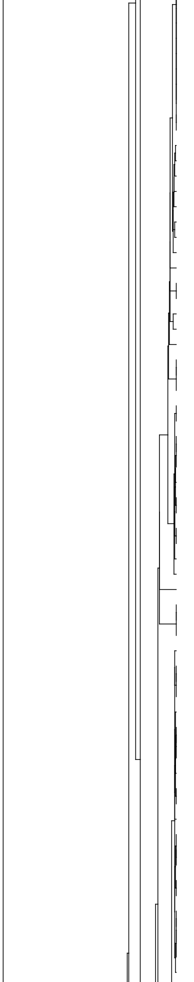

GCF-004300775.1	80	CC17	152	CC152	3233	2018	USA:Little Rock	Blood
GCF-004300285.1	80	CC17	152	CC152	3233	2018	USA:Little Rock	Blood
GCF-004300175.1	80	CC17	152	CC152	3233	2018	USA:Little Rock	Blood
GCF-005886715.1	80	CC17	152	CC152	3233	2018	USA:Arkansas	n/a
GCF-004300465.1	80	CC17	152	CC152	3233	2018	USA:Little Rock	Blood
GCF-004300555.1	80	CC17	152	CC152	3233	2018	USA:Little Rock	Blood
GCF-004300605.1	80	CC17	152	CC152	3233	2018	USA:Little Rock	Blood
GCF-004300075.1	80	CC17	152	CC152	3233	2018	USA:Little Rock	Blood
GCF-005886735.1	80	CC17	152	CC152	3233	2018	USA:Arkansas	n/a
GCF-022463755.1	80	CC17	152	CC152	16	2017	Israel: Jerusalem	Blood
GCF-900639725.1	80	CC17	152	CC152	16	2015	Netherlands	n/a
GCF-002997345.1	80	CC17	152	CC152	16	2013	France: Creteil	Rectal swab
GCF-900639585.1	80	CC17	152	CC152	16	2013	Netherlands	n/a
GCF-022469095.1	80	CC17	152	CC152	16	2016	Israel: Jerusalem	Blood
GCF-026941365.1	80	CC17	152	CC152	16	2017	Belgium: Veurne	Stool
GCF-026942235.1	80	CC17	152	CC152	16	2017	Belgium: Veurne	Stool
GCF-004300275.1	80	CC17	152	CC152	16	2018	USA:Little Rock	Blood
GCF-026941435.1	80	CC17	152	CC152	1840	2017	Belgium: Brussels	Stool
GCF-004152085.1	2181	CC17	152	CC152	16	2018	USA: St. Louis	Clinical isolate
GCF-004152475.1	2181	CC17	152	CC152	16	2018	USA: St. Louis	Clinical isolate
GCF-004151885.1	80	CC17	152	CC152	16	2018	USA: St. Louis	Hospital Surface
GCF-004151865.1	80	CC17	152	CC152	3418	2018	USA: St. Louis	Hospital Surface
GCF-016406485.1	80	CC17	152	CC152	5785	2015	USA: Dallas	Rectal swab
GCF-900180275.1	1454	CC17	152	CC152	5036	2015	United Kingdom	Human
GCF-900178685.1	1454	CC17	152	CC152	5036	2015	United Kingdom	Human
GCF-900178805.1	1454	CC17	152	CC152	5036	2015	United Kingdom	Human
GCF-015771975.1	80	CC17	152	CC152	1053	2017	USA: Pennsylvania	Rectal swab
GCF-015770765.1	80	CC17	152	CC152	1053	2017	USA: Pennsylvania	Rectal swab
GCF-026941865.1	80	CC17	152	CC152	1053	2017	Belgium: Bouge	Stool
GCF-026941325.1	80	CC17	152	CC152	1053	2017	Belgium: Bouge	Stool
GCF-026941345.1	80	CC17	152	CC152	1053	2017	Belgium: Bouge	Stool
GCF-026942195.1	80	CC17	152	CC152	1053	2017	Belgium: Bouge	Stool
GCF-002140595.1	80	CC17	152	CC152	1053	n/a	Germany: Berlin	Stool
GCF-002141195.1	80	CC17	152	CC152	1053	2015	Germany	Stool
GCF-026944725.1	80	CC17	152	CC152	1053	2017	Belgium: Roeselare	Rectal swab
GCF-026944695.1	80	CC17	152	CC152	1053	2017	Belgium: Roeselare	Rectal swab
GCF-017642135.1	80	CC17	152	CC152	1053	2017	South Africa: KwaZulu-Natal	Peadiatric - unoccupied bed
GCF-026942055.1	80	CC17	152	CC152	1053	2017	Belgium: Bouge	Blood
GCF-026948255.1	80	CC17	152	CC152	1206	2015	Belgium: Mouscron	Blood
GCF-022463235.1	80	CC17	111	CC152	6892	2020	Israel: Jerusalem	Blood
GCF-022463355.1	80	CC17	111	CC152	6892	2020	Israel: Jerusalem	Blood
GCF-022463095.1	80	CC17	111	CC152	6892	2020	Israel: Jerusalem	Blood
GCF-022468625.1	80	CC17	152	CC152	899	2018	Israel: Jerusalem	Blood
GCF-022468215.1	80	CC17	152	CC152	899	2016	Israel: Jerusalem	Blood
GCF-022467855.1	80	CC17	152	CC152	899	2017	Israel: Jerusalem	Blood
GCF-022464575.1	80	CC17	152	CC152	899	2016	Israel: Jerusalem	Blood
GCF-022468415.1	80	CC17	152	CC152	899	2017	Israel: Jerusalem	Blood
GCF-022468135.1	80	CC17	152	CC152	899	2016	Israel: Jerusalem	Blood
GCF-022467945.1	80	CC17	152	CC152	899	2016	Israel: Jerusalem	Blood
GCF-022464415.1	80	CC17	152	CC152	899	2014	Israel: Jerusalem	Blood
GCF-022468015.1	80	CC17	152	CC152	899	2016	Israel: Jerusalem	Blood
GCF-022468255.1	80	CC17	152	CC152	899	2016	Israel: Jerusalem	Blood
GCF-022464115.1	80	CC17	152	CC152	899	2012	Israel: Jerusalem	Blood
GCF-022468035.1	80	CC17	152	CC152	899	2016	Israel: Jerusalem	Blood
GCF-022469295.1	80	CC17	152	CC152	899	2014	Israel: Jerusalem	Blood
GCF-022464905.1	80	CC17	152	CC152	899	2015	Israel: Jerusalem	Blood
GCF-022469235.1	80	CC17	152	CC152	899	2015	Israel: Jerusalem	Blood
GCF-022463775.1	80	CC17	152	CC152	899	2017	Israel: Jerusalem	Blood
GCF-022464835.1	80	CC17	152	CC152	899	2015	Israel: Jerusalem	Blood
GCF-022468385.1	80	CC17	152	CC152	899	2017	Israel: Jerusalem	Blood
GCF-022464895.1	80	CC17	152	CC152	899	2014	Israel: Jerusalem	Blood
GCF-022464875.1	80	CC17	152	CC152	899	2014	Israel: Jerusalem	Blood
GCF-022464295.1	80	CC17	152	CC152	899	2016	Israel: Jerusalem	Blood
GCF-022464815.1	80	CC17	152	CC152	899	2016	Israel: Jerusalem	Blood

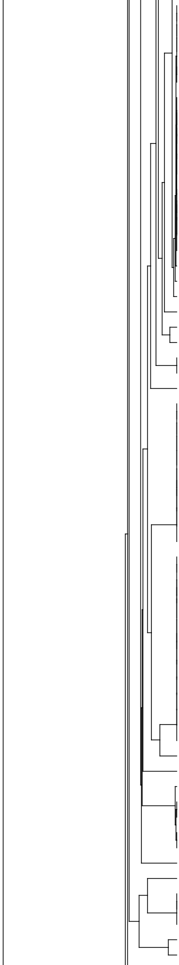

FI457	80	CC17	152	CC152	2680	2019	Czech republic: Brno	Rectal swab
FI453	80	CC17	152	CC152	2680	2019	Czech republic: Brno	Stool
FI492	80	CC17	152	CC152	2680	2019	Czech republic: Brno	Stool
FI494	80	CC17	152	CC152	2680	2019	Czech republic: Brno	Urine
FI510	80	CC17	152	CC152	2680	2020	Czech republic: Brno	Rectal swab
FI484	80	CC17	152	CC152	2680	2019	Czech republic: Brno	Urine
FI452	80	CC17	152	CC152	2680	2019	Czech republic: Brno	Urine
FI468	80	CC17	152	CC152	2680	2019	Czech republic: Brno	Rectal swab
FI455	80	CC17	152	CC152	2680	2019	Czech republic: Brno	Rectal swab
FI424	80	CC17	152	CC152	2680	2019	Czech republic: Brno	Blood
FI487	80	CC17	152	CC152	2680	2019	Czech republic: Brno	Rectal swab
FI440	80	CC17	152	CC152	2680	2019	Czech republic: Brno	Rectal swab
FI511	80	CC17	152	CC152	2680	2020	Czech republic: Brno	Oral cavity
FI480	80	CC17	152	CC152	2680	2019	Czech republic: Brno	Rectal swab
FI497	80	CC17	152	CC152	2680	2019	Czech republic: Brno	Rectal swab
FI482	80	CC17	152	CC152	2680	2019	Czech republic: Brno	Rectal swab
FI479	80	CC17	152	CC152	2680	2019	Czech republic: Brno	Rectal swab
FI495	80	CC17	152	CC152	2680	2019	Czech republic: Brno	Rectal swab
FI472	80	CC17	152	CC152	2680	2019	Czech republic: Brno	Rectal swab
FI458	80	CC17	26	CC152		2019	Czech republic: Brno	Rectal swab
GCF-022464515.1	80	CC17	137	CC152	6906	2015	Israel: Jerusalem	Blood
FI469	80	CC17	153	CC152	6771	2019	Czech republic: Brno	Rectal swab
FI474	80	CC17	153	CC152	6772	2019	Czech republic: Brno	Rectal swab
GCF-026945745.1	80	CC17	150	CC152	5196	2018	Belgium: Seraing	Rectal swab
GCF-026944685.1	80	CC17	150	CC152	5196	2017	Belgium: Brussels	Rectal swab
GCF-026943305.1	80	CC17	871	CC152	1618	2018	Belgium: Liege	Prothesis
GCF-900180265.1	80	CC17	143	CC152	3431	2015	United Kingdom	Human
GCF-900180285.1	80	CC17	143	CC152	3431	2015	United Kingdom	Human
GCF-900180295.1	80	CC17	143	CC152	3431	2015	United Kingdom	Human
GCF-900180305.1	80	CC17	143	CC152	3431	2015	United Kingdom	Human
GCF-900179885.1	80	CC17	143	CC152	3431	2015	United Kingdom	Human
GCF-900180075.1	80	CC17	143	CC152	3431	2015	United Kingdom	Human
GCF-900179995.1	80	CC17	143	CC152	3431	2015	United Kingdom	Human
GCF-900179425.1	80	CC17	143	CC152	3431	2015	United Kingdom	Human
GCF-900180085.1	80	CC17	143	CC152	3431	2015	United Kingdom	Human
GCF-900179765.1	80	CC17	143	CC152	3431	2015	United Kingdom	Human
GCF-026943895.1	612	CC17	1029	CC152	1026	2018	Belgium: Waregem	Clinical isolate
GCF-026946665.1	612	CC17	1029	CC152	1026	2018	Belgium: Waregem	Clinical isolate
GCF-026944485.1	612	CC17	1029	CC152	1026	2018	Belgium: Waregem	Rectal swab
GCF-026944645.1	612	CC17	1029	CC152	1026	2018	Belgium: Waregem	Rectal swab
GCF-026944215.1	612	CC17	1029	CC152	1026	2018	Belgium: Waregem	Rectal swab
GCF-026944605.1	612	CC17	1029	CC152	1026	2018	Belgium: Waregem	Rectal swab
GCF-026946745.1	612	CC17	1029	CC152	1026	2018	Belgium: Waregem	Rectal swab
GCF-026946645.1	612	CC17	1029	CC152	1026	2018	Belgium: Waregem	Rectal swab
GCF-026944565.1	612	CC17	1029	CC152	1026	2018	Belgium: Waregem	Rectal swab
GCF-026946805.1	612	CC17	1029	CC152	1026	2018	Belgium: Waregem	Clinical isolate
GCF-026944625.1	612	CC17	1029	CC152	1026	2018	Belgium: Waregem	Rectal swab
GCF-026944055.1	612	CC17	1029	CC152	1026	2018	Belgium: Waregem	Rectal swab
GCF-026943475.1	612	CC17	1029	CC152	1026	2018	Belgium: Waregem	Rectal swab
GCF-026944745.1	18	CC17	1024	CC152	6185	2017	Belgium: Ghent	Stool
GCF-005451945.1	80	CC17	142	CC152	3421	2018	China: Shenzhen	Stool
GCF-015768875.1	375	CC17	155	CC152	6837	2018	USA: Pennsylvania	Rectal swab
GCF-015771955.1	375	CC17	155	CC152	3719	2017	USA: Pennsylvania	Rectal swab
GCF-015772255.1	375	CC17	155	CC152	3719	2017	USA: Pennsylvania	Rectal swab
GCF-015771525.1	375	CC17	155	CC152	3719	2017	USA: Pennsylvania	Rectal swab
GCF-015771435.1	375	CC17	155	CC152	3719	2017	USA: Pennsylvania	Rectal swab
GCF-026944655.1	612	CC17	1027	CC152	2542	2017	Belgium: Ghent	Stool
GCF-026947465.1	80	CC17	838	CC152	1193	2016	Belgium: Namur	Blood
GCF-003938135.1	787	CC17	748	CC60	4849	2016	Pakistan	Light switch in hospital intensive care unit
GCF-003938005.1	787	CC17	748	CC60	4849	2016	Pakistan	Alcohol foam dispenser in hospital intensive care unit
GCF-003938525.1	787	CC17	748	CC60	4849	2016	Pakistan	Alcohol foam dispenser in hospital intensive care unit
GCF-900639555.1	80	CC17	144	CC152	106	2014	Netherlands	n/a
GCF-018516925.1	80	CC17	144	CC152	1476	2018	France: Corbeil-Essonnes	Rectal swab

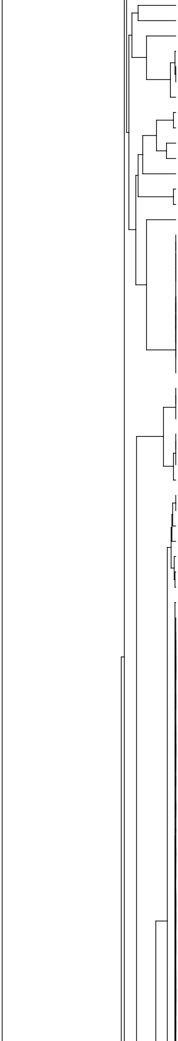

GCF-900639495.1	18	CC17	129	CC128	369	2012	Netherlands	n/a
GCF-000396885.1	18	CC17	593	CC128	773	1996	USA: New York	n/a
GCF-021122345.1	18	CC17	64	CC128	6882	2019	Belgium	Enviromental Sample
GCF-028335625.1	18	CC17	1019		235	2021	n/a	Stool
GCF-028335605.1	18	CC17	1019		235	2021	n/a	Stool
GCF-028335565.1	18	CC17	1019		235	2021	n/a	Stool
GCF-028335585.1	18	CC17	1019		289	2021	n/a	Stool
GCF-003350725.1	262	CC17	57	CC184		2008	USA: Utah	Blood
GCF-001990575.1	262	CC17	57	CC184	2646	2015	Chile	n/a
GCF-026202455.1	323	CC17	57	CC184		2021	China	Rectal swab
GCF-018517105.1	323	CC17	57	CC184	561	2016	France: Caen	Rectal swab
GCF-019356355.1	2254	CC17	250	CC184	6791	2018	China:Chongqing	Secretion
GCF-002562855.1	18	CC17	980	CC128	5025	2013	Russia: St.Petersburg	Skin
GCF-002562875.1	18	CC17	655	CC128	5025	2013	Russia: St.Petersburg	Skin
GCF-015768365.1	18	CC17	51	CC7	5674	2018	USA: Pennsylvania	Rectal swab
GCF-900178655.1	262	CC17	628	CC60	4994	2015	United Kingdom	Human
GCF-900179345.1	262	CC17	628	CC60	4994	2015	United Kingdom	Human
GCF-900178775.1	262	CC17	628	CC60	4994	2015	United Kingdom	Human
GCF-900180155.1	262	CC17	628	CC60	4994	2015	United Kingdom	Human
GCF-900178965.1	262	CC17	628	CC60	4994	2015	United Kingdom	Human
GCF-900179225.1	262	CC17	628	CC60	4994	2015	United Kingdom	Human
GCF-900179235.1	262	CC17	628	CC60	4994	2015	United Kingdom	Human
GCF-900179285.1	262	CC17	628	CC60	4994	2015	United Kingdom	Human
GCF-900178795.1	262	CC17	628	CC60	4994	2015	United Kingdom	Human
GCF-900179335.1	262	CC17	628	CC60	4994	2015	United Kingdom	Human
GCF-022463515.1	80	CC17	154	CC152	6895	2018	Israel: Jerusalem	Blood
GCF-022463595.1	80	CC17	154	CC152	6895	2017	Israel: Jerusalem	Blood
GCF-022463575.1	80	CC17	154	CC152	6895	2018	Israel: Jerusalem	Blood
GCF-017898005.1	80	CC17	139	CC152	1061	2019	India	Blood
GCF-017897965.1	80	CC17	139	CC152	1061	2019	India	Blood
GCF-012933195.2	80	CC17	139	CC152	1061	2019	India:Vellore	Blood
GCF-003020765.1	80	CC17	139	CC152	1061	2015	Australia	Blood
GCF-012933265.2	80	CC17	138		3453	2019	India:Vellore	Blood
GCF-017815675.1	80	CC17	138		3453	2019	India	Blood
GCF-015476295.1	80	CC17	138		3453	2014	Australia	Rectal swab
GCF-012933295.2	80	CC17	138		3455	2019	India:Vellore	Blood
GCF-015770155.1	80	CC17	138		5655	2017	USA: Pennsylvania	Rectal swab
GCF-015772535.1	80	CC17	138		3099	2017	USA: Pennsylvania	Rectal swab
GCF-015769025.1	80	CC17	138		3099	2018	USA: Pennsylvania	Rectal swab
FI199	80	CC17	140		6757	2018	Czech republic: Brno	Rectal swab
FI204	80	CC17	140		6757	2018	Czech republic: Brno	Rectal swab
FI113	80	CC17	140		6757	2017	Czech republic: Brno	Rectal swab
FI128	80	CC17	140		6757	2017	Czech republic: Brno	Rectal swab
FI121	80	CC17	140		6757	2017	Czech republic: Brno	Rectal swab
FI110	80	CC17	140		6757	2017	Czech republic: Brno	Rectal swab
FI129	80	CC17	140		6757	2017	Czech republic: Brno	Rectal swab
FI86	80	CC17	140		6757	2017	Czech republic: Brno	Rectal swab
FI244	80	CC17	140		6757	2018	Czech republic: Brno	Respiratory tract secretion
FI79	80	CC17	140		6757	2017	Czech republic: Brno	Rectal swab
FI131	80	CC17	140		6757	2017	Czech republic: Brno	Rectal swab
FI116	80	CC17	140		6757	2017	Czech republic: Brno	Rectal swab
FI132	80	CC17	140		6757	2017	Czech republic: Brno	Rectal swab
FI183	80	CC17	140		6757	2018	Czech republic: Brno	Rectal swab
FI114	80	CC17	140		6757	2017	Czech republic: Brno	Rectal swab
FI90	80	CC17	140		6757	2017	Czech republic: Brno	Stool
FI109	80	CC17	140		6757	2017	Czech republic: Brno	Rectal swab
FI43	80	CC17	140		6757	2017	Czech republic: Brno	Rectal swab
FI155	80	CC17	140		6757	2017	Czech republic: Brno	Rectal swab
FI130	80	CC17	140		6757	2017	Czech republic: Brno	Rectal swab
FI137	80	CC17	140		6757	2017	Czech republic: Brno	Rectal swab
FI135	80	CC17	140		6757	2017	Czech republic: Brno	Rectal swab
FI136	80	CC17	140		6757	2017	Czech republic: Brno	Rectal swab
FI149	80	CC17	140		6757	2017	Czech republic: Brno	Rectal swab
FI141	80	CC17	140		6757	2017	Czech republic: Brno	Oral cavity
FI65	80	CC17	140		6757	2017	Czech republic: Brno	Rectal swab
FI84	80	CC17	140		6757	2017	Czech republic: Brno	Stool
FI69	80	CC17	140		6757	2017	Czech republic: Brno	Rectal swab
FI127	80	CC17	140		6757	2017	Czech republic: Brno	Rectal swab

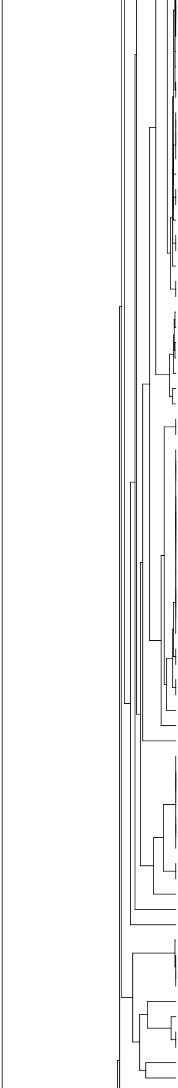

FI112	80	CC17	140		6757	2017	Czech republic: Brno	Rectal swab
FI88	80	CC17	140		6757	2017	Czech republic: Brno	Rectal swab
FI123	80	CC17	140		6757	2017	Czech republic: Brno	Oral cavity
FI101	80	CC17	140		6757	2017	Czech republic: Brno	Rectal swab
FI63	80	CC17	140		6757	2017	Czech republic: Brno	Rectal swab
FI122	80	CC17	140		6757	2017	Czech republic: Brno	Rectal swab
FI58	80	CC17	140		6757	2017	Czech republic: Brno	Rectal swab
FI175	80	CC17	140		6757	2018	Czech republic: Brno	Rectal swab
FI169	80	CC17	140		6757	2017	Czech republic: Brno	Urine
FI191	80	CC17	140		6757	2018	Czech republic: Brno	Abdomen cavity
FI95	80	CC17	140		6757	2017	Czech republic: Brno	Urinal catheter
FI144	80	CC17	140		6757	2017	Czech republic: Brno	Urine
FI184	80	CC17	140		6757	2018	Czech republic: Brno	Respiratory tract secretion
FI104	80	CC17	140		6757	2017	Czech republic: Brno	Rectal swab
FI91	80	CC17	140		6757	2017	Czech republic: Brno	Urine
FI449	80	CC17	140		6768	2019	Czech republic: Brno	Rectal swab
FI447	80	CC17	140		6768	2019	Czech republic: Brno	Rectal swab
FI478	80	CC17	140		6757	2019	Czech republic: Brno	Stool
FI53	80	CC17	140		6774	2017	Czech republic: Brno	Stool
FI73	80	CC17	140		6774	2017	Czech republic: Brno	Rectal swab
GCF-022464225.1	80	CC17	136	CC2	14	2014	Israel: Jerusalem	Blood
GCF-022467835.1	80	CC17	136	CC2	14	2016	Israel: Jerusalem	Blood
GCF-004302885.1	80	CC17	136	CC2	14	2013	Denmark: Copenhagen	Gastrointestinal tract
GCF-022469055.1	80	CC17	136	CC2	14	2018	Israel: Jerusalem	Blood
GCF-022468595.1	80	CC17	136	CC2	14	2017	Israel: Jerusalem	Blood
GCF-004151685.1	80	CC17	39	CC2	3416	2014	Pakistan: Islamabad	Clinical isolate
GCF-003938025.1	80	CC17	749	CC152	3411	2016	Pakistan	Nursing call button in hospital intensive care unit
FI55	80	CC17	174		6775	2017	Czech republic: Brno	Rectal swab
FI75	80	CC17	174		6775	2017	Czech republic: Brno	Rectal swab
FI166	80	CC17	173	CC162	6761	2017	Czech republic: Brno	Rectal swab
FI163	80	CC17	173	CC162	6761	2017	Czech republic: Brno	Rectal swab
FI156	80	CC17	173	CC162	6761	2017	Czech republic: Brno	Rectal swab
FI153	80	CC17	173	CC162	6761	2017	Czech republic: Brno	Rectal swab
FI164	80	CC17	173	CC162	6761	2017	Czech republic: Brno	Rectal swab
FI142	80	CC17	173	CC162	6761	2017	Czech republic: Brno	Rectal swab
FI150	80	CC17	173	CC162	6761	2017	Czech republic: Brno	Rectal swab
FI165	80	CC17	173	CC162	6761	2017	Czech republic: Brno	Rectal swab
FI138	80	CC17	173	CC162	6761	2017	Czech republic: Brno	Rectal swab
FI47	80	CC17	173	CC162	6761	2017	Czech republic: Brno	Rectal swab
FI50	80	CC17	173	CC162	6761	2017	Czech republic: Brno	Stool
FI49	80	CC17	173	CC162	6761	2017	Czech republic: Brno	Rectal swab
FI151	80	CC17	173	CC162	6761	2017	Czech republic: Brno	Wound
FI278	80	CC17	173	CC162	6761	2018	Czech republic: Brno	Wound
FI286	80	CC17	173	CC162	6761	2018	Czech republic: Brno	Stool
FI171	80	CC17	173	CC162	6761	2017	Czech republic: Brno	Rectal swab
FI176	80	CC17	173	CC162	6761	2018	Czech republic: Brno	Rectal swab
FI196	80	CC17	173	CC162	6763	2018	Czech republic: Brno	Rectal swab
FI134	80	CC17	175	CC152	6760	2017	Czech republic: Brno	Rectal swab
GCF-005517315.1	80	CC17	107	CC162	3422	2019	India	Pus
GCF-900180205.1	80	CC17	482	CC184	3429	2015	United Kingdom	Human
GCF-900180245.1	80	CC17	482	CC184	3429	2015	United Kingdom	Human
GCF-900178635.1	80	CC17	482	CC184	3429	2015	United Kingdom	Human
GCF-900179385.1	80	CC17	482	CC184	3429	2015	United Kingdom	Human
GCF-900179445.1	80	CC17	482	CC184	3429	2015	United Kingdom	Human
GCF-900179175.1	80	CC17	482	CC184	3429	2015	United Kingdom	Human
GCF-900178645.1	80	CC17	482	CC184	3429	2015	United Kingdom	Human
GCF-900180035.1	80	CC17	482	CC184	3105	2015	United Kingdom	Human
GCF-900179975.1	80	CC17	482	CC184	3105	2015	United Kingdom	Human
GCF-008728455.1	787	CC17	65	CC62	1737	2017	Denmark:Copenhagen	Rectal swab
GCF-016406465.1	612	CC17	121	CC152	5784	2015	USA: Dallas	Rectal swab
FI428	2247	CC17	169	CC162	6764	2019	Czech republic: Brno	Rectal swab
GCF-005235345.1	17	CC17	67	CC162	4687	2008	New Zealand	Clinical isolate
GCF-005234975.1	17	CC17	67	CC162	4687	2008	New Zealand	Stool
GCF-005235475.1	17	CC17	67	CC162	4687	2008	New Zealand	Rectal swab
GCF-005234435.1	17	CC17	67	CC162	4687	2008	New Zealand	Rectal swab
GCF-900635415.1	17	CC17	160	CC2	279	n/a	n/a	n/a
GCF-900639415.1	17	CC17	162	CC162	306	n/a	n/a	n/a
GCF-900639335.1	17	CC17	161	CC162	312	n/a	n/a	n/a
GCF-900639385.1	17	CC17	161	CC162	312	n/a	n/a	n/a
FI459	17	CC17	104	CC2	3035	2019	Czech republic: Brno	Rectal swab
GCF-015999405.1	17	CC17	103	CC2	3761	2014	Australia	Pleural fluid

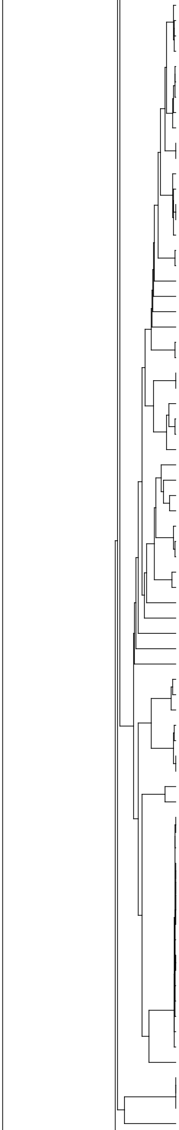

GCF-000295315.2	18	CC17	939	CC128	124	n/a	n/a	n/a
GCF-000295375.2	18	CC17	130	CC128	124	n/a	n/a	n/a
GCF-000295175.2	18	CC17	130	CC128	124	n/a	n/a	n/a
GCF-000295135.2	18	CC17	130	CC128	124	n/a	n/a	n/a
GCF-022468555.1	18	CC17	130	CC128	6903	2017	Israel: Jerusalem	Blood
GCF-022469135.1	18	CC17	130	CC128	6903	2011	Israel: Jerusalem	Blood
GCF-022464975.1	18	CC17	130	CC128	6903	2015	Israel: Jerusalem	Blood
GCF-022463875.1	18	CC17	130	CC128	6903	2018	Israel: Jerusalem	Blood
GCF-022469335.1	2275	CC17	130	CC128	6920	2014	Israel: Jerusalem	Blood
GCF-009846545.2	18	CC17	130	CC128	3435	n/a	Russia: Moscow	n/a
GCF-009846555.2	18	CC17	130	CC128	3435	n/a	Russia: Moscow	n/a
FI119	18	CC17	168	CC128	6759	2017	Czech republic: Brno	Rectal swab
FI168	18	CC17	168	CC128	6762	2017	Czech republic: Brno	Rectal swab
FI60	18	CC17	168	CC128	6762	2017	Czech republic: Brno	Rectal swab
FI76	18	CC17	168	CC128	6762	2017	Czech republic: Brno	Rectal swab
FI190	18	CC17	168	CC128	6759	2018	Czech republic: Brno	Rectal swab
GCF-022464675.1	80	CC17	130	CC128	6907	2016	Israel: Jerusalem	Blood
GCF-022467935.1	80	CC17	130	CC128	6907	2016	Israel: Jerusalem	Blood
GCF-022463815.1	78	CC17	126	CC128	6900	2017	Israel: Jerusalem	Blood
GCF-022469575.1	18	CC17	165	CC128	6928	2010	Israel: Jerusalem	Blood
GCF-000295495.2	18	CC17	937	CC128	780	n/a	n/a	n/a
GCF-022464755.1	18	CC17	22	CC128	6910	2016	Israel: Jerusalem	Blood
FI417	18	CC17	168	CC128		2019	Czech republic: Brno	Stool
FI439	1766	CC17	168	CC128		2019	Czech republic: Brno	Rectal swab
GCF-020162155.1	18	CC17	166	CC128	864	2015	Denmark	Clinical isolate
GCF-020162175.1	18	CC17	166	CC128	864	2015	Denmark	Clinical isolate
GCF-026073455.1	18	CC17	166	CC128		2011	Japan	n/a
GCF-900639355.1	18	CC17	166	CC128	173	n/a	n/a	n/a
GCF-900639365.1	18	CC17	166	CC128	317	n/a	n/a	n/a
GCF-016415485.1	262	CC17	78	CC128	5790	2015	USA: Dallas	Rectal swab
GCF-001720945.1	2272	CC17	134	CC128	5048	2011	USA:New York City	Blood
GCF-900639345.1	18	CC17	134	CC128	276	n/a	n/a	n/a
GCF-002562865.1	18	CC17	134	CC128	5024	2010	Russia: St.Petersburg	Burn wound
GCF-022464735.1	18	CC17	134	CC128	6909	2011	Israel: Jerusalem	Blood
GCF-000479145.1	18	CC17	127	CC128	222	2009	France: Nimes	Clinical isolate
GCF-000393755.1	18	CC17	595	CC128	222	2000	France: Hopital Pellegrin-Bordeaux	n/a
GCF-900638785.1	18	CC17	127	CC128	222	n/a	n/a	n/a
GCF-022469375.1	18	CC17	130	CC128	6912	2010	Israel: Jerusalem	Blood
GCF-022465015.1	18	CC17	130	CC128	6912	2010	Israel: Jerusalem	Blood
GCF-000294995.2	125	CC17	940	CC128	741	n/a	n/a	n/a
GCF-001055445.1	18	CC17	505	CC128	788	n/a	USA:Washington	Clinical isolate
GCF-002973795.1	18	CC17	657	CC128	4693	2013	France: Creteil	Rectal swab
FI488	18	CC17	131	CC128		2019	Czech republic: Brno	Rectal swab
GCF-022468535.1	132	CC17	113		6916	2017	Israel: Jerusalem	Blood
GCF-004299925.1	203	CC17	201		4780	2018	USA:Little Rock	Blood
GCF-004299995.1	203	CC17	201		4782	2018	USA:Little Rock	Blood
GCF-004300535.1	203	CC17	201		4783	2018	USA:Little Rock	Blood
GCF-016415345.1	1703	CC17	274		5788	2015	USA: Dallas	Rectal swab
GCF-016415445.1	1703	CC17	274		5788	2015	USA: Dallas	Rectal swab
GCF-016415545.1	1703	CC17	275		5791	2015	USA: Dallas	Rectal swab
GCF-016415565.1	1703	CC17	275		5788	2015	USA: Dallas	Rectal swab
GCF-019977575.1	17	CC17	100	CC7	6792	2014	Japan	Clinical isolate
GCF-009938285.1	17	CC17	101	CC7	1295	n/a	n/a	n/a
FI105	80	CC17	170	CC152	6758	2017	Czech republic: Brno	Rectal swab
FI61	80	CC17	170	CC152	6758	2017	Czech republic: Brno	Rectal swab
FI89	80	CC17	170	CC152	6758	2017	Czech republic: Brno	Rectal swab
FI162	80	CC17	170	CC152	6758	2017	Czech republic: Brno	Rectal swab
FI140	80	CC17	170	CC152	6758	2017	Czech republic: Brno	Rectal swab
FI139	80	CC17	170	CC152	6758	2017	Czech republic: Brno	Rectal swab
FI152	80	CC17	170	CC152	6758	2017	Czech republic: Brno	Swab
FI143	80	CC17	170	CC152	6758	2017	Czech republic: Brno	Stool
FI470	80	CC17	170	CC152	6758	2019	Czech republic: Brno	Stool
FI309	80	CC17	170	CC152	6758	2018	Czech republic: Brno	Rectal swab
FI360	80	CC17	170	CC152	6758	2018	Czech republic: Brno	Rectal swab
FI148	80	CC17	170	CC152	6758	2017	Czech republic: Brno	Rectal swab
FI433	80	CC17	170	CC152	6758	2019	Czech republic: Brno	Rectal swab
FI146	80	CC17	170	CC152	6758	2017	Czech republic: Brno	Urine
FI180	80	CC17	170	CC152	6758	2018	Czech republic: Brno	Urine
FI448	80	CC17	170	CC152	6758	2019	Czech republic: Brno	Rectal swab
GCF-002997315.1	17	CC17	167	CC162	4683	2013	France: Caen	Stool
GCF-003939745.1	612	CC17	147	CC152	2277	2016	Pakistan	Alcohol foam dispenser in hospital intensive care unit
GCF-003938035.1	612	CC17	147	CC152	2277	2016	Pakistan	Rail in hospital intensive care unit
GCF-003939765.1	612	CC17	147	CC152	2277	2016	Pakistan	Nursing call button in hospital intensive care unit
GCF-005576735.1	17	CC17	146	CC152	4688	2019	India	Pus

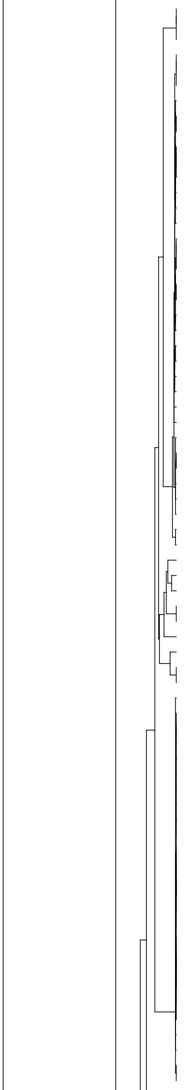

GCF-907177285.1	203	CC17	188	CC2	474	2018	Switzerland	Superficial wound
GCF-907176815.1	203	CC17	188	CC2	1028	2018	Switzerland	Superficial wound
GCF-907176135.1	203	CC17	188	CC2	474	2015	Switzerland	Superficial wound
GCF-026952595.1	203	CC17	188	CC2	20	2014	Belgium: Tournai	Blood
GCF-026928855.1	203	CC17	188	CC2	20	2016	Belgium: Tournai	Blood
GCF-026952485.1	203	CC17	188	CC2	20	2014	Belgium: Tournai	Blood
GCF-026952365.1	203	CC17	188	CC2	20	2014	Belgium: Assebroek	Blood
GCF-026952325.1	203	CC17	188	CC2	20	2014	Belgium: Leuven	Blood
GCF-026921235.1	203	CC17	188	CC2	20	2012	Belgium: Leuven	Blood
GCF-026952505.1	203	CC17	188	CC2	20	2014	Belgium: Edegem	Blood
GCF-026948505.1	203	CC17	188	CC2	20	2011	Belgium: Edegem	Blood
GCF-026923195.1	203	CC17	188	CC2	20	2016	Belgium: Edegem	Catheter
GCF-026950705.1	203	CC17	188	CC2	20	2012	Belgium: Turnhout	Blood
GCF-026952925.1	203	CC17	188	CC2	20	2016	Belgium: Leuven	Clinical isolate
GCF-026928645.1	203	CC17	188	CC2	20	2016	Belgium: Leuven	Tissue
GCF-026928635.1	203	CC17	188	CC2	20	2016	Belgium: Gilly	Blood
GCF-026928895.1	203	CC17	188	CC2	20	2015	Belgium: Gilly	Blood
GCF-026953255.1	203	CC17	188	CC2	20	2015	Belgium: Gilly	Blood
GCF-026928695.1	203	CC17	188	CC2	20	2016	Belgium: Gilly	Blood
GCF-026953125.1	203	CC17	188	CC2	20	2016	Belgium: Gilly	Blood
GCF-026929055.1	203	CC17	188	CC2	20	2015	Belgium: Gilly	Blood
GCF-026929035.1	203	CC17	188	CC2	20	2015	Belgium: Gilly	Blood
GCF-026952585.1	203	CC17	188	CC2	20	2013	Belgium: Ronse	Blood
GCF-003020745.1	203	CC17	188	CC2	20	2015	Australia	Blood
GCF-003719355.1	203	CC17	188	CC2	20	2015	China:Sichuan	Clinical isolate
GCF-900639575.1	203	CC17	188	CC2	20	2014	Netherlands	n/a
GCF-026953085.1	203	CC17	188	CC2	20	2016	Belgium: Gilly	Clinical isolate
GCF-026952765.1	203	CC17	188	CC2		2016	Belgium: Gilly	Clinical isolate
GCF-026950015.1	203	CC17	188	CC2	20	2017	Belgium: Tienen	Rectal swab
GCF-026950005.1	203	CC17	188	CC2	20	2017	Belgium: Tienen	Rectal swab
GCF-026950285.1	203	CC17	188	CC2	20	2017	Belgium: Tienen	Rectal swab
GCF-026950105.1	203	CC17	188	CC2	20	2017	Belgium: Tienen	Rectal swab
GCF-026950145.1	203	CC17	188	CC2	20	2017	Belgium: Tienen	Clinical isolate
GCF-026951315.1	203	CC17	188	CC2	20	2018	Belgium: Brussels	Stool
GCF-014489995.1	203	CC17	188	CC2	20	2016	Germany:Cologne	Clinical isolate
GCF-014490015.1	203	CC17	188	CC2	20	2016	Germany:Cologne	Clinical isolate
GCF-907163255.1	203	CC17	188	CC2	6814	2012	New Zealand	Blood
GCF-900639395.1	203	CC17	189		191	n/a	n/a	n/a
GCF-001518735.1	203	CC17	188	CC2	686	2014	USA	Stool
GCF-007917315.3	203	CC17	188	CC2	465	2014	Sweden:Halmstad	Stool
GCF-007917035.2	203	CC17	188	CC2	465	2014	Sweden:Halmstad	Stool
GCF-015325925.1	203	CC17	192	CC2	1243	2014	Australia	Rectal swab
GCF-019973595.1	203	CC17	188	CC2	1698	2019	Iran	Blood
GCF-003071425.1	203	CC17	188	CC2	859	2014	Denmark	Urine
GCF-003071445.1	203	CC17	188	CC2	859	2014	Denmark	Urine
GCF-022464315.1	78	CC17	29	CC2	2488	2014	Israel: Jerusalem	Blood
GCF-022464555.1	78	CC17	29	CC2	2488	2015	Israel: Jerusalem	Blood
GCF-022468095.1	78	CC17	29	CC2	2488	2016	Israel: Jerusalem	Blood
GCF-022463295.1	78	CC17	29	CC2	2488	2019	Israel: Jerusalem	Blood
GCF-022467895.1	78	CC17	29	CC2	2488	2016	Israel: Jerusalem	Blood
GCF-022467695.1	78	CC17	29	CC2	2488	2015	Israel: Jerusalem	Blood
GCF-022463005.1	78	CC17	29	CC2	2488	2019	Israel: Jerusalem	Blood
GCF-022464155.1	78	CC17	29	CC2	2488	2014	Israel: Jerusalem	Blood
GCF-022463155.1	78	CC17	29	CC2	2488	2019	Israel: Jerusalem	Blood
GCF-022464715.1	78	CC17	29	CC2	2488	2016	Israel: Jerusalem	Blood
GCF-022468265.1	78	CC17	29	CC2	2488	2017	Israel: Jerusalem	Blood
GCF-022468055.1	78	CC17	29	CC2	2488	2016	Israel: Jerusalem	Blood
GCF-022464325.1	78	CC17	29	CC2	2488	2014	Israel: Jerusalem	Blood
GCF-022464175.1	78	CC17	29	CC2	2488	2014	Israel: Jerusalem	Blood
GCF-022464195.1	78	CC17	29	CC2	2488	2014	Israel: Jerusalem	Blood
GCF-022467765.1	78	CC17	29	CC2	2488	2015	Israel: Jerusalem	Blood
GCF-022464535.1	78	CC17	29	CC2	2488	2015	Israel: Jerusalem	Blood
GCF-022467715.1	78	CC17	29	CC2	2488	2015	Israel: Jerusalem	Blood
GCF-022467755.1	78	CC17	29	CC2	2488	2015	Israel: Jerusalem	Blood
GCF-022464595.1	78	CC17	29	CC2	2488	2015	Israel: Jerusalem	Blood
GCF-022467795.1	78	CC17	29	CC2	2488	2015	Israel: Jerusalem	Blood
GCF-022464485.1	78	CC17	29	CC2	2488	2014	Israel: Jerusalem	Blood
GCF-022463995.1	78	CC17	29	CC2	2488	2013	Israel: Jerusalem	Blood
GCF-022463935.1	78	CC17	29	CC2	2488	2014	Israel: Jerusalem	Blood
GCF-022463915.1	78	CC17	29	CC2	2488	2014	Israel: Jerusalem	Blood
GCF-022463975.1	78	CC17	29	CC2	2488	2013	Israel: Jerusalem	Blood

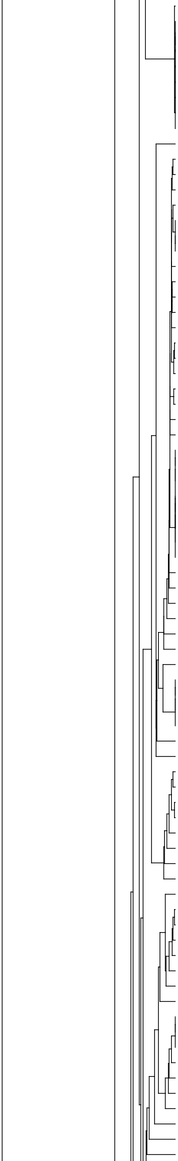

GCF-026953165.1	17	CC17	942	CC2	6180	2016	Belgium: Roeselare	Rectal swab
GCF-026929415.1	17	CC17	942	CC2	6180	2019	Belgium: Wilrijk	n/a
GCF-026953775.1	17	CC17	942	CC2	6180	2019	Belgium: Wilrijk	n/a
GCF-026951045.1	17	CC17	942	CC2	6180	2018	Belgium: Roeselare	Urine
GCF-026951145.1	17	CC17	942	CC2	6180	2018	Belgium: Roeselare	Stool
GCF-026950965.1	17	CC17	942	CC2	6180	2018	Belgium: Roeselare	Stool
GCF-026934765.1	17	CC17	942	CC2	6180	2017	Belgium: Roeselare	Rectal swab
GCF-026952885.1	17	CC17	942	CC2	6180	2016	Belgium: Roeselare	Urine
GCF-026953895.1	17	CC17	942	CC2	6180	2019	Belgium: Wilrijk	n/a
GCF-026945505.1	203	CC17	192	CC2	1194	2009	Belgium: Antwerpen	Blood
GCF-000295235.2	412	CC17	192	CC2	774	n/a	n/a	n/a
GCF-002158355.1	412	CC17	192	CC2	774	2007	USA: Utah	Blood
GCF-000295015.1	412	CC17	192	CC2	794	n/a	n/a	n/a
GCF-004300265.1	412	CC17	192	CC2	730	2018	USA:Little Rock	Blood
GCF-001059965.1	412	CC17	192	CC2	730	n/a	USA:Washington	Clinical isolate
GCF-001059755.1	412	CC17	192	CC2	730	n/a	USA:Washington	Clinical isolate
GCF-001059685.1	412	CC17	192	CC2	730	n/a	USA:Washington	Clinical isolate
GCF-002909305.1	412	CC17	192	CC2	774	2010	France: Clichy	Catheter
GCF-000395865.1	412	CC17	192	CC2	376	2010	Denmark	n/a
GCF-000295455.2	203	CC17	192	CC2	772	n/a	n/a	n/a
GCF-005235735.1	203	CC17	192	CC2	772	2005	New Zealand	Catheter
GCF-000157535.1	203	CC17	192	CC2	83	n/a	n/a	n/a
GCF-011386845.1	412	CC17	192	CC2	765	2005	Colombia: Bogota	Bronchial secretion
GCF-000295395.2	412	CC17	192	CC2	765	n/a	n/a	n/a
GCF-000295355.2	412	CC17	192	CC2	765	n/a	n/a	n/a
GCF-000294955.2	203	CC17	192	CC2	754	n/a	n/a	n/a
GCF-000294835.2	203	CC17	192	CC2	754	n/a	n/a	n/a
GCF-005235745.1	203	CC17	192	CC2	4323	2005	New Zealand	Urine
GCF-000295255.2	203	CC17	192	CC2	723	n/a	n/a	n/a
GCF-022509585.1	412	CC17	192	CC2	4809	2018	Pakistan: Islamabad	Dahi fermented milk product
GCF-008921725.1	412	CC17	192	CC2	4809	2012	Brazil	Urine
GCF-008921905.1	412	CC17	192	CC2	4809	2012	Brazil	Urine
GCF-008921735.1	412	CC17	192	CC2	4809	2012	Brazil	Urine
GCF-008921925.1	412	CC17	192	CC2	4809	2012	Brazil	Urine
GCF-008921825.1	412	CC17	192	CC2	4809	2012	Brazil	Urine
GCF-008921935.1	412	CC17	192	CC2	4809	2012	Brazil	Urine
GCF-008921845.1	412	CC17	192	CC2	4809	2012	Brazil	Urine
GCF-026945225.1	412	CC17	192	CC2	327	2010	Belgium: Leuven	Blood
GCF-000294915.2	412	CC17	192	CC2	734	n/a	n/a	n/a
GCF-003350705.1	412	CC17	192	CC2	4812	2007	USA: Utah	Blood
GCF-000295075.2	412	CC17	192	CC2	725	n/a	n/a	n/a
GCF-003350665.1	412	CC17	192	CC2	4810	2012	USA: Utah	Blood
GCF-026953615.1	203	CC17	192	CC2	1191	2019	Belgium: Wilrijk	n/a
GCF-026949405.1	203	CC17	192	CC2	1190	2012	Belgium: Liege	Blood
GCF-026934795.1	203	CC17	192	CC2	7090	2010	Denmark: Copenhagen	Clinical isolate
GCF-026929655.1	203	CC17	192	CC2	7090	2019	Belgium: Wilrijk	n/a
GCF-026929595.1	203	CC17	192	CC2	7090	2019	Belgium: Wilrijk	n/a
GCF-026934425.1	203	CC17	192	CC2	7090	2019	Belgium: Wilrijk	n/a
GCF-026073435.1	2060	CC17	192	CC2		2010	Japan	n/a
GCF-004152565.1	772	CC17	30	CC2	4848	2018	USA: St. Louis	Clinical isolate
GCF-000322405.1	78	CC17	208	CC2	86	2006	Netherlands	Blood
GCF-026945405.1	78	CC17	208	CC2	1196	2009	Belgium: Montignies-Le-Tilleul	Blood
GCF-006575625.1	549	CC17	208	CC2	86	2015	USA: Houston	Clinical isolate
GCF-000295595.2	549	CC17	208	CC2	86	n/a	n/a	n/a
GCF-002630975.1	78	CC17	208	CC2	5066	2012	Russia: St.Petersburg	Stool
GCF-026073915.1	78	CC17	208	CC2		2015	Japan	Urine
GCF-026073735.1	78	CC17	1033			2015	Japan	Urine
GCF-026073625.1	78	CC17	1031			2014	Japan	Urine
GCF-002334625.1	192	CC17	110	CC128	397	2008	Norway	Blood
GCF-026949325.1	192	CC17	52	CC2	10	2011	Belgium: Leuven	Blood
GCF-026949605.1	192	CC17	52	CC2	10	2008	Belgium: Huy	Blood
GCF-000395825.1	192	CC17	52	CC2	10	2010	Denmark	n/a
GCF-026949305.1	192	CC17	52	CC2		2013	Belgium: Huy	Urine
GCF-019780105.1	192	CC17	52	CC2	6878	2019	China	n/a
GCF-026949565.1	192	CC17	52	CC2	1198	2009	Belgium: Edegem	Blood
GCF-026929245.1	192	CC17	1010	CC2		2010	Denmark: Copenhagen	Clinical isolate
GCF-900639525.1	192	CC17	52	CC2	3	2013	Netherlands	n/a
GCF-900639515.1	192	CC17	52	CC2	3	2013	Netherlands	n/a
GCF-002442275.1	192	CC17	52	CC2	3	2012	Netherlands	Rectal swab
GCF-002442475.1	192	CC17	52	CC2	26	2012	Netherlands	Rectal swab
GCF-002442315.1	192	CC17	108	CC2	990	2012	Germany	Rectal swab
GCF-026949525.1	192	CC17	52	CC2	1246	2011	Belgium: Mons	Blood
GCF-021713095.1	192	CC17	56	CC2	6799	2018	Germany	n/a
GCF-009846615.2	192	CC17	52	CC2	3436	n/a	Russia: Moscow	n/a
GCF-023658035.1	192	CC17	52	CC2	6813	2018	China: Jinan	n/a
GCF-000951815.1	572	CC17	610		96	2013	China: Beijing	Bile

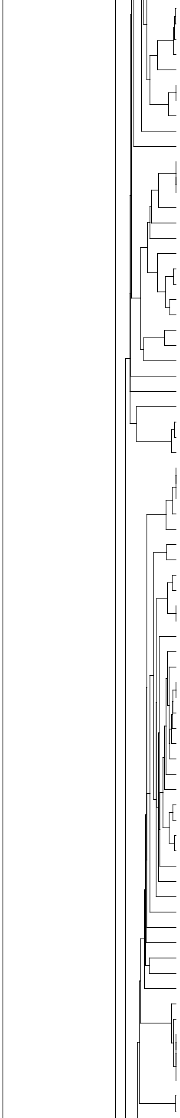

GCF-026309095.1	78	CC17	1020	CC184		2013	China:Liaoning province	Urine
GCF-026309035.1	78	CC17	1020	CC184		2013	China:Liaoning province	Urine
GCF-026308975.1	78	CC17	1020	CC184		2013	China:Liaoning province	Secretion
GCF-026304875.1	78	CC17	1020	CC184		2016	China:Liaoning province	Blood
GCF-026305175.1	363	CC17	1021	CC60		2015	China:Liaoning province	Urine
GCF-000322445.1	78	CC17	53	CC2	246	2010	Latvia	Clinical isolate
GCF-900639425.1	78	CC17	53	CC2	246	n/a	n/a	n/a
GCF-000322465.1	78	CC17	575		230	2010	Portugal	Clinical isolate
GCF-002630985.1	78	CC17	656	CC2	5065	2012	Russia: St.Petersburg	Wound
GCF-026949465.1	192	CC17	1023	CC128	1222	2012	Belgium: Gosselies	Blood
GCF-022699565.1	17	CC17	2	CC2	6793	2020	China: Shandong	Sanies
GCF-020091325.1	17	CC17	2	CC2	6793	2020	China:Shandong	Clinical isolate
GCF-022699545.1	17	CC17	2	CC2	6793	2020	China: Shandong	Urine
GCF-002024245.1	1421	CC17	2	CC2	5037	2014	South Korea	Rectal swab
GCF-026947505.1	17	CC17	2	CC2	2660	2016	Belgium: Brussels	Blood
GCF-025998395.1	117	CC17	1016	CC2		2021	Viet Nam:Hanoi	n/a
GCF-003020685.1	1421	CC17	14	CC2	1251	2015	Australia	Clinical isolate
GCF-002007625.1	1421	CC17	4	CC2	1471	2014	South Korea	Rectal swab
GCF-002025065.1	1421	CC17	4	CC2	1471	2014	Korea: republic of	Rectal swab
GCF-021172105.3	2096	CC17	4	CC2	6566	2021	South Korea: Daegu	n/a
GCF-002025045.1	1421	CC17	4	CC2	1747	2015	Korea: republic of	Rectal swab
GCF-003350815.1	584	CC17	48	CC2		2008	USA: Utah	Blood
GCF-003350635.1	969	CC17	48	CC2	4873	2009	USA: Utah	Blood
GCF-000395885.1	17	CC17	544	CC2	162	2010	Denmark	n/a
GCF-001990615.1	2300	CC17	620	CC128	5018	2015	Chile	n/a
GCF-022749455.1	192	CC17	54		6802	n/a	n/a	n/a
GCF-002562805.1	78	CC17	653	CC128	5064	2012	Russia: St.Petersburg	Blood
GCF-900092475.1	796	CC17	183	CC2	1219	2011	Australia	Clinical isolate
GCF-003020725.1	796	CC17	183	CC2	1219	2015	Australia	Blood
GCF-017584065.1	796	CC17	183	CC2	5874	2014	Australia	Rectal swab
GCF-026934565.1	78	CC17	576	CC2	1195	2005	Belgium: Gilly	Blood
GCF-026929425.1	78	CC17	576	CC2	1195	2019	Belgium: Wilrijk	n/a
GCF-026929355.1	78	CC17	576	CC2	1195	2019	Belgium: Wilrijk	n/a
GCF-000322365.1	78	CC17	576	CC2	4	2002	Germany	Clinical isolate
GCF-000322385.1	78	CC17	576	CC2	480	2005	Hungary	Clinical isolate
GCF-000295415.2	17	CC17	812	CC2	698	n/a	n/a	n/a
GCF-000295435.2	17	CC17	812	CC2	715	n/a	n/a	n/a
GCF-000479125.1	17	CC17	1	CC2	87	2004	Australia: Adelaide	Blood
GCF-000250945.1	17	CC17	1	CC2	1	1998	Australia: Melbourne	Blood
GCF-000397005.1	17	CC17	1	CC2	700	1996	n/a	n/a
GCF-000393695.1	17	CC17	1	CC2	700	1996	France: Hopital Toulouse	n/a
GCF-001721085.1	17	CC17	3	CC2	1095	2011	USA:New York City	Blood
GCF-000148025.1	17	CC17	2	CC2	699	n/a	n/a	n/a
GCF-000396945.1	17	CC17	2	CC2	708	1996	USA: New York	n/a
GCF-000395905.1	17	CC17	2	CC2	706	1996	Switzerland: Geneve	n/a
GCF-000395945.1	17	CC17	2	CC2	706	1996	Switzerland: Geneve	n/a
GCF-000395645.1	17	CC17	2	CC2	706	1994	France: Hopital Saint-Louis	n/a
GCF-000395665.1	17	CC17	2	CC2	711	1996	France: Centre Hospitalier Lyon-Sud	n/a
GCF-000393775.1	17	CC17	2	CC2	710	1992	n/a	n/a
GCF-000395545.1	17	CC17	2	CC2	705	1993	France: Hopital Saint-Louis	n/a
GCF-011386855.1	17	CC17	2	CC2	709	2001	Colombia: Bogota	Surgical wound
GCF-900044005.1	17	CC17	2	CC2	218	n/a	n/a	n/a
GCF-005234565.1	17	CC17	261	CC2	703	2004	New Zealand	Abdominal wound
GCF-000294855.2	17	CC17	261	CC2	703	n/a	n/a	n/a
GCF-000295215.2	17	CC17	2	CC2	701	n/a	n/a	n/a
GCF-000295195.2	17	CC17	2	CC2	701	n/a	n/a	n/a
GCF-026073415.1	17	CC17	2	CC2		n/a	Japan	n/a
GCF-000157595.1	17	CC17	2	CC2	77	n/a	n/a	n/a
GCF-000772525.1	17	CC17	2	CC2	102	1992	USA: Brooklyn	Clinical isolate
GCF-001996345.1	916	CC17	2	CC2	131	2013	Italy: Sassari	Bile
GCF-000392215.1	117	CC17	581	CC2	702	1994	n/a	n/a
GCF-000322205.1	117	CC17	480	CC2	517	2001	USA	Urine
GCF-000321545.1	80	CC17	564	CC128	501	1997	Israel	Blood
GCF-026929095.1	18	CC17	1025	CC128	1708	1993	USA: Iowa	Clinical isolate
GCF-004152365.1	17	CC17	50	CC2	4685	2014	Pakistan: Islamabad	Clinical isolate
GCF-026949185.1	17	CC17	276	CC2		2014	Belgium: Ronse	Catheter
GCF-026949225.1	17	CC17	276	CC2	2060	2014	Belgium: Leuven	Clinical isolate
GCF-026929575.1	17	CC17	276	CC2	2060	2019	Belgium: Wilrijk	n/a
GCF-026953855.1	17	CC17	276	CC2	2060	2019	Belgium: Wilrijk	n/a
GCF-026934785.1	17	CC17	276	CC2	2060	2018	Belgium: Leuven	Stool
GCF-026934385.1	17	CC17	276	CC2	2060	2019	Belgium: Wilrijk	n/a
GCF-900638805.1	78	CC17	27	CC2	523	n/a	n/a	n/a
GCF-000322425.1	78	CC17	27	CC2	523	n/a	Denmark	Stool

GCF-900180455.1	80	CC17	24	CC2	1549	2015	United Kingdom	Human
GCF-900179695.1	80	CC17	24	CC2	1549	2015	United Kingdom	Human
GCF-900179145.1	80	CC17	24	CC2	1549	2015	United Kingdom	Human
GCF-900179655.1	80	CC17	24	CC2	1549	2015	United Kingdom	Human
GCF-900179155.1	80	CC17	24	CC2	1549	2015	United Kingdom	Human
GCF-900179165.1	80	CC17	24	CC2	1549	2015	United Kingdom	Human
GCF-900179675.1	80	CC17	24	CC2	1549	2015	United Kingdom	Human
GCF-900180235.1	80	CC17	24	CC2	1549	2015	United Kingdom	Human
GCF-900179715.1	80	CC17	24	CC2	1549	2015	United Kingdom	Human
GCF-900179535.1	80	CC17	24	CC2	1549	2015	United Kingdom	Human
GCF-900179525.1	80	CC17	24	CC2	1549	2015	United Kingdom	Human
GCF-900180025.1	80	CC17	24	CC2	1549	2015	United Kingdom	Human
GCF-900180015.1	80	CC17	24	CC2	1549	2015	United Kingdom	Human
GCF-900179135.1	80	CC17	24	CC2	1549	2015	United Kingdom	Human
GCF-900178615.1	80	CC17	24	CC2	1549	2015	United Kingdom	Human
GCF-900179665.1	80	CC17	24	CC2	1549	2015	United Kingdom	Human
GCF-900178695.1	80	CC17	24	CC2	1549	2015	United Kingdom	Human
GCF-900178825.1	80	CC17	24	CC2	1549	2015	United Kingdom	Human
GCF-900178865.1	80	CC17	24	CC2	1549	2015	United Kingdom	Human
GCF-900178885.1	80	CC17	24	CC2	1549	2015	United Kingdom	Human
GCF-900179245.1	80	CC17	24	CC2	1549	2015	United Kingdom	Human
GCF-900179545.1	80	CC17	24	CC2	1549	2015	United Kingdom	Human
GCF-900179255.1	80	CC17	24	CC2	1549	2015	United Kingdom	Human
GCF-900179265.1	80	CC17	24	CC2	1549	2015	United Kingdom	Human
GCF-900179325.1	80	CC17	24	CC2	1549	2015	United Kingdom	Human
GCF-900179365.1	80	CC17	24	CC2	1549	2015	United Kingdom	Human
GCF-900179465.1	80	CC17	24	CC2	1549	2015	United Kingdom	Human
GCF-900179395.1	80	CC17	24	CC2	1549	2015	United Kingdom	Human
GCF-900179405.1	80	CC17	24	CC2	1549	2015	United Kingdom	Human
GCF-900179745.1	80	CC17	24	CC2	1549	2015	United Kingdom	Human
GCF-900179795.1	80	CC17	24	CC2	1549	2015	United Kingdom	Human
GCF-900179835.1	80	CC17	24	CC2	1549	2015	United Kingdom	Human
GCF-900179855.1	80	CC17	24	CC2	1549	2015	United Kingdom	Human
GCF-900179895.1	80	CC17	24	CC2	1549	2015	United Kingdom	Human
GCF-900179945.1	80	CC17	24	CC2	1549	2015	United Kingdom	Human
GCF-900179985.1	80	CC17	24	CC2	1549	2015	United Kingdom	Human
GCF-900179905.1	80	CC17	24	CC2	1549	2015	United Kingdom	Human
GCF-900179725.1	80	CC17	24	CC2	1549	2015	United Kingdom	Human
GCF-900179505.1	80	CC17	24	CC2	1549	2015	United Kingdom	Human
GCF-900179775.1	80	CC17	24	CC2	1549	2015	United Kingdom	Human
GCF-900179485.1	80	CC17	24	CC2	1549	2015	United Kingdom	Human
GCF-900178665.1	80	CC17	24	CC2	1549	2015	United Kingdom	Human
GCF-900178705.1	80	CC17	24	CC2	1549	2015	United Kingdom	Human
GCF-900179125.1	80	CC17	24	CC2	1549	2015	United Kingdom	Human
GCF-900178675.1	80	CC17	24	CC2	1549	2015	United Kingdom	Human
GCF-900179645.1	80	CC17	24	CC2	1549	2015	United Kingdom	Human
GCF-900179705.1	80	CC17	24	CC2	1549	2015	United Kingdom	Human
GCF-900179965.1	80	CC17	24	CC2	1549	2015	United Kingdom	Human
GCF-900179685.1	80	CC17	24	CC2	1549	2015	United Kingdom	Human
GCF-900180145.1	80	CC17	24	CC2	1549	2015	United Kingdom	Human
GCF-900179625.1	80	CC17	24	CC2	1549	2015	United Kingdom	Human
GCF-900179635.1	80	CC17	24	CC2	1549	2015	United Kingdom	Human
GCF-900179615.1	80	CC17	24	CC2	1549	2015	United Kingdom	Human
GCF-900179605.1	80	CC17	24	CC2	1549	2015	United Kingdom	Human
GCF-900179575.1	80	CC17	24	CC2	1549	2015	United Kingdom	Human
GCF-900179565.1	80	CC17	24	CC2	1549	2015	United Kingdom	Human
GCF-900180135.1	80	CC17	24	CC2	1549	2015	United Kingdom	Human
GCF-900179595.1	80	CC17	24	CC2	1549	2015	United Kingdom	Human
GCF-900178575.1	80	CC17	24	CC2	1549	2015	United Kingdom	Human
GCF-900178605.1	80	CC17	24	CC2	1549	2015	United Kingdom	Human
GCF-900179865.1	80	CC17	24	CC2	1549	2015	United Kingdom	Human
GCF-900179735.1	80	CC17	24	CC2	1549	2015	United Kingdom	Human
GCF-900179045.1	80	CC17	24	CC2	1549	2015	United Kingdom	Human
GCF-900179315.1	80	CC17	24	CC2	1549	2015	United Kingdom	Human

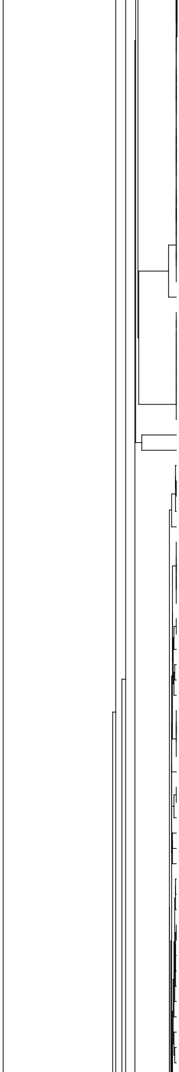

GCF-900179755.1	80	CC17	24	CC2	1549	2015	United Kingdom	Human
GCF-900179845.1	80	CC17	24	CC2	1549	2015	United Kingdom	Human
GCF-900180045.1	80	CC17	24	CC2	1549	2015	United Kingdom	Human
GCF-900179375.1	80	CC17	24	CC2	1549	2015	United Kingdom	Human
GCF-900179185.1	80	CC17	24	CC2	1549	2015	United Kingdom	Human
GCF-900179195.1	80	CC17	24	CC2	1549	2015	United Kingdom	Human
GCF-900179415.1	80	CC17	24	CC2	1549	2015	United Kingdom	Human
GCF-900179455.1	80	CC17	24	CC2	1549	2015	United Kingdom	Human
GCF-900179025.1	80	CC17	24	CC2	1549	2015	United Kingdom	Human
GCF-900178735.1	80	CC17	24	CC2	1549	2015	United Kingdom	Human
GCF-900178955.1	80	CC17	24	CC2	1549	2015	United Kingdom	Human
GCF-900178945.1	80	CC17	24	CC2	1549	2015	United Kingdom	Human
GCF-900178895.1	80	CC17	24	CC2	1549	2015	United Kingdom	Human
GCF-900178585.1	80	CC17	24	CC2	1549	2015	United Kingdom	Human
GCF-900179015.1	80	CC17	24	CC2	1549	2015	United Kingdom	Human
GCF-900179305.1	80	CC17	24	CC2	1549	2015	United Kingdom	Human
GCF-900179555.1	80	CC17	24	CC2	1549	2015	United Kingdom	Human
GCF-900180125.1	80	CC17	24	CC2	1549	2015	United Kingdom	Human
GCF-900179585.1	80	CC17	24	CC2	1549	2015	United Kingdom	Human
GCF-015770515.1	80	CC17	24	CC2	6852	2017	USA: Pennsylvania	Rectal swab
GCF-010722575.1	17	CC17	47	CC2	154	2016	Netherlands	Clinical isolate
GCF-010722535.1	17	CC17	47	CC2	154	2016	Netherlands	Clinical isolate
GCF-010722195.1	17	CC17	47	CC2	154	2016	Netherlands	Clinical isolate
GCF-010722815.1	17	CC17	47	CC2	154	2016	Netherlands	Clinical isolate
GCF-010722315.1	17	CC17	47	CC2	154	2016	Netherlands	Clinical isolate
GCF-010722695.1	17	CC17	47	CC2	154	2016	Netherlands	Clinical isolate
GCF-010722435.1	17	CC17	47	CC2	154	2016	Netherlands	Clinical isolate
GCF-008123945.1	17	CC17	47	CC2	154	2016	Netherlands:Maastricht	Stool
GCF-900179515.1	17	CC17	629	CC2	4162	2015	United Kingdom	Human
FI471	17	CC17	6	CC7	2026	2019	Czech republic: Brno	Rectal swab
GCF-015770505.1	17	CC17	8	CC2	3709	2017	USA: Pennsylvania	Rectal swab
GCF-015768845.1	17	CC17	8	CC2	3709	2018	USA: Pennsylvania	Rectal swab
GCF-015771615.1	17	CC17	8	CC2	3709	2017	USA: Pennsylvania	Rectal swab
GCF-015768105.1	17	CC17	8	CC2	3709	2018	USA: Pennsylvania	Rectal swab
GCF-015769845.1	17	CC17	8	CC2	6843	2017	USA: Pennsylvania	Rectal swab
GCF-015768905.1	17	CC17	8	CC2	4016	2018	USA: Pennsylvania	Rectal swab
GCF-015769615.1	17	CC17	8	CC2	4016	2017	USA: Pennsylvania	Rectal swab
GCF-015768775.1	17	CC17	8	CC2	4016	2018	USA: Pennsylvania	Rectal swab
GCF-015774295.1	17	CC17	8	CC2	4016	2017	USA: Pennsylvania	Rectal swab
GCF-015772375.1	17	CC17	10	CC2	4016	2017	USA: Pennsylvania	Rectal swab
GCF-015769035.1	17	CC17	8	CC2	3749	2018	USA: Pennsylvania	Rectal swab
GCF-015772185.1	17	CC17	8	CC2	3749	2017	USA: Pennsylvania	Rectal swab
GCF-015770755.1	17	CC17	8	CC2	3720	2017	USA: Pennsylvania	Rectal swab
GCF-015769195.1	17	CC17	8	CC2	3749	2018	USA: Pennsylvania	Rectal swab
GCF-015772265.1	17	CC17	8	CC2	3749	2017	USA: Pennsylvania	Rectal swab
GCF-015772355.1	17	CC17	8	CC2	3749	2017	USA: Pennsylvania	Rectal swab
GCF-015768985.1	17	CC17	8	CC2	3746	2018	USA: Pennsylvania	Rectal swab
GCF-015772075.1	17	CC17	8	CC2	3746	2017	USA: Pennsylvania	Rectal swab
GCF-015769655.1	17	CC17	8	CC2	3746	2018	USA: Pennsylvania	Wound
GCF-015770785.1	17	CC17	8	CC2	3746	2017	USA: Pennsylvania	Rectal swab
GCF-015769715.1	17	CC17	8	CC2	6841	2018	USA: Pennsylvania	Blood
GCF-015771635.1	17	CC17	8	CC2	3722	2017	USA: Pennsylvania	Rectal swab
GCF-015769115.1	17	CC17	8	CC2	3722	2018	USA: Pennsylvania	Rectal swab
GCF-015771675.1	17	CC17	8	CC2	3722	2017	USA: Pennsylvania	Rectal swab
GCF-015771695.1	17	CC17	8	CC2	5776	2017	USA: Pennsylvania	Rectal swab
GCF-015770595.1	17	CC17	8	CC2	6853	2017	USA: Pennsylvania	Rectal swab
GCF-015774275.1	17	CC17	8	CC2	6869	2017	USA: Pennsylvania	Urine
GCF-015768475.1	17	CC17	8	CC2	3104	2018	USA: Pennsylvania	Rectal swab
GCF-015771355.1	17	CC17	8	CC2	4958	2017	USA: Pennsylvania	Rectal swab
GCF-015770645.1	17	CC17	8	CC2	3104	2017	USA: Pennsylvania	Rectal swab
GCF-015769645.1	17	CC17	8	CC2	3104	2017	USA: Pennsylvania	Rectal swab
GCF-015769495.1	17	CC17	8	CC2	3104	2018	USA: Pennsylvania	Rectal swab
GCF-015773435.1	17	CC17	8	CC2	3104	2017	USA: Pennsylvania	Rectal swab
GCF-015773895.1	17	CC17	8	CC2	3104	2017	USA: Pennsylvania	Rectal swab
GCF-015772245.1	17	CC17	8	CC2	3104	2017	USA: Pennsylvania	Rectal swab
GCF-015769275.1	17	CC17	8	CC2	3104	2018	USA: Pennsylvania	Rectal swab
GCF-015771445.1	17	CC17	8	CC2	5761	2017	USA: Pennsylvania	Rectal swab
GCF-015768355.1	17	CC17	8	CC2	3104	2018	USA: Pennsylvania	Rectal swab
GCF-015771395.1	17	CC17	8	CC2	3104	2017	USA: Pennsylvania	Rectal swab
GCF-015772115.1	17	CC17	8	CC2	3104	2017	USA: Pennsylvania	Rectal swab

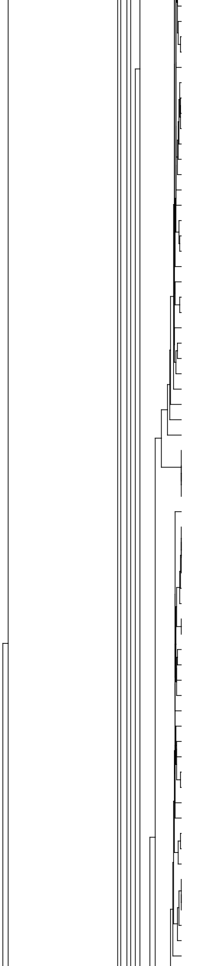

GCF-015771555.1	17	CC17	9	CC2	3104	2017	USA: Pennsylvania	Rectal swab
GCF-015706555.1	17	CC17	8	CC2	3104	2018	USA: Pennsylvania	Rectal swab
GCF-015773905.1	17	CC17	8	CC2	3104	2017	USA: Pennsylvania	Rectal swab
GCF-015771835.1	17	CC17	8	CC2	3104	2017	USA: Pennsylvania	Urine
GCF-015770175.1	17	CC17	8	CC2	3720	2017	USA: Pennsylvania	Rectal swab
GCF-015768415.1	17	CC17	8	CC2	3714	2018	USA: Pennsylvania	Rectal swab
GCF-015771405.1	17	CC17	8	CC2	3714	2017	USA: Pennsylvania	Rectal swab
GCF-015772135.1	17	CC17	8	CC2	3714	2017	USA: Pennsylvania	Rectal swab
GCF-015768065.1	17	CC17	8	CC2	3714	2018	USA: Pennsylvania	Rectal swab
GCF-015769155.1	17	CC17	8	CC2	3714	2018	USA: Pennsylvania	Rectal swab
GCF-015770315.1	17	CC17	8	CC2	5734	2017	USA: Pennsylvania	Rectal swab
GCF-015769815.1	17	CC17	8	CC2	3714	2017	USA: Pennsylvania	Rectal swab
GCF-015771965.1	17	CC17	8	CC2	6861	2017	USA: Pennsylvania	Rectal swab
GCF-015772145.1	17	CC17	8	CC2	6863	2017	USA: Pennsylvania	Rectal swab
GCF-015771845.1	17	CC17	43	CC2	6859	2017	USA: Pennsylvania	Rectal swab
GCF-015769105.1	17	CC17	43	CC2	4016	2018	USA: Pennsylvania	Rectal swab
GCF-015769225.1	17	CC17	43	CC2	5663	2018	USA: Pennsylvania	Rectal swab
GCF-015771255.1	17	CC17	8	CC2	5659	2017	USA: Pennsylvania	Rectal swab
GCF-015768535.1	17	CC17	8	CC2	6833	2018	USA: Pennsylvania	Rectal swab
GCF-015706535.1	17	CC17	8	CC2	3736	2018	USA: Pennsylvania	Rectal swab
GCF-015769075.1	17	CC17	8	CC2	3736	2018	USA: Pennsylvania	Rectal swab
GCF-015768455.1	17	CC17	8	CC2	6832	2018	USA: Pennsylvania	Rectal swab
GCF-015771855.1	17	CC17	8	CC2	4682	2017	USA: Pennsylvania	Rectal swab
GCF-015772465.1	17	CC17	8	CC2	4682	2017	USA: Pennsylvania	Rectal swab
GCF-015773445.1	17	CC17	8	CC2	5658	2017	USA: Pennsylvania	Rectal swab
GCF-015770275.1	17	CC17	8	CC2	5768	2017	USA: Pennsylvania	Wound
GCF-004152575.1	17	CC17	8	CC2	4686	2018	USA: St. Louis	Clinical isolate
GCF-015771295.1	17	CC17	8	CC2	5682	2017	USA: Pennsylvania	Rectal swab
GCF-015769215.1	2270	CC17	8	CC2	6839	2018	USA: Pennsylvania	Rectal swab
GCF-004152205.1	78	CC17	31	CC2	779	2018	USA: St. Louis	Clinical isolate
GCF-004152505.1	78	CC17	31	CC2	779	2018	USA: St. Louis	Clinical isolate
GCF-022509555.1	78	CC17	31	CC2	779	2018	Pakistan: Islamabad	Dahi fermented milk product
GCF-004152185.1	78	CC17	31	CC2	779	2018	USA: St. Louis	Clinical isolate
GCF-015771935.1	18	CC17	133	CC128	6860	2017	USA: Pennsylvania	Rectal swab
GCF-003350715.1	967	CC17	133	CC128	4872	2013	USA: Utah	Blood
GCF-002158375.1	967	CC17	133	CC128	4872	2014	USA: Utah	Blood
GCF-002158365.1	967	CC17	133	CC128	4872	2014	USA: Utah	Blood
GCF-002158435.1	967	CC17	133	CC128	4872	2014	USA: Utah	Blood
GCF-003350865.1	967	CC17	133	CC128	4872	2013	USA: Utah	Stool
GCF-003350905.1	967	CC17	133	CC128	4872	2014	USA: Utah	Stool
GCF-016415365.1	18	CC17	133	CC128	5780	2015	USA: Dallas	Rectal swab
GCF-016415385.1	18	CC17	133	CC128	5780	2015	USA: Dallas	Rectal swab
GCF-016406405.1	18	CC17	133	CC128	5783	2015	USA: Dallas	Rectal swab
GCF-018296025.1	18	CC17	133	CC128	170	2016	USA: Ann Arbor	Rectal swab
GCF-018219325.1	18	CC17	133	CC128	5919	2016	USA: Ann Arbor	Rectal swab
GCF-015773455.1	18	CC17	133	CC128	4965	2017	USA: Pennsylvania	Rectal swab
GCF-016415405.1	18	CC17	133	CC128	5789	2015	USA: Dallas	Rectal swab
GCF-015772085.1	18	CC17	133	CC128	6862	2017	USA: Pennsylvania	Rectal swab
GCF-001721905.1	18	CC17	133	CC128	170	2011	USA: New York	Blood
GCF-015772345.1	18	CC17	133	CC128	170	2017	USA: Pennsylvania	Rectal swab
GCF-015770015.1	18	CC17	133	CC128	6844	2017	USA: Pennsylvania	Rectal swab
GCF-015769005.1	18	CC17	133	CC128	170	2018	USA: Pennsylvania	Rectal swab
GCF-015769595.1	18	CC17	133	CC128	6840	2017	USA: Pennsylvania	Rectal swab
GCF-000395845.1	18	CC17	133	CC128	746	2010	Denmark	n/a
GCF-001060065.1	18	CC17	133	CC128	735	n/a	USA:Washington	Clinical isolate
GCF-001056925.1	18	CC17	133	CC128	735	n/a	USA:Washington	Clinical isolate
GCF-001052685.1	18	CC17	133	CC128	735	n/a	USA:Washington	Clinical isolate
GCF-004152405.1	18	CC17	267	CC128	4694	2018	USA: St. Louis	Clinical isolate
GCF-004152485.1	18	CC17	267	CC128	4694	2018	USA: St. Louis	Clinical isolate
GCF-004151895.1	18	CC17	267	CC128	4694	2018	USA: St. Louis	Clinical isolate
GCF-004151765.1	18	CC17	267	CC128	4694	2018	USA: St. Louis	Hospital Surface
GCF-004152075.1	18	CC17	267	CC128	4698	2018	USA: St. Louis	Clinical isolate
GCF-015769535.1	18	CC17	133	CC128	4484	2018	USA: Pennsylvania	Rectal swab

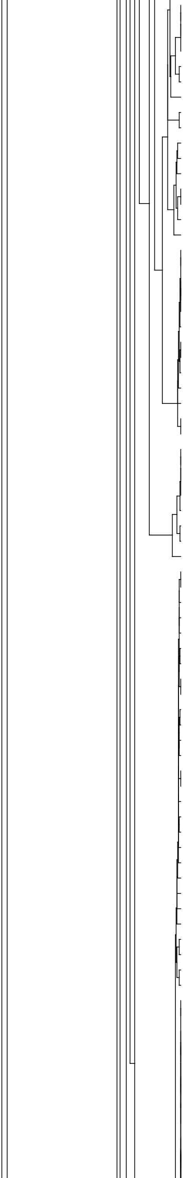

GCF-004151925.1	18	CC17	133	CC128	4696	2018	USA: St. Louis	Hospital Surface
GCF-004151875.1	18	CC17	133	CC128	4696	2018	USA: St. Louis	Hospital Surface
GCF-004151785.1	18	CC17	133	CC128	4696	2018	USA: St. Louis	Hospital Surface
GCF-004151795.1	18	CC17	133	CC128	4696	2018	USA: St. Louis	Hospital Surface
GCF-004152585.1	18	CC17	133	CC128	4695	2018	USA: St. Louis	Clinical isolate
GCF-004151775.1	18	CC17	133	CC128	4695	2018	USA: St. Louis	Hospital Surface
GCF-004152005.1	18	CC17	133	CC128	4697	2018	USA: St. Louis	Clinical isolate
GCF-015767965.1	18	CC17	133	CC128	3717	2018	USA: Pennsylvania	Rectal swab
GCF-015671075.1	18	CC17	133	CC128	3717	2016	USA	Stool
GCF-016126675.1	18	CC17	133	CC128	675	2015	USA: Dallas	Rectal swab
GCF-018219345.1	18	CC17	133	CC128	5920	2016	USA: Ann Arbor	Rectal swab
GCF-015768815.1	18	CC17	133	CC128	6835	2018	USA: Pennsylvania	Urine
GCF-015771655.1	18	CC17	133	CC128	4967	2017	USA: Pennsylvania	Rectal swab
GCF-015772485.1	18	CC17	133	CC128	4967	2017	USA: Pennsylvania	Rectal swab
GCF-016415025.1	18	CC17	133	CC128	5787	2015	USA: Dallas	Rectal swab
GCF-016415285.1	18	CC17	135	CC128	5778	2015	USA: Dallas	Rectal swab
GCF-015999605.1	17	CC17	117	CC2	5706	2015	USA: Dallas	Rectal swab
GCF-016406345.1	17	CC17	117	CC2	5706	2015	USA: Dallas	Rectal swab
GCF-016406365.1	17	CC17	117	CC2	5706	2015	USA: Dallas	Rectal swab
GCF-016406545.1	17	CC17	117	CC2	5706	2015	USA: Dallas	Rectal swab
GCF-016406425.1	17	CC17	117	CC2	5706	2015	USA: Dallas	Rectal swab
GCF-016415425.1	17	CC17	117	CC2	5706	2015	USA: Dallas	Rectal swab
GCF-016406565.1	17	CC17	117	CC2	5706	2015	USA: Dallas	Rectal swab
GCF-016415105.1	17	CC17	117	CC2	5706	2015	USA: Dallas	Rectal swab
GCF-016406585.1	17	CC17	117	CC2	5706	2015	USA: Dallas	Rectal swab
GCF-016415465.1	17	CC17	117	CC2	5706	2015	USA: Dallas	Rectal swab
GCF-016415325.1	17	CC17	117	CC2	5706	2015	USA: Dallas	Rectal swab
GCF-016406525.1	1383	CC17	117	CC2	5786	2015	USA: Dallas	Rectal swab
GCF-016455345.1	1383	CC17	117	CC2	5706	2015	USA: Dallas	Rectal swab
GCF-004300345.1	80	CC17	138		3419	2018	USA:Little Rock	Blood
GCF-004300005.1	80	CC17	138		3419	2018	USA:Little Rock	Blood
GCF-004300025.1	80	CC17	138		3419	2018	USA:Little Rock	Blood
GCF-004300105.1	80	CC17	138		3419	2018	USA:Little Rock	Blood
GCF-004300525.1	2276	CC17	138		3419	2018	USA:Little Rock	Blood
GCF-004300185.1	80	CC17	138		3419	2018	USA:Little Rock	Blood
GCF-004300505.1	80	CC17	138		3419	2018	USA:Little Rock	Blood
GCF-004300065.1	992	CC17	187	CC184	4874	2018	USA:Little Rock	Blood
GCF-020176925.1	117	CC17	17	CC17	24	2012	Netherlands	Blood
GCF-900639465.1	117	CC17	17	CC17	24	2010	Netherlands	n/a
GCF-026922095.1	117	CC17	17	CC17	24	2014	Belgium: Leuven	Blood
GCF-026929155.1	117	CC17	17	CC17	24	2013	Denmark: Copenhagen	Clinical isolate
GCF-001542895.1	117	CC17	17	CC17	24	2014	Sweden: Gaevle	Stool
GCF-026922335.1	117	CC17	17	CC17	24	2013	Belgium: Montignies-Le-Tilleul	Clinical isolate
GCF-026952225.1	117	CC17	17	CC17	24	2014	Belgium: Namur	Urine
GCF-026922355.1	117	CC17	17	CC17	24	2013	Belgium: La Louviere	Peritoneal
GCF-026922155.1	117	CC17	17	CC17	24	2013	Belgium: La Louviere	Peritoneal
GCF-022468475.1	117	CC17	17	CC17	24	2017	Israel: Jerusalem	Blood
GCF-022468695.1	117	CC17	17	CC17	24	2018	Israel: Jerusalem	Blood
GCF-900639565.1	117	CC17	17	CC17	24	2014	Netherlands	n/a
GCF-900639505.1	117	CC17	17	CC17	24	2012	Netherlands	n/a
GCF-026929115.1	117	CC17	17	CC17	24	2012	Denmark: Copenhagen	Clinical isolate
GCF-026953495.1	117	CC17	17	CC17	24	2012	Denmark: Copenhagen	Clinical isolate
GCF-900639535.1	117	CC17	17	CC17	24	2015	Netherlands	n/a
GCF-026950845.1	117	CC17	17	CC17	24	2010	Belgium: Leuven	Blood
GCF-001886635.1	117	CC17	17	CC17	24	2010	Spain: Madrid	Blood
GCF-003202445.1	795	CC17	17	CC17	24	2009	Saudi Arabia: Riyadh	Clinical isolate
GCF-020178155.1	117	CC17	17	CC17	24	2012	Netherlands	Blood
GCF-016864255.1	117	CC17	17	CC17	24	2017	Norway: Bergen	Urine
GCF-026953065.1	117	CC17	17	CC17	1199	2016	Belgium: Gilly	Blood
GCF-026952165.1	117	CC17	17	CC17		2014	Belgium: Edegem	Wound
GCF-026953225.1	117	CC17	17	CC17		2015	Belgium: Hasselt	Tissue
GCF-022468875.1	117	CC17	17	CC17	6899	2018	Israel: Jerusalem	Blood
GCF-022463795.1	117	CC17	17	CC17	6899	2018	Israel: Jerusalem	Blood
GCF-022468375.1	117	CC17	17	CC17	6914	2017	Israel: Jerusalem	Blood
GCF-022469465.1	117	CC17	17	CC17	6914	2013	Israel: Jerusalem	Blood
FI117	117	CC17	42	CC17	6756	2017	Czech republic: Brno	Rectal swab
FI78	117	CC17	42	CC17	6756	2017	Czech republic: Brno	Rectal swab
FI59	117	CC17	42	CC17	6756	2017	Czech republic: Brno	Rectal swab
FI71	117	CC17	42	CC17	6756	2017	Czech republic: Brno	Rectal swab
FI87	117	CC17	42	CC17	6756	2017	Czech republic: Brno	Rectal swab
FI238	117	CC17	42	CC17	6756	2018	Czech republic: Brno	Rectal swab
FI161	117	CC17	42	CC17	6756	2017	Czech republic: Brno	Urine
FI68	117	CC17	42	CC17	6756	2017	Czech republic: Brno	Rectal swab
FI64	117	CC17	42	CC17	6756	2017	Czech republic: Brno	Rectal swab
FI111	117	CC17	42	CC17	6756	2017	Czech republic: Brno	Rectal swab
FI44	117	CC17	42	CC17	6756	2017	Czech republic: Brno	Rectal swab
FI70	117	CC17	42	CC17	6756	2017	Czech republic: Brno	Rectal swab

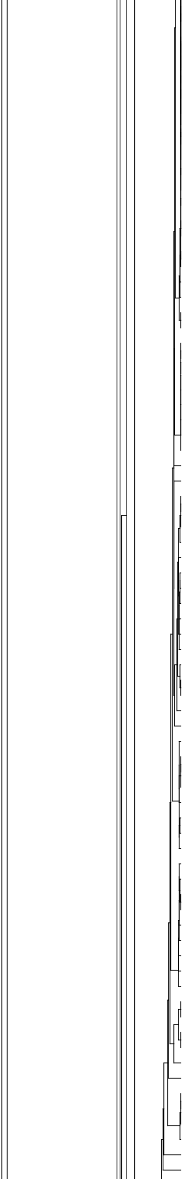

FI102	117	CC17	42	CC17	6756	2017	Czech republic: Brno	Rectal swab
FI77	117	CC17	42	CC17	6756	2017	Czech republic: Brno	Stool
FI62	117	CC17	42	CC17	6756	2017	Czech republic: Brno	Rectal swab
FI74	117	CC17	42	CC17	6756	2017	Czech republic: Brno	Rectal swab
FI93	117	CC17	42	CC17	6756	2017	Czech republic: Brno	Stool
FI66	117	CC17	42	CC17	6756	2017	Czech republic: Brno	Rectal swab
FI98	117	CC17	42	CC17	6756	2017	Czech republic: Brno	Rectal swab
FI92	117	CC17	42	CC17	6756	2017	Czech republic: Brno	Stool
FI99	117	CC17	42	CC17	6756	2017	Czech republic: Brno	Rectal swab
FI83	117	CC17	42	CC17	6756	2017	Czech republic: Brno	Urine
FI80	117	CC17	42	CC17	6756	2017	Czech republic: Brno	Rectal swab
FI51	117	CC17	42	CC17	6756	2017	Czech republic: Brno	Rectal swab
FI48	117	CC17	42	CC17	6756	2017	Czech republic: Brno	Rectal swab
FI115	117	CC17	42	CC17	6756	2017	Czech republic: Brno	Rectal swab
FI39	117	CC17	42	CC17	6756	2017	Czech republic: Brno	Rectal swab
FI41	117	CC17	42	CC17	6756	2017	Czech republic: Brno	Urine
FI108	117	CC17	42	CC17	6756	2017	Czech republic: Brno	Rectal swab
FI100	117	CC17	42	CC17	6756	2017	Czech republic: Brno	Rectal swab
FI124	2284	CC17	42	CC17	6756	2017	Czech republic: Brno	Rectal swab
FI54	117	CC17	42	CC17	6756	2017	Czech republic: Brno	Urine
FI67	117	CC17	42	CC17	6756	2017	Czech republic: Brno	Rectal swab
FI82	117	CC17	42	CC17	6756	2017	Czech republic: Brno	Rectal swab
GCF-001076335.1	117	CC17	17	CC17	697	n/a	USA:Washington	Clinical isolate
GCF-001052405.1	117	CC17	17	CC17	697	n/a	USA:Washington	Clinical isolate
GCF-001052435.1	117	CC17	17	CC17	697	n/a	USA:Washington	Clinical isolate
GCF-001052485.1	117	CC17	17	CC17	697	n/a	USA:Washington	Clinical isolate
GCF-001054055.1	117	CC17	17	CC17	697	n/a	USA:Washington	Clinical isolate
GCF-001053135.1	117	CC17	17	CC17	697	n/a	USA:Washington	Clinical isolate
GCF-001052395.1	117	CC17	17	CC17	697	n/a	USA:Washington	Clinical isolate
GCF-001054215.1	117	CC17	17	CC17	697	n/a	USA:Washington	Clinical isolate
FI45	117	CC17	17	CC17	6952	2017	Czech republic: Brno	Rectal swab
GCF-022468615.1	117	CC17	17	CC17	6917	2018	Israel: Jerusalem	Blood
GCF-022464215.1	117	CC17	17	CC17	3196	2014	Israel: Jerusalem	Blood
GCF-022464005.1	117	CC17	17	CC17	3196	2014	Israel: Jerusalem	Blood
GCF-022463645.1	117	CC17	17	CC17	3196	2017	Israel: Jerusalem	Blood
GCF-022463085.1	117	CC17	17	CC17	3196	2020	Israel: Jerusalem	Blood
GCF-026951165.1	1465	CC17	17	CC17	3196	2018	Belgium: Liege	Clinical isolate
GCF-026950655.1	117	CC17	17	CC17	3196	2017	Belgium: Brussels	Rectal swab
GCF-026943595.1	117	CC17	17	CC17	3196	2017	Belgium: Brussels	Rectal swab
GCF-026943555.1	117	CC17	17	CC17	3196	2017	Belgium: Brussels	Rectal swab
GCF-026953185.1	117	CC17	17	CC17	3196	2016	Belgium: Ath	Blood
GCF-026950835.1	117	CC17	17	CC17	3196	2018	Belgium: Mons	Rectal swab
GCF-026921135.1	117	CC17	17	CC17	3196	2017	Belgium: Bouge	Stool
GCF-026951835.1	117	CC17	17	CC17	3196	2017	Belgium: Waregem	Bladder probe
GCF-026949725.1	117	CC17	17	CC17	3196	2018	Belgium: Waregem	Rectal swab
GCF-026949785.1	117	CC17	17	CC17	3196	2017	Belgium: Waregem	Wound
GCF-026951405.1	117	CC17	17	CC17	3196	2018	Belgium: Boussu	Rectal swab
GCF-008728475.1	117	CC17	17	CC17	1180	2018	Denmark:Copenhagen	Rectal swab
GCF-026953045.1	117	CC17	17	CC17	1207	2016	Belgium: Brussels	Blood
GCF-026950325.1	117	CC17	17	CC17	1207	2017	Belgium: Godinne	Rectal swab
GCF-026950445.1	117	CC17	17	CC17	1207	2017	Belgium: Godinne	Rectal swab
GCF-026950365.1	117	CC17	17	CC17	1207	2017	Belgium: Godinne	Rectal swab
GCF-026950515.1	117	CC17	17	CC17	1207	2017	Belgium: Gilly	Stool
GCF-026951005.1	117	CC17	17	CC17	1207	2018	Belgium: Brussels	Rectal swab
GCF-026921695.1	117	CC17	17	CC17	1207	2018	Belgium: La Louviere	Rectal swab
GCF-026951305.1	117	CC17	17	CC17	1207	2018	Belgium: La Louviere	Tracheal aspirate
GCF-026953395.1	117	CC17	17	CC17	1192	2015	Belgium: Liege	Blood
GCF-026928925.1	117	CC17	17	CC17	1192	2015	Belgium: Antwerp	Blood
GCF-026928435.1	117	CC17	17	CC17	1192	2014	Belgium: Edegem	Blood
GCF-026952175.1	117	CC17	17	CC17	1192	2014	Belgium: Edegem	Blood
GCF-026950225.1	117	CC17	17	CC17	1192	2017	Belgium: Sint-Niklaas	Rectal swab
GCF-026949845.1	117	CC17	17	CC17	1192	2019	Belgium: Sint-Niklaas	Rectal swab
GCF-026921615.1	117	CC17	17	CC17	1192	2017	Belgium: Sint-Niklaas	Stool
GCF-026950455.1	117	CC17	17	CC17	1192	2017	Belgium: Sint-Niklaas	Urine
GCF-026922255.1	117	CC17	17	CC17	1192	2014	Belgium: Edegem	Clinical isolate
GCF-004300425.1	1478	CC17	17	CC17	4923	2018	USA:Little Rock	Blood
GCF-004300395.1	1478	CC17	17	CC17	4923	2018	USA:Little Rock	Blood
GCF-004152415.1	1478	CC17	17	CC17	3735	2018	USA: St. Louis	Hospital Surface
GCF-004151815.1	1478	CC17	17	CC17	3735	2018	USA: St. Louis	Hospital Surface
GCF-009697285.1	1478	CC17	17	CC17	3579	2017	Canada	Clinical isolate
GCF-900639715.1	117	CC17	17	CC17	268	2015	Netherlands	n/a
GCF-022463425.1	117	CC17	19	CC17	6891	2018	Israel: Jerusalem	Blood
GCF-022464075.1	117	CC17	19	CC17	6891	2012	Israel: Jerusalem	Blood
GCF-022464385.1	117	CC17	19	CC17	6891	2014	Israel: Jerusalem	Blood
GCF-022463075.1	117	CC17	19	CC17	6891	2020	Israel: Jerusalem	Blood
GCF-907165365.1	117	CC17	18	CC17	1477	2017	Switzerland	Rectal swab
FI57	117	CC17	42	CC17	6773	2017	Czech republic: Brno	Rectal swab

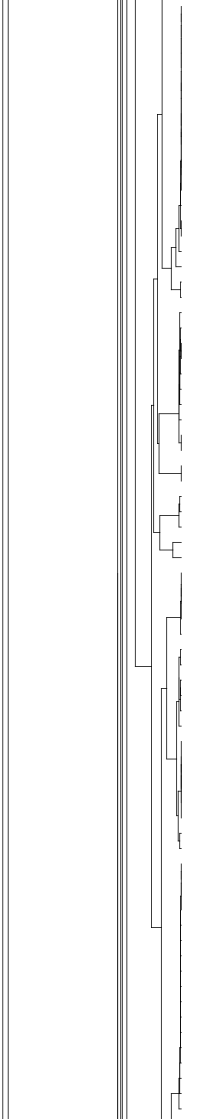

FI460	117	CC17	18	CC17	1526	2019	Czech republic: Brno	Rectal swab
FI446	117	CC17	18	CC17	1526	2019	Czech republic: Brno	Rectal swab
FIHEM15-6-19	117	CC17	18	CC17	1526	2017	Czech republic: Brno	Blood
FI450	117	CC17	18	CC17	1526	2019	Czech republic: Brno	Rectal swab
FI475	117	CC17	18	CC17	1526	2019	Czech republic: Brno	Rectal swab
FI464	117	CC17	18	CC17	1526	2019	Czech republic: Brno	Rectal swab
FI473	117	CC17	18	CC17	1526	2019	Czech republic: Brno	Rectal swab
FI466	117	CC17	18	CC17	1526	2019	Czech republic: Brno	Stool
FI461	117	CC17	18	CC17	1526	2019	Czech republic: Brno	Rectal swab
FI454	117	CC17	18	CC17	1526	2019	Czech republic: Brno	Rectal swab
FI465	117	CC17	18	CC17	1526	2019	Czech republic: Brno	Rectal swab
FI445	117	CC17	18	CC17	1526	2019	Czech republic: Brno	Rectal swab
FI451	117	CC17	18	CC17	1526	2019	Czech republic: Brno	Rectal swab
FI396	117	CC17	18	CC17	1526	2019	Czech republic: Brno	Urine
FI120	117	CC17	18	CC17	1526	2017	Czech republic: Brno	Rectal swab
FI94	117	CC17	18	CC17	1526	2017	Czech republic: Brno	Urine
FI422	117	CC17	18	CC17	1526	2019	Czech republic: Brno	Rectal swab
FI486	117	CC17	18	CC17		2019	Czech republic: Brno	Rectal swab
GCF-026948485.1	117	CC17	839	CC17	36	2017	Belgium: Brussels	Blood
GCF-026921075.1	117	CC17	839	CC17	36	2017	Belgium: Brussels	Urine
GCF-022464785.1	18	CC17	21	CC128	6902	2016	Israel: Jerusalem	Blood
GCF-022468675.1	18	CC17	21	CC128	6902	2018	Israel: Jerusalem	Blood
GCF-022464955.1	18	CC17	21	CC128	6902	2014	Israel: Jerusalem	Blood
GCF-022463855.1	18	CC17	21	CC128	6902	2018	Israel: Jerusalem	Blood
GCF-022468955.1	18	CC17	21	CC128	6902	2018	Israel: Jerusalem	Blood
GCF-022468435.1	18	CC17	21	CC128	6902	2017	Israel: Jerusalem	Blood
GCF-022468885.1	18	CC17	21	CC128	6902	2018	Israel: Jerusalem	Blood
GCF-022469215.1	2274	CC17	21	CC128	6902	2015	Israel: Jerusalem	Blood
GCF-022468315.1	18	CC17	45	CC128	6902	2018	Israel: Jerusalem	Blood
GCF-022468225.1	18	CC17	45	CC128	6902	2016	Israel: Jerusalem	Blood
FI462	80	CC17	25	CC2	6769	2019	Czech republic: Brno	Rectal swab
FI476	80	CC17	25	CC2	6769	2019	Czech republic: Brno	Rectal swab
GCF-026953245.1	117	CC17	128	CC128	118	2015	Belgium: Mechelen	Blood
GCF-026953205.1	117	CC17	128	CC128	118	2015	Belgium: Mechelen	Skin
GCF-026928915.1	117	CC17	128	CC128	118	2015	Belgium: Godinne	Wound
GCF-026951995.1	117	CC17	258	CC17	1203	2014	Belgium: Sint-Niklaas	Blood
GCF-900639455.1	117	CC17	258	CC17	449	n/a	n/a	n/a
GCF-900639625.1	117	CC17	709	CC17	103	2015	Netherlands	n/a
GCF-900639615.1	117	CC17	709	CC17	103	2015	Netherlands	n/a
GCF-900639595.1	117	CC17	709	CC17	103	2015	Netherlands	n/a
GCF-900639605.1	117	CC17	709	CC17	103	2015	Netherlands	n/a
GCF-900639545.1	117	CC17	709	CC17	103	2013	Netherlands	n/a
GCF-026951605.1	117	CC17	17	CC17	71	2018	Belgium: Brussels	Stool
GCF-026951825.1	117	CC17	17	CC17	71	2018	Belgium: Brussels	Urine
GCF-026951485.1	117	CC17	17	CC17	71	2018	Belgium: Verviers	Stool
GCF-026951565.1	117	CC17	17	CC17	71	2018	Belgium: Verviers	Urine
GCF-026949865.1	117	CC17	17	CC17	71	2017	Belgium: Verviers	Urine
GCF-026951215.1	117	CC17	17	CC17	71	2018	Belgium: Roeselare	Stool
GCF-026951465.1	117	CC17	17	CC17	469	2018	Belgium: Brussels	Rectal swab
GCF-026952025.1	117	CC17	17	CC17	469	2018	Belgium: Brussels	Clinical isolate
GCF-026951665.1	117	CC17	17	CC17	469	2018	Belgium: Brussels	Stool
GCF-026951985.1	117	CC17	17	CC17	469	2018	Belgium: Brussels	Stool
GCF-026949705.1	117	CC17	17	CC17	469	2018	Belgium: Brussels	Expectoration
GCF-026951705.1	117	CC17	17	CC17	469	2018	Belgium: Brussels	Stool
GCF-026950825.1	117	CC17	17	CC17	469	2018	Belgium: Seraing	Rectal swab
GCF-026953105.1	117	CC17	17	CC17	469	2016	Belgium: Leuven	Wound
GCF-026947085.1	117	CC17	18	CC17	929	2017	Belgium: Turnhout	Rectal swab
GCF-026949005.1	117	CC17	18	CC17	929	2016	Belgium: Overpelt	Wound
GCF-026946905.1	117	CC17	18	CC17	929	2018	Belgium: Leuven	Tissue
GCF-026948585.1	117	CC17	18	CC17	929	2017	Belgium: Leuven	Wound
GCF-026949025.1	117	CC17	18	CC17	929	2016	Belgium: Overpelt	Clinical isolate
GCF-026947165.1	117	CC17	18	CC17	2505	2017	Belgium: Tongeren	Rectal swab
GCF-026948845.1	117	CC17	18	CC17	929	2017	Belgium: Overpelt	Clinical isolate
GCF-026948875.1	117	CC17	18	CC17	2505	2016	Belgium: Overpelt	Wound
GCF-026949205.1	117	CC17	18	CC17	929	2016	Belgium: Leuven	Rectal swab
GCF-026948745.1	117	CC17	18	CC17	2505	2017	Belgium: Overpelt	Clinical isolate
GCF-026948855.1	117	CC17	18	CC17	2505	2017	Belgium: Overpelt	Clinical isolate
GCF-026947205.1	117	CC17	18	CC17	2505	2017	Belgium: Tongeren	Rectal swab
GCF-026947305.1	117	CC17	18	CC17	6184	2017	Belgium: Tongeren	Urine
GCF-026948655.1	117	CC17	18	CC17	6184	2017	Belgium: Overpelt	Stool
GCF-026947245.1	117	CC17	18	CC17	6184	2017	Belgium: Tongeren	Urine
GCF-026946945.1	117	CC17	18	CC17	6184	2017	Belgium: Tessenderlo	Urine
GCF-026949055.1	117	CC17	18	CC17		2016	Belgium: Overpelt	Urine

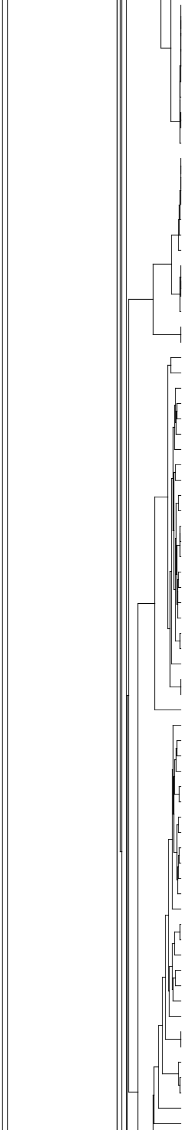

FI463	117	CC17	123	CC2	6770	2019	Czech republic: Brno	Rectal swab
FI429	117	CC17	123	CC2	6770	2019	Czech republic: Brno	Abdomen cavity
FI483	117	CC17	123	CC2	6770	2019	Czech republic: Brno	Rectal swab
FI502	117	CC17	123	CC2	6770	2019	Czech republic: Brno	Rectal swab
FI477	117	CC17	123	CC2	6770	2019	Czech republic: Brno	Rectal swab
FI498	117	CC17	123	CC2	6770	2019	Czech republic: Brno	Urine
FI501	117	CC17	123	CC2	6770	2019	Czech republic: Brno	Rectal swab
FI506	117	CC17	123	CC2	6770	2019	Czech republic: Brno	Rectal swab
FI507	117	CC17	123	CC2	6770	2019	Czech republic: Brno	Catheter
FI500	117	CC17	123	CC2	6770	2019	Czech republic: Brno	Rectal swab
GCF-026305275.1	343	CC17	889			2014	China:Liaoning province	Blood
GCF-026304715.1	343	CC17	889			2016	China:Liaoning province	Urine
GCF-026304775.1	343	CC17	889			2016	China:Liaoning province	Urine
GCF-026309075.1	343	CC17	889			2013	China:Liaoning province	Urine
GCF-026308825.1	343	CC17	889			2014	China:Liaoning province	Urine
GCF-026308905.1	343	CC17	889			2013	China:Liaoning province	Urine
GCF-026309175.1	343	CC17	889			2012	China:Liaoning province	Urine
GCF-026308785.1	363	CC17	1046			2014	China:Liaoning province	Urine
GCF-026308855.1	363	CC17	1046			2014	China:Liaoning province	Urine
GCF-026308695.1	363	CC17	1046			2014	China:Liaoning province	Urine
GCF-026305235.1	363	CC17	1046			2015	China:Liaoning province	Urine
GCF-026304975.1	363	CC17	894		6561	2016	China:Liaoning province	Drainage
GCF-026304865.1	363	CC17	894		6561	2016	China:Liaoning province	Puncture fluid
GCF-015770295.1	17	CC17	115	CC2	3747	2017	USA: Pennsylvania	Rectal swab
GCF-015770375.1	17	CC17	115	CC2	6850	2017	USA: Pennsylvania	Rectal swab
GCF-026409165.1	17	CC17	115	CC2		2019	USA	Clinical isolate
GCF-015771315.1	17	CC17	115	CC2	5810	2017	USA: Pennsylvania	Rectal swab
GCF-015770685.1	2273	CC17	115	CC2	5742	2017	USA: Pennsylvania	Rectal swab
GCF-015772285.1	17	CC17	115	CC2	6865	2017	USA: Pennsylvania	Rectal swab
GCF-015771465.1	17	CC17	115	CC2	6858	2017	USA: Pennsylvania	Urine
GCF-015768495.1	1471	CC17	115	CC2	5802	2018	USA: Pennsylvania	Rectal swab
GCF-015770305.1	1471	CC17	115	CC2	6849	2017	USA: Pennsylvania	Rectal swab
GCF-015770185.1	1471	CC17	115	CC2	6845	2017	USA: Pennsylvania	Rectal swab
GCF-015768285.1	1471	CC17	115	CC2	3712	2018	USA: Pennsylvania	Rectal swab
GCF-015770125.1	1471	CC17	115	CC2	3744	2017	USA: Pennsylvania	Rectal swab
GCF-015770695.1	1471	CC17	115	CC2	3744	2017	USA: Pennsylvania	Rectal swab
GCF-015770445.1	1471	CC17	115	CC2	3744	2017	USA: Pennsylvania	Rectal swab
GCF-015767995.1	1471	CC17	115	CC2	1001	2018	USA: Pennsylvania	Rectal swab
GCF-015772165.1	1471	CC17	115	CC2	4971	2017	USA: Pennsylvania	Rectal swab
GCF-015769785.1	1471	CC17	115	CC2	6842	2017	USA: Pennsylvania	Rectal swab
GCF-015769745.1	1471	CC17	115	CC2	5773	2017	USA: Pennsylvania	Rectal swab
GCF-015770735.1	1471	CC17	115	CC2	5646	2017	USA: Pennsylvania	Rectal swab
GCF-015771175.1	1471	CC17	115	CC2	5646	2017	USA: Pennsylvania	Rectal swab
GCF-015772455.1	1471	CC17	115	CC2	6867	2017	USA: Pennsylvania	Rectal swab
GCF-015768835.1	17	CC17	115	CC2	6836	2018	USA: Pennsylvania	Rectal swab
GCF-015769755.1	17	CC17	115	CC2	6836	2017	USA: Pennsylvania	Rectal swab
GCF-021228615.1	584	CC17	159	CC128	6796	2021	USA: Chicago	Blood
GCF-004300315.1	412	CC17	192	CC2	4816	2018	USA:Little Rock	Blood
GCF-001059995.1	412	CC17	192	CC2	797	n/a	USA:Washington	Clinical isolate
GCF-016406445.1	412	CC17	192	CC2	4028	2015	USA: Dallas	Rectal swab
GCF-015551885.1	412	CC17	192	CC2	4632	2018	USA	Stool
GCF-026947865.1	412	CC17	192	CC2	5740	2016	Belgium: Brussels	Clinical isolate
GCF-026948025.1	412	CC17	192	CC2	5740	2016	Belgium: Brussels	Clinical isolate
GCF-015773745.1	412	CC17	192	CC2	3723	2017	USA: Pennsylvania	Blood
GCF-015772995.1	412	CC17	192	CC2	5735	2017	USA: Pennsylvania	Rectal swab
GCF-015768485.1	412	CC17	192	CC2	5700	2018	USA: Pennsylvania	Rectal swab
GCF-015773285.1	412	CC17	192	CC2	5700	2017	USA: Pennsylvania	Rectal swab
GCF-015769235.1	412	CC17	192	CC2	5700	2018	USA: Pennsylvania	Rectal swab
GCF-015771205.1	412	CC17	192	CC2	5700	2017	USA: Pennsylvania	Rectal swab
GCF-004152605.1	412	CC17	192	CC2	4815	2018	USA: St. Louis	Clinical isolate
GCF-022469265.1	412	CC17	192	CC2	6919	2011	Israel: Jerusalem	Blood
GCF-022469255.1	412	CC17	192	CC2	6919	2011	Israel: Jerusalem	Blood
GCF-022464935.1	412	CC17	192	CC2	6911	2010	Israel: Jerusalem	Blood
GCF-022469525.1	412	CC17	192	CC2	6926	2012	Israel: Jerusalem	Blood
GCF-022469495.1	412	CC17	192	CC2	6924	2013	Israel: Jerusalem	Blood
GCF-022468925.1	412	CC17	193		6918	2018	Israel: Jerusalem	Blood
GCF-900639485.1	412	CC17	192	CC2	270	n/a	n/a	n/a
GCF-004300115.1	203	CC17	270		4781	2018	USA:Little Rock	Blood
GCF-004299965.1	203	CC17	270		4781	2018	USA:Little Rock	Blood
GCF-005234235.1	203	CC17	266	CC2	4784	2009	New Zealand	Rectal swab
GCF-005235395.1	203	CC17	266	CC2	4784	2008	New Zealand	Rectal swab
GCF-005234405.1	203	CC17	266	CC2	4784	2008	New Zealand	Rectal swab
GCF-000294975.2	494	CC17	192	CC2	732	n/a	n/a	n/a
GCF-015771145.1	412	CC17	197		6854	2017	USA: Pennsylvania	Rectal swab

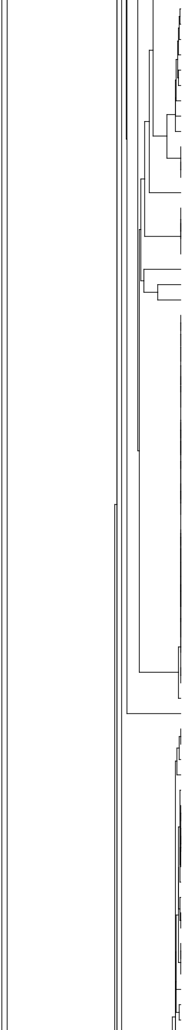

GCF-015770085.1	203	CC17	192	CC2	5765	2017	USA: Pennsylvania	Rectal swab
GCF-015771565.1	203	CC17	192	CC2	3057	2017	USA: Pennsylvania	Rectal swab
GCF-015768335.1	203	CC17	192	CC2	3057	2018	USA: Pennsylvania	Rectal swab
GCF-015769685.1	203	CC17	192	CC2	5765	2017	USA: Pennsylvania	Wound
GCF-015767955.1	203	CC17	192	CC2	3057	2018	USA: Pennsylvania	Rectal swab
GCF-015769175.1	203	CC17	192	CC2	3057	2018	USA: Pennsylvania	Rectal swab
GCF-015769635.1	203	CC17	192	CC2	4015	2018	USA: Pennsylvania	Urine
GCF-015771195.1	203	CC17	192	CC2	6855	2017	USA: Pennsylvania	Rectal swab
GCF-015768055.1	203	CC17	192	CC2	3057	2018	USA: Pennsylvania	Rectal swab
GCF-015772015.1	730	CC17	210	CC2	6830	2017	USA: Pennsylvania	Rectal swab
GCF-015768895.1	730	CC17	210	CC2	6830	2018	USA: Pennsylvania	Rectal swab
GCF-015767985.1	730	CC17	210	CC2	6830	2018	USA: Pennsylvania	Rectal swab
GCF-022469555.1	412	CC17	196		6927	2010	Israel: Jerusalem	Blood
GCF-009078415.1	896		191		4870	2012	Brazil: Sao Jose do Rio Preto	Broncoalveolar lavage
GCF-008921805.1	896		191		4870	2012	Brazil: Sao Jose do Rio Preto	Muscle biopsy
GCF-009078405.1	896		191		4870	2012	Brazil: Sao Jose do Rio Preto	Ascitic Fluid
GCF-008922055.1	896		190		4870	2012	Brazil: Sao Jose do Rio Preto	Urine
GCF-003350625.1	734	CC17	728		4842	2011	USA: Utah	Blood
GCF-015769545.1	1474		195		5820	2018	USA: Pennsylvania	Rectal swab
GCF-000294875.2	412	CC17	194	CC184	750	n/a	n/a	n/a
GCF-900178555.1	78	CC17	188	CC2	4164	2015	United Kingdom	Human
GCF-900178595.1	78	CC17	188	CC2	4164	2015	United Kingdom	Human
GCF-900178975.1	78	CC17	188	CC2	4164	2015	United Kingdom	Human
GCF-900178815.1	78	CC17	188	CC2	4164	2015	United Kingdom	Human
GCF-900178725.1	78	CC17	188	CC2	4164	2015	United Kingdom	Human
GCF-900178925.1	78	CC17	188	CC2	4164	2015	United Kingdom	Human
GCF-900179295.1	78	CC17	188	CC2	4164	2015	United Kingdom	Human
GCF-900178935.1	78	CC17	188	CC2	4164	2015	United Kingdom	Human
GCF-900178745.1	78	CC17	188	CC2	4164	2015	United Kingdom	Human
GCF-900178785.1	78	CC17	188	CC2	4164	2015	United Kingdom	Human
GCF-900178835.1	78	CC17	188	CC2	4164	2015	United Kingdom	Human
GCF-900179085.1	78	CC17	188	CC2	4164	2015	United Kingdom	Human
GCF-900178915.1	78	CC17	188	CC2	4164	2015	United Kingdom	Human
GCF-900178765.1	78	CC17	188	CC2	4164	2015	United Kingdom	Human
GCF-900178985.1	78	CC17	188	CC2	4164	2015	United Kingdom	Human
GCF-900180175.1	78	CC17	188	CC2	4164	2015	United Kingdom	Human
GCF-900178625.1	78	CC17	188	CC2	4164	2015	United Kingdom	Human
GCF-900178855.1	78	CC17	188	CC2	4164	2015	United Kingdom	Human
GCF-900178755.1	78	CC17	188	CC2	4164	2015	United Kingdom	Human
GCF-900178905.1	78	CC17	188	CC2	4164	2015	United Kingdom	Human
GCF-900178715.1	78	CC17	188	CC2	4164	2015	United Kingdom	Human
GCF-900178875.1	78	CC17	188	CC2	4164	2015	United Kingdom	Human
GCF-900179055.1	78	CC17	188	CC2	4164	2015	United Kingdom	Human
GCF-900178845.1	78	CC17	188	CC2	4164	2015	United Kingdom	Human
GCF-900179785.1	78	CC17	188	CC2	4164	2015	United Kingdom	Human
GCF-900179915.1	78	CC17	188	CC2	4164	2015	United Kingdom	Human
GCF-003957785.1	203	CC17	200		3232	2016	Australia: Brisbane	Urine
GCF-004152105.1	736	CC17	118	CC2	4845	2018	USA: St. Louis	Clinical isolate
GCF-004152175.1	736	CC17	118	CC2	4845	2018	USA: St. Louis	Clinical isolate
GCF-004151995.1	736	CC17	118	CC2	4845	2018	USA: St. Louis	Clinical isolate
GCF-004152285.1	736	CC17	158	CC128	4846	2018	USA: St. Louis	Clinical isolate
GCF-015770325.1	736	CC17	118	CC2	5724	2017	USA: Pennsylvania	Rectal swab
GCF-015768425.1	736	CC17	118	CC2	3713	2018	USA: Pennsylvania	Rectal swab
GCF-015772055.1	736	CC17	118	CC2	3713	2017	USA: Pennsylvania	Rectal swab
GCF-015768975.1	736	CC17	118	CC2	3713	2018	USA: Pennsylvania	Rectal swab
GCF-015769555.1	736	CC17	118	CC2	3713	2018	USA: Pennsylvania	Rectal swab
GCF-015771495.1	736	CC17	118	CC2	3713	2017	USA: Pennsylvania	Rectal swab
GCF-015768785.1	736	CC17	118	CC2	3713	2018	USA: Pennsylvania	Urine
GCF-015768585.1	736	CC17	118	CC2	3713	2018	USA: Pennsylvania	Rectal swab
GCF-015769515.1	736	CC17	118	CC2	3713	2018	USA: Pennsylvania	Rectal swab
GCF-015772435.1	736	CC17	118	CC2	3713	2017	USA: Pennsylvania	Rectal swab
GCF-015768255.1	736	CC17	118	CC2	5724	2018	USA: Pennsylvania	Rectal swab
GCF-015769455.1	736	CC17	118	CC2	5724	2018	USA: Pennsylvania	Rectal swab
GCF-015770675.1	736	CC17	118	CC2	5724	2017	USA: Pennsylvania	Rectal swab
GCF-015770075.1	736	CC17	118	CC2	3721	2017	USA: Pennsylvania	Rectal swab
GCF-015771785.1	736	CC17	118	CC2	5636	2017	USA: Pennsylvania	Rectal swab
GCF-015772515.1	736	CC17	118	CC2	5636	2017	USA: Pennsylvania	Rectal swab

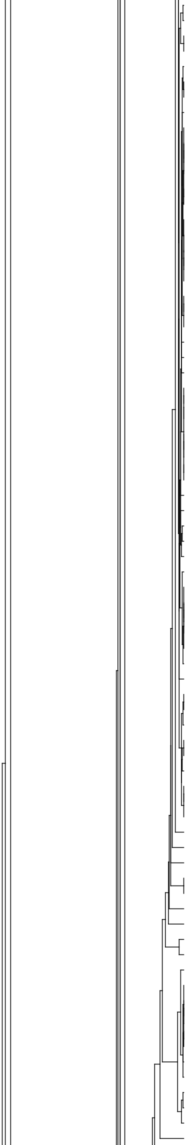

GCF-016406385.1	736	CC17	120		5782	2015	USA: Dallas	Rectal swab
GCF-004152305.1	736	CC17	120		5782	2018	USA: St. Louis	Clinical isolate
GCF-000295575.2	736	CC17	118	CC2	722	n/a	n/a	n/a
GCF-000294815.2	736	CC17	118	CC2	722	n/a	n/a	n/a
GCF-001720985.1	736	CC17	118	CC2	722	2011	USA:New York City	Blood
GCF-001953235.1	736	CC17	118	CC2	722	2011	USA: New York	Blood
GCF-001953255.1	736	CC17	118	CC2	722	2011	USA: New York	Blood
GCF-001721025.1	736	CC17	118	CC2	722	2011	USA:New York City	Blood
GCF-000394755.1	736	CC17	118	CC2	722	n/a	n/a	n/a
GCF-000394655.1	736	CC17	118	CC2	722	n/a	n/a	n/a
GCF-000394675.1	736	CC17	118	CC2	722	n/a	n/a	n/a
GCF-000394635.1	736	CC17	118	CC2	722	n/a	n/a	n/a
GCF-000394735.1	736	CC17	118	CC2	722	n/a	n/a	n/a
GCF-000394695.1	736	CC17	118	CC2	722	n/a	n/a	n/a
GCF-000407065.1	736	CC17	118	CC2	722	n/a	n/a	n/a
GCF-000407085.1	736	CC17	118	CC2	722	n/a	n/a	n/a
GCF-000407325.1	736	CC17	118	CC2	722	n/a	n/a	n/a
GCF-000407105.1	736	CC17	118	CC2	722	n/a	n/a	n/a
GCF-000407345.1	736	CC17	118	CC2	722	n/a	n/a	n/a
GCF-002761275.1	736	CC17	118	CC2	722	2013	USA: New York	Peritoneal fluid
GCF-002761255.1	736	CC17	118	CC2	722	2013	USA: New York	Peritoneal fluid
GCF-002777275.1	736	CC17	118	CC2	722	2013	USA: New York	Wound
GCF-004152065.1	736	CC17	118	CC2	722	2018	USA: St. Louis	Clinical isolate
GCF-001058945.1	736	CC17	118	CC2	722	n/a	USA:Washington	Clinical isolate
GCF-001055235.1	736	CC17	118	CC2	722	n/a	USA:Washington	Clinical isolate
GCF-002848625.1	736	CC17	118	CC2	722	2015	USA: New York	Blood
GCF-002848645.1	736	CC17	118	CC2	722	2015	USA: New York	Blood
GCF-002848685.1	736	CC17	118	CC2	722	2015	USA: New York	Blood
GCF-002848665.1	736	CC17	118	CC2	6777	2015	USA: New York	Blood
GCF-002848705.1	736	CC17	118	CC2	6777	2015	USA: New York	Blood
GCF-002848725.1	736	CC17	118	CC2	6777	2015	USA: New York	Blood
GCF-002848745.1	736	CC17	118	CC2	6777	2015	USA: New York	Blood
GCF-004299865.1	736	CC17	118	CC2	722	2018	USA:Little Rock	Blood
GCF-001721105.1	736	CC17	118	CC2	5026	2011	USA:New York City	Blood
GCF-001635875.1	736	CC17	118	CC2	722	2009	USA: New York	Blood
GCF-002880635.1	736	CC17	118	CC2	3704	2009	USA	n/a
GCF-022468915.1	736	CC17	118	CC2	3704	2018	Israel: Jerusalem	Blood
GCF-015770105.1	736	CC17	118	CC2	3728	2017	USA: Pennsylvania	Rectal swab
GCF-015771515.1	736	CC17	118	CC2	3728	2017	USA: Pennsylvania	Rectal swab
GCF-015768945.1	736	CC17	118	CC2	3728	2018	USA: Pennsylvania	Rectal swab
GCF-015772215.1	736	CC17	118	CC2	3728	2017	USA: Pennsylvania	Rectal swab
GCF-015772335.1	736	CC17	118	CC2	3728	2017	USA: Pennsylvania	Rectal swab
GCF-015769735.1	736	CC17	118	CC2	3728	2017	USA: Pennsylvania	Rectal swab
GCF-015771215.1	736	CC17	118	CC2	3728	2017	USA: Pennsylvania	Rectal swab
GCF-001721065.1	736	CC17	118	CC2	4000	2011	USA:New York City	Blood
GCF-004300195.1	736	CC17	118	CC2	4847	2018	USA:Little Rock	Blood
GCF-004299975.1	736	CC17	118	CC2	4847	2018	USA:Little Rock	Blood
GCF-004299885.1	736	CC17	118	CC2	4847	2018	USA:Little Rock	Blood
GCF-004300355.1	736	CC17	118	CC2	4847	2018	USA:Little Rock	Blood
GCF-004299875.1	736	CC17	118	CC2	4847	2018	USA:Little Rock	Blood
GCF-004299905.1	736	CC17	118	CC2	722	2018	USA:Little Rock	Blood
GCF-022647825.1	736	CC17	118	CC2	4847	2019	USA:Little Rock	Blood
GCF-022647945.1	736	CC17	118	CC2	4847	2019	USA:Little Rock	Blood
GCF-022648125.1	736	CC17	118	CC2	4847	2019	USA:Little Rock	Blood
GCF-001056745.1	736	CC17	118	CC2	752	n/a	USA:Washington	Clinical isolate
GCF-015770255.1	736	CC17	118	CC2	6848	2017	USA: Pennsylvania	Wound
GCF-015768555.1	736	CC17	118	CC2	6834	2018	USA: Pennsylvania	Rectal swab
GCF-004151975.1	736	CC17	118	CC2	4844	2018	USA: St. Louis	Clinical isolate
GCF-004151965.1	736	CC17	118	CC2	4844	2018	USA: St. Louis	Clinical isolate
GCF-000294935.2	664	CC17	118	CC2	747	n/a	n/a	n/a
GCF-004300375.1	448	CC17	182	CC2	4824	2018	USA:Little Rock	Blood
GCF-015770495.1	584	CC17	118	CC2	6851	2017	USA: Pennsylvania	Rectal swab
GCF-015770575.1	584	CC17	118	CC2	3741	2017	USA: Pennsylvania	Rectal swab
GCF-018219305.1	664	CC17	114		3551	2016	USA: Ann Arbor	Rectal swab
GCF-003350805.1	664	CC17	114		4839	2012	USA: Utah	Blood
GCF-003350785.1	664	CC17	114		4839	2012	USA: Utah	Blood
GCF-002158275.1	664	CC17	114		4839	2012	USA: Utah	Blood
GCF-002158285.1	664	CC17	114		4839	2013	USA: Utah	Blood
GCF-003350765.1	664	CC17	114		4839	2012	USA: Utah	Blood
GCF-002158385.1	664	CC17	114		4839	2014	USA: Utah	Blood
GCF-002158295.1	664	CC17	114		4839	2013	USA: Utah	Blood
GCF-016406505.1	664	CC17	114		5779	2015	USA: Dallas	Rectal swab
GCF-016415505.1	664	CC17	114		3776	2015	USA: Dallas	Rectal swab
GCF-016406605.1	664	CC17	114		5779	2015	USA: Dallas	Rectal swab
GCF-004300445.1	280	CC17	119		4796	2018	USA:Little Rock	Blood

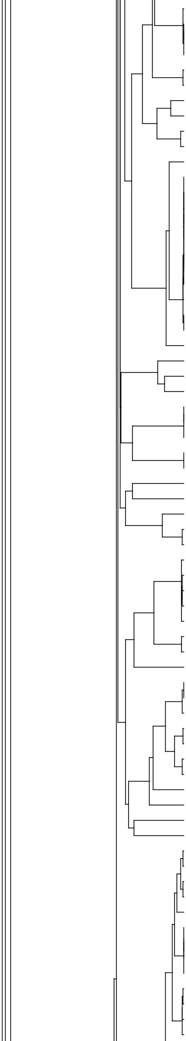

GCF-900179115.1	280	CC17	112	CC2	4185	2015	United Kingdom	Human
GCF-900178565.1	280	CC17	112	CC2	4185	2015	United Kingdom	Human
GCF-900178995.1	280	CC17	112	CC2	4185	2015	United Kingdom	Human
GCF-900179435.1	280	CC17	112	CC2	4185	2015	United Kingdom	Human
GCF-010119315.1	17	CC17	831	CC152	3439	2018	USA: Chicago	Blood
GCF-010119375.1	17	CC17	831	CC152	3439	2018	USA: Chicago	Blood
GCF-023204955.1	752	CC17	55		6803	2019	USA	Blood
GCF-000294895.2	752	CC17	46	CC2	785	n/a	n/a	n/a
GCF-022691445.1	280	CC17	46	CC2	6800	2004	USA	Urine
GCF-022693745.1	280	CC17	46	CC2	6800	2004	USA	Urine
GCF-002894565.1	80	CC17	141	CC128	120	2009	Saudi Arabia: Riyadh	Clinical isolate
GCF-005235575.1	375	CC17	141	CC128	4808	2007	New Zealand	Clinical isolate
GCF-005235645.1	375	CC17	141	CC128	4808	2007	New Zealand	Rectal swab
GCF-005235635.1	375	CC17	141	CC128	4808	2007	New Zealand	Rectal swab
GCF-005235045.1	375	CC17	141	CC128	4808	2008	New Zealand	Rectal swab
GCF-005235665.1	375	CC17	141	CC128	4808	2007	New Zealand	Rectal swab
GCF-005235685.1	375	CC17	141	CC128	4808	2007	New Zealand	Tissue
GCF-005234505.1	375	CC17	141	CC128	4808	2007	New Zealand	Rectal swab
GCF-005234295.1	375	CC17	141	CC128	4808	2008	New Zealand	Rectal swab
GCF-005235285.1	375	CC17	141	CC128	4808	2008	New Zealand	Rectal swab
GCF-005235075.1	375	CC17	141	CC128	4808	2008	New Zealand	Rectal swab
GCF-005235015.1	375	CC17	141	CC128	4808	2008	New Zealand	Rectal swab
GCF-026945195.1	80	CC17	1026	CC2	1451	2011	Belgium: Leuven	Blood
GCF-000390485.1	18	CC17	572	CC2	727	2005	Netherlands	Blood
GCF-000321725.1	64	CC17	514	CC128	507	2000	United Kingdom	Clinical isolate
GCF-026929615.1	18	CC17	514	CC128	4227	2019	Belgium: Wilrijk	n/a
GCF-003938285.1	2329	CC17	750	CC2	4911	2016	Pakistan	Light switch in hospital intensive care unit
GCF-003938485.1	2329	CC17	750	CC2	4911	2016	Pakistan	Rail in hospital intensive care unit
GCF-003938345.1	2329	CC17	750	CC2	4911	2016	Pakistan	Alcohol foam dispenser in hospital intensive care unit
GCF-000396845.1	20	CC17	586	CC2	762	1996	USA: New York	n/a
GCF-000395685.1	20	CC17	586	CC2	762	1996	USA: New York	n/a
GCF-005234245.1	18	CC17	132	CC128	4699	2009	New Zealand	Rectal swab
FI403	787	CC17	66	CC7		2019	Czech republic: Brno	Stool
GCF-009036045.1	323	CC17	203	CC128	6782	2015	Brazil: Parana	n/a
GCF-900639635.1	598	CC17	211	CC184	338	n/a	n/a	n/a
GCF-026945825.1	598	CC17	211	CC184	338	2018	Belgium: Seraing	Rectal swab
GCF-000148325.1	17	CC17	602	CC2	704	n/a	n/a	n/a
GCF-000148145.1	17	CC17	602	CC2	704	n/a	n/a	n/a
GCF-000148285.1	17	CC17	602	CC2	704	n/a	n/a	n/a
GCF-000147235.1	17	CC17	602	CC2	704	n/a	n/a	n/a
GCF-000147315.1	17	CC17	602	CC2	704	n/a	n/a	n/a
GCF-000295155.2	280	CC17	814	CC2	763	n/a	n/a	n/a
GCF-000295035.2	280	CC17	814	CC2	763	n/a	n/a	n/a
GCF-015772405.1	750	CC17	181	CC2	6866	2017	USA: Pennsylvania	Rectal swab
GCF-000396925.1	16	CC17	594	CC36	751	1996	USA: New York	n/a
GCF-000394575.1	16	CC17	594	CC36	751	1996	USA: New York	n/a
GCF-000394595.1	16	CC17	594	CC36	751	1996	USA: New York	n/a
GCF-026947425.1	16	CC17	13	CC2	1204	2003	Belgium: Ieper	Blood
GCF-026947345.1	16	CC17	13	CC2	1204	2004	Belgium: Brussels	Blood
GCF-001750885.1	16	CC17	13	CC2	943	2000	Netherlands: Utrecht	n/a
GCF-002442955.1	16	CC17	13	CC2	943	2002	Netherlands: Utrecht	Stool
GCF-900639745.1	16	CC17	2	CC2	812	n/a	n/a	n/a
GCF-026073115.1	16	CC17	1014	CC36		2000	Japan	Sputum
GCF-000321665.1	117	CC17	558	CC2	346	2001	USA	Stool
GCF-003937555.1	2086	CC17	747	CC128	4910	2016	Pakistan	Nursing call button in hospital intensive care unit
GCF-026948185.1	80	CC17	122	CC152	1201	2015	Belgium: Boussu	Blood
GCF-004152325.1	80	CC17	122	CC152	1201	2014	Pakistan: Islamabad	Clinical isolate
GCF-026943515.1	80	CC17	122	CC152		2018	Belgium: Boussu	Blood
GCF-026943945.1	80	CC17	122	CC152	1201	2018	Belgium: Boussu	Rectal swab
GCF-017815695.1	80	CC17	122	CC152	2748	2019	India	Blood
GCF-026946325.1	80	CC17	122	CC152	2955	2018	Belgium: Mons	Rectal swab
GCF-026946385.1	80	CC17	122	CC152	2955	2018	Belgium: Mons	Rectal swab
GCF-026946485.1	80	CC17	122	CC152	2955	2018	Belgium: Mons	Rectal swab
GCF-026946365.1	80	CC17	122	CC152	2955	2018	Belgium: Mons	Rectal swab
GCF-026941595.1	80	CC17	122	CC152	6182	2017	Belgium: Leuven	Clinical isolate
GCF-026941725.1	80	CC17	122	CC152	6182	2017	Belgium: Leuven	Rectal swab
GCF-026948075.1	80	CC17	122	CC152	1866	2016	Belgium: Leuven	Rectal swab
GCF-026942315.1	80	CC17	122	CC152	6182	2017	Belgium: Leuven	Rectal swab

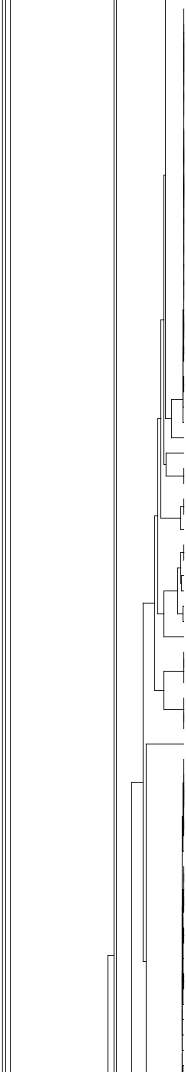

GCF-003940995.1	2100	CC17	746	CC128	1954	2016	Pakistan	Light switch in hospital intensive care unit
GCF-003939175.1	132	CC17	746	CC128	1954	2016	Pakistan	Alcohol foam dispenser in hospital intensive care unit
GCF-003938105.1	132	CC17	746	CC128	1954	2016	Pakistan	Nursing call button in hospital intensive care unit
GCF-003937355.1	132	CC17	746	CC128	1954	2016	Pakistan	Rail in hospital intensive care unit
GCF-003938515.1	132	CC17	746	CC128	1954	2016	Pakistan	Sink in hospital intensive care unit
GCF-003938305.1	132	CC17	746	CC128	1954	2016	Pakistan	Sink in hospital intensive care unit
GCF-003938045.1	132	CC17	746	CC128	1954	2016	Pakistan	Alcohol foam dispenser in hospital intensive care unit
GCF-003939215.1	132	CC17	746	CC128	1954	2016	Pakistan	Nursing call button in hospital intensive care unit
GCF-003940245.1	132	CC17	746	CC128	1954	2016	Pakistan	Nursing call button in hospital intensive care unit
GCF-003939705.1	132	CC17	746	CC128	1954	2016	Pakistan	Alcohol foam dispenser in hospital intensive care unit
GCF-003939145.1	132	CC17	746	CC128	1954	2016	Pakistan	Light switch in hospital intensive care unit
GCF-003938495.1	132	CC17	746	CC128	1954	2016	Pakistan	Light switch in hospital intensive care unit
GCF-003939165.1	132	CC17	746	CC128	1954	2016	Pakistan	Rail in hospital intensive care unit
GCF-003965125.1	132	CC17	746	CC128	1954	2016	Pakistan	Sink in hospital intensive care unit
GCF-003938365.1	132	CC17	746	CC128	1954	2016	Pakistan	Nursing call button in hospital intensive care unit
GCF-003938505.1	132	CC17	746	CC128	1954	2016	Pakistan	Nursing call button in hospital intensive care unit
GCF-003938065.1	2100	CC17	746	CC128	1954	2016	Pakistan	Nursing call button in hospital intensive care unit
GCF-003938265.1	132	CC17	746	CC128	1954	2016	Pakistan	Sink in hospital intensive care unit
GCF-003937575.1	2100	CC17	746	CC128	1954	2016	Pakistan	Rail in hospital intensive care unit
GCF-003937935.1	2100	CC17	746	CC128	1954	2016	Pakistan	Rail in hospital intensive care unit
GCF-003938395.1	132	CC17	746	CC128	1954	2016	Pakistan	Alcohol foam dispenser in hospital intensive care unit
GCF-003940215.1	132	CC17	746	CC128	1954	2016	Pakistan	Rail in hospital intensive care unit
GCF-003936585.1	132	CC17	746	CC128	1954	2016	Pakistan	Alcohol foam dispenser in hospital intensive care unit
GCF-003938445.1	132	CC17	746	CC128	1954	2016	Pakistan	Rail in hospital intensive care unit
GCF-003937285.1	132	CC17	746	CC128	1954	2016	Pakistan	Light switch in hospital intensive care unit
GCF-003936615.1	132	CC17	746	CC128	1954	2016	Pakistan	Nursing call button in hospital intensive care unit
GCF-003937635.1	132	CC17	746	CC128	1954	2016	Pakistan	Light switch in hospital intensive care unit
GCF-003938375.1	132	CC17	746	CC128	1954	2016	Pakistan	Sink in hospital intensive care unit
GCF-003939775.1	132	CC17	746	CC128	4723	2016	Pakistan	Light switch in hospital intensive care unit
GCF-003940185.1	80	CC17	746	CC128	2341	2016	Pakistan	Alcohol foam dispenser in hospital intensive care unit
GCF-026943565.1	80	CC17	1013	CC152	6187	2018	Belgium: Leuven	Rectal swab
GCF-026944365.1	80	CC17	1013	CC152	6187	2018	Belgium: Leuven	Rectal swab
GCF-003938125.1	80	CC17	144	CC152	3412	2016	Pakistan	Rail in hospital intensive care unit
GCF-003939205.1	80	CC17	144	CC152	3412	2016	Pakistan	Rail in hospital intensive care unit
GCF-003936605.1	80	CC17	144	CC152	3410	2016	Pakistan	Sink in hospital intensive care unit
FI514	761	CC17	7	CC7	6766	2022	Czech republic: Brno	Rectal swab
FI515	761	CC17	7	CC7	6766	2022	Czech republic: Brno	Urine
FI436	761	CC17	7	CC7	6766	2019	Czech republic: Brno	Rectal swab
FI490	761	CC17	7	CC7	6766	2019	Czech republic: Brno	Rectal swab
GCF-003940205.1	761	CC17	751	CC7		2016	Pakistan	Alcohol foam dispenser in hospital intensive care unit
GCF-003939805.1	761	CC17	751	CC7	2306	2016	Pakistan	Rail in hospital intensive care unit
GCF-021311175.1	761	CC17	5	CC7	6888	2019	Reunion: Saint-Denis	Rectal swab
GCF-012933055.2	80	CC17	148	CC152	3448	2019	India:Vellore	Blood
GCF-017898025.1	80	CC17	148	CC152	3448	2019	India	Blood
GCF-014874615.1	80	CC17	148	CC152	3448	2019	India	Blood
GCF-012933245.2	80	CC17	144	CC152	3449	2019	India:Vellore	Blood
GCF-012933165.2	80	CC17	144	CC152	3449	2019	India:Vellore	Blood
GCF-012933075.2	80	CC17	144	CC152	3449	2017	India:Vellore	Blood
GCF-003020705.1	78	CC17	125	CC184	4096	2015	Australia	Clinical isolate
GCF-026944785.1	80	CC17	102	CC2	1565	2017	Belgium: Godinne	Rectal swab
GCF-026941305.1	80	CC17	102	CC2	1565	2017	Belgium: Godinne	Rectal swab
GCF-026945125.1	80	CC17	102	CC2	1565	2017	Belgium: Godinne	Rectal swab
GCF-026945025.1	80	CC17	102	CC2	1565	2017	Belgium: Godinne	Rectal swab
GCF-026944945.1	80	CC17	102	CC2	1565	2017	Belgium: Godinne	Rectal swab
GCF-026945035.1	80	CC17	102	CC2	1565	2017	Belgium: Godinne	Rectal swab
GCF-026944805.1	80	CC17	102	CC2	1565	2017	Belgium: Godinne	Rectal swab
GCF-026943525.1	80	CC17	102	CC2	1565	2018	Belgium: Verviers	Clinical isolate
GCF-026942325.1	80	CC17	102	CC2	1565	2018	Belgium: Liege	Pus
GCF-026945625.1	80	CC17	102	CC2	1565	2018	Belgium: Seraing	Rectal swab
GCF-026946085.1	80	CC17	102	CC2	1565	2018	Belgium: Liege	Urine
GCF-026946065.1	80	CC17	102	CC2	1565	2018	Belgium: Liege	Rectal swab
GCF-026945785.1	80	CC17	102	CC2	1565	2018	Belgium: Seraing	Rectal swab
GCF-026946185.1	80	CC17	102	CC2	1565	2018	Belgium: Liege	Rectal swab
GCF-026945635.1	80	CC17	102	CC2	1565	2018	Belgium: Seraing	Rectal swab
GCF-026945465.1	80	CC17	102	CC2	1565	2018	Belgium: Seraing	Urine
GCF-026946145.1	80	CC17	102	CC2	1565	2018	Belgium: Liege	Rectal swab
GCF-026942385.1	80	CC17	102	CC2	1565	2017	Belgium: Liege	Urine
GCF-026942685.1	80	CC17	102	CC2	1565	2018	Belgium: Liege	Urine
GCF-026945565.1	80	CC17	102	CC2	1565	2018	Belgium: Seraing	Rectal swab
GCF-026945925.1	80	CC17	1009		1565	2018	Belgium: Liege	Rectal swab

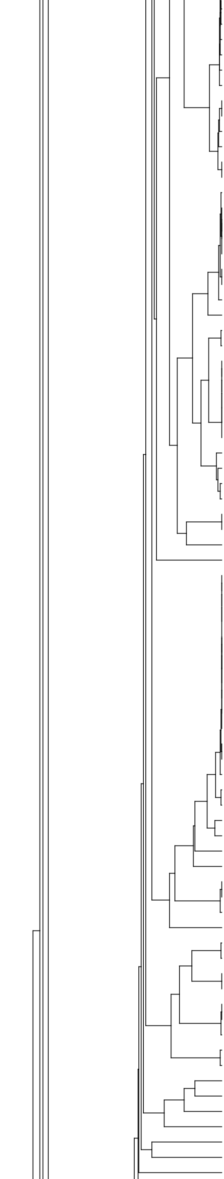

GCF-026946005.1	80	CC17	102	CC2	1565	2018	Belgium: Liege	Rectal swab
GCF-026946195.1	80	CC17	102	CC2	1565	2018	Belgium: Liege	Rectal swab
GCF-026943485.1	80	CC17	102	CC2	1565	2018	Belgium: Liege	Urine
GCF-026942985.1	80	CC17	102	CC2	1565	2017	Belgium: Liege	Urine
GCF-026945885.1	80	CC17	102	CC2	1565	2018	Belgium: Seraing	Rectal swab
GCF-026942915.1	80	CC17	102	CC2	1565	2018	Belgium: Liege	Urine
GCF-012932985.2	80	CC17	102	CC2	3446	2017	India:Vellore	Blood
GCF-012932975.2	80	CC17	102	CC2	3413	2018	India:Vellore	Blood
GCF-003938275.1	80	CC17	102	CC2	3413	2016	Pakistan	Rail in hospital intensive care unit
GCF-026304695.1	80	CC17	102	CC2		2016	China:Liaoning province	Blood
GCF-026941385.1	80	CC17	102	CC2		2017	Belgium: Brussels	Stool
GCF-026941405.1	80	CC17	102	CC2	3446	2017	Belgium: Brussels	Rectal swab
GCF-026308655.1	78	CC17	79		6877	2014	China:Liaoning province	Drainage
GCF-026305515.1	78	CC17	79		6877	2014	China:Liaoning province	Secretion
GCF-026308995.1	78	CC17	79		1746	2013	China:Liaoning province	Urine
GCF-026309055.1	78	CC17	79		1746	2013	China:Liaoning province	Urine
GCF-026309135.1	78	CC17	79		1746	2013	China:Liaoning province	Urine
GCF-019734135.1	78	CC17	79		6877	2020	China	Stool
GCF-019734085.1	78	CC17	79		6877	2020	China	Stool
GCF-002848385.1	78	CC17	79		1746	2016	China	Fooder
GCF-003719365.1	78	CC17	732		4767	2015	China:Sichuan	Clinical isolate
GCF-010120755.1	78	CC17	171		3398	2017	China:Hangzhou	Clinical isolate
GCF-006007925.1	78	CC17	171		3398	2018	China:Hangzhou	Urine
GCF-019733965.1	78	CC17	172		6875	2020	China	Stool
GCF-019734075.1	78	CC17	172		6875	2020	China	Stool
GCF-019733825.1	78	CC17	172		6875	2020	China	Stool
GCF-019731665.1	78	CC17	172		6875	2020	China	Stool
GCF-019731655.1	78	CC17	172		6875	2020	China	Stool
GCF-019734055.1	78	CC17	172		6875	2020	China	Stool
GCF-026304855.1	78	CC17	1049			2016	China:Liaoning province	Ascites
GCF-026305475.1	78	CC17	1047			2014	China:Liaoning province	Ascites
GCF-026304705.1	78	CC17	1049			2016	China:Liaoning province	Urine
GCF-026304885.1	78	CC17	1049			2016	China:Liaoning province	Urine
GCF-009733995.1	78	CC17	109		3433	2018	China:Hangzhou	Clinical isolate
GCF-009734005.1	78	CC17	109		3433	2017	China:Hangzhou	Clinical isolate
GCF-009938075.1	78	CC17	145	CC152	3438	2017	China: Hangzhou	Urine
GCF-026309245.1	363	CC17	1032			2012	China:Liaoning province	Ascites
GCF-900179105.1	80	CC17	252	CC2	993	2015	United Kingdom	Human
GCF-900179075.1	80	CC17	252	CC2	993	2015	United Kingdom	Human
GCF-900180195.1	80	CC17	252	CC2	993	2015	United Kingdom	Human
GCF-900179935.1	80	CC17	252	CC2	993	2015	United Kingdom	Human
GCF-900179065.1	80	CC17	252	CC2	993	2015	United Kingdom	Human
GCF-900180465.1	80	CC17	252	CC2	993	2015	United Kingdom	Human
GCF-900180185.1	80	CC17	252	CC2	993	2015	United Kingdom	Human
GCF-900179095.1	80	CC17	252	CC2	993	2015	United Kingdom	Human
GCF-900179215.1	80	CC17	252	CC2	993	2015	United Kingdom	Human
GCF-900180325.1	80	CC17	252	CC2	993	2015	United Kingdom	Human
GCF-900180115.1	80	CC17	252	CC2	993	2015	United Kingdom	Human
GCF-900180165.1	80	CC17	252	CC2	993	2015	United Kingdom	Human
GCF-900179925.1	80	CC17	252	CC2	993	2015	United Kingdom	Human
GCF-026942285.1	80	CC17	252	CC2	993	2017	Belgium: Brussels	Stool
GCF-012933285.2	80	CC17	252	CC2	3454	2019	India:Vellore	Blood
GCF-017603725.1	80	CC17	252	CC2	3454	2020	India	Blood
GCF-004368135.1	80	CC17	658		3420	2013	France: Boulogne-Billancourt	Rectal swab
GCF-002973715.1	80	CC17	658		4770	2015	France: Narbonne	Rectal swab
GCF-022468105.1	80	CC17	149	CC152	6913	2016	Israel: Jerusalem	Blood
GCF-022463275.1	117	CC17	106	CC2	2094	2020	Israel: Jerusalem	Blood
GCF-012933345.2	80	CC17	106	CC2	1557	2019	India:Vellore	Blood
GCF-021311345.1	80	CC17	106	CC2	1557	2019	Reunion: Saint-Denis	Rectal swab
GCF-027286245.1	80	CC17	106	CC2	1557	2022	China: Zhuhai	Urine
GCF-002973755.2	17	CC17	251	CC2	3432	2015	France: Corbeille-Essonnes	Rectal swab
GCF-020736585.1	976		164	CC162	6795	2018	China:wenzhou	n/a
GCF-020736625.1	976		164	CC162	6795	2018	China:wenzhou	n/a
GCF-019733855.1	17	CC17	163	CC162	6876	2020	China	Stool
GCF-019733915.1	17	CC17	163	CC162	6876	2020	China	Stool
GCF-026934465.1	80	CC17	1030	CC152	6179	2019	Belgium: Wilrijk	n/a
GCF-026934965.1	80	CC17	1030	CC152	6179	2017	Belgium: Leuven	Rectal swab
GCF-026953915.1	80	CC17	1030	CC152	6179	2019	Belgium: Wilrijk	n/a
GCF-002630965.1	2305	CC17	654		5019	2015	Russia: St.Petersburg	Wound
GCF-002562815.1	2305	CC17	654		5019	2016	Russia: St.Petersburg	Wound
GCF-002894545.1	546		706	CC2	121	2009	Saudi Arabia: Riyadh	Clinical isolate
GCF-025599075.1	31	CC17	1037	CC2	5917	2020	India: Puducherry	Pus
GCF-026952785.1	280	CC17	1037	CC2	6188	2018	Belgium: Brussels	Rectal swab
FI489	17	CC17	105	CC2		2019	Czech republic: Brno	Respiratory tract secretion
GCF-018517185.1	1586	CC17	231	CC184	6786	2018	France: Tourcoing	Digestive
GCF-026947765.1	17	CC17	1006	CC2	1200	2012	Belgium: Edegem	Blood
GCF-001587115.1	904	CC17	262	CC2	739	2010	Germany	n/a

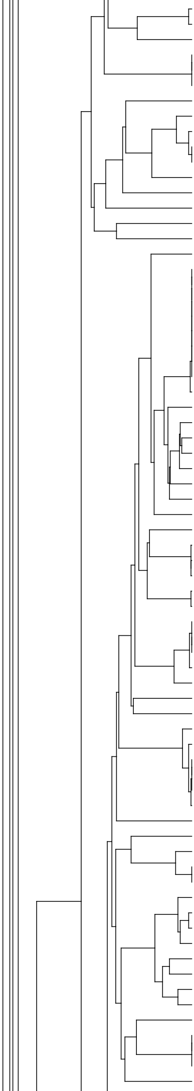

GCF-026305405.1	414	CC17	609		95	2014	China:Liaoning province	Blood
GCF-000934365.1	414	CC17	609		95	2013	China: Beijing	Urine
GCF-026308935.1	78	CC17	1022	CC60		2013	China:Liaoning province	Urine
FI442	787	CC17	94		6767	2019	Czech republic: Brno	Rectal swab
FI444	787	CC17	94		6767	2019	Czech republic: Brno	Rectal swab
FI443	787	CC17	94		6767	2019	Czech republic: Brno	Rectal swab
GCF-000322345.1	78	CC17	577	CC2	365	1999	Italy	Drainage
GCF-000395605.1	50	CC17	585	CC184	796	1993	France: Hopital Saint-Louis	n/a
GCF-000390465.1	50	CC17	561		127	n/a	Italy	Blood
GCF-000397045.1	50	CC17	561		127	1996	France: Hopital Toulouse	n/a
GCF-000393675.1	50	CC17	561		127	1996	France: Hopital Toulouse	n/a
GCF-900639655.1	266		177	CC184	436	n/a	n/a	n/a
GCF-900638825.1	398	CC17	184	CC184	801	n/a	n/a	n/a
GCF-002174435.1	413		540	CC73	1437	2015	Germany	Stool
GCF-900639405.1	456	CC17	93	CC184	827	n/a	n/a	n/a
GCF-000321985.1	92	CC17	568	CC62	493	1965	Netherlands	Stomach
GCF-000321765.1	21	CC17	562	CC36	145	1994	Belgium	Rumen
GCF-000262105.1	160	CC17	33	CC36	89	2011	China	Broncoalveolar lavage
GCF-009735345.1	160	CC17	33	CC36	89	n/a	n/a	n/a
GCF-013867835.1	160	CC17	33	CC36	89	2020	n/a	n/a
GCF-000411035.1	160	CC17	33	CC36	89	n/a	China	n/a
GCF-000411015.1	160	CC17	33	CC36	89	n/a	China	n/a
GCF-015767695.1	160	CC17	33	CC36	89	n/a	n/a	n/a
GCF-000321845.1	160	CC17	33	CC36	89	2001	Germany	Stool
GCF-026953455.1	160	CC17	33	CC36	89	1991	Germany	Clinical isolate
GCF-900683475.1	160	CC17	33	CC36	89	n/a	n/a	n/a
GCF-000321825.1	159	CC17	40	CC36	602	2001	South Africa	Caecum
GCF-000392165.1	22	CC17	40	CC36	777	1995	Denmark	n/a
GCF-028437725.1	22	CC17	40	CC36		n/a	Peru: Lima	n/a
GCF-000321965.1	22	CC17	40	CC36	484	1960	Netherlands	Pus
GCF-023182815.2	22	CC17	40	CC36		2018	China: Xinjiang	n/a
GCF-900148655.1	22	CC17	1048		4948	n/a	n/a	Stool
GCF-000395785.1	22	CC17	589	CC36	789	2010	Denmark	n/a
GCF-005166365.1	1054		233	CC62	4856	2012	Egypt:Cairo	Infant stool
GCF-023375225.1	416		179	CC184	6807	2021	n/a	Stool
GCF-021122055.1	22	CC17	178		1381	2019	Belgium	Enviromental Sample
GCF-021122425.1	22	CC17	178		1381	2019	Belgium	Enviromental Sample
GCF-021122985.1	22	CC17	178		1381	2019	Belgium	Enviromental Sample
GCF-000336405.1	860		180		18	n/a	n/a	Milk
GCF-000393855.1	860		180		18	n/a	n/a	n/a
GCF-902160955.1	22	CC17	40	CC36	4706	2018	United Kingdom	Stool
GCF-902159925.1	22	CC17	40	CC36	4706	2018	United Kingdom	Stool
GCF-902159555.1	22	CC17	40	CC36	4706	2018	United Kingdom	Stool
GCF-002140215.1	22	CC17	40	CC36	4706	2015	Germany	Stool
GCF-021123545.1	22	CC17	40	CC36	5626	2019	Belgium	Enviromental Sample
GCF-900635365.1	21	CC17	38	CC36	578	n/a	n/a	n/a
GCF-004332055.1	32	CC17	81	CC62	3618	2016	USA:Texas	Animal-cattle-steer
GCF-000321945.1	104		37	CC36	614	1959	Netherlands	n/a
GCF-023375445.1	104		37	CC36	4444	2021	n/a	Stool
GCF-028335225.1	104		37	CC36	4444	2021	n/a	Stool
GCF-028335485.1	104		37	CC36	4444	2021	n/a	Stool
GCF-028335465.1	104		37	CC36	4444	2021	n/a	Stool
GCF-028551565.1	104		37	CC36	4444	2021	n/a	Stool
GCF-009659825.1	32	CC17	268	CC62	6454	2015	USA	Soil
GCF-003320475.1	22	CC17	720	CC36		n/a	n/a	Breast meat
GCF-003320855.1	22	CC17	16	CC2	4710	n/a	USA: Detroit	Breast meat
GCF-902160525.1	22	CC17	16	CC2	4707	2018	United Kingdom	Stool
GCF-902160485.1	22	CC17	16	CC2	4707	2018	United Kingdom	Stool
GCF-003320165.1	21	CC17	712	CC17		n/a	USA: St. Paul	Breast meat
GCF-003320695.1	21	CC17	697	CC17	4705	n/a	USA: Baltimore	Breast meat
GCF-003320115.1	21	CC17	697	CC17	4705	n/a	USA: Little Rock	Breast meat
GCF-023375205.1	21	CC17	20	CC36	6806	2021	n/a	Stool
GCF-902162355.1	21	CC17	176	CC62	6443	2018	United Kingdom	Stool
GCF-002140435.1	2198		642		5034	n/a	Germany	Stool
GCF-001298485.1	21	CC17	205	CC62	78	1992	Germany	n/a
GCF-001412695.1	21	CC17	205	CC62	122	2012	Germany: Wernigerode	Laboratory strain
GCF-023743905.1	2260		62	CC62	6936	2019	Greece: Western Macedonia	Artisanal Greek Kefalograviera cheese
GCF-023743695.1	113	CC17	209	CC62	6932	2019	Greece: Western Macedonia	Artisanal Greek Kefalograviera cheese
GCF-023743655.1	113	CC17	209	CC62	6932	2019	Greece: Western Macedonia	Artisanal Greek Kefalograviera cheese
GCF-023743525.1	113	CC17	209	CC62	6932	2019	Greece: Western Macedonia	Artisanal Greek Kefalograviera cheese
GCF-002140955.1	22	CC17	635	CC184	4947	2015	Germany	Stool

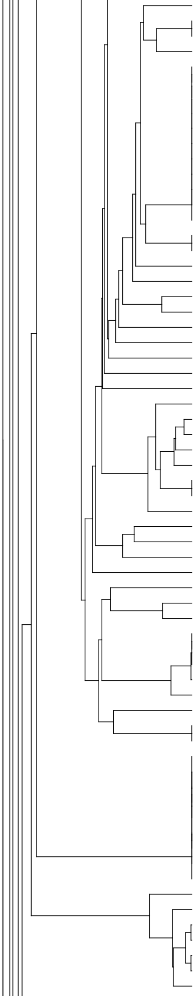

GCF-000415365.1	32	CC17	599	CC36	88	n/a	n/a	n/a
GCF-002140515.1	32	CC17	640	CC36	5057	n/a	Germany: Berlin	Stool
GCF-002141135.1	32	CC17	640	CC36	5057	n/a	Germany: Berlin	Stool
GCF-000767365.1	536	CC17	36	CC36	147	2011	Denmark	n/a
GCF-027679605.1	2170		36	CC36		2014	China: Shenzhen	n/a
GCF-027677565.1	2170		36	CC36		2014	China: Shenzhen	n/a
GCF-027677885.1	2170		36	CC36		2014	China: Shenzhen	n/a
GCF-027678045.1	2170		36	CC36		2014	China: Shenzhen	n/a
GCF-027678105.1	2170		36	CC36		2014	China: Shenzhen	n/a
GCF-027678185.1	2170		36	CC36		2014	China: Shenzhen	n/a
GCF-027678325.1	2170		36	CC36		2014	China: Shenzhen	n/a
GCF-027678505.1	2170		36	CC36		2014	China: Shenzhen	n/a
GCF-027678795.1	2170		36	CC36		2014	China: Shenzhen	n/a
GCF-027679265.1	2170		36	CC36		2014	China: Shenzhen	n/a
GCF-027678305.1	2170		36	CC36		2014	China: Shenzhen	n/a
GCF-002140455.1	32	CC17	641	CC36	5056	n/a	Germany: Brandenberg	Stool
GCF-002140475.1	32	CC17	641	CC36	5056	2015	Germany	Stool
GCF-018279145.1	32	CC17	217	CC36	6783	2019	China:Sichuan	Animal
GCF-021121925.1	420		34	CC36	6881	2019	Belgium	Enviromental Sample
GCF-019731045.1	92	CC17	908	CC36	6872	2020	China	Stool
GCF-000321645.1	92	CC17	566	CC36	584	1998	Netherlands	Stool
GCF-021398505.1	2067		277	CC36	6797	2018	China: Beijing	n/a
GCF-000415305.1	92	CC17	601	CC36	738	n/a	n/a	n/a
GCF-024372785.1	2370		945	CC36		2016	Mexico:Sinaloa	Gut
GCF-002983785.1	683		707	CC36	4841	2014	USA:Washington	Blood
GCF-000415345.1	832		600	CC36	90	n/a	n/a	n/a
GCF-017641685.1	907	CC17	63	CC62	5877	2017	South Africa: KwaZulu-Natal	Clinical isolate
GCF-018517065.1	324	CC17	259	CC62	4439	2017	France: Rennes	Digestive
GCF-025800645.1	324	CC17	259	CC62		2020	China: Jiangsu	Enviromental Sample
GCF-028335265.1	324	CC17	213	CC62		2021	n/a	Stool
GCF-009832485.1	2251	CC17	213	CC62	6819	2017	USA:Pennsylvania	Stool
GCF-002140295.1	2301		644	CC62	5017	2013	USA: Massachusetts	n/a
GCF-002140315.1	2301		644	CC62	5017	n/a	USA: MA, Cape Cod	Stool
GCF-028891525.1	2062	CC17	442	CC2		2021	China:zhumadian	Sewage
GCF-902165245.1	1533		272	CC36	4876	2018	United Kingdom	Stool
GCF-018784845.1	2253		221	CC36	6870	n/a	Ireland	Stool
GCF-010671845.1	2280	CC17	35	CC36	3440	1969	Bulgaria:Sofia	Yoghurt
GCF-902165865.1	2266		212		4880	2018	United Kingdom	Stool
GCF-003320715.1	1973	CC17	722		4475	n/a	USA: Tuscaloosa	Breast meat
GCF-021122805.1	55	CC17	15	CC2	6884	2019	Belgium	Enviromental Sample
GCF-019175425.1	957		245		6787	2020	Switzerland: Grenchen	Stream surface water
GCF-023743755.1	1956		32	CC62	6935	2019	Greece: Epirus	Artisanal Greek Feta cheese
GCF-023744175.1	1956		32	CC62	6935	2019	Greece: Epirus	Artisanal Greek Feta cheese
GCF-023743775.1	1956		32	CC62	6935	2019	Greece: Epirus	Artisanal Greek Feta cheese
GCF-023744235.1	1956		32	CC62	6935	2019	Greece: Epirus	Artisanal Greek Feta cheese
GCF-015554935.1	1956		32	CC62	4636	2016	USA	Stool
GCF-019042295.1	442	CC17	44	CC36	6871	2014	Russia: Orenburg	n/a
GCF-006351785.1	19	CC17	23	CC128	4704	2019	South Korea	Stool
GCF-006337045.1	19	CC17	23	CC128	4704	2019	South Korea	Stool
GCF-023744275.1	2184		98		6931	2019	Greece: Western Macedonia	Artisanal Greek Kefalograviera cheese
GCF-023743935.1	2184		98		6931	2019	Greece: Western Macedonia	Artisanal Greek Kefalograviera cheese
GCF-023743815.1	2184		98		6931	2019	Greece: Western Macedonia	Artisanal Greek Kefalograviera cheese
GCF-023743605.1	2184		98		6931	2019	Greece: Western Macedonia	Artisanal Greek Kefalograviera cheese
GCF-023743555.1	2184		98		6931	2019	Greece: Epirus	Artisanal Greek Feta cheese
GCF-023743455.1	2184		98		6931	2019	Greece: Epirus	Sheep milk
GCF-023743895.1	2184		98		6931	2019	Greece: Western Macedonia	Artisanal Greek Kefalograviera cheese
GCF-023743675.1	2184		98		6931	2019	Greece: Western Macedonia	Artisanal Greek Kefalograviera cheese
GCF-023743635.1	2184		98		6931	2019	Greece: Western Macedonia	Artisanal Greek Kefalograviera cheese
GCF-004300545.1	38		246	CC184	6456	2018	USA:Little Rock	Blood
GCF-003319975.1	38		587	CC184	6455	n/a	USA: Louisville	Breast meat
GCF-003320485.1	38		587	CC184		n/a	USA: Denver	Breast meat
GCF-003320505.1	38		587	CC184		n/a	USA: Denver	Breast meat
GCF-000395745.1	38		587	CC184	718	2010	Denmark	n/a
GCF-000395805.1	38		587	CC184	718	2010	Denmark	n/a
GCF-028335525.1	38		587	CC184		2021	n/a	Stool

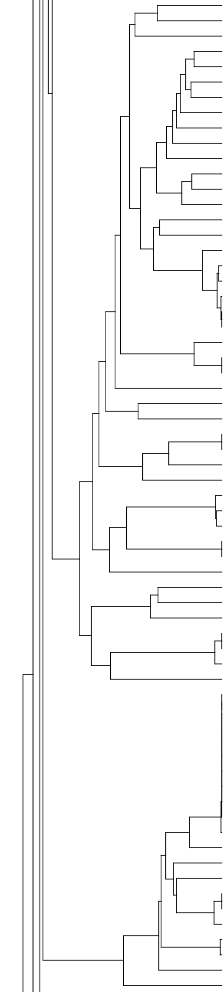

GCF-000322045.1	66	CC17	571	CC60	601	1982	Netherlands	River water
GCF-002850515.1	66	CC17	69		1748	2015	China: Jiangxi	n/a
GCF-027674745.1	822		397	CC60		2014	China: Shenzhen	n/a
GCF-001622975.1	437		613		5003	n/a	Spain	Pig
GCF-003933425.1	822		745	CC60		2017	South Africa: Ozwatini	Rectal swab
GCF-003933435.1	97		298	CC184	4779	2017	South Africa: Ozwatini	Rectal swab
GCF-026204185.1	1630		1041	CC60		2021	China	Rectal swab
GCF-000391745.1	123	CC17	579	CC60	205	1998	Denmark	n/a
GCF-003933355.1	636		1015	CC60	4837	2017	South Africa: Ozwatini	Rectal swab
GCF-027672285.1	97		298	CC184		2014	China: Shenzhen	n/a
GCF-025800625.1	636		1042	CC60		2020	China: Jiangsu	Enviromental Sample
GCF-007923905.2	97		1043	CC73		2017	China: Henan	Chicken
GCF-021122605.1	97		202	CC89	6883	2019	Belgium	Enviromental Sample
GCF-021560195.1	29	CC17	91	CC89	6798	2016	China:Wenzhou	n/a
GCF-009659845.1	2257		269		4932	2015	USA	Collard green
GCF-023375345.1	29	CC17	235	CC60	6808	2021	n/a	Stool
GCF-000395925.1	66	CC17	590		740	1996	Switzerland: Geneve	n/a
GCF-000395525.1	636		583	CC60	767	1994	France: Hopital Saint-Louis	n/a
GCF-000395505.1	636		583	CC60	721	1994	France: Aix en Provence	n/a
GCF-000394495.1	636		583	CC60	726	1989	France: Hopital E. Herriot	n/a
GCF-000395445.1	636		583	CC60	726	1994	France: Aix en Provence	n/a
GCF-000395465.1	636		583	CC60	726	1994	France: Aix en Provence	n/a
GCF-007923925.2	1660		1045	CC36		2017	China: HeBei	Chicken
GCF-002973675.1	650		72	CC60	240	2016	France: Chambery	Urine
GCF-018517085.1	650		72	CC60	240	2016	France: Chambery	Urine
GCF-016743855.1	868		71	CC73	2913	2017	China:henan	Swine
GCF-000767345.1	2265	CC17	68	CC60	768	2011	Denmark	n/a
GCF-000322005.1	66	CC17	570	CC36	482	1979	Netherlands	Gut
GCF-902165525.1	524		99	CC89	3120	2018	United Kingdom	Stool
GCF-902158975.1	524		99	CC89	3120	2018	United Kingdom	Stool
GCF-023375405.1	153		240		6811	2021	n/a	Stool
GCF-021124145.1	1146		240		6887	2019	Belgium	Enviromental Sample
GCF-026948645.1	168	CC17	1017	CC62		2010	Belgium: Edegem	Blood
GCF-023299725.1	168	CC17	1017	CC62	431	2022	n/a	Pet food
GCF-018517045.1	168	CC17	1017	CC62	324	2017	France: Amiens	Urine
GCF-002141005.1	372	CC17	633	CC36	5001	n/a	Brazil	Stool
GCF-002141035.1	372	CC17	633	CC36	5001	n/a	Brazil	Stool
GCF-022464235.1	2259		60	CC60	6904	2014	Israel: Jerusalem	Blood
GCF-003320635.1	148	CC17	721	CC60	618	n/a	n/a	Breast meat
GCF-000391985.1	82		580	CC60	534	1997	Denmark	n/a
GCF-006351845.1	1091		230	CC60	4858	2015	USA:Maryland	Chicken
GCF-003320735.1	2250		186	CC184		n/a	USA: Fairfax	Breast meat
GCF-003320245.1	2250		186	CC184		n/a	USA: Chelsea	Breast meat
GCF-006280355.1	2250		186	CC184	4929	2018	USA:Delaware	Chicken
GCF-019731315.1	1577		214	CC73	6874	2020	China	Stool
GCF-004328235.1	2281		41		6449	2016	USA	n/a
GCF-004328225.1	2281		41		6449	2016	USA	n/a
GCF-004332255.1	2281		41		6449	2016	USA	n/a
GCF-004328245.1	2281		41		6449	2016	USA	n/a
GCF-002803675.1	2281		41		6449	2016	USA	Stool
GCF-004332275.1	2281		41		6449	2016	USA	n/a
GCF-004328255.1	2281		41		6449	2016	USA	n/a
GCF-004099895.1	2281		41		6449	2013	USA	n/a
GCF-004099785.1	2281		41		6449	2013	USA	n/a
GCF-004328385.1	2281		41		6449	2016	USA	n/a
GCF-002141075.1	27		645	CC36	5054	2015	Germany	Stool
GCF-000322165.1	27		574		622	2001	Denmark	Poultry
GCF-028335205.1	2103		462			2021	n/a	Stool
GCF-000321485.1	27		490		508	1995	Netherlands	Ascites
GCF-000321785.1	27		490		508	1995	Belgium	n/a
GCF-028437655.1	27		490			n/a	Peru: Lima	n/a
GCF-000395765.1	417		588		712	2010	Denmark	n/a
GCF-000393435.1	417		588		712	n/a	n/a	n/a
GCF-002140735.1	27		639		5055	n/a	USA: Montana	Stool
GCF-002006745.1	1046		621		5012	2008	Mexico:Jalisco	Handmade Mexican Cotija cheese

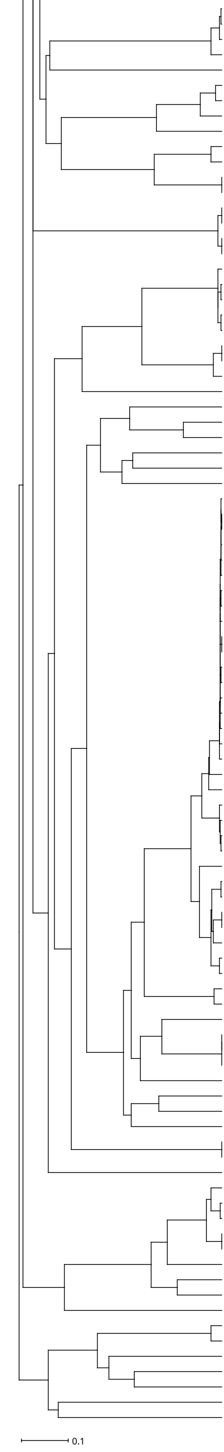

GCF-003320775.1	1950	355		4890	n/a	USA: Tampa	Breast meat
GCF-003320755.1	1950	355		4890	n/a	USA: Pennsylvania	Breast meat
GCF-003320665.1	1950	355		4902	n/a	USA: Chicago	Breast meat
GCF-003320595.1	1950	355			n/a	USA: Dallas	Breast meat
GCF-003320215.1	2315	713		4871	n/a	USA: Phoenix	Breast meat
GCF-003320135.1	2308	665	CC184	4898	n/a	USA: Baltimore	Breast meat
GCF-003320255.1	2308	665	CC184		n/a	USA: Boise	Breast meat
GCF-003319985.1	2194	494		4794	n/a	USA: Phoenix	Breast meat
GCF-003320315.1	2317	494		6476	n/a	USA: Boston	Breast meat
GCF-003320055.1	785	598			n/a	USA: Little Rock	Breast meat
GCF-000393415.1	785	598		719	n/a	n/a	n/a
GCF-000395725.1	784	383		5028	2010	Denmark	n/a
GCF-000395705.1	784	383		5028	2010	Denmark	n/a
GCF-902165965.1	253	80	CC36	4792	2018	United Kingdom	Stool
GCF-902164105.1	253	80	CC36	4792	2018	United Kingdom	Stool
GCF-902159355.1	253	80	CC36	2443	2018	United Kingdom	Stool
GCF-902159365.1	253	80	CC36	2443	2018	United Kingdom	Stool
GCF-023744215.1	545	90	CC89	6937	2019	Greece: Western Macedonia	Artisanal Greek Kefalograviera cheese
GCF-023743575.1	545	90	CC89	6930	2019	Greece: Epirus	Artisanal Greek Feta cheese
GCF-023743415.1	545	90	CC89	6930	2019	Greece: Epirus	Artisanal Greek Feta cheese
GCF-023744135.1	545	90	CC89	6929	2019	Greece: Epirus	Artisanal Greek Feta cheese
GCF-023743405.1	545	90	CC89	6929	2019	Greece: Epirus	Artisanal Greek Feta cheese
GCF-900143355.1	59	90	CC89	5060	n/a	n/a	Stool
GCF-900148665.1	59	90	CC89	5060	n/a	n/a	Stool
GCF-018219285.1	59	90	CC89	5918	2016	USA: Ann Arbor	Rectal swab
GCF-007625025.1	70	260		1417	2016	Russia: Orenburg	Intestines
GCF-004099795.1	130	236	CC89	4722	2013	USA	n/a
GCF-015554345.1	286	234	CC89	4635	2016	USA	Stool
GCF-009735525.1	1449	92	CC89	4866	n/a	n/a	Kefir grains
GCF-000804415.1	156	608	CC89	94	2011	Denmark	n/a
GCF-000321565.1	150	565	CC89	636	n/a	Belgium	n/a
GCF-900637035.1	1034	88	CC89	4855	n/a	n/a	n/a
GCF-000392195.1	6	242		128	1995	Denmark	n/a
GCF-000391945.1	6	242		128	1997	Denmark	n/a
GCF-000392125.1	6	242		128	1995	Denmark	n/a
GCF-000395425.1	6	242		128	2002	Denmark	n/a
GCF-000391885.1	6	242		128	2001	Denmark	n/a
GCF-000391825.1	6	242		128	2001	Denmark	n/a
GCF-000392105.1	6	242		128	1999	Denmark	n/a
GCF-000392025.1	6	242		128	1998	Denmark	n/a
GCF-900634805.1	6	242		128	n/a	n/a	n/a
GCF-000396725.1	6	242		128	2008	USA: Michigan	n/a
GCF-000396705.1	6	242		128	2008	USA: Michigan	n/a
GCF-000392145.1	6	242		128	1995	Denmark	n/a
GCF-000392045.1	6	242		128	1998	Denmark	n/a
GCF-000392005.1	6	242		128	1997	Denmark	n/a
GCF-000391845.1	6	242		128	2001	Denmark	n/a
GCF-002442445.1	6	242		128	2012	Netherlands	Stool
GCF-000391905.1	6	242		128	2002	Denmark	n/a
GCF-000396765.1	6	242		128	2008	USA: Michigan	n/a
GCF-000391965.1	6	242		743	1997	Denmark	n/a
GCF-000804385.1	5	242		92	2011	Denmark	n/a
GCF-026947615.1	6	242		6177	2017	Belgium: Eeklo	Stool
GCF-026947645.1	6	242		6177	2017	Belgium: Eeklo	Stool
GCF-026947705.1	6	242		6177	2017	Belgium: Eeklo	Stool
GCF-026947945.1	6	242		6177	2017	Belgium: Eeklo	Stool
GCF-000391925.1	147	578	CC89	586	2003	Denmark	n/a
GCF-000396685.1	5	86	CC89	749	2008	USA: Michigan	n/a
GCF-000396785.1	5	86	CC89	749	2008	USA: Michigan	n/a
GCF-000392065.1	185	243	CC89	648	1998	Denmark	n/a
GCF-000391865.1	185	243	CC89	648	2001	Denmark	n/a
GCF-000321605.1	5	243	CC89	549	n/a	Spain	n/a
GCF-900638815.1	5	85	CC89	21	n/a	n/a	n/a
GCF-000321745.1	5	85	CC89	21	2002	Netherlands	Stool
GCF-000397025.1	88	547		759	1996	Switzerland: Geneve	n/a
GCF-000394555.1	88	547		782	1994	Switzerland: Geneve	n/a
GCF-021123205.1	1864	241	CC89	6885	2019	Belgium	Enviromental Sample
GCF-026203655.1	2165	89	CC89		2021	China	Rectal swab
GCF-026203285.1	2165	89	CC89		2021	China	Rectal swab
GCF-026204135.1	2165	89	CC89		2021	China	Rectal swab
GCF-900639705.1	1147	244	CC89	563	n/a	n/a	n/a
GCF-000395565.1	1714	363	CC89	775	1994	France: Hopital Saint-Louis	n/a
GCF-027662275.1	1122	1051	CC89		2014	China: Shenzhen	n/a
GCF-900638765.1	1141	198		673	n/a	n/a	n/a
GCF-900143345.1	672	624	CC89	5007	n/a	n/a	Stool
GCF-900148565.1	672	624	CC89	5007	n/a	n/a	Stool
GCF-023743585.1	1853	199		6933	2019	Greece: Western Macedonia	Artisanal Greek Kefalograviera cheese
GCF-003320455.1	54	674		4753	n/a	USA: Tuscaloosa	Breast meat
GCF-003320575.1	54	674		4750	n/a	USA: Dallas	Breast meat
GCF-003320795.1	54	723		4750	n/a	USA: Fairfax	Breast meat
GCF-002140255.1	54	643		5059	n/a	Brazil	Stool
GCF-002140375.1	54	643		5059	n/a	Brazil	Stool
GCF-900066025.1	54	97		565	1998	n/a	Stool
GCF-000321705.1	127	559		488	n/a	Spain	Blood
GCF-000415265.2	127	330		758	n/a	n/a	n/a
GCF-000157555.1	52	548		82	n/a	n/a	n/a
GCF-000322285.1	69	500		510	2002	Netherlands	Blood
GCF-000322025.1	69	500		620	1981	Netherlands	River water
GCF-015546985.1	2252	273		4627	2016	USA	Stool
GCF-902161815.1	512	96		4829	2018	United Kingdom	Stool
GCF-020514405.1	2258	95		6879	2012	Croatia: Zagorje	Soft fresh cheese
GCF-000396805.1	771	592		742	1994	n/a	n/a
GCF-000295275.2	649	936		781	n/a	n/a	n/a
